# Supplementary material for: Roles of SmeYZ, SbiAB, and SmeDEF Efflux Systems in Iron Homeostasis of Stenotrophomonas maltophilia
Source: Microbiol Spectr. 2022 Jun 1;10(3):e02448-21. doi: 10.1128/spectrum.02448-21 (PMC9241820; doi:10.1128/spectrum.02448-21)
Supplement: SUPPLEMENTAL FILE 1 — Supplemental material. Download spectrum.02448-21-s001.pdf, PDF file, 1 MB [file spectrum.02448-21-s001.pdf]

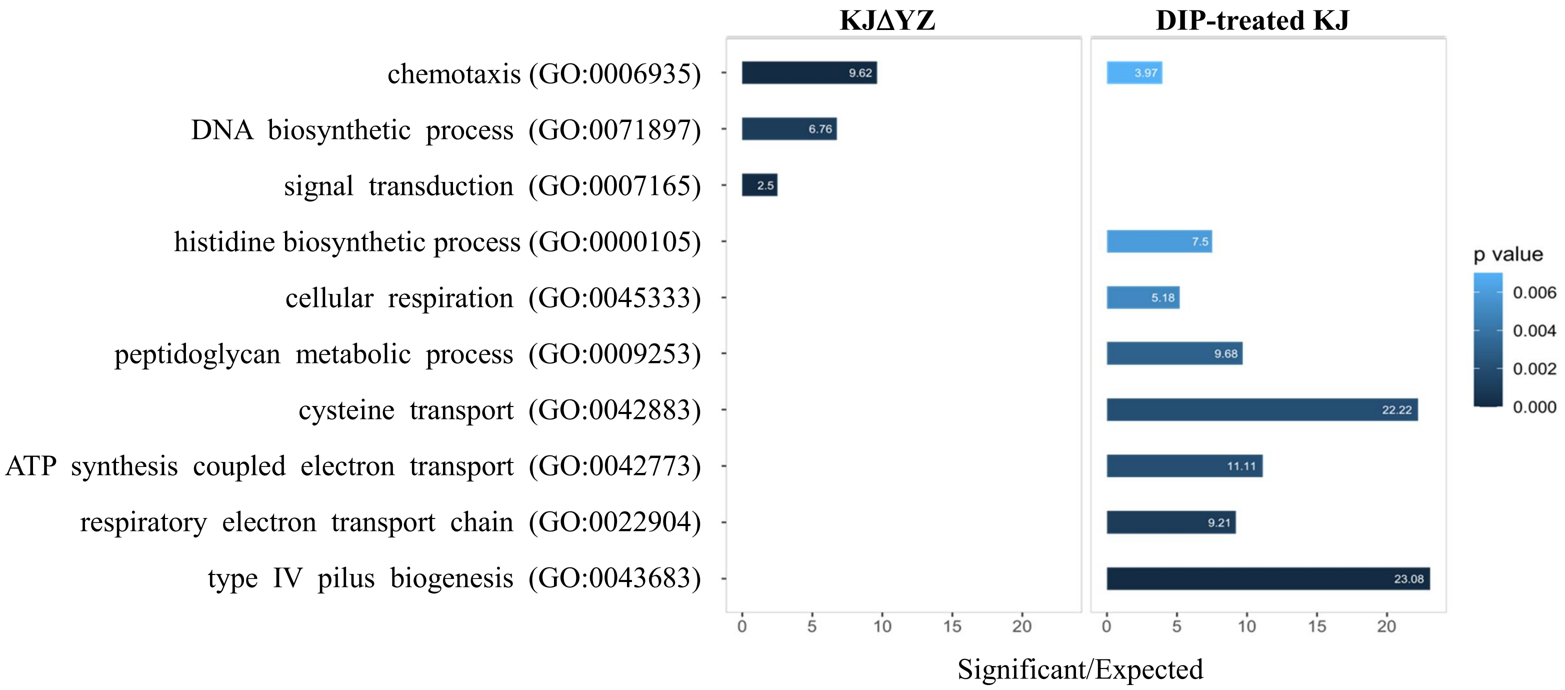

Fig. S1. Gene ontology classification of downregulated genes in KJΔYZ and DIP-treated KJ. Gene enrichment analysis on the downregulated genes was performed using topGO package with Fisher exact test and weighted 01 algorithm. The GO terms with $p < 0.01$ were selected as significantly enriched functional groups. The bar showed the fold enrichment of the GO term.

(A) **(B)**

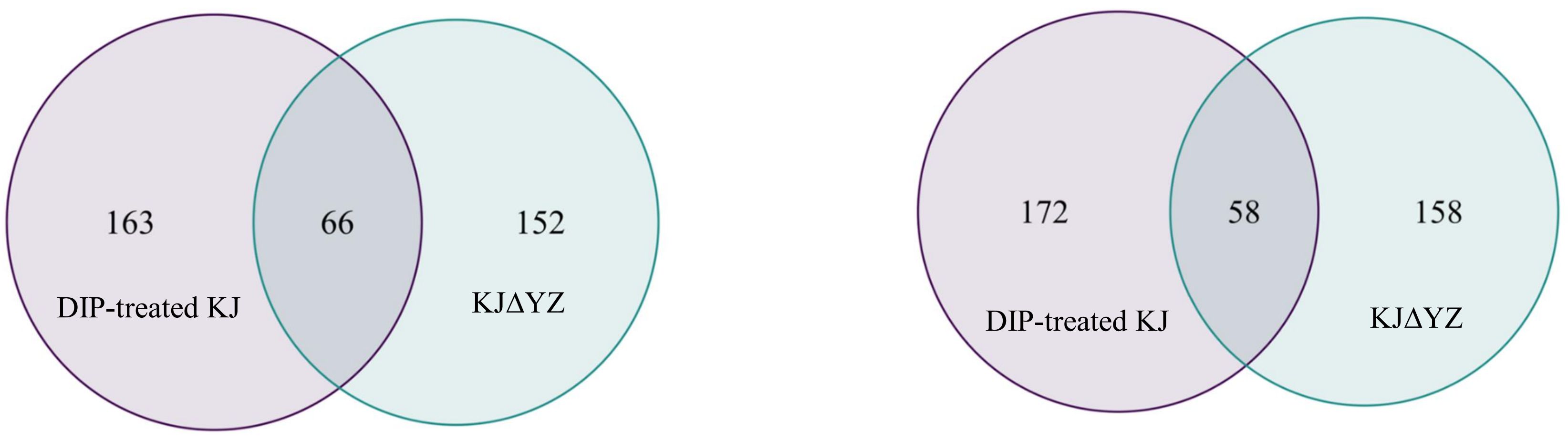

Fig. S2. Venn diagram of KJΔYZ and DIP-treated KJ differentially expressed genes. The differentially expressed genes (DEGs) in KJΔYZ and DIP-treated KJ were counted and plotted using venn.diagram function in VennDiagram package. Venn diagram showing the number of genes with ≥ 3-fold expression change in KJΔYZ (green) and DIP-treated KJ (purple). (A) upregulated DEGs. (B) downregulated DEGs.

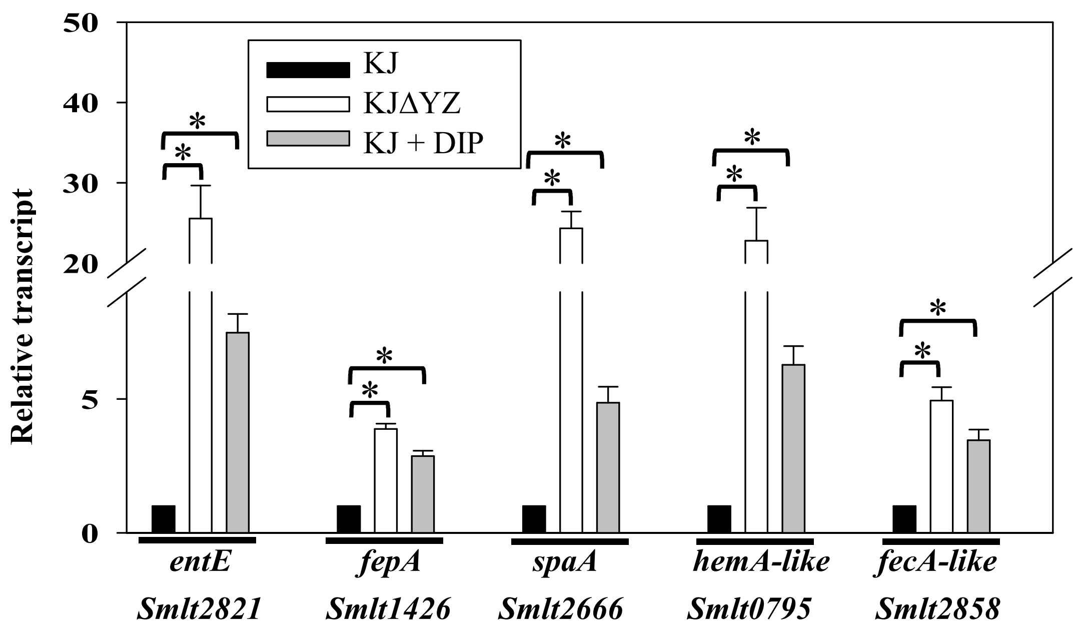

Fig. S3. Determination of *entC*, *fepA, spaA*, *Smlt0795*, and *Smlt2858* expression in KJ, KJΔYZ, and DIP-treated KJ by qRT-PCR. DNA-free RNA was isolated from logarithmical-phase bacterial cells. The relative fold changes in gene expression were calculated using the 2–ΔΔ*CT* method, with the expression of wild-type KJ as 1. The DIP added was 30 μg/ml. Error bars represent the ±1 standard deviation for triplicates. *, $P < 0.01$, significance calculated by Student's *t* test.

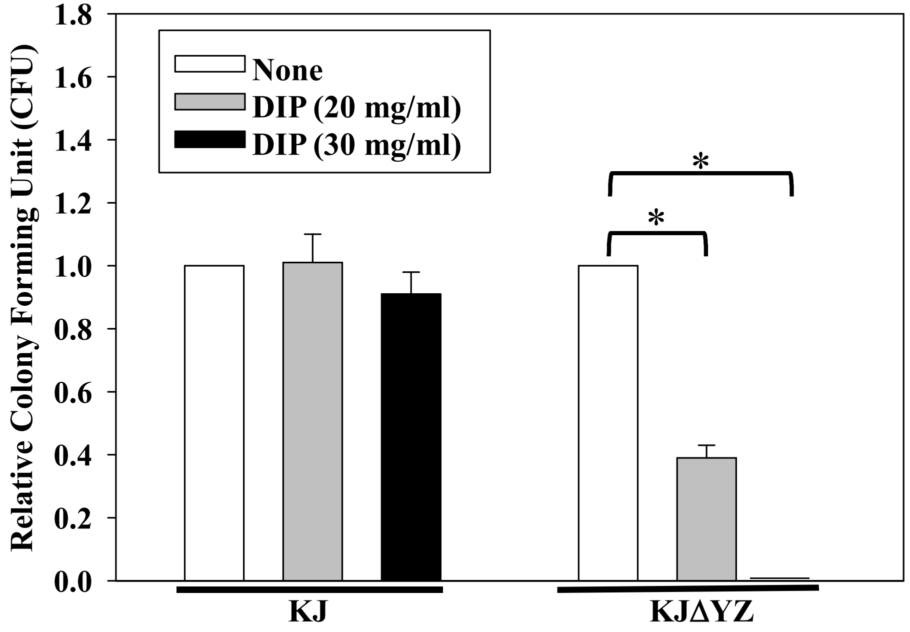

Fig. S4. DIP tolerance of KJ and KJΔYZ. The logarithmically-grown KJ and KJΔYZ cells were serially diluted and spread onto LB agar with or without DIP as indicated to obtain distinct colonies. Bacterial viability was monitored by counting the numbers of CFU. The relative CFU was calculated using the CFU in the DIP-free plate as 1. All experiments were carried out at least three times. Bars represent the average values from three independent experiments. Error bars represent the standard error of the mean. *, $P < 0.001$, significance calculated by Student's t test.

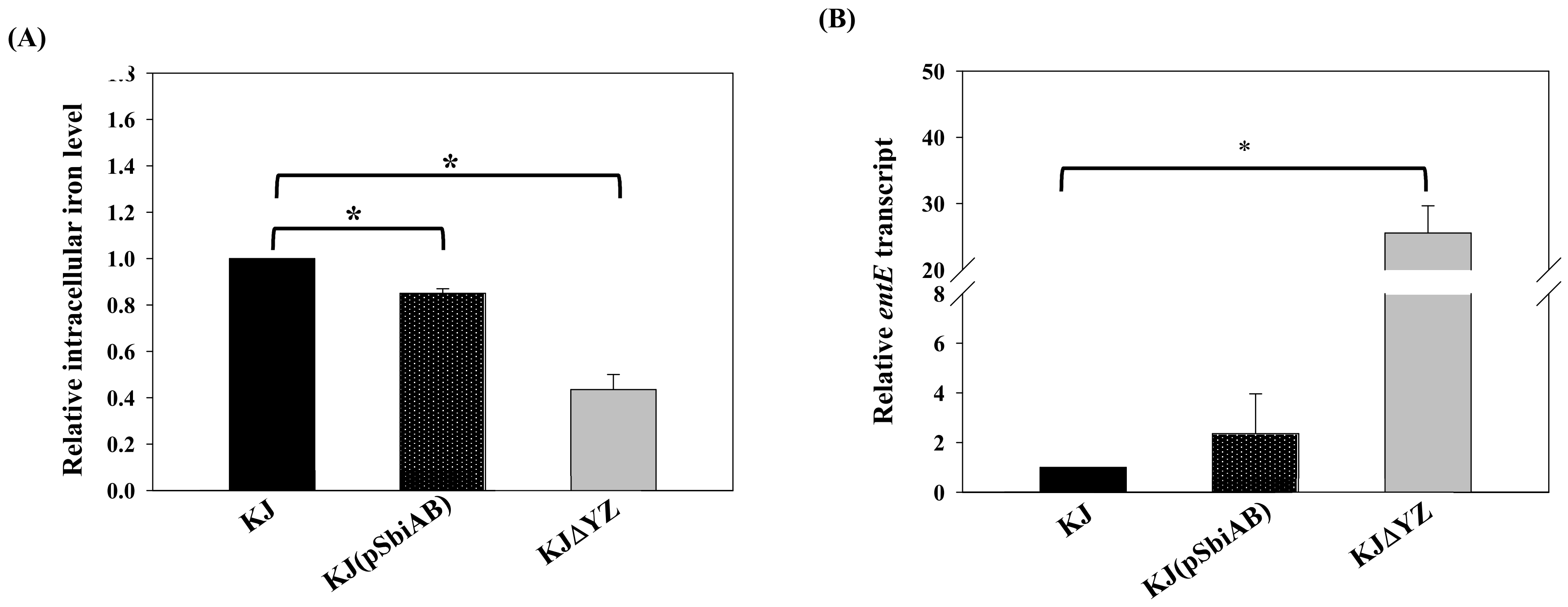

Fig. S5. Impact of *sbiAB* overexpression on intracellular iron level and the expression of stenobactin synthesis genes. Error bars represent the ±1 standard deviation for triplicates. *, $P < 0.01$, significance calculated by Student's *t* test. (A) The intracellular iron levels of wild-type KJ, KJ(pSbiAB), and KJΔYZ. Overnight-cultured bacterial cells were inoculated into fresh LB medium and incubated for 5 h. The amounts of intracellular iron in the strains assayed were determined by inductively coupled plasma mass spectrometry (ICP-MS). The relative iron levels were calculated using the iron level of KJ cells as 1. (B) Determination of *entE* transcript of KJ, KJ(pSbiAB), and KJΔYZ by qRT-PCR. DNA-free RNA was isolated from logarithmical-phase bacterial cells. The relative fold changes in gene expression were calculated using the 2–ΔΔ*CT* method, with the expression of wild-type KJ as 1.

Table S1 Transcriptome analysis of KJΔYZ and DIP-treated KJ compared to wild-type KJ

	TPM[a]			TPM[a]		
**locus_tag**	**KJ**	**Δ*smeYZ***	**Fold[b]**	**KJ (DIP)**	**Fold[b]**	**Product**
Smlt0001	125.09	106.18	-1.18	109.58	-1.14	chromosomal replication initiator protein DnaA
Smlt0002	281.52	329.60	+1.17	377.47	+1.34	DNA polymerase III subunit beta
Smlt0003	0.00	0.00	0	0.00	0	hypothetical protein
Smlt0004	171.65	221.62	+1.29	128.88	-1.33	DNA replication/repair protein RecF
Smlt0005	629.92	1240.66	+1.97	787.04	+1.25	DNA topoisomerase (ATP-hydrolyzing) subunit B
Smlt0006	95.27	163.34	+1.71	133.29	+1.4	CPBP family intramembrane metalloprotease
Smlt0007	239.55	389.57	+1.63	429.50	+1.79	M48 family metallopeptidase
Smlt0008	1317.02	1499.09	+1.14	2292.15	+1.74	tetratricopeptide repeat protein
Smlt0009	606.55	1540.12	+2.54	2934.91	+4.84	TonB, energy transducer
Smlt0010	1264.12	2320.45	+1.84	3833.05	+3.03	ExbB, TonB-system energizer
Smlt0011	1161.73	2457.52	+2.12	3683.60	+3.17	ExbD, biopolymer transporter
Smlt0012	1422.69	2912.36	+2.05	6241.82	+4.39	ExbD, biopolymer transporter
Smlt0013	25.08	27.28	+1.09	46.42	+1.85	cardiolipin synthase
Smlt0014	27.65	23.11	-1.2	70.29	+2.54	hypothetical protein
Smlt0015	123.63	93.48	-1.32	234.04	+1.89	pyridoxine 5'-phosphate synthase
Smlt0016	171.09	212.25	+1.24	70.34	-2.43	hypothetical protein
Smlt0017	0.00	0.00	0	0.00	0	helix-turn-helix domain-containing protein
Smlt0018	0.00	0.00	0	0.00	0	hypothetical protein
Smlt0019	0.00	0.00	0	0.00	0	PA0069 family radical SAM protein
Smlt0020	0.00	0.00	0	0.00	0	hypothetical protein
Smlt0021	0.00	0.00	0	0.00	0	hypothetical protein
Smlt0023	0.00	0.00	0	0.00	0	tyrosine-type recombinase/integrase
Smlt0024	0.00	0.00	0	0.00	0	hypothetical protein
Smlt0025	0.00	0.00	0	0.00	0	hypothetical protein
Smlt0026	0.00	0.00	0	0.00	0	IS3-like element ISStma5 family transposase

Smlt0027	0.00	0.00	0	0.00	0	ISXac3 related insertion element
Smlt0028	0.00	0.00	0	0.00	0	UvrD-helicase domain-containing protein
Smlt0029	0.00	0.00	0	0.00	0	ATP-binding protein
Smlt0032	0.00	0.00	0	0.00	0	MFS transporter
Smlt0033	0.00	0.00	0	0.00	0	hypothetical protein
Smlt0034	0.00	0.00	0	0.00	0	hypothetical protein
Smlt0035	0.00	0.00	0	0.00	0	HupE/UreJ family protein
Smlt0036	0.00	0.00	0	0.00	0	CusA/CzcA family heavy metal efflux RND transporter
Smlt0037	0.00	0.00	0	0.00	0	efflux RND transporter periplasmic adaptor subunit
Smlt0038	0.00	0.00	0	0.00	0	TolC family protein
Smlt0039	0.00	0.00	0	0.00	0	hypothetical protein
Smlt0040	0.00	0.00	0	0.00	0	cyclin-dependent kinase inhibitor 3 family protein
Smlt0041	0.00	0.00	0	0.00	0	conserved hypothetical protein, pseudogene
Smlt0042	0.00	0.00	0	0.00	0	RHS-repeat protein
Smlt0043	0.00	0.00	0	0.00	0	wall-associated protein
Smlt0044	0.00	0.00	0	0.00	0	RHS-repeat protein
Smlt0045	0.00	0.00	0	0.00	0	hypothetical protein
Smlt0046	105.75	158.28	+1.5	243.73	+2.3	2OG-Fe(II) oxygenase
Smlt0047	173.48	222.58	+1.28	198.37	+1.14	class 1 fructose-bisphosphatase
Smlt0048	414.27	718.10	+1.73	598.34	+1.44	aspartate/tyrosine/aromatic aminotransferase
Smlt0049	13.68	22.76	+1.66	26.40	+1.93	TonB-dependent outer membrane receptor
Smlt0050	4.37	3.38	-1.29	12.85	+2.94	hypothetical protein
Smlt0051	6.24	5.00	-1.25	35.87	+5.75	hypothetical protein
Smlt0052	4.91	6.50	+1.32	32.02	+6.52	Ku protein
Smlt0053	10.19	15.76	+1.55	30.90	+3.03	DNA ligase D
Smlt0054	0.00	0.00	0	0.00	0	hypothetical protein
Smlt0055	0.00	0.00	0	0.00	0	alcohol dehydrogenase, pseudogene
Smlt0056	0.00	0.00	0	0.00	0	integrase arm-type DNA-binding domain-containing protein
Smlt0057	0.00	0.00	0	0.00	0	AlpA family transcriptional regulator

Smlt0058	0.00	0.00	0	0.00	0	hypothetical protein
Smlt0059	0.00	0.00	0	0.00	0	ogr/Delta-like zinc finger family protein
Smlt0060	0.00	0.00	0	0.00	0	hypothetical protein
Smlt0061	0.00	0.00	0	0.00	0	hypothetical protein
Smlt0062	0.00	0.00	0	0.00	0	hypothetical protein
Smlt0063	0.00	0.00	0	0.00	0	toprim domain-containing protein
Smlt0064	0.00	0.00	0	0.00	0	DUF927 domain-containing protein
Smlt0065	0.00	0.00	0	0.00	0	hypothetical protein
Smlt0066	0.00	0.00	0	0.00	0	phage portal protein
Smlt0067	0.00	0.00	0	0.00	0	helix-turn-helix domain-containing protein
Smlt0068	0.00	0.00	0	0.00	0	type II toxin-antitoxin system RelE/ParE family toxin
Smlt0069	0.00	0.00	0	0.00	0	hypothetical protein
Smlt0073	78.70	78.90	+1.	100.52	+1.28	bifunctional 4-hydroxy-2-oxoglutarate aldolase/2-dehydro-3-deoxy-phosphogluconate aldolase
Smlt0074	44.65	80.10	+1.79	136.68	+3.06	cysteine dioxygenase family protein
Smlt0075	210.05	304.81	+1.45	197.43	-1.06	LacI family DNA-binding transcriptional regulator
Smlt0076	0.00	0.00	0	0.00	0	immunity 26 domain-containing protein
Smlt0077	578.88	805.78	+1.39	1820.54	+3.14	phenylalanine 4-monooxygenase
Smlt0078	81.60	94.83	+1.16	144.63	+1.77	Lrp/AsnC family transcriptional regulator
Smlt0079	35.06	39.70	+1.13	41.71	+1.19	hypothetical protein
Smlt0080	92.71	73.81	-1.26	118.93	+1.28	patatin-like phospholipase family protein
Smlt0082	54.27	89.00	+1.64	82.90	+1.53	TIGR00266 family protein
Smlt0083	85.85	101.18	+1.18	45.57	-1.88	TonB-dependent outer membrane receptor
Smlt0084	16.87	15.55	-1.09	35.42	+2.1	phosphatase PAP2 family protein
Smlt0085	2.06	3.12	+1.52	1.81	-1.14	hypothetical protein
Smlt0086	2.13	3.87	+1.82	1.97	-1.08	DUF4785 family protein
Smlt0087	52.65	50.10	-1.05	69.08	+1.31	ATP-binding cassette domain-containing protein
Smlt0088	29.96	56.02	+1.87	57.78	+1.93	fatty acid desaturase
Smlt0089	0.00	0.00	0	0.00	0	EF-hand domain-containing protein

Smlt0090	85.13	75.11	-1.13	133.25	+1.57	formamidopyrimidine-dna glycosylase (fapy-dna glycosylase) (dna-(apurinic or apyrimidinic site) lyase mutm)
Smlt0091	128.57	134.66	+1.05	160.28	+1.25	glucan biosynthesis protein D
Smlt0092	239.79	166.25	-1.44	255.39	+1.07	thymidine kinase
Smlt0093	32.86	29.78	-1.1	33.41	+1.02	sel1 repeat family protein
Smlt0094	61.12	68.02	+1.11	83.46	+1.37	SEL1-like repeat protein
Smlt0095	250.02	209.01	-1.2	341.76	+1.37	UvrD-helicase domain-containing protein
Smlt0096	148.78	128.83	-1.15	187.36	+1.26	GNAT family N-acetyltransferase
Smlt0097	60.13	56.35	-1.07	50.23	-1.2	DUF480 domain-containing protein
Smlt0098	231.33	194.57	-1.19	323.00	+1.4	5'-nucleotidase, lipoprotein e(P4) family
Smlt0099	135.94	141.18	+1.04	168.53	+1.24	orotidine-5'-phosphate decarboxylase
Smlt0101	45.17	36.02	-1.25	56.41	+1.25	hypothetical protein
Smlt0102	151.34	139.48	-1.09	213.56	+1.41	glycerol-3-phosphate 1-O-acyltransferase PlsB
Smlt0103	116.25	164.58	+1.42	160.59	+1.38	tRNA 2-thiocytidine(32) synthetase TtcA
Smlt0104	306.91	265.19	-1.16	352.60	+1.15	recombination-associated protein RdgC
Smlt0105	99.66	144.73	+1.45	155.86	+1.56	M48 family metallopeptidase
Smlt0106	41.13	37.90	-1.09	61.76	+1.5	methylglyoxal synthase
Smlt0107	1.10	2.21	+2.02	1.24	+1.13	response regulator
Smlt0108	14.44	50.28	+3.48	7.65	-1.89	FAD-dependent oxidoreductase
Smlt0109	41.79	63.21	+1.51	30.92	-1.35	glutamate synthase large subunit
Smlt0110	4.21	8.19	+1.95	6.83	+1.62	DNA topoisomerase IB
Smlt0111	25.13	51.69	+2.06	53.91	+2.15	hypothetical protein
Smlt0112	10.85	17.03	+1.57	48.19	+4.44	SDR family oxidoreductase
Smlt0113	49.15	93.00	+1.89	87.64	+1.78	hypothetical protein
Smlt0114	49.44	96.67	+1.96	64.49	+1.3	hypothetical protein
Smlt0115	2.75	4.39	+1.6	3.54	+1.29	serine hydrolase
Smlt0116	24.94	25.58	+1.03	24.33	-1.02	BlaI/MecI/CopY family transcriptional regulator
Smlt0117	13.68	14.85	+1.09	14.04	+1.03	BlaR protein
Smlt0118	0.00	0.00	0	0.00	0	hypothetical protein
Smlt0119	18.67	28.93	+1.55	12.17	-1.53	ribonuclease H-like domain-containing protein

Smlt0120	51.81	92.38	+1.78	38.91	-1.33	DEAD/DEAH box helicase
Smlt0121	72.49	88.16	+1.22	157.31	+2.17	hypothetical protein
Smlt0122	45.91	43.26	-1.06	76.06	+1.66	hypothetical protein
Smlt0123	6.88	27.46	+3.99	19.52	+2.84	hypothetical protein
Smlt0124	12.36	18.13	+1.47	25.24	+2.04	hypothetical protein
Smlt0125	0.00	0.00	0	0.00	0	BatA domain-containing protein
Smlt0126	0.00	0.00	0	0.00	0	DUF58 domain-containing protein
Smlt0127	5.59	19.78	+3.54	16.78	+3.	MoxR family ATPase
Smlt0128	3.94	13.87	+3.52	10.52	+2.67	DUF4159 domain-containing protein
Smlt0129	19.54	90.73	+4.64	51.52	+2.64	TldD/PmbA family protein
Smlt0130	20.71	82.89	+4.	46.87	+2.26	TldD/PmbA family protein
Smlt0131	12.43	39.76	+3.2	32.58	+2.62	TldD/PmbA family protein
Smlt0133	0.00	0.00	0	0.00	0	DUF938 domain-containing protein
Smlt0134	0.00	0.00	0	0.00	0	type II toxin-antitoxin system HipA family toxin
Smlt0135	0.00	0.00	0	0.00	0	helix-turn-helix transcriptional regulator
Smlt0136	103.92	74.14	-1.4	16.09	-6.46	indolepyruvate ferredoxin oxidoreductase family protein
Smlt0137	148.54	197.65	+1.33	64.05	-2.32	hypothetical protein
Smlt0138	45.69	30.03	-1.52	58.55	+1.28	AAA family ATPase
Smlt0139	73.60	155.29	+2.11	109.59	+1.49	ParA family protein
Smlt0140	245.79	334.52	+1.36	303.94	+1.24	histidine phosphatase family protein
Smlt0141	76.13	76.33	+1.	57.06	-1.33	YceI family protein
Smlt0142	58.47	48.80	-1.2	80.51	+1.38	phospholipase D family protein
Smlt0143	118.03	131.39	+1.11	216.33	+1.83	hotdog fold thioesterase
Smlt0144	154.98	106.62	-1.45	185.25	+1.2	1-acyl-sn-glycerol-3-phosphate acyltransferase
Smlt0145	136.40	107.31	-1.27	226.52	+1.66	alpha/beta fold hydrolase
Smlt0146	167.44	177.08	+1.06	229.95	+1.37	tetratricopeptide repeat protein
Smlt0147	236.66	276.22	+1.17	338.07	+1.43	YbhB/YbcL family Raf kinase inhibitor-like protein
Smlt0148	0.00	0.00	0	0.00	0	META domain-containing protein
Smlt0149	245.40	220.24	-1.11	210.48	-1.17	META and DUF4377 domain-containing protein

Smlt0150	16.98	21.14	+1.25	15.02	-1.13	undecaprenyl-diphosphate phosphatase
Smlt0151	112.67	312.77	+2.78	101.88	-1.11	type I glutamate--ammonia ligase
Smlt0152	13.57	91.78	+6.77	16.58	+1.22	P-II family nitrogen regulator
Smlt0153	11.28	63.25	+5.61	12.67	+1.12	ammonium transporter
Smlt0154	104.36	52.57	-1.99	50.96	-2.05	N-acetylmuramoyl-L-alanine amidase
Smlt0155	65.06	84.83	+1.3	75.00	+1.15	MltA domain-containing protein
Smlt0156	174.73	202.83	+1.16	150.20	-1.16	hypothetical protein
Smlt0157	6.12	3.82	-1.6	5.40	-1.13	TonB-dependent outer membrane receptor
Smlt0158	32.09	48.26	+1.5	45.29	+1.41	two-component sensor histidine kinase
Smlt0159	27.11	45.18	+1.67	42.27	+1.56	nitrogen regulation protein NR(I)
Smlt0160	220.10	406.77	+1.85	489.75	+2.23	superoxide dismutase family protein
Smlt0161	447.33	1048.07	+2.34	803.81	+1.8	superoxide dismutase family protein
Smlt0162	108.86	58.19	-1.87	96.39	-1.13	TonB family protein
Smlt0163	259.30	324.15	+1.25	276.19	+1.07	hypothetical protein
Smlt0164	354.29	346.57	-1.02	472.92	+1.33	acetyl-CoA C-acetyltransferase
Smlt0165	76.50	149.31	+1.95	80.15	+1.05	porphyrin biosynthesis protein
Smlt0166	76.24	168.17	+2.21	106.71	+1.4	uroporphyrinogen-III C-methyltransferase
Smlt0167	0.00	0.00	0	0.00	0	uroporphyrinogen-III synthase
Smlt0168	75.07	29.33	-2.56	94.72	+1.26	YiiD C-terminal domain-containing protein
Smlt0169	163.89	118.15	-1.39	167.31	+1.02	hypothetical protein
Smlt0170	235.48	167.91	-1.4	306.33	+1.3	rhodanese-like domain-containing protein
Smlt0171	562.93	710.11	+1.26	826.71	+1.47	protein-export chaperone SecB
Smlt0172	179.52	242.04	+1.35	238.39	+1.33	NAD(P)-dependent glycerol-3-phosphate dehydrogenase
Smlt0173	0.00	0.00	0	0.00	0	nuclear transport factor 2 family protein
Smlt0174	0.00	0.00	0	0.00	0	hypothetical protein
Smlt0175	0.00	0.00	0	0.00	0	conserved hypothetical protein
Smlt0176	0.00	0.00	0	0.00	0	ACR3 family arsenite efflux transporter
Smlt0177	0.00	0.00	0	0.00	0	arsenate reductase ArsC
Smlt0178	40.67	76.13	+1.87	23.88	-1.7	metalloregulator ArsR/SmtB family transcription factor

Smlt0179	18.93	29.90	+1.58	14.43	-1.31	arsenical resistance protein ArsH
Smlt0180	0.00	0.00	0	0.00	0	hypothetical protein
Smlt0181	0.00	0.00	0	0.00	0	L,D-transpeptidase
Smlt0182	0.00	0.00	0	0.00	0	hypothetical protein
Smlt0183	0.00	0.00	0	0.00	0	DUF2268 domain-containing putative Zn-dependent protease
Smlt0184	741.92	1628.48	+2.19	167.60	-4.43	Ax21 family protein
Smlt0185	29.16	42.46	+1.46	20.56	-1.42	helix-turn-helix transcriptional regulator
Smlt0186	51.90	661.04	+12.74	99.64	+1.92	VOC family protein
Smlt0187	356.64	407.18	+1.14	81.36	-4.38	Dyp-type peroxidase
Smlt0188	79.88	65.17	-1.23	8.62	-9.26	hypothetical protein
Smlt0189	9.79	9.34	-1.05	9.66	-1.01	nicotinamide riboside transporter PnuC
Smlt0190	7.92	9.91	+1.25	16.11	+2.04	TonB-dependent outer membrane receptor
Smlt0191	18.23	14.94	-1.22	14.01	-1.3	phosphotransferase
Smlt0192	239.68	647.60	+2.7	140.39	-1.71	glycine zipper 2TM domain-containing protein
Smlt0193	162.87	184.47	+1.13	407.02	+2.5	peptidoglycan-binding protein
Smlt0194	254.55	195.09	-1.3	482.85	+1.9	hypothetical protein
Smlt0195	128.02	182.27	+1.42	172.39	+1.35	response regulator transcription factor
Smlt0196	0.00	0.00	0	0.00	0	transporter substrate-binding domain-containing protein
Smlt0197	33.57	16.35	-2.05	25.80	-1.3	c-type cytochrome
Smlt0198	188.74	215.15	+1.14	110.52	-1.71	electron transfer flavoprotein-ubiquinone oxidoreductase
Smlt0199	146.45	210.06	+1.43	208.61	+1.42	acyl-CoA dehydrogenase family protein
Smlt0200	98.83	71.79	-1.38	95.63	-1.03	tRNA (cytidine(34)-2'-O)-methyltransferase
Smlt0201	5.22	6.98	+1.34	3.58	-1.46	hypothetical protein
Smlt0203	95.92	141.92	+1.48	211.56	+2.21	insulinase family protein
Smlt0204	104.80	141.79	+1.35	167.46	+1.6	DUF4156 domain-containing protein
Smlt0205	348.07	157.47	-2.21	339.87	-1.02	3-oxoacyl-ACP synthase III
Smlt0206	43.16	26.33	-1.64	32.22	-1.34	alpha/beta fold hydrolase
Smlt0207	23.39	5.97	-3.92	14.75	-1.59	YkgJ family cysteine cluster protein
Smlt0208	96.89	204.55	+2.11	107.14	+1.11	AMP-binding protein

Smlt0209	55.58	100.12	+1.8	67.78	+1.22	NAD-dependent epimerase/dehydratase family protein
Smlt0210	17.40	88.53	+5.09	54.60	+3.14	DUF1328 domain-containing protein
Smlt0211	60.52	61.08	+1.01	95.52	+1.58	D-alanine--D-alanine ligase
Smlt0212	125.90	103.73	-1.21	161.22	+1.28	SCP2 sterol-binding domain-containing protein
Smlt0213	118.60	95.55	-1.24	166.23	+1.4	ubiquinone biosynthesis regulatory protein kinase UbiB
Smlt0214	0.00	0.00	0	0.00	0	YaiO family outer membrane beta-barrel protein
Smlt0215	0.00	0.00	0	0.00	0	acetyltransferase
Smlt0216	0.00	0.00	0	0.00	0	glycosyltransferase family 2 protein
Smlt0217	0.00	0.00	0	0.00	0	PIG-L family deacetylase
Smlt0218	0.00	0.00	0	0.00	0	O-methyltransferase
Smlt0219	59.22	38.51	-1.54	62.83	+1.06	pseudouridylate synthase
Smlt0220	36.84	75.79	+2.06	35.23	-1.05	DUF2059 domain-containing protein
Smlt0221	203.67	284.24	+1.4	265.18	+1.3	aminoacyl-tRNA hydrolase
Smlt0222	201.73	208.80	+1.04	161.96	-1.25	lysophospholipid acyltransferase family protein
Smlt0223	207.21	189.91	-1.09	401.46	+1.94	peptidyl-dipeptidase Dcp
Smlt0224	3.71	3.72	+1.	3.18	-1.17	3-dehydroquinate dehydratase
Smlt0225	5.63	6.00	+1.07	2.52	-2.23	hypothetical protein
Smlt0226	69.62	108.01	+1.55	105.63	+1.52	signal peptidase I
Smlt0227	107.97	40.44	-2.67	48.82	-2.21	MFS transporter
Smlt0228	38.03	56.55	+1.49	41.98	+1.1	LysR family transcriptional regulator
Smlt0230	195.76	814.42	+4.16	87.73	-2.23	LysR family transcriptional regulator
Smlt0231	11.47	10.87	-1.06	13.14	+1.15	malate synthase A
Smlt0232	25.13	46.45	+1.85	33.60	+1.34	isocitrate lyase
Smlt0233	131.94	48.02	-2.75	111.92	-1.18	GGDEF domain-containing protein
Smlt0234	0.00	0.00	0	0.00	0	putative peptide modification system cyclase
Smlt0236	0.00	0.00	0	0.00	0	putative peptide maturation dehydrogenase
Smlt0237	193.11	471.05	+2.44	123.48	-1.56	acetyl/propionyl/methylcrotonyl-CoA carboxylase subunit alpha
Smlt0238	209.29	433.27	+2.07	147.40	-1.42	methylcrotonoyl-CoA carboxylase
Smlt0239	329.50	643.03	+1.95	266.53	-1.24	isovaleryl-CoA dehydrogenase

Smlt0240	46.30	60.11	+1.3	43.91	-1.05	TetR/AcrR family transcriptional regulator
Smlt0241	155.59	406.83	+2.61	95.57	-1.63	c-type cytochrome
Smlt0242	24.56	40.04	+1.63	62.96	+2.56	ExeM/NucH family extracellular endonuclease
Smlt0244	25.66	31.80	+1.24	29.11	+1.13	membrane protein
Smlt0245	122.29	188.17	+1.54	114.30	-1.07	alpha/beta hydrolase
Smlt0246	111.01	101.71	-1.09	107.13	-1.04	cell division protein ZapE
Smlt0247	335.94	279.28	-1.2	1496.66	+4.46	ribonucleoside-diphosphate reductase subunit alpha
Smlt0248	703.10	535.70	-1.31	1975.46	+2.81	ribonucleotide-diphosphate reductase subunit beta
Smlt0249	57.39	38.87	-1.48	71.33	+1.24	hypothetical protein
Smlt0250	28.10	18.84	-1.49	35.76	+1.27	LysE family translocator
Smlt0251	15.75	13.87	-1.14	24.36	+1.55	acyl-CoA thioesterase
Smlt0252	29.11	38.03	+1.31	25.17	-1.16	NAD-dependent protein deacetylase
Smlt0253	31.16	12.24	-2.55	38.13	+1.22	NADH:flavin oxidoreductase/NADH oxidase
Smlt0254	32.08	45.79	+1.43	50.48	+1.57	LysR family transcriptional regulator
Smlt0255	102.92	157.31	+1.53	164.59	+1.6	formyltetrahydrofolate deformylase
Smlt0257	54.59	42.86	-1.27	93.20	+1.71	bifunctional nicotinamidase/pyrazinamidase
Smlt0258	178.84	109.93	-1.63	267.79	+1.5	epoxyqueuosine reductase QueH
Smlt0259	0.00	0.00	0	0.00	0	hypothetical protein
Smlt0260	70.69	71.04	+1.	23.10	-3.06	nicotinate phosphoribosyltransferase
Smlt0261	157.07	79.49	-1.98	81.21	-1.93	bifunctional nicotinamide-nucleotide adenylyltransferase/Nudix hydroxylase
Smlt0262	132.91	173.64	+1.31	45.98	-2.89	patatin-like phospholipase family protein
Smlt0263	28.66	12.10	-2.37	29.40	+1.03	XRE family transcriptional regulator
Smlt0264	951.06	863.20	-1.1	6027.74	+6.34	CoA-acylating methylmalonate-semialdehyde dehydrogenase
Smlt0265	3295.84	2174.36	-1.52	4254.29	+1.29	acyl-CoA dehydrogenase family protein
Smlt0266	1019.45	626.76	-1.63	2072.31	+2.03	enoyl-CoA hydratase
Smlt0267	730.69	432.59	-1.69	1782.09	+2.44	enoyl-CoA hydratase/isomerase family protein
Smlt0268	1576.71	1089.35	-1.45	2465.90	+1.56	3-hydroxyisobutyrate dehydrogenase
Smlt0269	24.78	31.44	+1.27	77.74	+3.14	organic hydroperoxide resistance protein

Smlt0270	20.14	19.51	-1.03	30.36	+1.51	cation transporter
Smlt0271	0.00	0.00	0	0.00	0	hypothetical protein
Smlt0272	0.00	0.00	0	0.00	0	hypothetical protein
Smlt0273	137.42	117.97	-1.16	231.67	+1.69	peptidoglycan-binding protein
Smlt0274	88.68	63.39	-1.4	121.83	+1.37	DUF1311 domain-containing protein
Smlt0275	109.37	163.16	+1.49	53.68	-2.04	tRNA dihydrouridine(20/20a) synthase DusA
Smlt0276	723.00	1327.10	+1.84	593.45	-1.22	hypothetical protein
Smlt0277	721.77	905.39	+1.25	382.45	-1.89	response regulator transcription factor
Smlt0278	124.65	128.73	+1.03	104.69	-1.19	sensor histidine kinase
Smlt0279	0.00	0.00	0	0.00	0	conserved hypothetical protein
Smlt0280	103.29	118.17	+1.14	71.52	-1.44	zinc-dependent peptidase
Smlt0281	149.71	356.63	+2.38	232.93	+1.56	hypothetical protein
Smlt0282	126.44	104.31	-1.21	154.37	+1.22	bifunctional biotin--[acetyl-CoA-carboxylase] ligase/biotin operon repressor BirA
Smlt0283	55.72	47.28	-1.18	73.46	+1.32	type III pantothenate kinase
Smlt0284	0.00	0.00	0	0.00	0	SPOR domain-containing protein
Smlt0285	0.00	0.00	0	0.00	0	integrase
Smlt0286	0.00	0.00	0	0.00	0	hypothetical protein
Smlt0287	0.00	0.00	0	0.00	0	conserved hypothetical protein
Smlt0288	0.00	0.00	0	0.00	0	hypothetical protein
Smlt0289	0.00	0.00	0	0.00	0	hypothetical protein
Smlt0290	0.00	0.00	0	0.00	0	toprim domain-containing protein
Smlt0291	0.00	0.00	0	0.00	0	hypothetical protein
Smlt0293	0.00	0.00	0	0.00	0	hypothetical protein
Smlt0294	0.00	0.00	0	0.00	0	ogr/Delta-like zinc finger family protein
Smlt0295	0.00	0.00	0	0.00	0	DNA-binding protein
Smlt0296	0.00	0.00	0	0.00	0	XRE family transcriptional regulator
Smlt0297	0.00	0.00	0	0.00	0	nucleotide-binding protein
Smlt0298	0.00	0.00	0	0.00	0	PIN domain-containing protein

Smlt0299	0.00	0.00	0	0.00	0	phage late control D family protein
Smlt0300	0.00	0.00	0	0.00	0	phage tail protein
Smlt0301	0.00	0.00	0	0.00	0	phage tail tape measure protein
Smlt0302	0.00	0.00	0	0.00	0	GpE family phage tail protein
Smlt0303	0.00	0.00	0	0.00	0	phage tail assembly protein
Smlt0304	0.00	0.00	0	0.00	0	phage major tail tube protein
Smlt0305	0.00	0.00	0	0.00	0	phage tail sheath protein
Smlt0306	0.00	0.00	0	0.00	0	GPW/gp25 family protein
Smlt0307	0.00	0.00	0	0.00	0	phage baseplate assembly protein V
Smlt0308	0.00	0.00	0	0.00	0	hypothetical protein
Smlt0309	0.00	0.00	0	0.00	0	phage tail protein
Smlt0310	0.00	0.00	0	0.00	0	phage tail protein I
Smlt0311	0.00	0.00	0	0.00	0	baseplate J/gp47 family protein
Smlt0312	0.00	0.00	0	0.00	0	phage virion morphogenesis protein
Smlt0313	0.00	0.00	0	0.00	0	phage tail protein
Smlt0314	0.00	0.00	0	0.00	0	hypothetical protein
Smlt0315	0.00	0.00	0	0.00	0	glycoside hydrolase family 19 protein
Smlt0316	0.00	0.00	0	0.00	0	phage holin family protein
Smlt0317	0.00	0.00	0	0.00	0	membrane protein
Smlt0318	0.00	0.00	0	0.00	0	tail protein X
Smlt0319	0.00	0.00	0	0.00	0	head completion/stabilization protein
Smlt0320	0.00	0.00	0	0.00	0	terminase
Smlt0321	0.00	0.00	0	0.00	0	phage major capsid protein, P2 family
Smlt0322	0.00	0.00	0	0.00	0	GPO family capsid scaffolding protein
Smlt0323	0.00	0.00	0	0.00	0	terminase ATPase subunit family protein
Smlt0324	0.00	0.00	0	0.00	0	phage portal protein
Smlt0325	0.00	0.00	0	0.00	0	site-specific DNA-methyltransferase
Smlt0327	0.00	0.00	0	0.00	0	conserved hypothetical protein
Smlt0328	0.00	0.00	0	0.00	0	hypothetical protein

Smlt0329	0.00	0.00	0	0.00	0	NYN domain-containing protein
Smlt0330	0.00	0.00	0	0.00	0	DNA recombinase
Smlt0331	0.00	0.00	0	0.00	0	hypothetical protein
Smlt0332	0.00	0.00	0	0.00	0	hypothetical protein
Smlt0333	0.00	0.00	0	0.00	0	hypothetical protein
Smlt0334	188.23	198.74	+1.06	85.81	-2.19	type II toxin-antitoxin system RelE/ParE family toxin
Smlt0335	166.22	204.28	+1.23	43.51	-3.82	putative addiction module antidote protein
Smlt0336	0.00	0.00	0	0.00	0	hypothetical protein
Smlt0337	10.16	7.14	-1.42	23.36	+2.3	hypothetical protein
Smlt0339	0.00	0.00	0	0.00	0	DUF3011 domain-containing protein
Smlt0340	0.00	0.00	0	0.00	0	ankyrin repeat domain-containing protein
Smlt0341	748.31	585.96	-1.28	946.42	+1.26	arginase
Smlt0342	2482.60	5185.70	+2.09	4372.22	+1.76	entericidin A/B family lipoprotein
Smlt0343	386.49	393.86	+1.02	1083.62	+2.8	CsbD family protein
Smlt0344	4.84	11.50	+2.38	7.40	+1.53	hypothetical protein
Smlt0345	133.42	166.64	+1.25	241.35	+1.81	tryptophan--tRNA ligase
Smlt0346	10.66	19.33	+1.81	17.56	+1.65	hypothetical protein
Smlt0347	119.36	66.96	-1.78	124.87	+1.05	MBL fold metallo-hydrolase
Smlt0348	207.44	195.27	-1.06	322.56	+1.55	M28 family metallopeptidase
Smlt0349	0.00	0.00	0	0.00	0	hypothetical protein
Smlt0350	51.22	59.89	+1.17	79.39	+1.55	MFS transporter
Smlt0351	0.00	0.00	0	0.00	0	ABC transporter permease
Smlt0352	0.00	0.00	0	0.00	0	ATP-binding cassette domain-containing protein
Smlt0353	0.00	0.00	0	0.00	0	hypothetical protein
Smlt0354	0.00	0.00	0	0.00	0	HEAT repeat domain-containing protein
Smlt0355	0.00	0.00	0	0.00	0	hypothetical protein
Smlt0357	14.48	8.91	-1.63	21.97	+1.52	drug/metabolite exporter YedA
Smlt0358	34.80	24.02	-1.45	53.07	+1.53	EamA family transporter RarD
Smlt0359	107.99	35.97	-3.	220.21	+2.04	TonB-dependent outer membrane receptor

Smlt0360	9.06	25.84	+2.85	11.83	+1.31	ABC transporter permease
Smlt0361	7.81	25.72	+3.29	11.46	+1.47	ABC transporter permease
Smlt0362	4.59	16.88	+3.68	7.01	+1.53	ABC transporter ATP-binding protein
Smlt0363	15.69	30.78	+1.96	19.82	+1.26	efflux RND transporter periplasmic adaptor subunit
Smlt0364	1.51	2.32	+1.53	1.23	-1.23	ABC transporter ATP-binding protein
Smlt0365	0.39	0.85	+2.21	0.33	-1.16	ABC transporter permease
Smlt0366	15.02	18.62	+1.24	31.69	+2.11	sensor histidine kinase
Smlt0367	17.38	19.45	+1.12	32.87	+1.89	response regulator transcription factor
Smlt0368	0.00	0.00	0	0.00	0	transposase
Smlt0369	0.00	0.00	0	0.00	0	transposase
Smlt0370	0.00	0.00	0	0.00	0	hypothetical protein
Smlt0371	44.23	53.60	+1.21	7.12	-6.21	cytochrome b
Smlt0372	50.81	41.07	-1.24	27.40	-1.85	catalase family peroxidase
Smlt0373	415.39	364.15	-1.14	548.52	+1.32	M17 family metallopeptidase
Smlt0374	185.03	155.87	-1.19	238.34	+1.29	HAD-IA family hydrolase
Smlt0375	0.00	0.00	0	0.00	0	hypothetical protein
Smlt0376	0.00	0.00	0	0.00	0	hypothetical protein
Smlt0377	39.46	32.23	-1.22	46.25	+1.17	AI-2E family transporter
Smlt0378	198.46	186.42	-1.06	214.84	+1.08	hypothetical protein
Smlt0379	311.35	301.33	-1.03	462.34	+1.48	phage holin family protein
Smlt0380	184.16	187.30	+1.02	309.44	+1.68	hypothetical protein
Smlt0381	123.71	101.24	-1.22	95.93	-1.29	Do family serine endopeptidase
Smlt0382	0.00	0.00	0	0.00	0	hypothetical protein
Smlt0383	203.25	99.72	-2.04	307.94	+1.52	hypothetical protein
Smlt0384	21.54	25.09	+1.16	22.45	+1.04	hypothetical protein
Smlt0385	52.15	80.88	+1.55	44.75	-1.17	DUF1428 domain-containing protein
Smlt0386	14.93	22.85	+1.53	13.82	-1.08	DPP IV N-terminal domain-containing protein
Smlt0387	4927.18	5242.33	+1.06	1354.36	-3.64	Ax21 family protein
Smlt0389	76.63	60.54	-1.27	18.63	-4.11	response regulator transcription factor

Smlt0390	407.72	309.95	-1.32	450.05	+1.1	DUF6165 family protein
Smlt0391	15.75	10.35	-1.52	12.10	-1.3	TorF family putative porin
Smlt0392	19.91	13.60	-1.46	7.74	-2.57	MFS transporter
Smlt0393	22.75	37.29	+1.64	12.50	-1.82	LysR family transcriptional regulator
Smlt0394	223.61	233.94	+1.05	195.88	-1.14	DUF1456 family protein
Smlt0395	5.43	1.88	-2.88	2.74	-1.98	hypothetical protein
Smlt0396	48.76	33.14	-1.47	49.33	+1.01	SDR family NAD(P)-dependent oxidoreductase
Smlt0397	154.31	254.39	+1.65	209.38	+1.36	SPOR domain-containing protein
Smlt0398	277.79	312.28	+1.12	230.62	-1.2	arginine--tRNA ligase
Smlt0399	1.60	2.01	+1.25	0.90	-1.77	DNA repair protein RadC
Smlt0400	5.17	3.32	-1.56	5.73	+1.11	response regulator transcription factor
Smlt0401	89.36	92.81	+1.04	114.77	+1.28	bifunctional phosphopantothenoylcysteine decarboxylase/phosphopantothenate--cysteine ligase CoaBC
Smlt0402	133.61	145.85	+1.09	183.86	+1.38	dUTP diphosphatase
Smlt0403	0.00	0.00	0	0.00	0	phosphomannomutase
Smlt0404	69.59	63.50	-1.1	111.59	+1.6	response regulator transcription factor
Smlt0405	19.01	20.42	+1.07	35.15	+1.85	sensor histidine kinase KdpD
Smlt0406	3.24	5.75	+1.77	10.68	+3.29	potassium-transporting ATPase subunit KdpC
Smlt0407	10.92	16.05	+1.47	18.43	+1.69	potassium-transporting ATPase subunit KdpB
Smlt0408	6.00	6.57	+1.1	21.69	+3.62	potassium-transporting ATPase subunit KdpA
Smlt0410	209.09	207.48	-1.01	355.60	+1.7	hypothetical protein
Smlt0411	180.72	171.43	-1.05	340.79	+1.89	orotate phosphoribosyltransferase
Smlt0412	96.57	82.88	-1.17	141.65	+1.47	exodeoxyribonuclease III
Smlt0413	89.99	65.03	-1.38	96.58	+1.07	MFS transporter
Smlt0414	91.72	82.83	-1.11	70.36	-1.3	hypothetical protein
Smlt0415	53.81	70.73	+1.31	105.36	+1.96	anhydro-N-acetylmuramic acid kinase
Smlt0416	165.12	189.88	+1.15	414.99	+2.51	peptidoglycan DD-metalloendopeptidase family protein
Smlt0417	266.75	386.59	+1.45	231.76	-1.15	tyrosine--tRNA ligase
Smlt0418	103.16	196.40	+1.9	376.63	+3.65	M28 family metallopeptidase

Smlt0419	39.46	41.78	+1.06	69.09	+1.75	hypothetical protein
Smlt0420	109.79	134.57	+1.23	144.26	+1.31	peptidoglycan DD-metalloendopeptidase family protein
Smlt0421	228.07	259.83	+1.14	267.22	+1.17	S41 family peptidase
Smlt0422	24.48	50.90	+2.08	22.87	-1.07	rhomboid family intramembrane serine protease
Smlt0423	151.95	656.46	+4.32	270.90	+1.78	outer membrane protein transport protein
Smlt0424	17.22	16.92	-1.02	15.16	-1.14	nicotinamide riboside transporter PnuC
Smlt0425	0.00	0.00	0	0.00	0	AAA family ATPase
Smlt0426	110.25	73.37	-1.5	155.17	+1.41	bifunctional proline dehydrogenase/L-glutamate gamma-semialdehyde dehydrogenase PutA
Smlt0427	171.50	329.28	+1.92	231.91	+1.35	DUF2244 domain-containing protein
Smlt0428	55.46	157.43	+2.84	40.42	-1.37	cytochrome c oxidase subunit II
Smlt0429	21.76	81.43	+3.74	8.92	-2.44	cytochrome c oxidase subunit I
Smlt0430	12.09	58.27	+4.82	3.91	-3.09	hypothetical protein
Smlt0431	16.84	21.11	+1.25	4.95	-3.4	cytochrome c oxidase assembly protein
Smlt0432	29.18	11.49	-2.54	10.09	-2.89	cytochrome c oxidase subunit 3
Smlt0433	30.30	68.76	+2.27	71.71	+2.37	twin transmembrane helix small protein
Smlt0434	6.65	4.08	-1.63	3.69	-1.8	SURF1 family protein
Smlt0435	78.17	115.16	+1.47	94.29	+1.21	hypothetical protein
Smlt0436	141.98	102.78	-1.38	98.00	-1.45	COX15/CtaA family protein
Smlt0436A	106.35	71.56	-1.49	75.74	-1.4	heme o synthase
Smlt0438	47.27	37.42	-1.26	30.91	-1.53	hypothetical protein
Smlt0439	17.45	18.57	+1.06	19.02	+1.09	bile acid:sodium symporter
Smlt0440	0.00	0.00	0	0.00	0	tyrosine-protein phosphatase
Smlt0441	140.00	114.46	-1.22	128.80	-1.09	DNA primase
Smlt0442	32.01	36.49	+1.14	31.93	-1.	YihY/virulence factor BrkB family protein
Smlt0443	195.79	150.46	-1.3	209.60	+1.07	GatB/YqeY domain-containing protein
Smlt0444	7905.41	4913.01	-1.61	7245.97	-1.09	30S ribosomal protein S21
Smlt0445	337.74	625.89	+1.85	365.92	+1.08	tRNA (adenosine(37)-N6)-threonylcarbamoyltransferase complex transferase subunit TsaD

Smlt0446	15.22	41.34	+2.72	30.79	+2.02	hypothetical protein
Smlt0447	340.56	504.33	+1.48	420.60	+1.24	dihydroneopterin aldolase
Smlt0448	155.71	177.11	+1.14	130.06	-1.2	mechanosensitive ion channel
Smlt0449	90.93	85.14	-1.07	110.92	+1.22	glycoside hydrolase family 3 C-terminal domain-containing protein
Smlt0450	56.06	61.40	+1.1	74.34	+1.33	hypothetical protein
Smlt0451	52.52	124.79	+2.38	67.83	+1.29	PQQ-dependent sugar dehydrogenase
Smlt0452	40.28	29.57	-1.36	40.87	+1.01	membrane protein
Smlt0453	37.93	34.92	-1.09	202.63	+5.34	hypothetical protein
Smlt0454	12.62	19.94	+1.58	21.38	+1.69	amino acid permease
Smlt0455	19.79	25.22	+1.27	35.13	+1.78	L,D-transpeptidase family protein
Smlt0456	16.07	18.91	+1.18	32.28	+2.01	MurR/RpiR family transcriptional regulator
Smlt0458	28.92	33.73	+1.17	29.37	+1.02	dipeptide epimerase
Smlt0459	26.81	30.51	+1.14	39.48	+1.47	SH3 domain-containing protein
Smlt0460	0.00	0.00	0	0.00	0	transglutaminase-like domain-containing protein
Smlt0461	10.00	21.62	+2.16	5.41	-1.85	TonB-dependent outer membrane receptor
Smlt0462	9.12	22.86	+2.51	6.36	-1.43	serine hydrolase
Smlt0463	31.78	46.14	+1.45	33.03	+1.04	M15 family metallopeptidase
Smlt0465	0.00	0.00	0	0.00	0	IS110-like element ISStma6 family transposase
Smlt0466	22.09	15.43	-1.43	16.98	-1.3	hypothetical protein
Smlt0467	231.43	284.49	+1.23	212.19	-1.09	EF-hand domain-containing protein
Smlt0468	479.61	321.06	-1.49	463.57	-1.03	HAD family hydrolase
Smlt0469	103.15	92.55	-1.11	123.30	+1.2	hypothetical protein
Smlt0470	187.52	182.17	-1.03	193.41	+1.03	VOC family protein
Smlt0471	165.32	177.48	+1.07	194.80	+1.18	acetyl-CoA hydrolase/transferase family protein
Smlt0473	33.79	42.28	+1.25	58.27	+1.72	bifunctional [glutamate--ammonia ligase]-adenylyl-L-tyrosine phosphorylase/[glutamate--ammonia-ligase] adenylyltransferase
Smlt0476	72.98	63.32	-1.15	30.47	-2.39	queuosine precursor transporter
Smlt0477	104.94	64.54	-1.63	146.62	+1.4	hypothetical protein
Smlt0478	33.36	19.33	-1.73	57.30	+1.72	hypothetical protein

Smlt0479	38.49	20.21	-1.9	49.82	+1.29	DUF2145 domain-containing protein
Smlt0480	0.00	0.00	0	0.00	0	hypothetical protein
Smlt0481	38.33	51.04	+1.33	55.62	+1.45	mitochondrial fission ELM1 family protein
Smlt0482	15.69	13.09	-1.2	14.22	-1.1	malonic semialdehyde reductase
Smlt0483	48.45	57.22	+1.18	48.86	+1.01	YceI family protein
Smlt0484	17.63	10.97	-1.61	26.88	+1.52	hypothetical protein
Smlt0485	0.00	0.00	0	0.00	0	hypothetical protein
Smlt0487	71.14	378.23	+5.32	567.66	+7.98	ygjH/viuB, ferric reductase
Smlt0488	41.39	211.17	+5.1	367.92	+8.89	PadR family transcriptional regulator
Smlt0490	1391.80	1229.85	-1.13	1810.20	+1.3	pyruvate dehydrogenase (acetyl-transferring), homodimeric type
Smlt0490A	0.00	0.00	0	0.00	0	helicase, pseudogene
Smlt0493	0.00	0.00	0	0.00	0	IS3-like element ISStma9 family transposase
Smlt0494	0.00	0.00	0	0.00	0	ISXac3 like transposase
Smlt0496	0.00	0.00	0	0.00	0	DUF3427 domain-containing protein
Smlt0497	0.00	0.00	0	0.00	0	McrC family protein
Smlt0498	0.00	0.00	0	0.00	0	hypothetical protein
Smlt0498A	0.00	0.00	0	0.00	0	protease
Smlt0499	0.00	0.00	0	0.00	0	hypothetical protein
Smlt0500	46.68	27.42	-1.7	76.50	+1.64	hypothetical protein
Smlt0501	113.70	53.45	-2.13	132.98	+1.17	hypothetical protein
Smlt0502	20.01	18.76	-1.07	28.04	+1.4	hypothetical protein
Smlt0503	10.20	146.64	+14.38	1279.72	+125.49	YncE family protein
Smlt0504	0.00	0.00	0	0.00	0	hypothetical protein
Smlt0505	0.00	0.00	0	0.00	0	hypothetical protein
Smlt0506	1.56	1.89	+1.21	2.40	+1.54	nuclear transport factor 2 family protein
Smlt0507	10.85	7.06	-1.54	27.48	+2.53	alpha/beta hydrolase
Smlt0508	45.93	49.25	+1.07	58.61	+1.28	hypothetical protein
Smlt0509	23.80	26.63	+1.12	33.14	+1.39	hypothetical protein
Smlt0510	52.55	45.76	-1.15	40.30	-1.3	hypothetical protein

Smlt0510A	0.00	0.00	0	0.00	0	hypothetical protein
Smlt0510B	0.00	0.00	0	0.00	0	hypothetical protein
Smlt0512	0.00	0.00	0	0.00	0	polysaccharide deacetylase family protein
Smlt0513	42.30	49.44	+1.17	22.58	-1.87	hypothetical protein
Smlt0514	0.00	0.00	0	0.00	0	hypothetical protein
Smlt0515	5.79	10.97	+1.9	8.41	+1.45	hypothetical protein
Smlt0516	39.92	40.78	+1.02	29.77	-1.34	hypothetical protein
Smlt0517	5.10	5.89	+1.15	7.43	+1.46	CDF family Co(II)/Ni(II) efflux transporter DmeF
Smlt0518	0.00	0.00	0	0.00	0	IS481-like element ISStma1 family transposase
Smlt0519	0.81	1.12	+1.38	0.86	+1.06	MerC domain-containing protein
Smlt0520	5.86	5.40	-1.09	2.71	-2.16	GTP-binding protein
Smlt0521	8.30	12.73	+1.53	4.34	-1.91	ATP-binding cassette domain-containing protein
Smlt0522	10.22	9.80	-1.04	11.36	+1.11	LysR family transcriptional regulator
Smlt0523	1.79	1.41	-1.27	1.20	-1.49	serine hydrolase
Smlt0524	15.52	41.84	+2.7	5.45	-2.85	MFS transporter
Smlt0525	7.23	6.53	-1.11	15.45	+2.14	EAL domain-containing protein
Smlt0526	32.76	16.23	-2.02	38.61	+1.18	D-serine/D-alanine/glycine transporter
Smlt0527	17.66	9.10	-1.94	13.16	-1.34	lytic transglycosylase domain-containing protein
Smlt0528	0.00	0.00	0	0.00	0	NHL repeat protein
Smlt0529	13.84	28.94	+2.09	16.80	+1.21	YciI family protein
Smlt0531	1.64	2.41	+1.47	2.52	+1.54	YciI family protein
Smlt0532	3.56	4.13	+1.16	4.18	+1.17	VOC family protein
Smlt0533	0.00	0.00	0	0.00	0	VOC family protein
Smlt0534	8.87	10.02	+1.13	6.90	-1.29	DUF1428 domain-containing protein
Smlt0535	20.48	23.52	+1.15	40.60	+1.98	sigma-70 family RNA polymerase sigma factor
Smlt0537	122.64	10.43	-11.76	27.88	-4.4	hypothetical protein
Smlt0538	105.66	11.86	-8.91	40.60	-2.6	hypothetical protein
Smlt0539	167.14	24.30	-6.88	61.79	-2.7	hypothetical protein
Smlt0540	128.91	17.98	-7.17	58.49	-2.2	hypothetical protein

Smlt0541	88.42	157.46	+1.78	94.63	+1.07	M28 family metallopeptidase
Smlt0542	15.92	21.85	+1.37	19.61	+1.23	histidine kinase
Smlt0543	15.94	15.00	-1.06	16.05	+1.01	LytTR family DNA-binding domain-containing protein
Smlt0544	6.77	9.40	+1.39	4.53	-1.49	hypothetical protein
Smlt0545	27.22	256.07	+9.41	27.32	+1.	hypothetical protein
Smlt0546	39.00	47.32	+1.21	46.08	+1.18	oxygenase MpaB family protein
Smlt0547	59.11	96.63	+1.63	35.08	-1.68	TetR family transcriptional regulator
Smlt0548	2.49	3.78	+1.52	1.29	-1.93	MFS transporter
Smlt0549	1.54	3.19	+2.07	1.28	-1.2	MFS transporter
Smlt0550	245.64	275.93	+1.12	487.45	+1.98	M13 family metallopeptidase
Smlt0551	42.41	44.24	+1.04	32.76	-1.29	EAL domain-containing protein
Smlt0552	16.33	28.11	+1.72	17.41	+1.07	LysR family transcriptional regulator
Smlt0553	2.57	3.04	+1.18	2.47	-1.04	DoxX family protein
Smlt0554	3.82	2.74	-1.39	3.97	+1.04	dioxygenase
Smlt0555	51.27	66.20	+1.29	65.08	+1.27	Na+/H+ antiporter
Smlt0556	134.44	107.25	-1.25	180.01	+1.34	DUF3297 family protein
Smlt0557	97.61	58.99	-1.65	127.31	+1.3	hypothetical protein
Smlt0558	0.00	0.00	0	0.00	0	IS481-like element ISStma1 family transposase
Smlt0559	212.80	102.48	-2.08	183.92	-1.16	DUF4031 domain-containing protein
Smlt0560	691.36	394.70	-1.75	545.69	-1.27	hypothetical protein
Smlt0561	99.72	16.48	-6.05	83.09	-1.2	flagellar motor protein MotB
Smlt0562	72.93	21.89	-3.33	42.29	-1.72	flagellar motor stator protein MotA
Smlt0563	14.82	45.86	+3.09	7.49	-1.98	peptide-methionine (R)-S-oxide reductase MsrB
Smlt0564	46.47	168.81	+3.63	36.56	-1.27	hypothetical protein
Smlt0566	30.16	19.76	-1.53	16.54	-1.82	Lrp/AsnC ligand binding domain-containing protein
Smlt0567	519.39	90.65	-5.73	606.66	+1.17	D-amino acid dehydrogenase
Smlt0568	396.82	117.62	-3.37	564.60	+1.42	alanine racemase
Smlt0569	279.26	328.79	+1.18	321.38	+1.15	DUF3016 domain-containing protein
Smlt0570	0.00	0.00	0	0.00	0	sensor histidine kinase/response regulator fusion protein

Smlt0571	209.61	398.53	+1.9	186.68	-1.12	DUF3247 family protein
Smlt0572	47.12	33.40	-1.41	226.74	+4.81	hypothetical protein
Smlt0573	73.20	64.99	-1.13	84.33	+1.15	class I SAM-dependent methyltransferase
Smlt0574	121.90	140.55	+1.15	164.47	+1.35	pseudouridine synthase
Smlt0575	121.58	193.87	+1.59	138.09	+1.14	HAD family phosphatase
Smlt0576	248.08	274.90	+1.11	537.52	+2.17	beta-ketoacyl-ACP synthase I
Smlt0577	340.30	207.50	-1.64	454.81	+1.34	3-hydroxyacyl-[acyl-carrier-protein] dehydratase FabA
Smlt0578	22.64	27.73	+1.22	37.27	+1.65	DNA polymerase IV
Smlt0580	39.85	24.63	-1.62	27.16	-1.47	MBL fold metallo-hydrolase
Smlt0581	7.55	3.39	-2.23	10.02	+1.33	NAD(P)H-binding protein
Smlt0582	25.86	36.77	+1.42	24.33	-1.06	LysR family transcriptional regulator
Smlt0583	143.27	78.78	-1.82	116.04	-1.23	class I SAM-dependent methyltransferase
Smlt0584	300.78	255.53	-1.18	291.19	-1.03	phosphoglycolate phosphatase
Smlt0585	85.18	81.75	-1.04	117.36	+1.38	TonB-dependent vitamin B12 receptor
Smlt0586	184.26	153.17	-1.2	206.51	+1.12	histidine phosphatase family protein
Smlt0587	164.29	204.43	+1.24	210.39	+1.28	TfoX/Sxy family protein
Smlt0588	296.90	429.57	+1.45	327.16	+1.1	GAF domain-containing protein
Smlt0589	33.51	28.95	-1.16	36.00	+1.07	dethiobiotin synthase
Smlt0590	25.60	36.69	+1.43	30.91	+1.21	PLP-dependent aminotransferase family protein
Smlt0591	2.55	4.28	+1.68	6.71	+2.63	peptide-methionine (S)-S-oxide reductase MsrA
Smlt0592	2.64	3.24	+1.23	3.62	+1.37	redoxin family protein
Smlt0593	0.39	1.44	+3.68	1.36	+3.46	peptide-methionine (R)-S-oxide reductase MsrB
Smlt0594	35.29	45.29	+1.28	61.33	+1.74	response regulator transcription factor
Smlt0595	13.80	16.92	+1.23	26.98	+1.96	HAMP domain-containing histidine kinase
Smlt0596	17.02	69.36	+4.08	10.58	-1.61	HAMP domain-containing protein
Smlt0597	120.63	337.47	+2.8	64.33	-1.88	winged helix-turn-helix domain-containing protein
Smlt0598	61.40	563.81	+9.18	113.79	+1.85	asparagine synthase (glutamine-hydrolyzing)
Smlt0599	0.00	0.00	0	0.00	0	hypothetical protein
Smlt0600	27.73	8.37	-3.31	14.82	-1.87	phasin family protein

Smlt0601	257.34	254.39	-1.01	237.29	-1.08	6-carboxytetrahydropterin synthase QueD
Smlt0602	11.40	40.65	+3.57	2.51	-4.53	TonB-dependent outer membrane receptor
Smlt0603	2.48	20.76	+8.39	1.40	-1.77	carboxypeptidase A
Smlt0605	565.12	946.15	+1.67	307.84	-1.84	DEAD/DEAH box helicase
Smlt0608	670.49	490.84	-1.37	411.92	-1.63	fumarylacetoacetate hydrolase family protein
Smlt0609	358.16	360.75	+1.01	309.52	-1.16	maleylacetoacetate isomerase
Smlt0610	0.00	0.00	0	0.00	0	UdgX family uracil-DNA binding protein
Smlt0611	48.68	72.63	+1.49	25.61	-1.9	putative DNA modification/repair radical SAM protein
Smlt0612	196.31	70.36	-2.79	60.05	-3.27	PilT/PilU family type 4a pilus ATPase
Smlt0613	240.40	304.59	+1.27	270.10	+1.12	DUF4398 domain-containing protein
Smlt0614	128.39	152.76	+1.19	168.86	+1.32	hypothetical protein
Smlt0615	6007.50	6200.87	+1.03	3558.20	-1.69	YdcH family protein
Smlt0616	611.03	523.07	-1.17	598.19	-1.02	pyridoxal-phosphate dependent enzyme
Smlt0617	377.17	411.57	+1.09	416.27	+1.1	cystathionine gamma-synthase
Smlt0618	0.00	0.00	0	0.00	0	ABC transporter permease
Smlt0619	0.00	0.00	0	0.00	0	ABC transporter ATP-binding protein
Smlt0620	0.00	0.00	0	0.00	0	CatB-related O-acetyltransferase
Smlt0621	0.00	0.00	0	0.00	0	class I SAM-dependent methyltransferase
Smlt0622	0.00	0.00	0	0.00	0	glycoside hydrolase family 99-like domain-containing protein
Smlt0623	0.00	0.00	0	0.00	0	glycosyl transferase
Smlt0624	0.00	0.00	0	0.00	0	hypothetical protein
Smlt0625	0.00	0.00	0	0.00	0	SGNH/GDSL hydrolase family protein
Smlt0626	0.00	0.00	0	0.00	0	hypothetical protein
Smlt0627	0.00	0.00	0	0.00	0	GtrA family protein
Smlt0628	0.00	0.00	0	0.00	0	GtrA family protein
Smlt0629	0.00	0.00	0	0.00	0	NAD(P)/FAD-dependent oxidoreductase
Smlt0630	0.00	0.00	0	0.00	0	NAD-dependent epimerase/dehydratase family protein
Smlt0631	0.00	0.00	0	0.00	0	class I SAM-dependent methyltransferase
Smlt0632	0.00	0.00	0	0.00	0	SDR family oxidoreductase

Smlt0633	0.00	0.00	0	0.00	0	FAD-binding oxidoreductase
Smlt0634	0.00	0.00	0	0.00	0	UbiA family prenyltransferase
Smlt0635	0.00	0.00	0	0.00	0	hypothetical protein
Smlt0636	0.00	0.00	0	0.00	0	glycosyltransferase family 2 protein
Smlt0637	0.00	0.00	0	0.00	0	multidrug transporter
Smlt0638	0.00	0.00	0	0.00	0	TIM barrel protein
Smlt0639	0.00	0.00	0	0.00	0	NAD(P)-dependent oxidoreductase
Smlt0640	0.00	0.00	0	0.00	0	FAD-binding oxidoreductase
Smlt0641	0.00	0.00	0	0.00	0	glycosyltransferase
Smlt0642	0.00	0.00	0	0.00	0	flippase-like domain-containing protein
Smlt0644	0.00	0.00	0	0.00	0	sulfatase-like hydrolase/transferase
Smlt0645	435.75	696.73	+1.6	860.00	+1.97	electron transfer flavoprotein subunit alpha/FixB family protein
Smlt0646	411.80	604.18	+1.47	870.36	+2.11	electron transfer flavoprotein subunit beta/FixA family protein
Smlt0647	902.32	907.40	+1.01	1444.24	+1.6	dTDP-glucose 4,6-dehydratase
Smlt0648	464.43	563.24	+1.21	632.78	+1.36	glucose-1-phosphate thymidylyltransferase RfbA
Smlt0649	292.40	362.44	+1.24	342.23	+1.17	dTDP-4-dehydrorhamnose 3,5-epimerase
Smlt0650	0.00	0.00	0	0.00	0	dTDP-4-dehydrorhamnose reductase
Smlt0651	0.00	0.00	0	0.00	0	hypothetical protein
Smlt0652	377.46	372.71	-1.01	403.29	+1.07	mannose-1-phosphate guanylyltransferase/mannose-6-phosphate isomerase
Smlt0653	358.06	407.01	+1.14	419.11	+1.17	phosphomannomutase/phosphoglucomutase
Smlt0654	0.00	0.00	0	0.00	0	DEAD/DEAH box helicase
Smlt0655	0.00	0.00	0	0.00	0	CoA transferase subunit A
Smlt0656	793.27	863.74	+1.09	961.93	+1.21	CoA transferase subunit B
Smlt0657	142.52	102.21	-1.39	154.92	+1.09	glycoside hydrolase family 99-like domain-containing protein
Smlt0658	0.00	0.00	0	0.00	0	alpha-ketoglutarate-dependent dioxygenase AlkB
Smlt0659	0.00	0.00	0	0.00	0	ABC-type transport auxiliary lipoprotein family protein
Smlt0660	76.13	107.15	+1.41	133.05	+1.75	MCE family protein
Smlt0661	105.35	131.92	+1.25	170.44	+1.62	ABC transporter ATP-binding protein

Smlt0662	45.69	58.58	+1.28	65.67	+1.44	ABC transporter permease
Smlt0663	28.54	33.74	+1.18	45.53	+1.6	threonine/serine exporter family protein
Smlt0664	690.93	1187.02	+1.72	1086.04	+1.57	H-NS histone family protein
Smlt0665	376.49	292.74	-1.29	570.25	+1.51	proline--tRNA ligase
Smlt0666	0.00	0.00	0	0.00	0	IS110-like element ISStma7 family transposase
Smlt0668	200.85	228.50	+1.14	285.02	+1.42	DUF4124 domain-containing protein
Smlt0669	228.90	367.89	+1.61	232.16	+1.01	CDP-diacylglycerol--serine O-phosphatidyltransferase
Smlt0670	0.00	0.00	0	0.00	0	hypothetical protein
Smlt0671	123.84	145.84	+1.18	99.25	-1.25	ribosomal protein S18-alanine N-acetyltransferase
Smlt0672	13.76	14.37	+1.04	15.54	+1.13	EamA family transporter
Smlt0673	173.82	215.85	+1.24	224.72	+1.29	valine--tRNA ligase
Smlt0674	75.22	88.66	+1.18	86.44	+1.15	DNA polymerase III subunit chi
Smlt0675	152.02	171.05	+1.13	142.59	-1.07	leucyl aminopeptidase
Smlt0676	80.34	102.17	+1.27	75.74	-1.06	LPS export ABC transporter permease LptF
Smlt0677	96.37	149.93	+1.56	89.80	-1.07	LPS export ABC transporter permease LptG
Smlt0678	0.00	0.00	0	0.00	0	hypothetical protein
Smlt0679	0.00	0.00	0	0.00	0	RDD family protein
Smlt0680	52.65	58.01	+1.1	68.70	+1.3	site-specific tyrosine recombinase XerD
Smlt0681	112.66	90.95	-1.24	120.41	+1.07	DsbC family protein
Smlt0682	6.21	8.04	+1.29	7.34	+1.18	chitinase
Smlt0684	165.44	171.78	+1.04	196.38	+1.19	phosphoribosylformylglycinamidine synthase
Smlt0685	5.30	31.13	+5.87	5.43	+1.02	cell surface protein
Smlt0686	16.61	82.80	+4.99	12.00	-1.38	S8 family serine peptidase
Smlt0687	118.35	127.98	+1.08	181.38	+1.53	type II secretion system ATPase GspE
Smlt0688	33.84	40.47	+1.2	50.00	+1.48	type II secretion system F family protein
Smlt0689	49.41	62.96	+1.27	62.12	+1.26	type II secretion system major pseudopilin GspG
Smlt0690	51.18	70.76	+1.38	58.88	+1.15	GspH/FimT family pseudopilin
Smlt0691	26.37	37.89	+1.44	47.00	+1.78	prepilin-type N-terminal cleavage/methylation domain-containing protein

Smlt0692	34.54	69.92	+2.02	61.75	+1.79	type II secretion system protein J
Smlt0693	26.35	46.22	+1.75	60.16	+2.28	general secretion pathway protein GspK
Smlt0694	48.95	64.46	+1.32	65.99	+1.35	PilN domain-containing protein
Smlt0695	32.73	36.70	+1.12	52.47	+1.6	general secretion pathway protein GspM
Smlt0696	30.73	37.57	+1.22	45.99	+1.5	hypothetical protein
Smlt0697	100.12	182.82	+1.83	160.29	+1.6	type II secretion system secretin GspD
Smlt0698	0.00	0.00	0	0.00	0	IS110-like element ISStma7 family transposase
Smlt0700	0.00	0.00	0	0.00	0	hypothetical protein
Smlt0701	41.06	52.63	+1.28	37.10	-1.11	glycosyltransferase
Smlt0702	0.00	0.00	0	0.00	0	glycosyltransferase family 2 protein
Smlt0703	82.67	84.88	+1.03	109.32	+1.32	nicotinate phosphoribosyltransferase
Smlt0704	9.27	11.14	+1.2	6.97	-1.33	hypothetical protein
Smlt0706	6748.22	7370.29	+1.09	15134.77	+2.24	fimbrial protein
Smlt0707	1881.07	2325.41	+1.24	2416.38	+1.28	molecular chaperone
Smlt0708	1454.96	2057.05	+1.41	1743.13	+1.2	fimbrial biogenesis outer membrane usher protein
Smlt0709	0.00	0.00	0	0.00	0	type 1 fimbrial protein
Smlt0710	0.00	0.00	0	0.00	0	UDP-N-acetylglucosamine 2-epimerase (non-hydrolyzing)
Smlt0711	0.00	0.00	0	0.00	0	cellulose biosynthesis cyclic di-GMP-binding regulatory protein BcsB
Smlt0712	0.00	0.00	0	0.00	0	glycosyl transferase family protein
Smlt0713	0.00	0.00	0	0.00	0	hypothetical protein
Smlt0714	0.00	0.00	0	0.00	0	DUF4434 domain-containing protein
Smlt0715	63.49	94.63	+1.49	70.82	+1.12	NCS2 family permease
Smlt0716	815.73	1103.84	+1.35	557.94	-1.46	energy-dependent translational throttle protein EttA
Smlt0717	70.46	313.29	+4.45	44.44	-1.59	RcnB family protein
Smlt0718	764.30	972.84	+1.27	956.09	+1.25	serine hydroxymethyltransferase
Smlt0719	0.00	0.00	0	0.00	0	hypothetical protein
Smlt0720	0.00	0.00	0	0.00	0	hypothetical protein
Smlt0721	0.00	0.00	0	0.00	0	hypothetical protein

Smlt0722	69.76	48.77	-1.43	59.44	-1.17	transcriptional regulator NrdR
Smlt0723	152.25	179.95	+1.18	212.26	+1.39	bifunctional diaminohydroxyphosphoribosylaminopyrimidine deaminase/5-amino-6-(5-phosphoribosylamino)uracil reductase RibD
Smlt0724	0.00	0.00	0	0.00	0	hypothetical protein
Smlt0725	105.64	77.18	-1.37	115.04	+1.09	hypothetical protein
Smlt0726	0.00	0.00	0	0.00	0	hypothetical protein
Smlt0727	162.30	253.17	+1.56	129.78	-1.25	riboflavin synthase
Smlt0728	89.16	169.79	+1.9	88.89	-1.	3,4-dihydroxy-2-butanone-4-phosphate synthase
Smlt0729	79.42	47.64	-1.67	67.67	-1.17	6,7-dimethyl-8-ribityllumazine synthase
Smlt0730	284.93	184.76	-1.54	330.72	+1.16	transcription antitermination factor NusB
Smlt0731	64.90	45.10	-1.44	53.07	-1.22	thiamine-phosphate kinase
Smlt0732	0.00	0.00	0	0.00	0	fimbria/pilus periplasmic chaperone
Smlt0733	0.00	0.00	0	0.00	0	type 1 fimbrial protein
Smlt0734	0.00	0.00	0	0.00	0	fimbrial protein
Smlt0735	0.00	0.00	0	0.00	0	fimbrial biogenesis outer membrane usher protein
Smlt0736	0.00	0.00	0	0.00	0	type 1 fimbrial protein
Smlt0737	0.00	0.00	0	0.00	0	acyltransferase family protein
Smlt0738	52.66	81.09	+1.54	107.38	+2.04	FAD-binding protein
Smlt0739	0.00	0.00	0	0.00	0	metal-dependent hydrolase
Smlt0741	71.31	113.63	+1.59	64.93	-1.1	YraN family protein
Smlt0742	126.58	178.17	+1.41	124.14	-1.02	penicillin-binding protein activator
Smlt0743	128.61	86.44	-1.49	154.38	+1.2	16S rRNA (cytidine(1402)-2'-O)-methyltransferase
Smlt0745	0.00	0.00	0	0.00	0	DM13 domain-containing protein
Smlt0746	22.53	48.83	+2.17	27.47	+1.22	NRDE family protein
Smlt0747	688.68	1304.66	+1.89	963.76	+1.4	division/cell wall cluster transcriptional repressor MraZ
Smlt0748	516.41	629.49	+1.22	399.67	-1.29	16S rRNA (cytosine(1402)-N(4))-methyltransferase RsmH
Smlt0749	148.51	229.73	+1.55	189.53	+1.28	cell division protein FtsL
Smlt0750	261.99	343.05	+1.31	391.79	+1.5	cell division protein

Smlt0751	226.04	293.96	+1.3	320.72	+1.42	UDP-N-acetylmuramoyl-L-alanyl-D-glutamate--2,6-diaminopimelate ligase
Smlt0752	170.79	238.19	+1.39	294.94	+1.73	UDP-N-acetylmuramoyl-tripeptide--D-alanyl-D-alanine ligase
Smlt0753	153.78	157.65	+1.03	197.26	+1.28	phospho-N-acetylmuramoyl-pentapeptide-transferase
Smlt0754	194.03	221.22	+1.14	258.08	+1.33	putative lipid II flippase FtsW
Smlt0755	238.65	287.78	+1.21	397.11	+1.66	undecaprenyldiphospho-muramoylpentapeptide beta-N-acetylglucosaminyltransferase
Smlt0756	418.59	443.41	+1.06	599.54	+1.43	UDP-N-acetylmuramate--L-alanine ligase
Smlt0757	426.07	490.09	+1.15	713.71	+1.68	D-alanine--D-alanine ligase
Smlt0758	188.69	234.38	+1.24	304.91	+1.62	cell division protein FtsQ/DivIB
Smlt0759	390.58	467.86	+1.2	612.44	+1.57	cell division protein FtsA
Smlt0760	640.13	963.79	+1.51	908.04	+1.42	cell division protein FtsZ
Smlt0761	443.53	608.71	+1.37	337.07	-1.32	UDP-3-O-acyl-N-acetylglucosamine deacetylase
Smlt0762	22.79	26.81	+1.18	23.50	+1.03	DUF721 domain-containing protein
Smlt0763	1004.18	1560.07	+1.55	826.75	-1.21	M23 family metallopeptidase
Smlt0764	566.81	951.87	+1.68	568.59	+1.	preprotein translocase subunit SecA
Smlt0765	96.12	121.88	+1.27	231.94	+2.41	Nudix family hydrolase
Smlt0766	0.00	0.00	0	0.00	0	DUF4124 domain-containing protein
Smlt0767	43.53	75.66	+1.74	21.07	-2.07	methylenetetrahydrofolate reductase [NAD(P)H]
Smlt0768	2.85	5.29	+1.86	2.66	-1.07	alpha/beta hydrolase
Smlt0769	8.28	9.38	+1.13	15.39	+1.86	histidine kinase
Smlt0770	23.59	25.34	+1.07	41.52	+1.76	LytTR family DNA-binding domain-containing protein
Smlt0771	2.41	2.38	-1.01	4.31	+1.79	amidohydrolase family protein
Smlt0772	78.37	53.23	-1.47	116.00	+1.48	DUF3228 family protein
Smlt0773	119.45	190.18	+1.59	46.26	-2.58	hypothetical protein
Smlt0774	131.07	152.35	+1.16	62.41	-2.1	Cys-tRNA(Pro) deacylase
Smlt0775	2402.61	2701.99	+1.12	2611.83	+1.09	adenosylhomocysteinase
Smlt0777	41.13	75.99	+1.85	104.39	+2.54	prolyl oligopeptidase family serine peptidase
Smlt0778	32.75	56.72	+1.73	52.11	+1.59	phosphoenolpyruvate carboxylase

Smlt0779	64.69	33.47	-1.93	72.52	+1.12	acyl-CoA thioesterase
Smlt0780	477.88	269.35	-1.77	486.71	+1.02	methionine adenosyltransferase
Smlt0781	1.19	1.45	+1.23	0.71	-1.67	lysophospholipid acyltransferase family protein
Smlt0782	1.60	1.66	+1.04	1.23	-1.3	UDP-2,3-diacylglucosamine diphosphatase
Smlt0783	23.21	22.23	-1.04	37.89	+1.63	metal-dependent hydrolase
Smlt0784	0.00	0.00	0	0.00	0	glycosyltransferase family 2 protein
Smlt0785	0.00	0.00	0	0.00	0	hypothetical protein
Smlt0786	43.34	40.29	-1.08	59.56	+1.37	class I SAM-dependent methyltransferase
Smlt0787	77.52	89.54	+1.16	117.78	+1.52	tRNA dihydrouridine synthase DusB
Smlt0788	49.04	26.58	-1.85	34.27	-1.43	hypothetical protein
Smlt0789	0.00	0.00	0	0.00	0	LysR family transcriptional regulator
Smlt0790	0.00	0.00	0	0.00	0	MFS transporter
Smlt0791	133.54	141.67	+1.06	209.86	+1.57	ribokinase
Smlt0792	122.99	88.19	-1.39	140.43	+1.14	nucleoside transporter NupC
Smlt0793	181.43	263.02	+1.45	172.80	-1.05	aldo/keto reductase
Smlt0794	10.05	52.90	+5.26	3312.74	+329.53	HemP, hemin uptake protein
Smlt0795	2.51	86.44	+34.49	1420.40	+566.7	HemA, TonB-dependent outer membrane receptor
Smlt0796	1.13	47.00	+41.56	880.11	+778.21	HemB
Smlt0797	4.47	100.07	+22.37	1050.66	+234.87	HemC
Smlt0798	8.72	21.29	+2.44	11.18	+1.28	FtsX-like permease family protein
Smlt0799	18.97	38.26	+2.02	24.59	+1.3	ATP-binding cassette domain-containing protein
Smlt0800	46.43	72.81	+1.57	50.57	+1.09	arylesterase
Smlt0801	954.17	1396.57	+1.46	792.66	-1.2	response regulator transcription factor
Smlt0802	90.57	108.64	+1.2	86.24	-1.05	HAMP domain-containing histidine kinase
Smlt0803	27.39	40.17	+1.47	61.25	+2.24	GDSL-type esterase/lipase family protein
Smlt0804	53.31	51.68	-1.03	39.73	-1.34	diacylglycerol kinase
Smlt0805	138.53	85.97	-1.61	103.11	-1.34	TerC family protein
Smlt0806	18.25	16.91	-1.08	7.06	-2.58	MFS transporter
Smlt0807	0.00	0.00	0	0.00	0	ISPsy9 like transposase

Smlt0808	0.00	0.00	0	0.00	0	ISPsy9 like transposase integrase
Smlt0809	20.64	21.84	+1.06	30.28	+1.47	LemA family protein
Smlt0810	51.04	81.67	+1.6	74.75	+1.46	TPM domain-containing protein
Smlt0811	21.02	26.89	+1.28	35.70	+1.7	TPM domain-containing protein
Smlt0812	128.04	115.23	-1.11	147.28	+1.15	prolipoprotein diacylglyceryl transferase
Smlt0813	252.93	213.90	-1.18	321.22	+1.27	thymidylate synthase
Smlt0814	132.10	99.26	-1.33	211.47	+1.6	dihydrofolate reductase
Smlt0815	0.00	0.00	0	0.00	0	DUF1287 domain-containing protein
Smlt0816	137.20	147.09	+1.07	196.12	+1.43	symmetrical bis(5'-nucleosyl)-tetraphosphatase
Smlt0817	141.96	161.62	+1.14	204.02	+1.44	Co2+/Mg2+ efflux protein ApaG
Smlt0818	75.11	85.78	+1.14	88.36	+1.18	16S rRNA (adenine(1518)-N(6)/adenine(1519)-N(6))-dimethyltransferase RsmA
Smlt0819	79.47	103.72	+1.31	109.37	+1.38	4-hydroxythreonine-4-phosphate dehydrogenase PdxA
Smlt0820	666.78	1007.54	+1.51	886.50	+1.33	peptidylprolyl isomerase
Smlt0821	191.38	245.06	+1.28	337.55	+1.76	LPS-assembly protein LptD
Smlt0822	65.52	71.14	+1.09	96.05	+1.47	histone deacetylase family protein
Smlt0823	122.26	140.15	+1.15	108.48	-1.13	cob(I)yrinic acid a,c-diamide adenosyltransferase
Smlt0824	268.07	430.61	+1.61	247.98	-1.08	hypothetical protein
Smlt0825	234.48	368.28	+1.57	232.86	-1.01	2-octaprenyl-6-methoxyphenyl hydroxylase
Smlt0826	166.04	222.20	+1.34	143.54	-1.16	UbiH/UbiF family hydroxylase
Smlt0827	70.26	115.76	+1.65	87.07	+1.24	hypothetical protein
Smlt0828	55.42	82.26	+1.48	71.00	+1.28	23S rRNA (cytidine(2498)-2'-O)-methyltransferase RlmM
Smlt0829	31.58	32.48	+1.03	31.09	-1.02	nucleoside deaminase
Smlt0830	11.16	13.00	+1.17	14.51	+1.3	DUF6348 family protein
Smlt0831	32.86	29.26	-1.12	53.78	+1.64	membrane protein
Smlt0832	235.03	237.50	+1.01	232.13	-1.01	glutamine--tRNA ligase/YqeY domain fusion protein
Smlt0833	174.50	291.81	+1.67	250.55	+1.44	DUF2007 domain-containing protein
Smlt0834	290.83	882.02	+3.03	206.42	-1.41	peptide-methionine (S)-S-oxide reductase MsrA
Smlt0835	0.00	0.00	0	0.00	0	ISPsy9 like transposase

Smlt0836	0.00	0.00	0	0.00	0	ISPsy9 like transposase
Smlt0837	184.35	360.50	+1.96	182.83	-1.01	transaldolase
Smlt0838	32.76	68.20	+2.08	39.70	+1.21	nucleoside diphosphate kinase regulator
Smlt0839	89.72	116.71	+1.3	98.05	+1.09	LysR family transcriptional regulator
Smlt0840	126.23	238.93	+1.89	108.29	-1.17	alkyl hydroperoxide reductase subunit F
Smlt0841	244.90	292.95	+1.2	362.37	+1.48	peroxiredoxin
Smlt0842	64.32	73.79	+1.15	72.30	+1.12	peptide chain release factor N(5)-glutamine methyltransferase
Smlt0843	263.97	315.11	+1.19	301.73	+1.14	prolyl aminopeptidase
Smlt0844	24.79	36.94	+1.49	15.32	-1.62	LysR family transcriptional regulator
Smlt0845	2.13	1.70	-1.25	1.39	-1.53	MFS transporter
Smlt0846	0.00	0.00	0	0.00	0	N-formylglutamate amidohydrolase
Smlt0847	166.82	188.72	+1.13	166.63	-1.	NUDIX hydrolase
Smlt0848	93.28	89.15	-1.05	67.36	-1.38	exodeoxyribonuclease IX
Smlt0849	61.53	71.33	+1.16	45.55	-1.35	nitroreductase
Smlt0851	182.81	183.98	+1.01	169.53	-1.08	DUF1631 domain-containing protein
Smlt0852	112.26	194.96	+1.74	144.41	+1.29	NAD(P) transhydrogenase subunit alpha
Smlt0853	34.11	34.93	+1.02	31.36	-1.09	DUF3106 domain-containing protein
Smlt0854	3.23	8.90	+2.76	3.90	+1.21	hypothetical protein
Smlt0855	11.73	19.06	+1.62	16.39	+1.4	RNA polymerase sigma factor
Smlt0856	36.64	92.00	+2.51	77.44	+2.11	NAD(P) transhydrogenase subunit alpha
Smlt0857	77.00	172.31	+2.24	105.86	+1.37	NAD(P)(+) transhydrogenase (Re/Si-specific) subunit beta
Smlt0858	217.93	539.57	+2.48	264.97	+1.22	hypothetical protein
Smlt0859	401.43	578.89	+1.44	538.73	+1.34	putative Fe-S cluster assembly protein SufT
Smlt0860	365.27	542.77	+1.49	512.86	+1.4	branched-chain amino acid aminotransferase
Smlt0861	22.75	33.65	+1.48	17.85	-1.27	S8 family serine peptidase
Smlt0862	448.19	346.81	-1.29	753.12	+1.68	asparaginase domain-containing protein
Smlt0863	22.89	25.75	+1.13	18.28	-1.25	DUF2069 domain-containing protein
Smlt0864	73.87	124.93	+1.69	135.84	+1.84	NAD(P)H:quinone oxidoreductase
Smlt0865	39.27	30.93	-1.27	46.77	+1.19	YihY family inner membrane protein

Smlt0866	90.08	86.84	-1.04	87.53	-1.03	redoxin domain-containing protein
Smlt0868	105.92	117.51	+1.11	94.73	-1.12	polyphosphate kinase 2
Smlt0869	61.18	62.16	+1.02	52.75	-1.16	tetratricopeptide repeat protein
Smlt0870	157.61	102.40	-1.54	98.98	-1.59	peptide chain release factor 1
Smlt0871	141.81	120.25	-1.18	91.14	-1.56	glutamyl-tRNA reductase
Smlt0872	134.60	252.93	+1.88	210.00	+1.56	tetratricopeptide repeat protein
Smlt0873	213.28	346.39	+1.62	284.81	+1.34	lipoprotein insertase outer membrane protein LolB
Smlt0874	476.29	535.40	+1.12	607.30	+1.28	4-(cytidine 5'-diphospho)-2-C-methyl-D-erythritol kinase
Smlt0875	311.24	237.10	-1.31	190.08	-1.64	ribose-phosphate diphosphokinase
Smlt0876	3824.82	4122.23	+1.08	4369.18	+1.14	50S ribosomal protein L25/general stress protein Ctc
Smlt0877	281.01	237.51	-1.18	255.30	-1.1	aminoacyl-tRNA hydrolase
Smlt0878	277.69	197.09	-1.41	259.11	-1.07	redox-regulated ATPase YchF
Smlt0880	0.00	0.00	0	0.00	0	autotransporter outer membrane beta-barrel domain-containing protein
Smlt0881	0.00	0.00	0	0.00	0	response regulator
Smlt0882	0.00	0.00	0	0.00	0	hybrid sensor histidine kinase/response regulator
Smlt0883	0.00	0.00	0	0.00	0	EAL domain-containing response regulator
Smlt0884	0.00	0.00	0	0.00	0	response regulator
Smlt0885	16.74	15.82	-1.06	13.39	-1.25	TonB-dependent outer membrane receptor
Smlt0886	45.02	55.87	+1.24	21.22	-2.12	cyanophycinase
Smlt0887	43.15	43.52	+1.01	21.99	-1.96	isoaspartyl peptidase/L-asparaginase
Smlt0890	0.00	0.00	0	0.00	0	elongation factor Tu
Smlt0891	1787.47	1479.19	-1.21	1107.35	-1.61	preprotein translocase subunit SecE
Smlt0892	1412.36	1301.58	-1.09	782.64	-1.8	transcription termination/antitermination protein NusG
Smlt0894	1978.05	2087.58	+1.06	2102.14	+1.06	50S ribosomal protein L11
Smlt0895	1379.56	1556.39	+1.13	1503.99	+1.09	50S ribosomal protein L1
Smlt0896	2569.50	1917.62	-1.34	3771.15	+1.47	50S ribosomal protein L10
Smlt0897	1688.75	1089.63	-1.55	2054.74	+1.22	50S ribosomal protein L7/L12
Smlt0898	825.24	1002.44	+1.21	934.99	+1.13	DNA-directed RNA polymerase subunit beta

Smlt0899	863.95	1237.99	+1.43	1183.72	+1.37	DNA-directed RNA polymerase subunit beta'
Smlt0901	2544.16	1954.93	-1.3	3610.05	+1.42	30S ribosomal protein S12
Smlt0902	4430.23	3560.45	-1.24	4858.07	+1.1	30S ribosomal protein S7
Smlt0903	1888.70	1634.89	-1.16	1990.75	+1.05	elongation factor G
Smlt0904	0.00	0.00	0	0.00	0	elongation factor Tu
Smlt0905	6493.96	7227.93	+1.11	6064.39	-1.07	30S ribosomal protein S10
Smlt0906	4004.44	4775.23	+1.19	4133.23	+1.03	50S ribosomal protein L3
Smlt0907	2284.40	2958.57	+1.3	2369.92	+1.04	50S ribosomal protein L4
Smlt0908	1355.37	1617.83	+1.19	1382.41	+1.02	50S ribosomal protein L23
Smlt0909	2691.85	3378.76	+1.26	2846.89	+1.06	50S ribosomal protein L2
Smlt0910	2561.79	3299.38	+1.29	2729.16	+1.07	30S ribosomal protein S19
Smlt0911	1005.28	1172.38	+1.17	1005.78	+1.	50S ribosomal protein L22
Smlt0912	1956.50	2276.10	+1.16	2185.76	+1.12	30S ribosomal protein S3
Smlt0913	1643.23	1794.48	+1.09	1546.56	-1.06	50S ribosomal protein L16
Smlt0914	1683.03	2006.47	+1.19	2448.07	+1.45	50S ribosomal protein L29
Smlt0915	1759.24	1984.93	+1.13	2343.93	+1.33	30S ribosomal protein S17
Smlt0916	2487.25	2874.33	+1.16	2513.60	+1.01	50S ribosomal protein L14
Smlt0917	880.33	910.41	+1.03	1217.69	+1.38	50S ribosomal protein L24
Smlt0918	2803.54	2945.16	+1.05	3441.61	+1.23	50S ribosomal protein L5
Smlt0919	1147.69	1235.66	+1.08	1060.55	-1.08	30S ribosomal protein S14
Smlt0920	3125.06	2986.19	-1.05	2827.39	-1.11	30S ribosomal protein S8
Smlt0921	3249.35	3567.80	+1.1	3383.62	+1.04	50S ribosomal protein L6
Smlt0922	2673.21	3389.89	+1.27	3255.70	+1.22	50S ribosomal protein L18
Smlt0923	3863.52	5258.94	+1.36	4697.65	+1.22	30S ribosomal protein S5
Smlt0924	225.40	339.27	+1.51	272.83	+1.21	50S ribosomal protein L30
Smlt0925	2506.75	3380.38	+1.35	3339.29	+1.33	50S ribosomal protein L15
Smlt0926	448.35	592.33	+1.32	461.71	+1.03	preprotein translocase subunit SecY
Smlt0928	2266.10	2128.76	-1.06	1861.42	-1.22	30S ribosomal protein S13
Smlt0929	3154.45	3312.15	+1.05	3686.50	+1.17	30S ribosomal protein S11

Smlt0930	3030.32	3996.17	+1.32	2926.38	-1.04	30S ribosomal protein S4
Smlt0931	1451.09	1967.07	+1.36	1418.87	-1.02	DNA-directed RNA polymerase subunit alpha
Smlt0933	1114.25	1387.20	+1.24	1577.75	+1.42	50S ribosomal protein L17
Smlt0935	125.36	82.04	-1.53	148.24	+1.18	disulfide bond formation protein B
Smlt0936	7.73	5.91	-1.31	7.81	+1.01	TonB-dependent outer membrane receptor
Smlt0937	81.16	60.08	-1.35	108.07	+1.33	3-deoxy-7-phosphoheptulonate synthase class II
Smlt0939	14.63	30.54	+2.09	35.16	+2.4	mechanosensitive ion channel family protein
Smlt0940	90.77	148.29	+1.63	97.22	+1.07	amidase
Smlt0941	110.68	72.71	-1.52	70.70	-1.57	DUF2127 domain-containing protein
Smlt0942	875.29	651.00	-1.34	636.86	-1.37	translational GTPase TypA
Smlt0943	838.09	748.91	-1.12	801.35	-1.05	peptidylprolyl isomerase
Smlt0944	2096.50	1269.47	-1.65	2186.17	+1.04	malate dehydrogenase
Smlt0945	149.81	142.04	-1.05	182.32	+1.22	RluA family pseudouridine synthase
Smlt0947	272.68	291.60	+1.07	879.14	+3.22	propionate--CoA ligase
Smlt0948	137.03	80.88	-1.69	45.47	-3.01	hypothetical protein
Smlt0949	89.82	81.14	-1.11	51.50	-1.74	suppressor of fused domain protein
Smlt0950	121.72	168.43	+1.38	97.05	-1.25	glutathione S-transferase N-terminal domain-containing protein
Smlt0951	178.22	461.46	+2.59	96.39	-1.85	cell wall hydrolase
Smlt0952	10.25	14.95	+1.46	7.63	-1.34	NADPH-dependent 2,4-dienoyl-CoA reductase
Smlt0953	12.93	20.20	+1.56	6.81	-1.9	membrane protein
Smlt0954	109.55	159.86	+1.46	99.79	-1.1	zinc-binding alcohol dehydrogenase family protein
Smlt0955	11175.52	8612.71	-1.3	9216.49	-1.21	OmpA family protein
Smlt0956	281.21	273.70	-1.03	403.85	+1.44	hypothetical protein
Smlt0957	206.36	281.11	+1.36	334.62	+1.62	rRNA pseudouridine synthase
Smlt0958	379.90	664.31	+1.75	718.98	+1.89	hypothetical protein
Smlt0959	138.07	149.05	+1.08	172.59	+1.25	glycine C-acetyltransferase
Smlt0960	83.85	55.58	-1.51	62.11	-1.35	TorF family putative porin
Smlt0961	365.48	348.22	-1.05	406.93	+1.11	L-threonine 3-dehydrogenase
Smlt0962	129.81	181.23	+1.4	199.56	+1.54	S46 family peptidase

Smlt0963	33.78	43.72	+1.29	55.84	+1.65	histidine phosphatase family protein
Smlt0964	87.84	126.62	+1.44	129.65	+1.48	bifunctional tetrahydrofolate synthase/dihydrofolate synthase
Smlt0965	162.85	212.52	+1.31	193.13	+1.19	SPOR domain-containing protein
Smlt0966	157.94	190.06	+1.2	164.18	+1.04	CvpA family protein
Smlt0967	461.22	490.31	+1.06	356.41	-1.29	amidophosphoribosyltransferase
Smlt0968	228.56	253.11	+1.11	250.01	+1.09	ferritin-like domain-containing protein
Smlt0969	0.00	0.00	0	0.00	0	conserved hypothetical protein
Smlt0970	37.46	33.17	-1.13	62.43	+1.67	peptidyl-Asp metalloendopeptidase
Smlt0971	320.36	179.31	-1.79	513.80	+1.6	UDP-2,3-diacylglucosamine diphosphatase
Smlt0972	0.84	1.54	+1.84	0.32	-2.6	phosphatase PAP2 family protein
Smlt0973	0.66	0.80	+1.21	0.96	+1.46	glycosyltransferase family 1 protein
Smlt0974	2.60	1.76	-1.48	2.73	+1.05	TonB-dependent outer membrane receptor
Smlt0975	107.05	77.69	-1.38	129.40	+1.21	exopolyphosphatase
Smlt0976	24.41	29.21	+1.2	41.31	+1.69	polyphosphate kinase 1
Smlt0977	23.72	21.42	-1.11	28.34	+1.2	phosphate regulon sensor histidine kinase PhoR
Smlt0978	24.26	29.18	+1.2	43.92	+1.81	phosphate regulon transcriptional regulator PhoB
Smlt0979	76.72	134.95	+1.76	97.15	+1.27	M48 family metalloprotease
Smlt0980	151.83	173.82	+1.14	171.15	+1.13	glutaredoxin 3
Smlt0981	160.58	178.45	+1.11	155.78	-1.03	carboxymuconolactone decarboxylase family protein
Smlt0982	1690.14	1534.40	-1.1	2571.23	+1.52	isocitrate dehydrogenase
Smlt0983	0.00	0.00	0	0.00	0	cysteine hydrolase
Smlt0984	0.00	0.00	0	0.00	0	LysR family transcriptional regulator
Smlt0985	0.00	0.00	0	0.00	0	flavin reductase family protein
Smlt0986	227.68	219.44	-1.04	308.13	+1.35	Bax inhibitor-1/YccA family protein
Smlt0987	0.00	0.00	0	0.00	0	hypothetical protein
Smlt0988	970.20	1038.69	+1.07	621.11	-1.56	trigger factor
Smlt0989	586.33	844.93	+1.44	461.35	-1.27	ATP-dependent Clp endopeptidase proteolytic subunit ClpP
Smlt0990	1396.52	2164.12	+1.55	1513.52	+1.08	ATP-dependent Clp protease ATP-binding subunit ClpX
Smlt0991	320.74	662.69	+2.07	375.17	+1.17	endopeptidase La

Smlt0992	2267.13	2219.09	-1.02	1688.61	-1.34	HU family DNA-binding protein
Smlt0993	366.65	446.29	+1.22	572.06	+1.56	peptidylprolyl isomerase
Smlt0994	229.08	327.71	+1.43	201.27	-1.14	lytic transglycosylase domain-containing protein
Smlt0995	270.83	444.13	+1.64	326.13	+1.2	hydroxyacylglutathione hydrolase
Smlt0996	180.56	221.72	+1.23	206.19	+1.14	hypothetical protein
Smlt0997	236.84	188.79	-1.25	285.13	+1.2	ribonuclease HI
Smlt0998	224.75	247.53	+1.1	226.01	+1.01	DNA polymerase III subunit epsilon
Smlt0999	71.23	59.95	-1.19	108.74	+1.53	protein phosphatase 2C domain-containing protein
Smlt1001	38.29	4.88	-7.84	153.16	+4.	autotransporter domain-containing protein
Smlt1002	558.30	1089.52	+1.95	531.75	-1.05	DUF6165 family protein
Smlt1003	116.30	103.69	-1.12	237.51	+2.04	glycosyltransferase family 9 protein
Smlt1004	410.05	1085.73	+2.65	719.62	+1.75	3-deoxy-D-manno-octulosonic acid kinase
Smlt1005	196.34	339.81	+1.73	234.58	+1.19	MBL fold metallo-hydrolase
Smlt1006	58.77	163.40	+2.78	106.90	+1.82	nuclear transport factor 2 family protein
Smlt1007	12.09	20.93	+1.73	8.84	-1.37	quaternary ammonium compound efflux SMR transporter SugE
Smlt1009	10.11	14.41	+1.43	7.09	-1.42	autotransporter-associated beta strand repeat-containing protein
Smlt1010	20.89	18.92	-1.1	22.38	+1.07	GNAT family N-acetyltransferase
Smlt1011	0.00	0.00	0	0.00	0	transmembrane protein
Smlt1012	0.00	0.00	0	0.00	0	hypothetical protein
Smlt1013	0.00	0.00	0	0.00	0	DNA adenine methylase
Smlt1014	226.86	401.40	+1.77	283.18	+1.25	DNA polymerase III subunit gamma/tau
Smlt1015	413.86	482.83	+1.17	362.39	-1.14	YbaB/EbfC family nucleoid-associated protein
Smlt1016	167.25	179.42	+1.07	165.48	-1.01	recombination mediator RecR
Smlt1017	47.75	48.07	+1.01	46.20	-1.03	histidine triad nucleotide-binding protein
Smlt1018	611.74	896.85	+1.47	669.46	+1.09	Slp family lipoprotein
Smlt1019	17.71	18.47	+1.04	29.12	+1.64	DUF3488 and transglutaminase-like domain-containing protein
Smlt1020	16.04	9.95	-1.61	24.19	+1.51	DUF58 domain-containing protein
Smlt1021	63.70	45.43	-1.4	54.97	-1.16	AAA family ATPase
Smlt1022	124.92	161.19	+1.29	138.61	+1.11	glycosyltransferase family 39 protein

Smlt1023	53.57	45.25	-1.18	79.90	+1.49	Maf family nucleotide pyrophosphatase
Smlt1024	1192.44	988.86	-1.21	783.07	-1.52	YceD family protein
Smlt1025	7660.62	5323.93	-1.44	8951.22	+1.17	50S ribosomal protein L32
Smlt1026	716.42	608.30	-1.18	570.87	-1.25	ketoacyl-ACP synthase III
Smlt1027	282.52	715.38	+2.53	175.80	-1.61	hypothetical protein
Smlt1028	387.91	280.48	-1.38	475.68	+1.23	ACP S-malonyltransferase
Smlt1029	576.72	489.43	-1.18	828.93	+1.44	3-oxoacyl-ACP reductase FabG
Smlt1030	3199.62	2047.24	-1.56	4878.23	+1.52	acyl carrier protein
Smlt1031	637.49	521.36	-1.22	657.41	+1.03	beta-ketoacyl-ACP synthase II
Smlt1032	98.37	79.48	-1.24	110.16	+1.12	aminodeoxychorismate synthase component I
Smlt1034	133.75	97.29	-1.37	185.71	+1.39	endolytic transglycosylase MltG
Smlt1035	109.47	86.07	-1.27	136.03	+1.24	dTMP kinase
Smlt1036	124.01	122.40	-1.01	175.38	+1.41	DNA polymerase III subunit delta'
Smlt1037	194.24	167.33	-1.16	188.65	-1.03	PilZ domain-containing protein
Smlt1038	130.21	146.73	+1.13	198.25	+1.52	tautomerase family protein
Smlt1039	27.59	20.99	-1.31	26.03	-1.06	phage tail protein
Smlt1040	4.54	5.01	+1.1	5.50	+1.21	tail protein X
Smlt1041	10.63	2.49	-4.27	4.64	-2.29	phage tail protein
Smlt1042	0.00	0.00	0	0.00	0	phage tail tape measure protein
Smlt1043	21.76	7.15	-3.05	7.17	-3.04	phage tail assembly protein
Smlt1044	22.14	8.42	-2.63	10.41	-2.13	phage major tail tube protein
Smlt1045	14.73	7.26	-2.03	6.26	-2.35	phage tail sheath subtilisin-like domain-containing protein
Smlt1046	0.00	0.00	0	0.00	0	hypothetical protein
Smlt1047	0.00	0.00	0	0.00	0	tail fiber domain-containing protein
Smlt1048	4.94	4.55	-1.09	2.43	-2.04	phage tail protein I
Smlt1049	9.08	4.45	-2.04	2.29	-3.96	baseplate J/gp47 family protein
Smlt1050	4.67	1.72	-2.72	1.28	-3.64	GPW/gp25 family protein
Smlt1051	4.34	3.52	-1.23	1.47	-2.95	phage baseplate assembly protein V
Smlt1052	5.84	4.05	-1.44	1.67	-3.5	hypothetical protein

Smlt1053	1.20	2.86	+2.39	0.15	-7.81	hypothetical protein
Smlt1054	5.27	3.42	-1.54	0.30	-17.7	glycoside hydrolase family 104 protein
Smlt1055	6.44	5.69	-1.13	0.58	-11.15	hypothetical protein
Smlt1056	4.22	4.65	+1.1	0.88	-4.8	helix-turn-helix domain-containing protein
Smlt1057	41.82	56.33	+1.35	17.81	-2.35	hypothetical protein
Smlt1058	32.80	77.73	+2.37	5.53	-5.93	hypothetical protein
Smlt1059	0.00	0.00	0	0.00	0	hypothetical protein
Smlt1060	0.00	0.00	0	0.00	0	hypothetical protein
Smlt1061	27.91	36.88	+1.32	5.60	-4.98	toprim domain-containing protein
Smlt1064	428.58	517.28	+1.21	178.86	-2.4	hypothetical protein
Smlt1065	291.24	362.46	+1.24	77.49	-3.76	hypothetical protein
Smlt1066	480.24	582.78	+1.21	243.46	-1.97	DUF2946 domain-containing protein
Smlt1067	32.04	76.50	+2.39	33.19	+1.04	TonB-dependent outer membrane receptor
Smlt1068	23.68	87.23	+3.68	27.72	+1.17	PepSY domain-containing protein
Smlt1069	15.36	22.08	+1.44	19.09	+1.24	MFS transporter
Smlt1070	38.13	73.34	+1.92	65.81	+1.73	DeoR/GlpR family DNA-binding transcription regulator
Smlt1071	44.45	76.65	+1.72	58.93	+1.33	SmQnr family pentapeptide repeat protein
Smlt1072	8.82	18.61	+2.11	6.18	-1.43	class I SAM-dependent methyltransferase
Smlt1073	1.94	1.19	-1.63	1.99	+1.02	glutathione S-transferase family protein
Smlt1074	66.94	94.11	+1.41	63.56	-1.05	LysR family transcriptional regulator
Smlt1075	23.71	17.78	-1.33	7.87	-3.01	PLP-dependent aminotransferase family protein
Smlt1076	13.61	10.21	-1.33	6.87	-1.98	DMT family transporter
Smlt1077	25.68	18.29	-1.4	19.89	-1.29	LysR family transcriptional regulator
Smlt1078	2.78	3.62	+1.3	1.53	-1.82	MFS transporter
Smlt1080	45.61	63.88	+1.4	48.65	+1.07	GFA family protein
Smlt1081	27.57	23.70	-1.16	36.38	+1.32	DUF4105 domain-containing protein
Smlt1082	50.97	36.66	-1.39	64.09	+1.26	alpha/beta hydrolase
Smlt1083	13.41	7.43	-1.81	7.66	-1.75	MFS transporter
Smlt1084	97.58	74.78	-1.3	126.75	+1.3	redox-sensitive transcriptional activator SoxR

Smlt1085	32.51	27.04	-1.2	28.06	-1.16	short chain dehydrogenase
Smlt1086	23.75	44.35	+1.87	19.15	-1.24	LysR family transcriptional regulator
Smlt1087	187.11	125.90	-1.49	223.75	+1.2	pyrroline-5-carboxylate reductase
Smlt1088	29.02	16.69	-1.74	50.81	+1.75	YggS family pyridoxal phosphate-dependent enzyme
Smlt1089	395.28	112.93	-3.5	79.61	-4.97	type IV pilus twitching motility protein PilT
Smlt1090	480.93	149.41	-3.22	75.76	-6.35	PilT/PilU family type 4a pilus ATPase
Smlt1091	74.94	58.76	-1.28	134.18	+1.79	aldo/keto reductase
Smlt1092	6.45	5.92	-1.09	7.66	+1.19	LysR family transcriptional regulator
Smlt1093	0.00	0.00	0	0.00	0	IS110-like element ISStma7 family transposase
Smlt1094	0.00	0.00	0	0.00	0	DUF4287 domain-containing protein
Smlt1095	32.16	10.11	-3.18	10.31	-3.12	YitT family protein
Smlt1096	30.79	30.17	-1.02	51.26	+1.66	DNA-3-methyladenine glycosylase I
Smlt1097	34.16	28.24	-1.21	50.83	+1.49	DUF4153 domain-containing protein
Smlt1098	365.94	481.85	+1.32	435.62	+1.19	YqgE/AlgH family protein
Smlt1099	126.73	199.16	+1.57	227.57	+1.8	Holliday junction resolvase RuvX
Smlt1100	327.83	316.06	-1.04	408.88	+1.25	aspartate carbamoyltransferase catalytic subunit
Smlt1101	0.00	0.00	0	0.00	0	transposase
Smlt1102	42.14	55.57	+1.32	79.88	+1.9	prolyl oligopeptidase family serine peptidase
Smlt1103	78.11	45.87	-1.7	60.33	-1.29	magnesium transporter
Smlt1104	93.76	170.38	+1.82	112.68	+1.2	phosphoenolpyruvate--protein phosphotransferase
Smlt1105	126.78	215.38	+1.7	142.29	+1.12	HPr family phosphocarrier protein
Smlt1106	121.25	285.14	+2.35	187.63	+1.55	hypothetical protein
Smlt1108	150.03	146.08	-1.03	131.28	-1.14	RNase adapter RapZ
Smlt1109	57.80	53.01	-1.09	48.35	-1.2	HPr(Ser) kinase/phosphatase
Smlt1110	75.23	69.60	-1.08	69.46	-1.08	PTS sugar transporter subunit IIA
Smlt1111	1676.49	1000.41	-1.68	912.20	-1.84	ribosome-associated translation inhibitor RaiA
Smlt1112	160.18	178.15	+1.11	263.41	+1.64	RNA polymerase factor sigma-54
Smlt1113	204.20	234.79	+1.15	254.52	+1.25	LPS export ABC transporter ATP-binding protein
Smlt1114	186.71	224.11	+1.2	246.11	+1.32	lipopolysaccharide transport periplasmic protein LptA

Smlt1115	70.55	82.47	+1.17	65.84	-1.07	LPS export ABC transporter periplasmic protein LptC
Smlt1116	96.82	148.66	+1.54	97.95	+1.01	HAD hydrolase family protein
Smlt1117	68.40	65.18	-1.05	71.22	+1.04	KpsF/GutQ family sugar-phosphate isomerase
Smlt1118	663.55	574.79	-1.15	754.05	+1.14	BolA family transcriptional regulator
Smlt1119	244.44	216.03	-1.13	339.27	+1.39	UDP-N-acetylglucosamine 1-carboxyvinyltransferase
Smlt1120	144.86	152.26	+1.05	225.77	+1.56	hypothetical protein
Smlt1121	155.31	230.36	+1.48	215.52	+1.39	DUF3108 domain-containing protein
Smlt1122	108.14	174.08	+1.61	189.70	+1.75	DUF3108 domain-containing protein
Smlt1123	35.30	37.94	+1.07	78.89	+2.23	phosphoribosylglycinamide formyltransferase
Smlt1124	20.81	14.81	-1.41	31.99	+1.54	DUF2238 domain-containing protein
Smlt1125	44.56	34.20	-1.3	39.40	-1.13	hypothetical protein
Smlt1126	284.92	245.89	-1.16	271.92	-1.05	phosphoribosylformylglycinamidine cyclo-ligase
Smlt1127	90.48	113.33	+1.25	129.11	+1.43	DUF2066 domain-containing protein
Smlt1128	87.78	98.33	+1.12	86.59	-1.01	AI-2E family transporter
Smlt1129	182.21	241.82	+1.33	257.32	+1.41	DnaA regulatory inactivator Hda
Smlt1130	35.10	46.45	+1.32	59.82	+1.7	nucleotidyltransferase family protein
Smlt1131	68.12	53.68	-1.27	108.95	+1.6	phosphotransferase
Smlt1132	123.15	140.50	+1.14	266.66	+2.17	GlsB/YeaQ/YmgE family stress response membrane protein
Smlt1133	575.51	758.76	+1.32	705.94	+1.23	M20 family metallopeptidase
Smlt1134	62278.21	74334.68	+1.19	50256.71	-1.24	helix-hairpin-helix domain-containing protein
Smlt1135	70.26	43.97	-1.6	37.94	-1.85	L,D-transpeptidase
Smlt1136	116.73	152.18	+1.3	102.68	-1.14	murein L,D-transpeptidase catalytic domain family protein
Smlt1137	82.45	87.97	+1.07	111.18	+1.35	HutD family protein
Smlt1138	9.68	25.92	+2.68	175.68	+18.15	sulfite reductase subunit alpha
Smlt1139	17.92	57.00	+3.18	237.54	+13.26	FAD:protein FMN transferase
Smlt1140	20.44	52.05	+2.55	196.36	+9.61	DUF4198 domain-containing protein
Smlt1141	12.20	19.72	+1.62	101.38	+8.31	DUF2271 domain-containing protein
Smlt1142	30.21	30.20	-1.	246.84	+8.17	PepSY-associated TM helix domain-containing protein
Smlt1144c	2.71	15.77	+5.82	5.65	+2.08	TonB-dependent outer membrane receptor

Smlt1145	6.42	11.05	+1.72	73.85	+11.49	hypothetical protein
Smlt1146	28.25	40.61	+1.44	199.05	+7.05	Fe^{2+}-dependent dioxygenase
Smlt1147	11.52	30.27	+2.63	124.54	+10.81	sel1 repeat family protein
Smlt1148	7.58	42.84	+5.65	488.70	+64.45	TonB-dependent outer membrane receptor
Smlt1149	28.86	38.41	+1.33	440.89	+15.28	inner membrane transmembrane protein
Smlt1150	293.66	249.33	-1.18	246.32	-1.19	non-heme iron oxygenase ferredoxin subunit
Smlt1151	35.46	30.04	-1.18	41.64	+1.17	GNAT family N-acetyltransferase
Smlt1152	0.00	0.00	0	0.00	0	transposase
Smlt1153	96.87	163.74	+1.69	388.94	+4.02	cysteine desulfurase
Smlt1154	67.46	137.14	+2.03	301.34	+4.47	Fe-S cluster assembly protein SufD
Smlt1155	93.63	178.46	+1.91	400.47	+4.28	Fe-S cluster assembly ATPase SufC
Smlt1156	50.08	51.76	+1.03	182.42	+3.64	hypothetical protein
Smlt1157	105.02	180.59	+1.72	304.45	+2.9	Fe-S cluster assembly protein SufB
Smlt1158	70.93	78.27	+1.1	172.82	+2.44	SUF system Fe-S cluster assembly regulator
Smlt1159	208.63	256.33	+1.23	312.45	+1.5	SET domain-containing protein-lysine N-methyltransferase
Smlt1160	51.93	43.82	-1.19	87.19	+1.68	type 1 glutamine amidotransferase
Smlt1161	91.94	137.26	+1.49	68.48	-1.34	DUF1439 domain-containing protein
Smlt1162	45.29	27.73	-1.63	33.88	-1.34	hypothetical protein
Smlt1163	16.84	11.85	-1.42	19.13	+1.14	DUF3861 family protein
Smlt1164	3675.51	2072.32	-1.77	5064.71	+1.38	HU family DNA-binding protein
Smlt1165	443.47	378.80	-1.17	850.21	+1.92	OsmC family protein
Smlt1166	70.76	73.92	+1.04	109.37	+1.55	PhzF family phenazine biosynthesis protein
Smlt1167	77.47	89.01	+1.15	129.27	+1.67	PhzF family phenazine biosynthesis protein
Smlt1168	152.80	191.36	+1.25	214.45	+1.4	bifunctional aspartate kinase/diaminopimelate decarboxylase
Smlt1169	135.58	247.30	+1.82	251.68	+1.86	hypothetical protein
Smlt1170	84.70	187.15	+2.21	188.65	+2.23	UDP-N-acetylmuramoyl-L-alanine--D-glutamate ligase
Smlt1171	44.43	46.22	+1.04	76.53	+1.72	SMI1/KNR4 family protein
Smlt1172	38.08	55.36	+1.45	46.42	+1.22	dienelactone hydrolase family protein
Smlt1173	497.58	519.31	+1.04	520.62	+1.05	polyprenyl synthetase family protein

Smlt1174	19.76	29.95	+1.52	41.14	+2.08	alpha-glucosidase family protein
Smlt1175	3.61	6.67	+1.85	11.42	+3.16	TonB-dependent outer membrane receptor
Smlt1176	1.36	3.61	+2.65	2.68	+1.97	glycoside hydrolase family 97 protein
Smlt1177	13.48	20.30	+1.51	18.75	+1.39	Six-hairpin glycosidase-like protein
Smlt1178	8.31	13.64	+1.64	7.73	-1.07	MFS transporter
Smlt1179	18.72	34.03	+1.82	25.36	+1.35	cyclomaltodextrin glucanotransferase
Smlt1180	51.04	39.98	-1.28	47.24	-1.08	SseB family protein
Smlt1181	38.43	44.91	+1.17	50.78	+1.32	GNAT family N-acetyltransferase
Smlt1183	0.00	0.00	0	0.00	0	hypothetical protein
Smlt1184	3.55	5.24	+1.48	10.96	+3.08	ABC transporter permease subunit
Smlt1185	5.59	6.54	+1.17	15.85	+2.83	ABC transporter ATP-binding protein
Smlt1186	22.87	27.81	+1.22	38.63	+1.69	LacI family DNA-binding transcriptional regulator
Smlt1187	1126.66	808.63	-1.39	1099.39	-1.02	single-stranded DNA-binding protein
Smlt1188	20.55	225.06	+10.95	24.53	+1.19	TIM barrel protein
Smlt1189	47.45	360.85	+7.61	35.74	-1.33	GMC family oxidoreductase
Smlt1190	41.49	370.76	+8.94	29.17	-1.42	gluconate 2-dehydrogenase subunit 3 family protein
Smlt1191	23.84	210.89	+8.85	11.91	-2.	DUF1080 domain-containing protein
Smlt1192	23.86	344.54	+14.44	41.36	+1.73	cytochrome c
Smlt1193	27.92	348.76	+12.49	39.23	+1.41	sugar phosphate isomerase/epimerase
Smlt1194	19.57	258.38	+13.2	27.87	+1.42	Gfo/Idh/MocA family oxidoreductase
Smlt1195	17.02	188.99	+11.1	21.05	+1.24	nucleoside permease
Smlt1196	48.33	524.91	+10.86	57.92	+1.2	sugar phosphate isomerase/epimerase
Smlt1197	53.41	126.94	+2.38	64.96	+1.22	LacI family transcriptional regulator
Smlt1198	11.31	20.54	+1.82	8.56	-1.32	sensor domain-containing diguanylate cyclase
Smlt1199	44.53	39.57	-1.13	38.52	-1.16	AI-2E family transporter
Smlt1200	41.05	42.38	+1.03	43.95	+1.07	GGDEF domain-containing protein
Smlt1201	3.12	1.97	-1.59	2.80	-1.12	PaaI family thioesterase
Smlt1202	0.87	1.53	+1.77	0.67	-1.3	hypothetical protein
Smlt1203	12.92	29.08	+2.25	68.74	+5.32	pyridoxamine 5'-phosphate oxidase family protein

Smlt1204	10.00	7.16	-1.4	4.01	-2.49	DUF2867 domain-containing protein
Smlt1205	17.64	18.13	+1.03	6.50	-2.71	metalloregulator ArsR/SmtB family transcription factor
Smlt1206	50.00	49.03	-1.02	93.64	+1.87	hypothetical protein
Smlt1207	22.55	29.69	+1.32	18.02	-1.25	DUF2975 domain-containing protein
Smlt1208	16.75	24.40	+1.46	17.38	+1.04	helix-turn-helix transcriptional regulator
Smlt1209	30.28	41.08	+1.36	30.78	+1.02	TerC family protein
Smlt1210	17.57	16.98	-1.04	31.83	+1.81	metallophosphoesterase
Smlt1211	1.65	3.25	+1.97	1.90	+1.15	ferric reductase-like transmembrane domain-containing protein
Smlt1212	0.00	0.00	0	0.00	0	putative DNA-binding domain-containing protein
Smlt1213	0.53	0.30	-1.81	0.27	-1.95	DUF692 domain-containing protein
Smlt1214	0.48	0.26	-1.81	0.23	-2.08	EF-hand domain-containing protein
Smlt1215	0.36	1.07	+2.94	0.28	-1.3	DoxX family protein
Smlt1216	3.70	2.93	-1.26	2.48	-1.49	alpha/beta hydrolase
Smlt1217	0.00	0.00	0	0.00	0	redoxin domain-containing protein
Smlt1218	30.93	26.27	-1.18	48.48	+1.57	response regulator
Smlt1219	20.31	18.23	-1.11	35.68	+1.76	HAMP domain-containing histidine kinase
Smlt1220	33.83	21.00	-1.61	54.97	+1.62	hypothetical protein
Smlt1221	43.68	76.00	+1.74	36.81	-1.19	MFS transporter
Smlt1222	157.87	223.16	+1.41	147.07	-1.07	nucleoside hydrolase
Smlt1223	0.00	0.00	0	0.00	0	hypothetical protein
Smlt1224	131.52	151.60	+1.15	210.54	+1.6	GntR family transcriptional regulator
Smlt1225	15.43	37.16	+2.41	24.86	+1.61	hypothetical protein
Smlt1226	19.40	32.79	+1.69	52.88	+2.73	malonate decarboxylase subunit epsilon
Smlt1227	20.63	27.44	+1.33	50.56	+2.45	triphosphoribosyl-dephospho-CoA synthase MdcB
Smlt1228	0.00	0.00	0	0.00	0	malonate decarboxylase holo-[acyl-carrier-protein] synthase
Smlt1229	0.00	0.00	0	0.00	0	biotin-independent malonate decarboxylase subunit gamma
Smlt1230	39.32	44.75	+1.14	73.82	+1.88	biotin-independent malonate decarboxylase subunit beta
Smlt1231	37.82	29.80	-1.27	50.09	+1.32	malonate decarboxylase acyl carrier protein
Smlt1232	48.98	50.96	+1.04	66.09	+1.35	malonate decarboxylase subunit alpha

Smlt1233	2.58	31.79	+12.33	296.30	+114.91	TonB-dependent outer membrane receptor
Smlt1234	29.28	25.42	-1.15	30.95	+1.06	malate dehydrogenase (quinone)
Smlt1235	47.57	44.85	-1.06	22.62	-2.1	GNAT family N-acetyltransferase
Smlt1236	0.00	0.00	0	0.00	0	hypothetical protein
Smlt1237	9.82	6.06	-1.62	6.88	-1.43	LysR family transcriptional regulator ArgP
Smlt1238	166.20	4.19	-39.71	99.13	-1.68	LysE family transporter
Smlt1240	0.00	0.00	0	0.00	0	prolyl oligopeptidase, pseudogene
Smlt1241	69.34	35.88	-1.93	50.10	-1.38	oligopeptide:H+ symporter
Smlt1242	2.03	1.16	-1.76	1.54	-1.32	heparan-alpha-glucosaminide N-acetyltransferase domain-containing protein
Smlt1243	5.28	5.21	-1.01	6.24	+1.18	hypothetical protein
Smlt1245	70.68	66.98	-1.06	140.46	+1.99	oligopeptide transporter, OPT family
Smlt1246	187.06	137.85	-1.36	285.49	+1.53	S9 family peptidase
Smlt1247	24.03	25.03	+1.04	52.25	+2.17	DUF819 family protein
Smlt1249	159.12	318.91	+2.	123.98	-1.28	hypothetical protein
Smlt1250	478.09	767.54	+1.61	403.51	-1.18	cell division topological specificity factor MinE
Smlt1251	1176.18	1589.30	+1.35	1221.56	+1.04	septum site-determining protein MinD
Smlt1252	367.53	463.66	+1.26	488.91	+1.33	septum site-determining protein MinC
Smlt1253	141.83	259.06	+1.83	242.27	+1.71	GNAT family N-acetyltransferase
Smlt1254	12.72	24.60	+1.93	30.40	+2.39	sensor histidine kinase
Smlt1255	82.79	116.50	+1.41	122.57	+1.48	response regulator transcription factor
Smlt1256	931.29	594.20	-1.57	1359.63	+1.46	SRPBCC family protein
Smlt1257	43.17	69.77	+1.62	161.92	+3.75	DUF423 domain-containing protein
Smlt1259	84.80	90.28	+1.06	121.26	+1.43	D-alanyl-D-alanine carboxypeptidase family protein
Smlt1260	30.34	47.06	+1.55	18.05	-1.68	membrane protein
Smlt1261	3.35	5.55	+1.66	3.70	+1.1	DUF4177 domain-containing protein
Smlt1262	43.14	64.71	+1.5	33.48	-1.29	hypothetical protein
Smlt1263	48.64	84.89	+1.75	38.99	-1.25	SPFH domain-containing protein
Smlt1264	45.38	35.78	-1.27	60.17	+1.33	formate-dependent phosphoribosylglycinamide formyltransferase

Smlt1265	181.14	154.06	-1.18	276.43	+1.53	endonuclease/exonuclease/phosphatase family protein
Smlt1266	52.16	75.27	+1.44	39.59	-1.32	arginyltransferase
Smlt1268	5.51	10.97	+1.99	9.61	+1.74	EF-hand domain-containing protein
Smlt1269	12.50	27.73	+2.22	14.99	+1.2	sigma-70 family RNA polymerase sigma factor
Smlt1270	126.39	134.11	+1.06	200.21	+1.58	hypothetical protein
Smlt1271	137.11	128.77	-1.06	185.09	+1.35	acyl-CoA thioesterase II
Smlt1273	80.40	108.49	+1.35	95.23	+1.18	enoyl-CoA hydratase/isomerase family protein
Smlt1274	158.72	177.18	+1.12	264.13	+1.66	copper chaperone PCu(A)C
Smlt1275	109.04	140.95	+1.29	199.58	+1.83	hypothetical protein
Smlt1276	185.90	414.54	+2.23	392.27	+2.11	acyl-CoA thioesterase
Smlt1277	138.28	232.96	+1.68	200.86	+1.45	excinuclease ABC subunit UvrA
Smlt1278	5131.32	4512.08	-1.14	4406.92	-1.16	50S ribosomal protein L21
Smlt1279	4478.06	4340.85	-1.03	3282.75	-1.36	50S ribosomal protein L27
Smlt1280	284.76	237.25	-1.2	152.96	-1.86	GTPase ObgE
Smlt1282	0.00	0.00	0	0.00	0	DUF2274 domain-containing protein
Smlt1283	0.00	0.00	0	0.00	0	TrbI/VirB10 family protein
Smlt1284	0.00	0.00	0	0.00	0	P-type conjugative transfer protein TrbG
Smlt1285	0.00	0.00	0	0.00	0	conjugal transfer protein TrbF
Smlt1286	0.00	0.00	0	0.00	0	P-type conjugative transfer protein TrbL
Smlt1287	0.00	0.00	0	0.00	0	P-type conjugative transfer protein TrbJ
Smlt1288	0.00	0.00	0	0.00	0	conjugal transfer protein TrbE
Smlt1289	0.00	0.00	0	0.00	0	VirB3 family type IV secretion system protein
Smlt1290	0.00	0.00	0	0.00	0	TrbC/VirB2 family protein
Smlt1291	0.00	0.00	0	0.00	0	P-type conjugative transfer ATPase TrbB
Smlt1292	0.00	0.00	0	0.00	0	CopG family transcriptional regulator
Smlt1293	0.00	0.00	0	0.00	0	conjugal transfer protein TraG
Smlt1294	0.00	0.00	0	0.00	0	EexN family lipoprotein
Smlt1295	0.00	0.00	0	0.00	0	LysR family transcriptional regulator
Smlt1296	0.00	0.00	0	0.00	0	regulator, pseudogene

Smlt1297	0.00	0.00	0	0.00	0	esterase, pseudogene
Smlt1298	0.00	0.00	0	0.00	0	hypothetical protein
Smlt1299	0.00	0.00	0	0.00	0	NAD(P)H-dependent oxidoreductase
Smlt1300	0.00	0.00	0	0.00	0	LysR family transcriptional regulator
Smlt1301	0.00	0.00	0	0.00	0	EexN family lipoprotein
Smlt1302	0.00	0.00	0	0.00	0	LysR family transcriptional regulator
Smlt1303	0.00	0.00	0	0.00	0	hypothetical protein
Smlt1304	0.00	0.00	0	0.00	0	carboxymuconolactone decarboxylase family protein
Smlt1305	0.00	0.00	0	0.00	0	MerR family transcriptional regulator
Smlt1306	0.00	0.00	0	0.00	0	MFS transporter
Smlt1307	0.00	0.00	0	0.00	0	LysR family transcriptional regulator
Smlt1308	0.00	0.00	0	0.00	0	relaxase/mobilization nuclease and DUF3363 domain-containing protein
Smlt1309	0.00	0.00	0	0.00	0	conserved hypothetical protein
Smlt1310	0.00	0.00	0	0.00	0	S26 family signal peptidase
Smlt1311	0.00	0.00	0	0.00	0	DUF2840 domain-containing protein
Smlt1312	0.00	0.00	0	0.00	0	chromosome partitioning protein ParB
Smlt1313	1.30	2.86	+2.21	0.95	-1.36	AAA family ATPase
Smlt1314	0.00	0.00	0	0.00	0	replication initiator protein A
Smlt1315	0.00	0.00	0	0.00	0	helix-turn-helix domain-containing protein
Smlt1316	0.00	0.00	0	0.00	0	DUF2285 domain-containing protein
Smlt1318	0.00	0.00	0	0.00	0	DUF2958 domain-containing protein
Smlt1319	0.00	0.00	0	0.00	0	helix-turn-helix domain-containing protein
Smlt1320	0.00	0.00	0	0.00	0	DUF736 domain-containing protein
Smlt1320A	0.00	0.00	0	0.00	0	conserved hypothetical protein
Smlt1322	0.00	0.00	0	0.00	0	hypothetical protein
Smlt1323	0.00	0.00	0	0.00	0	IS630-like element ISStma10 family transposase
Smlt1324	0.00	0.00	0	0.00	0	hypothetical protein
Smlt1325	0.00	0.00	0	0.00	0	hypothetical protein

Smlt1326	0.00	0.00	0	0.00	0	ParB/RepB/Spo0J family partition protein
Smlt1327	0.00	0.00	0	0.00	0	DUF945 domain-containing protein
Smlt1328	0.00	0.00	0	0.00	0	conserved hypothetical protein
Smlt1329	0.00	0.00	0	0.00	0	nucleoid-associated protein
Smlt1331	0.00	0.00	0	0.00	0	hypothetical protein
Smlt1332	0.00	0.00	0	0.00	0	RadC DNA repair protein, pseudogene
Smlt1333	0.00	0.00	0	0.00	0	hypothetical protein
Smlt1334	0.00	0.00	0	0.00	0	DNA-binding transcriptional regulator
Smlt1335	0.00	0.00	0	0.00	0	type II toxin-antitoxin system RelE/ParE family toxin
Smlt1336	0.00	0.00	0	0.00	0	site-specific integrase
Smlt1337	3119.44	2125.12	-1.47	1708.02	-1.83	30S ribosomal protein S20
Smlt1338	38.61	44.58	+1.15	36.48	-1.06	murein biosynthesis integral membrane protein MurJ
Smlt1339	207.26	246.68	+1.19	153.36	-1.35	bifunctional riboflavin kinase/FAD synthetase
Smlt1340	215.43	330.36	+1.53	281.38	+1.31	isoleucine--tRNA ligase
Smlt1341	100.96	184.29	+1.83	136.15	+1.35	signal peptidase II
Smlt1342	487.18	576.14	+1.18	735.14	+1.51	4-hydroxy-3-methylbut-2-enyl diphosphate reductase
Smlt1343	26.41	33.07	+1.25	69.23	+2.62	M61 family metallopeptidase
Smlt1344	8.94	14.49	+1.62	27.58	+3.08	lysoplasmalogenase
Smlt1345	43.04	20.10	-2.14	60.56	+1.41	hypothetical protein
Smlt1346	25.84	12.08	-2.14	24.80	-1.04	hypothetical protein
Smlt1347	19.05	24.60	+1.29	36.40	+1.91	FecA-like TonB-dependent outer membrane receptor
Smlt1348	78.59	90.40	+1.15	129.98	+1.65	FecR-like protein
Smlt1349	59.13	83.03	+1.4	94.42	+1.6	FecI-like RNA polymerase sigma factor
Smlt1350	37.00	15.44	-2.4	120.30	+3.25	autotransporter outer membrane beta-barrel domain-containing protein
Smlt1351	56.57	75.66	+1.34	96.63	+1.71	2-hydroxyacid dehydrogenase
Smlt1353	0.00	0.00	0	0.00	0	hypothetical protein
Smlt1354	0.00	0.00	0	0.00	0	lipoprotein

Smlt1355	0.00	0.00	0	0.00	0	conserved hypothetical protein found associated with cell wall in gram positives
Smlt1356	54.43	44.59	-1.22	32.27	-1.69	hypothetical protein
Smlt1357	82.54	158.75	+1.92	102.96	+1.25	hypothetical protein
Smlt1358	35.18	68.97	+1.96	39.55	+1.12	hypothetical protein
Smlt1359	30.31	63.89	+2.11	51.22	+1.69	GyrI-like domain-containing protein
Smlt1360	881.07	803.32	-1.1	732.12	-1.2	ubiquinol oxidase subunit II
Smlt1361	524.11	676.16	+1.29	670.75	+1.28	cytochrome o ubiquinol oxidase subunit I
Smlt1362	688.36	957.44	+1.39	1256.59	+1.83	cytochrome o ubiquinol oxidase subunit III
Smlt1363	160.71	209.93	+1.31	195.87	+1.22	cytochrome o ubiquinol oxidase subunit IV
Smlt1365	24.84	13.06	-1.9	32.59	+1.31	bifunctional diguanylate cyclase/phosphodiesterase
Smlt1366	147.89	256.77	+1.74	89.19	-1.66	DNA repair protein RadA
Smlt1367	71.62	74.95	+1.05	87.00	+1.21	ATP-binding protein
Smlt1368	109.68	106.99	-1.03	54.25	-2.02	tetratricopeptide repeat protein
Smlt1369	71.99	66.73	-1.08	43.62	-1.65	hypothetical protein
Smlt1370	41.16	63.94	+1.55	85.13	+2.07	cytochrome c biogenesis protein CcsA
Smlt1371	400.03	424.48	+1.06	415.05	+1.04	signal recognition particle protein
Smlt1372	52.57	64.50	+1.23	80.87	+1.54	nitronate monooxygenase
Smlt1373	75.77	83.50	+1.1	101.28	+1.34	aminotransferase class IV family protein
Smlt1374	4450.09	3324.86	-1.34	2539.53	-1.75	30S ribosomal protein S16
Smlt1375	453.55	252.34	-1.8	338.19	-1.34	ribosome maturation factor RimM
Smlt1376	229.96	174.61	-1.32	235.49	+1.02	tRNA (guanosine(37)-N1)-methyltransferase TrmD
Smlt1377	1301.80	954.44	-1.36	828.98	-1.57	50S ribosomal protein L19
Smlt1378	128.83	299.80	+2.33	113.23	-1.14	DUF6404 family protein
Smlt1379	144.21	315.37	+2.19	248.17	+1.72	DUF1801 domain-containing protein
Smlt1380	114.94	197.79	+1.72	152.39	+1.33	DUF1801 domain-containing protein
Smlt1381	13.21	8.60	-1.54	19.73	+1.49	MATE family efflux transporter
Smlt1383	61.35	60.36	-1.02	46.80	-1.31	RNA-binding S4 domain-containing protein
Smlt1384	24.51	32.10	+1.31	70.08	+2.86	DUF937 domain-containing protein

Smlt1385	50.41	54.31	+1.08	22.99	-2.19	catalase
Smlt1386	137.80	111.02	-1.24	187.63	+1.36	hypothetical protein
Smlt1387	37.23	74.86	+2.01	35.48	-1.05	DNA mismatch repair protein MutS
Smlt1388	0.00	0.00	0	0.00	0	barstar family protein
Smlt1389	0.00	0.00	0	0.00	0	ShlB/FhaC/HecB family hemolysin secretion/activation protein
Smlt1390	0.00	0.00	0	0.00	0	outer membrane surface heamagglutinin protein
Smlt1391	0.00	0.00	0	0.00	0	heamagglutinin/hemolysin related protein, pseudogene
Smlt1395	0.00	0.00	0	0.00	0	heamagglutinin/heamolysin, pseudogene
Smlt1396A	0.00	0.00	0	0.00	0	DUF596 domain-containing protein
Smlt1399	0.00	0.00	0	0.00	0	hypothetical protein
Smlt1400	0.00	0.00	0	0.00	0	DUF2628 domain-containing protein
Smlt1401	0.00	0.00	0	0.00	0	hypothetical protein
Smlt1402	11.67	8.23	-1.42	9.36	-1.25	methyl-accepting chemotaxis protein
Smlt1403	614.00	1207.54	+1.97	443.86	-1.38	glycine zipper 2TM domain-containing protein
Smlt1404	35.43	39.53	+1.12	66.71	+1.88	ABC transporter permease
Smlt1405	26.46	37.11	+1.4	40.48	+1.53	ABC transporter permease
Smlt1406	55.10	76.55	+1.39	74.55	+1.35	efflux RND transporter periplasmic adaptor subunit
Smlt1407	54.78	64.07	+1.17	61.77	+1.13	TolC family protein
Smlt1408	16.73	13.73	-1.22	26.13	+1.56	LysR family transcriptional regulator ArgP
Smlt1409	0.00	0.00	0	0.00	0	LysE/ArgO family amino acid transporter
Smlt1410	77.13	117.94	+1.53	247.07	+3.2	type I methionyl aminopeptidase
Smlt1411	34.34	60.93	+1.77	133.03	+3.87	ParD-like family protein
Smlt1412	7.33	14.33	+1.95	15.85	+2.16	VOC family protein
Smlt1413	22.49	102.28	+4.55	69.83	+3.1	hypothetical protein
Smlt1414	40.35	138.03	+3.42	102.43	+2.54	GNAT family N-acetyltransferase
Smlt1415	67.81	290.67	+4.29	164.72	+2.43	aminotransferase class III-fold pyridoxal phosphate-dependent enzyme
Smlt1416	41.01	97.12	+2.37	42.31	+1.03	MtnX-like HAD-IB family phosphatase
Smlt1417	7.40	23.86	+3.23	19.97	+2.7	DegT/DnrJ/EryC1/StrS family aminotransferase

Smlt1418	13.42	32.32	+2.41	18.23	+1.36	DMT family transporter
Smlt1419	8.59	27.73	+3.23	9.90	+1.15	EamA family transporter
Smlt1420	48.37	121.96	+2.52	55.18	+1.14	alpha/beta fold hydrolase
Smlt1421	15.08	20.68	+1.37	31.20	+2.07	sensor histidine kinase
Smlt1422	32.52	32.72	+1.01	70.36	+2.16	response regulator
Smlt1423	27.07	25.67	-1.05	81.91	+3.03	metallophosphoesterase
Smlt1424	0.00	0.00	0	0.00	0	Major Facilitator Superfamily transmembrane protein
Smlt1425	28.91	12.51	-2.31	31.69	+1.1	GGDEF domain-containing protein
Smlt1426	23.29	146.79	+6.3	2791.57	+119.87	FepA, TonB-dependent outer membrane receptor
Smlt1427	103.46	145.66	+1.41	64.70	-1.6	hypothetical protein
Smlt1428	119.90	3719.86	+31.03	354.35	+2.96	cation diffusion facilitator family transporter
Smlt1429	29.49	54.72	+1.86	25.58	-1.15	deoxyribonuclease V
Smlt1430	824.23	802.43	-1.03	1294.91	+1.57	2,3-diphosphoglycerate-dependent phosphoglycerate mutase
Smlt1431	100.56	167.98	+1.67	243.62	+2.42	M13 family metallopeptidase
Smlt1432	29.65	18.31	-1.62	31.41	+1.06	HD domain-containing protein
Smlt1433	125.81	122.50	-1.03	150.25	+1.19	transcription-repair coupling factor
Smlt1434	281.28	606.27	+2.16	252.32	-1.11	GNAT family N-acetyltransferase
Smlt1435	72.23	66.03	-1.09	85.10	+1.18	23S rRNA (adenine(2030)-N(6))-methyltransferase RlmJ
Smlt1436	19.63	20.17	+1.03	35.32	+1.8	two-component system response regulator CreB
Smlt1437	28.18	28.76	+1.02	38.39	+1.36	two-component system sensor histidine kinase CreC
Smlt1438	7.03	10.34	+1.47	8.06	+1.15	cell envelope integrity protein CreD
Smlt1439	20.55	27.33	+1.33	44.20	+2.15	hypothetical protein
Smlt1440	64.88	92.21	+1.42	156.34	+2.41	M20/M25/M40 family metallo-hydrolase
Smlt1441	650.37	738.31	+1.14	549.40	-1.18	glutamate--tRNA ligase
Smlt1442	271.55	451.31	+1.66	237.20	-1.14	transcriptional repressor
Smlt1443	51.18	52.44	+1.02	47.13	-1.09	HlyD family secretion protein
Smlt1444	21.89	23.06	+1.05	14.19	-1.54	MFS transporter
Smlt1445	110.63	144.52	+1.31	66.12	-1.67	TetR/AcrR family transcriptional regulator%3B helix-turn-helix transcriptional regulator

Smlt1446	8.67	8.63	-1.01	3.62	-2.39	TonB-dependent outer membrane receptor
Smlt1447	60.64	31.15	-1.95	12.25	-4.95	MerC domain-containing protein
Smlt1448	1272.10	1064.87	-1.19	1275.95	+1.	30S ribosomal protein THX
Smlt1449	225.95	135.40	-1.67	169.21	-1.34	ectonucleotide pyrophosphatase/phosphodiesterase
Smlt1450	0.00	0.00	0	0.00	0	conserved hypothetical protein
Smlt1451	16.86	20.97	+1.24	10.36	-1.63	methylated-DNA--[protein]-cysteine S-methyltransferase
Smlt1452	9.84	10.80	+1.1	8.82	-1.12	DNA-3-methyladenine glycosylase 2
Smlt1453	0.00	0.00	0	0.00	0	hypothetical protein
Smlt1454	102.99	152.95	+1.49	105.24	+1.02	hypothetical protein
Smlt1455	64.76	96.30	+1.49	94.99	+1.47	DUF4019 domain-containing protein
Smlt1456	0.00	0.00	0	0.00	0	SMI1/KNR4 family protein
Smlt1458	38.28	73.38	+1.92	44.35	+1.16	MAPEG family protein
Smlt1459	666.48	365.73	-1.82	221.07	-3.01	5-demethoxyubiquinol-8 5-hydroxylase UbiM
Smlt1460	34.64	27.73	-1.25	32.63	-1.06	RNA-binding S4 domain-containing protein
Smlt1461	360.91	296.15	-1.22	193.05	-1.87	DEAD/DEAH box helicase
Smlt1462	37.82	37.68	-1.	23.85	-1.59	EAL domain-containing protein
Smlt1463	72.50	47.57	-1.52	69.87	-1.04	GGDEF domain-containing protein
Smlt1464	206.90	240.60	+1.16	176.68	-1.17	pseudouridine synthase
Smlt1465	235.13	199.63	-1.18	283.10	+1.2	sensor domain-containing diguanylate cyclase
Smlt1466	60.91	76.60	+1.26	51.16	-1.19	AAA family ATPase
Smlt1467	52.65	56.69	+1.08	40.01	-1.32	DMT family transporter
Smlt1468	11.51	10.06	-1.14	17.97	+1.56	DUF72 domain-containing protein
Smlt1469	34.47	32.04	-1.08	59.97	+1.74	VOC family protein
Smlt1470	45.53	52.16	+1.15	78.30	+1.72	serine hydrolase
Smlt1471	74.33	74.43	+1.	87.30	+1.17	multidrug efflux ABC transporter SmrA
Smlt1472	54.97	51.28	-1.07	66.48	+1.21	bifunctional glucose-1-phosphatase/inositol phosphatase
Smlt1473	4.66	2.70	-1.73	8.37	+1.8	polysaccharide lyase
Smlt1474	100.28	147.62	+1.47	243.69	+2.43	DUF885 domain-containing protein
Smlt1475	0.00	0.00	0	0.00	0	GFA family protein

Smlt1476	83.06	61.17	-1.36	102.65	+1.24	DUF4349 domain-containing protein
Smlt1477	67.19	160.44	+2.39	101.80	+1.52	hypothetical protein
Smlt1478	2.95	1.67	-1.76	4.91	+1.66	LysR family transcriptional regulator
Smlt1479	0.00	0.00	0	0.00	0	transmembrane protein, pseudogene
Smlt1480	0.00	0.00	0	0.00	0	type II secretion system protein, pseudogene
Smlt1481	0.00	0.00	0	0.00	0	transmembrane protein, pseudogene
Smlt1482	0.00	0.00	0	0.00	0	peptidyl-prolyl cis-trans isomerase
Smlt1483	33.06	41.46	+1.25	87.02	+2.63	peptidylprolyl isomerase
Smlt1484	11.95	26.82	+2.24	17.26	+1.44	PadR family transcriptional regulator
Smlt1485	8.30	10.34	+1.25	6.76	-1.23	sensor domain-containing protein
Smlt1486	78.62	96.61	+1.23	124.71	+1.59	polyhydroxyalkanoate depolymerase
Smlt1487	41.84	20.69	-2.02	59.96	+1.43	class I SAM-dependent methyltransferase
Smlt1488	97.06	107.64	+1.11	47.34	-2.05	CopD family protein
Smlt1490	504.13	299.67	-1.68	434.36	-1.16	acetyl-CoA carboxylase carboxyltransferase subunit alpha
Smlt1491	113.18	82.52	-1.37	115.63	+1.02	hypothetical protein
Smlt1492	115.16	87.15	-1.32	115.17	+1.	DNA polymerase III subunit alpha
Smlt1493	59.91	67.84	+1.13	87.26	+1.46	ribonuclease HII
Smlt1494	99.78	89.83	-1.11	104.65	+1.05	lipid-A-disaccharide synthase
Smlt1495	270.07	226.15	-1.19	275.57	+1.02	acyl-ACP--UDP-N-acetylglucosamine O-acyltransferase
Smlt1496	187.26	148.26	-1.26	179.46	-1.04	3-hydroxyacyl-ACP dehydratase FabZ
Smlt1497	107.60	84.25	-1.28	166.65	+1.55	UDP-3-O-(3-hydroxymyristoyl)glucosamine N-acyltransferase
Smlt1498	302.67	277.87	-1.09	330.95	+1.09	outer membrane protein assembly factor BamA
Smlt1499	115.23	117.60	+1.02	140.22	+1.22	RIP metalloprotease RseP
Smlt1500	124.20	130.83	+1.05	170.99	+1.38	1-deoxy-D-xylulose-5-phosphate reductoisomerase
Smlt1501	68.82	68.98	+1.	73.52	+1.07	phosphatidate cytidylyltransferase
Smlt1502	158.15	151.44	-1.04	204.25	+1.29	di-trans,poly-cis-decaprenylcistransferase
Smlt1503	276.18	219.75	-1.26	345.28	+1.25	ribosome recycling factor
Smlt1504	370.67	252.09	-1.47	423.67	+1.14	UMP kinase
Smlt1505	298.06	494.67	+1.66	320.61	+1.08	GGDEF domain-containing protein

Smlt1506	1052.79	1128.33	+1.07	917.62	-1.15	translation elongation factor Ts
Smlt1507	710.05	716.68	+1.01	591.56	-1.2	30S ribosomal protein S2
Smlt1508	14.99	9.76	-1.54	10.57	-1.42	hypothetical protein
Smlt1509	28.50	3.04	-9.36	17.55	-1.62	hypothetical protein
Smlt1510	0.00	0.00	0	0.00	0	spore coat U domain-containing protein
Smlt1511	1.90	3.88	+2.04	1.49	-1.28	fimbria/pilus outer membrane usher protein
Smlt1512	2.31	4.10	+1.78	1.62	-1.43	molecular chaperone
Smlt1513	1.28	2.35	+1.84	1.58	+1.24	spore coat U domain-containing protein
Smlt1513A	490.17	666.07	+1.36	412.44	-1.19	type I methionyl aminopeptidase
Smlt1514	93.50	149.99	+1.6	95.65	+1.02	[protein-PII] uridylyltransferase
Smlt1515	165.70	335.59	+2.03	239.89	+1.45	2,3,4,5-tetrahydropyridine-2,6-dicarboxylate N-succinyltransferase
Smlt1517	145.12	187.63	+1.29	263.87	+1.82	arsenate reductase
Smlt1518	200.12	243.46	+1.22	301.93	+1.51	succinyl-diaminopimelate desuccinylase
Smlt1520	11.41	35.20	+3.09	13.78	+1.21	asparagine synthase B
Smlt1521	33.11	17.13	-1.93	16.33	-2.03	cation:proton antiporter
Smlt1522	47.71	62.97	+1.32	89.55	+1.88	penicillin acylase family protein
Smlt1524	422.16	469.05	+1.11	31.70	-13.32	bacterioferritin
Smlt1525	348.59	254.03	-1.37	299.47	-1.16	DNA topoisomerase IV subunit A
Smlt1526	77.70	148.09	+1.91	78.08	+1.	helix-turn-helix transcriptional regulator
Smlt1527	21.40	20.42	-1.05	101.99	+4.77	multidrug efflux system transcriptional regulator EmrR
Smlt1528	24.48	38.20	+1.56	140.70	+5.75	multidrug efflux transporter outer membrane subunit EmrC
Smlt1529	37.12	77.49	+2.09	235.39	+6.34	multidrug efflux MFS transporter periplasmic adaptor subunit EmrA
Smlt1530	62.52	110.24	+1.76	425.36	+6.8	multidrug efflux MFS transporter permease subunit EmrB
Smlt1530A	17.02	5.96	-2.85	8.31	-2.05	hypothetical protein
Smlt1532	21.45	14.79	-1.45	18.00	-1.19	LysR family transcriptional regulator
Smlt1533	0.00	0.00	0	0.00	0	transmembrane protein
Smlt1534	290.80	454.92	+1.56	353.55	+1.22	ribonuclease R

Smlt1535	77.58	112.65	+1.45	88.84	+1.15	GFA family protein
Smlt1536	100.83	71.12	-1.42	70.52	-1.43	23S rRNA (guanosine(2251)-2'-O)-methyltransferase RlmB
Smlt1537	9.53	24.73	+2.59	43.96	+4.61	TolC family protein
Smlt1538	8.74	18.98	+2.17	44.44	+5.09	MacB family efflux pump subunit
Smlt1539	19.86	38.89	+1.96	67.63	+3.4	efflux RND transporter periplasmic adaptor subunit
Smlt1540	180.06	213.18	+1.18	277.77	+1.54	response regulator
Smlt1541	50.48	62.54	+1.24	119.08	+2.36	HAMP domain-containing protein
Smlt1542	44.73	44.36	-1.01	56.19	+1.26	ligand-binding sensor domain-containing diguanylate cyclase
Smlt1543	154.66	190.70	+1.23	151.16	-1.02	ribonuclease T
Smlt1544	0.00	0.00	0	0.00	0	DoxX family protein
Smlt1545	0.00	0.00	0	0.00	0	putative DNA-binding domain-containing protein
Smlt1546	3.96	4.18	+1.06	8.37	+2.11	DUF692 domain-containing protein
Smlt1547	12.67	23.77	+1.88	25.81	+2.04	hypothetical protein
Smlt1548	11.71	6.93	-1.69	6.63	-1.77	phosphate signaling complex protein PhoU
Smlt1549	16.60	6.31	-2.63	10.88	-1.53	phosphate ABC transporter ATP-binding protein PstB
Smlt1550	3.67	2.02	-1.81	1.48	-2.48	phosphate ABC transporter permease PstA
Smlt1551	3.03	0.69	-4.42	1.50	-2.03	phosphate ABC transporter permease subunit PstC
Smlt1552	22.09	6.65	-3.32	10.54	-2.1	phosphate ABC transporter substrate-binding protein PstS
Smlt1554	1.41	2.37	+1.68	1.11	-1.27	phosphate ABC transporter substrate-binding protein PstS
Smlt1555	9.39	5.97	-1.57	6.05	-1.55	OprO/OprP family phosphate-selective porin
Smlt1556	55.62	56.43	+1.01	25.24	-2.2	endonuclease III
Smlt1557	21.72	24.21	+1.11	31.26	+1.44	hypothetical protein
Smlt1558	61.63	64.60	+1.05	71.45	+1.16	enoyl-CoA hydratase/isomerase family protein
Smlt1559	1589.54	1805.36	+1.14	2662.43	+1.67	FKBP-type peptidyl-prolyl cis-trans isomerase
Smlt1560	121.82	158.32	+1.3	209.55	+1.72	CoA pyrophosphatase
Smlt1561	110.21	151.09	+1.37	185.39	+1.68	sulfurtransferase
Smlt1562	114.49	91.85	-1.25	124.73	+1.09	N-acetylmuramoyl-L-alanine amidase
Smlt1563	78.25	77.05	-1.02	98.17	+1.25	alpha/beta hydrolase

Smlt1564	74.34	62.93	-1.18	68.84	-1.08	bifunctional 23S rRNA (guanine(2069)-N(7))-methyltransferase RlmK/23S rRNA (guanine(2445)-N(2))-methyltransferase RlmL
Smlt1565	0.00	0.00	0	0.00	0	DUF3325 domain-containing protein
Smlt1566	6.63	31.39	+4.74	256.27	+38.66	PepSY domain-containing protein
Smlt1567	4.79	24.03	+5.02	263.79	+55.11	DUF3649 domain-containing protein
Smlt1568	44.23	43.18	-1.02	39.60	-1.12	DUF445 family protein
Smlt1569	255.09	108.56	-2.35	163.03	-1.56	hypothetical protein
Smlt1570	20.12	24.47	+1.22	21.07	+1.05	aspartate aminotransferase family protein
Smlt1571	24.07	43.24	+1.8	26.21	+1.09	aldehyde dehydrogenase
Smlt1572	0.00	0.00	0	0.00	0	hypothetical protein
Smlt1573	14.58	19.56	+1.34	24.60	+1.69	MHS family MFS transporter
Smlt1574	627.65	112.36	-5.59	181.49	-3.46	bacteriohemerythrin
Smlt1575	60.70	20.55	-2.95	41.99	-1.45	diguanylate cyclase
Smlt1576	15.62	16.86	+1.08	16.09	+1.03	MASE1 domain-containing protein
Smlt1577	25.88	22.70	-1.14	28.23	+1.09	MASE1 domain-containing protein
Smlt1578	53.37	74.13	+1.39	53.95	+1.01	FAD-binding oxidoreductase
Smlt1579	85.88	106.12	+1.24	129.09	+1.5	gamma-glutamyl-gamma-aminobutyrate hydrolase family protein
Smlt1580	132.56	117.89	-1.12	145.47	+1.1	glutamine synthetase family protein
Smlt1581	340.98	259.32	-1.31	349.50	+1.03	polyamine ABC transporter substrate-binding protein
Smlt1582	0.65	2.49	+3.86	1.10	+1.7	NAD(P)-binding domain-containing protein
Smlt1584	122.87	92.78	-1.32	114.26	-1.08	polyamine ABC transporter ATP-binding protein
Smlt1585	34.34	29.43	-1.17	42.54	+1.24	ABC transporter permease subunit
Smlt1586	30.59	44.11	+1.44	41.04	+1.34	ABC transporter permease subunit
Smlt1587	101.47	110.01	+1.08	99.25	-1.02	NAD-dependent succinate-semialdehyde dehydrogenase
Smlt1589	43.93	45.13	+1.03	58.03	+1.32	magnesium and cobalt transport protein CorA
Smlt1590	125.18	173.30	+1.38	167.45	+1.34	DUF4105 domain-containing protein
Smlt1591	416.30	438.70	+1.05	562.15	+1.35	CBS domain-containing protein
Smlt1592	11.16	8.49	-1.31	17.34	+1.55	hypothetical protein
Smlt1593	11.27	8.47	-1.33	9.80	-1.15	hypothetical protein

Smlt1594	139.15	92.76	-1.5	192.77	+1.39	hypothetical protein
Smlt1595	359.76	256.08	-1.4	606.59	+1.69	rRNA maturation RNase YbeY
Smlt1596	259.11	186.11	-1.39	370.72	+1.43	PhoH family protein
Smlt1597	130.94	77.11	-1.7	119.53	-1.1	HlyD family efflux transporter periplasmic adaptor subunit
Smlt1598	46.59	24.13	-1.93	24.55	-1.9	ABC transporter ATP-binding protein
Smlt1599	44.78	30.50	-1.47	21.09	-2.12	ABC transporter permease
Smlt1600	114.34	83.84	-1.36	36.47	-3.14	tRNA (N6-isopentenyl adenosine(37)-C2)-methylthiotransferase MiaB
Smlt1601	14.81	29.34	+1.98	14.23	-1.04	glutathione S-transferase
Smlt1602	27.31	46.70	+1.71	20.06	-1.36	WYL domain-containing protein
Smlt1603	71.71	62.87	-1.14	65.69	-1.09	lytic transglycosylase domain-containing protein
Smlt1604	238.94	568.69	+2.38	235.52	-1.01	ubiquinol-cytochrome c reductase iron-sulfur subunit
Smlt1605	66.48	133.17	+2.	21.11	-3.15	cytochrome bc complex cytochrome b subunit
Smlt1606	153.31	292.73	+1.91	34.88	-4.39	cytochrome c1
Smlt1607	402.94	811.26	+2.01	428.40	+1.06	glutathione S-transferase N-terminal domain-containing protein
Smlt1608	276.78	282.45	+1.02	277.13	+1.	ClpXP protease specificity-enhancing factor
Smlt1609	16.75	35.79	+2.14	22.12	+1.32	DUF3301 domain-containing protein
Smlt1610	25.36	37.78	+1.49	22.12	-1.15	DUF2272 domain-containing protein
Smlt1611	171.23	120.49	-1.42	197.06	+1.15	carboxylating nicotinate-nucleotide diphosphorylase
Smlt1612	85.73	59.47	-1.44	114.71	+1.34	hypothetical protein
Smlt1613	296.20	331.29	+1.12	277.72	-1.07	5-(carboxyamino)imidazole ribonucleotide mutase
Smlt1614	223.69	245.74	+1.1	189.88	-1.18	5-(carboxyamino)imidazole ribonucleotide synthase
Smlt1615	97.66	61.79	-1.58	91.49	-1.07	carboxymuconolactone decarboxylase family protein
Smlt1616	101.18	166.24	+1.64	158.58	+1.57	superoxide dismutase [Fe]
Smlt1617	315.44	442.92	+1.4	649.77	+2.06	Grx4 family monothiol glutaredoxin
Smlt1618	102.57	137.80	+1.34	129.47	+1.26	SDR family NAD(P)-dependent oxidoreductase
Smlt1619	1170.90	1788.36	+1.53	338.01	-3.46	TonB-dependent outer membrane receptor
Smlt1620	233.19	300.01	+1.29	406.06	+1.74	nucleotide 5'-monophosphate nucleosidase PpnN
Smlt1621	551.73	224.27	-2.46	85.91	-6.42	Tfp pilus assembly protein FimT/FimU

Smlt1622	276.60	142.31	-1.94	65.64	-4.21	type IV pilus modification protein PilV
Smlt1623	554.45	301.77	-1.84	106.31	-5.22	PilW family protein
Smlt1624	282.23	166.86	-1.69	58.17	-4.85	pilus assembly protein
Smlt1625	0.00	0.00	0	0.00	0	pilus assembly protein
Smlt1626	681.22	406.98	-1.67	136.43	-4.99	type IV pilin protein
Smlt1627	2.25	4.49	+2.	2.54	+1.13	GspH/FimT family pseudopilin
Smlt1628	231.59	253.69	+1.1	217.76	-1.06	excinuclease ABC subunit UvrB
Smlt1629	0.00	0.00	0	0.00	0	hypothetical protein
Smlt1630	72.20	204.24	+2.83	34.49	-2.09	hypothetical protein
Smlt1631	19.84	35.76	+1.8	44.63	+2.25	TlpA family protein disulfide reductase
Smlt1632	35.83	49.56	+1.38	44.18	+1.23	hypothetical protein
Smlt1633	16.93	8.17	-2.07	24.60	+1.45	hypothetical protein
Smlt1634	0.00	0.00	0	0.00	0	MipA/OmpV family protein
Smlt1635	30.29	21.54	-1.41	33.97	+1.12	response regulator transcription factor
Smlt1636	34.11	28.91	-1.18	40.25	+1.18	HAMP domain-containing histidine kinase
Smlt1637	7.70	10.16	+1.32	4.02	-1.91	DNA internalization-related competence protein ComEC/Rec2
Smlt1638	381.64	395.51	+1.04	581.65	+1.52	MotA/TolQ/ExbB proton channel family protein
Smlt1639	59.53	51.33	-1.16	106.99	+1.8	biopolymer transporter ExbD
Smlt1640	175.49	182.23	+1.04	245.32	+1.4	lipid A export permease/ATP-binding protein MsbA
Smlt1641	98.01	116.96	+1.19	128.93	+1.32	tetraacyldisaccharide 4'-kinase
Smlt1642	84.55	134.68	+1.59	88.57	+1.05	3-deoxy-manno-octulosonate cytidylyltransferase
Smlt1643	50.57	96.46	+1.91	73.77	+1.46	low molecular weight phosphotyrosine protein phosphatase
Smlt1644	83.93	86.37	+1.03	93.42	+1.11	hypothetical protein
Smlt1645	89.70	96.31	+1.07	113.05	+1.26	excinuclease ABC subunit UvrC
Smlt1646	186.51	227.69	+1.22	178.78	-1.04	CDP-diacylglycerol--glycerol-3-phosphate 3-phosphatidyltransferase
Smlt1648	2.93	1.95	-1.5	1.99	-1.47	NAD(P)/FAD-dependent oxidoreductase
Smlt1651	74.98	263.67	+3.52	132.52	+1.77	efflux transporter outer membrane subunit
Smlt1652	46.63	144.06	+3.09	78.10	+1.68	ABC transporter permease

Smlt1653	49.64	127.24	+2.56	110.77	+2.23	ABC transporter ATP-binding protein
Smlt1654	63.77	186.62	+2.93	130.99	+2.05	HlyD family efflux transporter periplasmic adaptor subunit
Smlt1655	0.00	0.00	0	0.00	0	hypothetical protein
Smlt1656	0.00	0.00	0	0.00	0	hypothetical protein
Smlt1657	0.00	0.00	0	0.00	0	hypothetical protein
Smlt1658	0.00	0.00	0	0.00	0	hypothetical protein
Smlt1659	0.32	0.36	+1.1	0.12	-2.6	hypothetical protein
Smlt1660	0.00	0.00	0	0.00	0	modification methylase
Smlt1661	0.00	0.00	0	0.00	0	hypothetical protein
Smlt1662	0.00	0.00	0	0.00	0	IS3-like element ISStma9 family transposase
Smlt1663	0.00	0.00	0	0.00	0	ISXac3 like transposase
Smlt1664	0.00	0.00	0	0.00	0	hypothetical protein
Smlt1665	0.00	0.00	0	0.00	0	conserved hypothetical protein
Smlt1666	0.00	0.00	0	0.00	0	conserved hypothetical protein
Smlt1667	0.00	0.00	0	0.00	0	NUDIX domain-containing protein
Smlt1668	0.00	0.00	0	0.00	0	GNAT family N-acetyltransferase
Smlt1669	0.00	0.00	0	0.00	0	GNAT family N-acetyltransferase
Smlt1670	0.00	0.00	0	0.00	0	DUF4124 domain-containing protein
Smlt1671	0.00	0.00	0	0.00	0	flavin reductase
Smlt1672	0.00	0.00	0	0.00	0	winged helix-turn-helix domain-containing protein
Smlt1674	385.36	1111.71	+2.88	574.53	+1.49	hypothetical protein
Smlt1675	12.74	26.11	+2.05	22.36	+1.75	hypothetical protein
Smlt1677	56.51	109.32	+1.93	155.36	+2.75	ATP-binding protein
Smlt1678	55.93	103.40	+1.85	28.97	-1.93	LysR family transcriptional regulator
Smlt1679	8.87	5.30	-1.67	7.39	-1.2	aldo/keto reductase
Smlt1680	8.26	7.49	-1.1	8.32	+1.01	SRPBCC family protein
Smlt1681	5.64	5.88	+1.04	3.57	-1.58	DUF6265 family protein
Smlt1682	65.99	123.79	+1.88	105.37	+1.6	AraC family transcriptional regulator
Smlt1683	68.11	80.65	+1.18	34.13	-2.	fluoride efflux transporter CrcB

Smlt1684	77.65	156.06	+2.01	81.39	+1.05	transcriptional regulator
Smlt1685	6.46	9.11	+1.41	5.93	-1.09	hypothetical protein
Smlt1686	409.11	1212.79	+2.96	454.89	+1.11	hypothetical protein
Smlt1687	142.56	60.32	-2.36	64.80	-2.2	4Fe-4S binding protein
Smlt1688	124.09	81.75	-1.52	106.34	-1.17	hypothetical protein
Smlt1689	324.48	340.94	+1.05	343.71	+1.06	group III truncated hemoglobin
Smlt1690	1352.85	1307.78	-1.03	372.56	-3.63	oxygen-independent coproporphyrinogen III oxidase
Smlt1691	1199.75	792.34	-1.51	344.28	-3.48	BLUF domain-containing protein
Smlt1692	195.84	233.03	+1.19	262.90	+1.34	nucleotide sugar dehydrogenase
Smlt1693	220.80	157.16	-1.4	106.00	-2.08	FMN-binding glutamate synthase family protein
Smlt1694	170.54	138.11	-1.23	82.15	-2.08	GNAT family N-acetyltransferase
Smlt1695	18.24	12.93	-1.41	18.62	+1.02	TIGR03862 family flavoprotein
Smlt1696	16.81	12.24	-1.37	26.51	+1.58	FKBP-type peptidyl-prolyl cis-trans isomerase
Smlt1697	260.18	554.85	+2.13	225.06	-1.16	DUF6164 family protein
Smlt1699	383.16	616.58	+1.61	297.58	-1.29	sulfurtransferase
Smlt1700	56.17	80.85	+1.44	29.29	-1.92	LLM class flavin-dependent oxidoreductase
Smlt1701	10.60	26.56	+2.51	47.30	+4.46	pyridoxamine 5'-phosphate oxidase family protein
Smlt1703	48.44	110.29	+2.28	74.14	+1.53	exopolysaccharide biosynthesis protein
Smlt1704	172.46	287.13	+1.66	221.86	+1.29	hemolysin family protein
Smlt1705	465.00	397.12	-1.17	506.78	+1.09	DUF47 family protein
Smlt1706	370.19	310.08	-1.19	359.50	-1.03	inorganic phosphate transporter
Smlt1707	8.66	15.23	+1.76	38.40	+4.43	S10 family peptidase
Smlt1708	24.35	12.36	-1.97	24.07	-1.01	GNAT family N-acetyltransferase
Smlt1709	178.80	202.83	+1.13	278.37	+1.56	DNA topoisomerase IV subunit B
Smlt1710	0.00	0.00	0	0.00	0	hypothetical protein
Smlt1711	447.12	537.63	+1.2	385.26	-1.16	CTP synthase
Smlt1712	315.37	433.08	+1.37	249.01	-1.27	3-deoxy-8-phosphooctulonate synthase
Smlt1713	84.71	121.92	+1.44	54.20	-1.56	hypothetical protein
Smlt1714	63.06	71.60	+1.14	49.43	-1.28	hypothetical protein

Smlt1715	256.57	271.97	+1.06	350.59	+1.37	phosphopyruvate hydratase
Smlt1716	170.81	210.57	+1.23	222.54	+1.3	cell division protein FtsB
Smlt1717	127.39	132.47	+1.04	108.60	-1.17	2-C-methyl-D-erythritol 4-phosphate cytidylyltransferase
Smlt1718	91.60	79.35	-1.15	96.29	+1.05	2-C-methyl-D-erythritol 2,4-cyclodiphosphate synthase
Smlt1719	139.76	129.38	-1.08	138.77	-1.01	tRNA pseudouridine(13) synthase TruD
Smlt1720	41.33	35.43	-1.17	55.53	+1.34	Smr/MutS family protein
Smlt1721	663.00	661.71	-1.	730.53	+1.1	5'/3'-nucleotidase SurE
Smlt1722	313.70	331.30	+1.06	313.58	-1.	protein-L-isoaspartate(D-aspartate) O-methyltransferase
Smlt1723	223.74	238.62	+1.07	225.10	+1.01	DedA family protein
Smlt1724	402.78	573.63	+1.42	589.05	+1.46	peptidoglycan DD-metalloendopeptidase family protein
Smlt1725	58.69	81.07	+1.38	66.30	+1.13	hypothetical protein
Smlt1726	70.37	77.87	+1.11	53.70	-1.31	Mth938-like domain-containing protein
Smlt1727	82.29	84.28	+1.02	84.55	+1.03	ribosome assembly RNA-binding protein YhbY
Smlt1728	154.96	351.30	+2.27	155.26	+1.	23S rRNA (uridine(2552)-2'-O)-methyltransferase RlmE
Smlt1729	650.04	1499.88	+2.31	637.35	-1.02	ATP-dependent zinc metalloprotease FtsH
Smlt1730	211.19	278.20	+1.32	165.58	-1.28	hypothetical protein
Smlt1731	0.00	0.00	0	0.00	0	hypothetical protein
Smlt1733	0.00	0.00	0	0.00	0	IS110-like element ISStma7 family transposase
Smlt1734	127.43	101.37	-1.26	188.02	+1.48	dihydropteroate synthase
Smlt1735	59.59	75.69	+1.27	67.39	+1.13	tRNA (adenosine(37)-N6)-dimethylallyltransferase MiaA
Smlt1736	747.13	1153.85	+1.54	975.53	+1.31	RNA chaperone Hfq
Smlt1737	367.15	406.00	+1.11	342.79	-1.07	GTPase HflX
Smlt1738	123.38	148.40	+1.2	122.57	-1.01	nicotinamide-nucleotide amidohydrolase family protein
Smlt1739	44.93	79.83	+1.78	58.07	+1.29	2-polyprenylphenol 6-hydroxylase
Smlt1740	122.81	217.14	+1.77	75.53	-1.63	transcriptional repressor LexA
Smlt1741	775.31	1568.17	+2.02	371.24	-2.09	recombinase RecA
Smlt1742	356.14	592.33	+1.66	194.04	-1.84	recombination regulator RecX
Smlt1743	303.43	419.11	+1.38	250.63	-1.21	alanine--tRNA ligase
Smlt1744	711.09	906.56	+1.27	1053.97	+1.48	carbon storage regulator CsrA

Smlt1745	0.00	0.00	0	0.00	0	hypothetical protein
Smlt1746	22.58	17.52	-1.29	18.86	-1.2	TonB-dependent outer membrane receptor
Smlt1747	59.94	55.12	-1.09	27.05	-2.22	hypothetical protein
Smlt1748	7.11	3.39	-2.1	5.57	-1.28	pyruvate dehydrogenase (acetyl-transferring), homodimeric type
Smlt1749	8.88	1.25	-7.1	5.59	-1.59	hypothetical protein
Smlt1750	5.99	1.93	-3.1	1.57	-3.81	FecI-like RNA polymerase sigma factor
Smlt1751	0.81	2.11	+2.61	0.45	-1.79	FecR-like protein
Smlt1753	1.05	0.82	-1.28	1.22	+1.16	FecA-like TonB-dependent outer membrane receptor
Smlt1754	3.45	3.91	+1.13	5.30	+1.54	alkaline phosphatase D family protein
Smlt1755	31.21	40.31	+1.29	42.79	+1.37	phospholipase C, phosphocholine-specific
Smlt1756	38.19	20.07	-1.9	5.81	-6.57	TonB-dependent outer membrane receptor
Smlt1757	869.70	282.16	-3.08	224.03	-3.88	c-type cytochrome
Smlt1758	396.68	174.42	-2.27	252.28	-1.57	cytochrome c
Smlt1759	52.76	25.20	-2.09	27.03	-1.95	alpha/beta fold hydrolase
Smlt1760	26.79	11.02	-2.43	23.51	-1.14	MFS transporter
Smlt1761	13.00	16.14	+1.24	21.69	+1.67	alpha/beta hydrolase
Smlt1762	3.08	27.32	+8.88	263.39	+85.59	TonB-dependent outer membrane receptor
Smlt1763	6.12	5.81	-1.05	6.89	+1.13	pirin family protein
Smlt1764	0.00	0.00	0	0.00	0	LysR-family transcriptional regulator
Smlt1766	101.10	90.73	-1.11	73.51	-1.38	alpha/beta fold hydrolase
Smlt1767	66.55	126.02	+1.89	97.44	+1.46	bifunctional hydroxymethylpyrimidine kinase/phosphomethylpyrimidine kinase
Smlt1768	27.39	49.99	+1.83	45.52	+1.66	DUF1275 domain-containing protein
Smlt1769	74.95	116.83	+1.56	106.98	+1.43	hypothetical protein
Smlt1770	120.98	144.06	+1.19	155.50	+1.29	PhoH family protein
Smlt1771	58.27	55.98	-1.04	87.16	+1.5	peroxiredoxin
Smlt1772	87.22	101.73	+1.17	115.58	+1.33	glycine cleavage system protein R
Smlt1773	216.49	231.90	+1.07	263.09	+1.22	4-hydroxy-tetrahydrodipicolinate synthase
Smlt1774	694.05	665.71	-1.04	503.89	-1.38	hypothetical protein

Smlt1774B	245.27	133.78	-1.83	216.94	-1.13	ferredoxin family protein
Smlt1775	32.80	52.48	+1.6	51.48	+1.57	sugar porter family MFS transporter
Smlt1777	153.47	154.51	+1.01	192.23	+1.25	polynucleotide adenylyltransferase PcnB
Smlt1778	124.11	120.51	-1.03	177.18	+1.43	2-amino-4-hydroxy-6-hydroxymethyldihydropteridine diphosphokinase
Smlt1779	137.76	120.10	-1.15	141.71	+1.03	3-methyl-2-oxobutanoate hydroxymethyltransferase
Smlt1780	181.04	179.76	-1.01	183.53	+1.01	pantoate--beta-alanine ligase
Smlt1781	30.73	29.90	-1.03	28.68	-1.07	hypothetical protein
Smlt1782	145.65	97.82	-1.49	393.30	+2.7	aspartate 1-decarboxylase
Smlt1783	211.95	232.56	+1.1	269.82	+1.27	glucose-6-phosphate isomerase
Smlt1784	354.25	279.38	-1.27	392.83	+1.11	response regulator transcription factor
Smlt1785	84.06	73.29	-1.15	96.44	+1.15	HAMP domain-containing histidine kinase
Smlt1786	391.51	418.59	+1.07	463.71	+1.18	flavodoxin-dependent (E)-4-hydroxy-3-methylbut-2-enyl-diphosphate synthase
Smlt1787	175.02	156.51	-1.12	184.50	+1.05	phosphatase PAP2 family protein
Smlt1788	421.89	431.50	+1.02	373.48	-1.13	response regulator
Smlt1789	258.86	292.69	+1.13	92.32	-2.8	bifunctional 4-hydroxy-2-oxoglutarate aldolase/2-dehydro-3-deoxy-phosphogluconate aldolase
Smlt1790	96.79	150.20	+1.55	53.35	-1.81	phosphogluconate dehydratase
Smlt1791	32.95	43.27	+1.31	35.99	+1.09	6-phosphogluconolactonase
Smlt1792	107.86	119.43	+1.11	108.33	+1.	glucokinase
Smlt1793	188.54	166.27	-1.13	160.10	-1.18	glucose-6-phosphate dehydrogenase
Smlt1794	239.07	164.77	-1.45	165.41	-1.45	sn-glycerol-3-phosphate ABC transporter ATP-binding protein UgpC
Smlt1795	103.71	137.23	+1.32	97.83	-1.06	folate-binding protein YgfZ
Smlt1796	162.11	80.86	-2.	82.64	-1.96	succinate dehydrogenase, cytochrome b556 subunit
Smlt1797	168.33	109.85	-1.53	75.09	-2.24	succinate dehydrogenase, hydrophobic membrane anchor protein
Smlt1798	996.52	762.44	-1.31	652.11	-1.53	succinate dehydrogenase flavoprotein subunit
Smlt1799	591.85	385.38	-1.54	304.18	-1.95	succinate dehydrogenase iron-sulfur subunit
Smlt1800	265.25	156.70	-1.69	120.55	-2.2	succinate dehydrogenase assembly factor 2

Smlt1801	41.05	30.13	-1.36	13.20	-3.11	hypothetical protein
Smlt1802	242.58	337.93	+1.39	270.16	+1.11	lipoprotein-releasing ABC transporter permease subunit
Smlt1803	155.44	236.06	+1.52	168.20	+1.08	lipoprotein-releasing ABC transporter ATP-binding protein LolD
Smlt1804	59.31	93.55	+1.58	42.88	-1.38	DUF6491 family protein
Smlt1805	49.43	58.24	+1.18	31.87	-1.55	oxidative damage protection protein
Smlt1806	60.79	67.89	+1.12	43.73	-1.39	A/G-specific adenine glycosylase
Smlt1807	115.53	121.54	+1.05	95.70	-1.21	signal recognition particle-docking protein FtsY
Smlt1808	176.93	115.36	-1.53	76.59	-2.31	hypothetical protein
Smlt1809	392.00	574.70	+1.47	195.60	-2.	molecular chaperone HtpG
Smlt1810	44.27	35.63	-1.24	64.35	+1.45	16S rRNA (guanine(966)-N(2))-methyltransferase RsmD
Smlt1811	263.00	248.27	-1.06	373.36	+1.42	pantetheine-phosphate adenylyltransferase
Smlt1812	1777.93	1527.40	-1.16	2265.03	+1.27	hypothetical protein
Smlt1813	149.99	185.86	+1.24	236.72	+1.58	YfhL family 4Fe-4S dicluster ferredoxin
Smlt1814	141.47	221.80	+1.57	239.07	+1.69	gamma-glutamyltransferase
Smlt1815	321.11	151.12	-2.12	465.36	+1.45	MBL fold metallo-hydrolase
Smlt1816	23.59	19.81	-1.19	22.47	-1.05	PsiF family protein
Smlt1817	41.06	72.45	+1.76	51.83	+1.26	metalloregulator ArsR/SmtB family transcription factor
Smlt1818	138.85	245.94	+1.77	95.85	-1.45	SRPBCC family protein
Smlt1819	125.66	79.51	-1.58	144.09	+1.15	uracil phosphoribosyltransferase
Smlt1820	7.88	8.92	+1.13	9.28	+1.18	TonB-dependent outer membrane receptor
Smlt1821	9.23	5.87	-1.57	11.34	+1.23	phytase
Smlt1822	7.99	9.24	+1.16	12.13	+1.52	metallophosphoesterase family protein
Smlt1823	32.61	35.44	+1.09	36.37	+1.12	inorganic diphosphatase
Smlt1824	11.99	3.98	-3.01	9.76	-1.23	hypothetical protein
Smlt1825	38.23	28.91	-1.32	38.84	+1.02	DNA photolyase family protein
Smlt1826	1132.78	1172.11	+1.03	1222.45	+1.08	OmpA family lipoprotein
Smlt1827	46.46	87.30	+1.88	69.34	+1.49	LysR family transcriptional regulator
Smlt1828	5.62	6.57	+1.17	10.67	+1.9	hypothetical protein
Smlt1829	1.37	4.32	+3.15	9.85	+7.19	SDR family NAD(P)-dependent oxidoreductase

Smlt1830	2.96	6.19	+2.09	25.26	+8.54	efflux RND transporter periplasmic adaptor subunit
Smlt1831	1.59	2.02	+1.27	9.38	+5.89	multidrug efflux RND transporter permease subunit
Smlt1832	3.06	2.48	-1.24	19.32	+6.3	SDR family oxidoreductase
Smlt1833	3.61	5.79	+1.61	21.25	+5.89	efflux transporter outer membrane subunit
Smlt1834	39.29	22.01	-1.79	38.40	-1.02	VOC family protein
Smlt1835	4.00	11.03	+2.76	6.37	+1.59	hypothetical protein
Smlt1836	73.18	28.71	-2.55	50.12	-1.46	endonuclease/exonuclease/phosphatase family protein
Smlt1837	0.00	0.00	0	0.00	0	hypothetical protein
Smlt1838	0.00	0.00	0	0.00	0	hypothetical protein
Smlt1839	0.00	0.00	0	0.00	0	LuxR C-terminal-related transcriptional regulator
Smlt1840	36.01	51.65	+1.43	43.41	+1.21	protein deglycase HchA
Smlt1841	54.44	46.41	-1.17	71.80	+1.32	alpha/beta fold hydrolase
Smlt1842	53.53	166.75	+3.12	49.17	-1.09	isoprenylcysteine carboxylmethyltransferase family protein
Smlt1843	0.00	0.00	0	0.00	0	hypothetical protein
Smlt1844	0.00	0.00	0	0.00	0	modification methylase
Smlt1844A	0.00	0.00	0	0.00	0	modification methylase
Smlt1844B	0.00	0.00	0	0.00	0	hypothetical protein
Smlt1845	0.00	0.00	0	0.00	0	ISXac3 like transposase
Smlt1846	0.00	0.00	0	0.00	0	IS3-like element ISStma9 family transposase
Smlt1846A	0.00	0.00	0	0.00	0	hypothetical protein
Smlt1846B	0.00	0.00	0	0.00	0	AAA family ATPase
Smlt1849	0.00	0.00	0	0.00	0	transmembrane protein
Smlt1849A	0.00	0.00	0	0.00	0	hypothetical protein
Smlt1850	0.00	0.00	0	0.00	0	hypothetical protein
Smlt1851	0.00	0.00	0	0.00	0	lysozyme
Smlt1852	0.00	0.00	0	0.00	0	DUF6127 family protein
Smlt1853	0.00	0.00	0	0.00	0	DUF2793 domain-containing protein
Smlt1854	0.00	0.00	0	0.00	0	phage tail protein
Smlt1855	0.00	0.00	0	0.00	0	hypothetical protein

Smlt1856	0.00	0.00	0	0.00	0	hypothetical protein
Smlt1857	0.00	0.00	0	0.00	0	DUF2163 domain-containing protein
Smlt1858	0.00	0.00	0	0.00	0	hypothetical protein
Smlt1859	0.00	0.00	0	0.00	0	hypothetical protein
Smlt1860	0.00	0.00	0	0.00	0	tail protein
Smlt1861	0.00	0.00	0	0.00	0	DUF6441 family protein
Smlt1862	0.00	0.00	0	0.00	0	hypothetical protein
Smlt1863	0.00	0.00	0	0.00	0	hypothetical protein
Smlt1864	0.00	0.00	0	0.00	0	hypothetical protein
Smlt1865	0.00	0.00	0	0.00	0	hypothetical protein
Smlt1866	0.00	0.00	0	0.00	0	major capsid protein
Smlt1867	0.00	0.00	0	0.00	0	head decoration protein
Smlt1868	0.00	0.00	0	0.00	0	S49 family peptidase
Smlt1869	0.00	0.00	0	0.00	0	phage portal protein
Smlt1870	0.00	0.00	0	0.00	0	hypothetical protein
Smlt1871	0.00	0.00	0	0.00	0	hypothetical protein
Smlt1872	0.00	0.00	0	0.00	0	hypothetical protein
Smlt1873	0.00	0.00	0	0.00	0	phage terminase large subunit family protein
Smlt1874	0.00	0.00	0	0.00	0	hypothetical protein
Smlt1875	0.00	0.00	0	0.00	0	hypothetical protein
Smlt1876	0.00	0.00	0	0.00	0	DUF3489 domain-containing protein
Smlt1877	0.00	0.00	0	0.00	0	hypothetical protein
Smlt1878	0.00	0.00	0	0.00	0	transposition helper protein
Smlt1879	0.00	0.00	0	0.00	0	IS3 family transposase
Smlt1880	0.00	0.00	0	0.00	0	hypothetical protein
Smlt1881	0.00	0.00	0	0.00	0	site-specific DNA-methyltransferase
Smlt1882	0.00	0.00	0	0.00	0	site-specific DNA-methyltransferase
Smlt1883	0.00	0.00	0	0.00	0	DUF6362 family protein
Smlt1884	0.00	0.00	0	0.00	0	hypothetical protein

Smlt1885	0.00	0.00	0	0.00	0	hypothetical protein
Smlt1886	0.00	0.00	0	0.00	0	phage/plasmid primase, P4 family
Smlt1887	0.00	0.00	0	0.00	0	hypothetical protein
Smlt1888	0.00	0.00	0	0.00	0	isoleucyl-tRNA synthetase
Smlt1889	0.00	0.00	0	0.00	0	DUF6511 domain-containing protein
Smlt1890	0.00	0.00	0	0.00	0	hypothetical protein
Smlt1891	0.00	0.00	0	0.00	0	ATP-binding protein
Smlt1892	0.00	0.00	0	0.00	0	hypothetical protein
Smlt1893	0.00	0.00	0	0.00	0	hypothetical protein
Smlt1894	0.00	0.00	0	0.00	0	hypothetical protein
Smlt1895	0.00	0.00	0	0.00	0	hypothetical protein
Smlt1896	0.00	0.00	0	0.00	0	hypothetical protein
Smlt1897	0.00	0.00	0	0.00	0	hypothetical protein
Smlt1898	0.00	0.00	0	0.00	0	helix-turn-helix domain-containing protein
Smlt1900	0.00	0.00	0	0.00	0	DUF2924 domain-containing protein
Smlt1901	0.00	0.00	0	0.00	0	recombinase family protein
Smlt1902	0.00	0.00	0	0.00	0	hypothetical protein
Smlt1903	0.00	0.00	0	0.00	0	hypothetical protein
Smlt1905	0.00	0.00	0	0.00	0	hypothetical protein
Smlt1906	0.00	0.00	0	0.00	0	XRE family transcriptional regulator
Smlt1907	0.00	0.00	0	0.00	0	site-specific integrase
Smlt1908	0.00	0.00	0	0.00	0	hypothetical protein
Smlt1911	0.00	0.00	0	0.00	0	hypothetical protein
Smlt1912	0.00	0.00	0	0.00	0	hypothetical protein
Smlt1913	0.00	0.00	0	0.00	0	hypothetical protein
Smlt1914	0.00	0.00	0	0.00	0	hypothetical protein
Smlt1915	0.00	0.00	0	0.00	0	hypothetical protein
Smlt1916	0.00	0.00	0	0.00	0	HNH endonuclease
Smlt1917	0.00	0.00	0	0.00	0	hypothetical protein

Smlt1918	0.00	0.00	0	0.00	0	recombination-associated protein RdgC
Smlt1920	0.00	0.00	0	0.00	0	hypothetical protein
Smlt1921	0.00	0.00	0	0.00	0	hypothetical protein
Smlt1922	0.00	0.00	0	0.00	0	helix-turn-helix domain-containing protein
Smlt1923	0.00	0.00	0	0.00	0	hypothetical protein
Smlt1924	0.00	0.00	0	0.00	0	hypothetical protein
Smlt1925	0.00	0.00	0	0.00	0	helix-turn-helix domain-containing protein
Smlt1926	0.00	0.00	0	0.00	0	hypothetical protein
Smlt1927	0.00	0.00	0	0.00	0	hypothetical protein
Smlt1929	0.00	0.00	0	0.00	0	hypothetical protein
Smlt1930	0.00	0.00	0	0.00	0	hypothetical protein
Smlt1931	0.00	0.00	0	0.00	0	hypothetical protein
Smlt1932	0.00	0.00	0	0.00	0	helix-turn-helix domain-containing protein
Smlt1933	0.00	0.00	0	0.00	0	hypothetical protein
Smlt1934	0.00	0.00	0	0.00	0	hypothetical protein
Smlt1935	0.00	0.00	0	0.00	0	DUF1064 domain-containing protein
Smlt1936	0.00	0.00	0	0.00	0	hypothetical protein
Smlt1937	0.00	0.00	0	0.00	0	hypothetical protein
Smlt1938	0.00	0.00	0	0.00	0	hypothetical protein
Smlt1939	0.00	0.00	0	0.00	0	hypothetical protein
Smlt1940	0.00	0.00	0	0.00	0	hypothetical protein
Smlt1941	0.00	0.00	0	0.00	0	hypothetical protein
Smlt1944	0.00	0.00	0	0.00	0	lysozyme
Smlt1946	0.00	0.00	0	0.00	0	hypothetical protein
Smlt1947	0.00	0.00	0	0.00	0	hypothetical protein
Smlt1948	0.00	0.00	0	0.00	0	hypothetical protein
Smlt1949	0.00	0.00	0	0.00	0	hypothetical protein
Smlt1950	0.00	0.00	0	0.00	0	hypothetical protein
Smlt1951	0.00	0.00	0	0.00	0	hypothetical protein

Smlt1952	0.00	0.00	0	0.00	0	hypothetical protein
Smlt1953	0.00	0.00	0	0.00	0	terminase small subunit
Smlt1954	0.00	0.00	0	0.00	0	DNA packaging protein
Smlt1955	0.00	0.00	0	0.00	0	hypothetical protein
Smlt1956	0.00	0.00	0	0.00	0	hypothetical protein
Smlt1957	0.00	0.00	0	0.00	0	hypothetical protein
Smlt1958	0.00	0.00	0	0.00	0	hypothetical protein
Smlt1959	0.00	0.00	0	0.00	0	hypothetical protein
Smlt1960	0.00	0.00	0	0.00	0	hypothetical protein
Smlt1961	0.00	0.00	0	0.00	0	N4-gp56 family major capsid protein
Smlt1962	0.00	0.00	0	0.00	0	hypothetical protein
Smlt1963	0.00	0.00	0	0.00	0	hypothetical protein
Smlt1964	0.00	0.00	0	0.00	0	hypothetical protein
Smlt1965	0.00	0.00	0	0.00	0	hypothetical protein
Smlt1967	0.00	0.00	0	0.00	0	hypothetical protein
Smlt1968	0.00	0.00	0	0.00	0	hypothetical protein
Smlt1969	0.00	0.00	0	0.00	0	hypothetical protein
Smlt1970	0.00	0.00	0	0.00	0	hypothetical protein
Smlt1971	0.00	0.00	0	0.00	0	hypothetical protein
Smlt1972	0.00	0.00	0	0.00	0	hypothetical protein
Smlt1973	0.00	0.00	0	0.00	0	hypothetical protein
Smlt1974	0.00	0.00	0	0.00	0	hypothetical protein
Smlt1975	0.00	0.00	0	0.00	0	hypothetical protein
Smlt1976	0.00	0.00	0	0.00	0	conserved hypothetical protein
Smlt1977	0.00	0.00	0	0.00	0	hypothetical protein
Smlt1978	0.00	0.00	0	0.00	0	SOS response-associated peptidase family protein
Smlt1979	0.00	0.00	0	0.00	0	conserved hypothetical protein
Smlt1980	0.00	0.00	0	0.00	0	hypothetical protein
Smlt1982	27.63	38.91	+1.41	64.22	+2.32	hypothetical protein

Smlt1983	299.79	506.09	+1.69	310.60	+1.04	SsrA-binding protein SmpB
Smlt1983A	54.69	46.48	-1.18	83.72	+1.53	type II toxin-antitoxin system RatA family toxin
Smlt1984	27.76	25.80	-1.08	44.70	+1.61	RnfH family protein
Smlt1985	289.51	426.01	+1.47	367.22	+1.27	outer membrane protein assembly factor BamE
Smlt1986	929.70	1378.60	+1.48	2459.88	+2.65	ferric iron uptake transcriptional regulator
Smlt1987	0.00	0.00	0	0.00	0	SGNH/GDSL hydrolase family protein
Smlt1988	30.80	52.92	+1.72	20.48	-1.5	LysR family transcriptional regulator
Smlt1989	65.23	109.47	+1.68	61.28	-1.06	DNA repair protein RecN
Smlt1990	36.18	64.13	+1.77	38.03	+1.05	heat-inducible transcriptional repressor HrcA
Smlt1991	391.15	869.34	+2.22	254.24	-1.54	nucleotide exchange factor GrpE
Smlt1992	1181.16	2634.32	+2.23	561.62	-2.1	molecular chaperone DnaK
Smlt1993	445.47	630.42	+1.42	261.35	-1.7	molecular chaperone DnaJ
Smlt1994	0.00	0.00	0	0.00	0	helix-turn-helix transcriptional regulator
Smlt1995	3.52	2.50	-1.41	3.57	+1.02	MFS transporter
Smlt1997	80.21	64.07	-1.25	146.78	+1.83	pyridoxal kinase
Smlt1998	61.86	49.55	-1.25	66.47	+1.07	prephenate dehydrogenase
Smlt2000	44.85	89.17	+1.99	223.46	+4.98	hypothetical protein
Smlt2001	42.91	57.71	+1.35	48.99	+1.14	ATP-binding cassette domain-containing protein
Smlt2002	96.74	117.19	+1.21	94.54	-1.02	IMPACT family protein
Smlt2003	29.38	41.03	+1.4	41.18	+1.4	hypothetical protein
Smlt2004	48.09	53.06	+1.1	84.69	+1.76	RNA polymerase sigma factor
Smlt2005	52.61	21.34	-2.47	53.18	+1.01	GGDEF domain-containing protein
Smlt2006	208.67	187.53	-1.11	154.08	-1.35	TIGR00730 family Rossman fold protein
Smlt2007	430.32	605.64	+1.41	731.35	+1.7	aminotransferase class III-fold pyridoxal phosphate-dependent enzyme
Smlt2008	63.54	56.94	-1.12	74.58	+1.17	tRNA preQ1(34) S-adenosylmethionine ribosyltransferase-isomerase QueA
Smlt2009	144.19	118.65	-1.22	115.43	-1.25	tRNA guanosine(34) transglycosylase Tgt
Smlt2010	745.66	815.05	+1.09	759.85	+1.02	preprotein translocase subunit YajC

Smlt2011	399.59	508.97	+1.27	437.87	+1.1	protein translocase subunit SecD
Smlt2012	241.25	354.74	+1.47	275.14	+1.14	protein translocase subunit SecF
Smlt2015	416.11	320.36	-1.3	575.71	+1.38	trans-2-enoyl-CoA reductase family protein
Smlt2016	90.33	68.85	-1.31	96.59	+1.07	EAL domain-containing protein
Smlt2017	103.58	64.31	-1.61	101.74	-1.02	NAD(P)/FAD-dependent oxidoreductase
Smlt2018	276.86	112.44	-2.46	191.78	-1.44	YaeQ family protein
Smlt2019	0.00	0.00	0	0.00	0	pseudouridine synthase
Smlt2020	19.74	59.38	+3.01	20.92	+1.06	YaiI/YqxD family protein
Smlt2022	37650.23	22018.44	-1.71	24276.32	-1.55	cold-shock protein
Smlt2023	15.19	17.24	+1.14	18.60	+1.22	glutathione S-transferase
Smlt2024	93.45	166.44	+1.78	94.13	+1.01	YdiU family protein
Smlt2026	159.22	96.36	-1.65	94.42	-1.69	adenine phosphoribosyltransferase
Smlt2027	120.40	168.50	+1.4	138.83	+1.15	ATP-dependent RNA helicase DbpA
Smlt2028	45.73	61.55	+1.35	4.64	-9.85	YeiH family protein
Smlt2029	9.30	19.75	+2.13	7.97	-1.17	LysR family transcriptional regulator
Smlt2030	280.73	310.16	+1.1	226.74	-1.24	ATP-binding cassette domain-containing protein
Smlt2031	90.25	104.26	+1.16	19.32	-4.67	cytochrome c
Smlt2032	56.26	87.89	+1.56	37.07	-1.52	c-type cytochrome
Smlt2033	10.67	27.30	+2.56	18.84	+1.77	MexH family multidrug efflux RND transporter periplasmic adaptor subunit
Smlt2034	7.47	14.99	+2.01	11.11	+1.49	efflux RND transporter permease subunit
Smlt2035	8.60	17.95	+2.09	13.81	+1.61	efflux RND transporter permease subunit
Smlt2036	223.80	294.72	+1.32	352.02	+1.57	TraB/GumN family protein
Smlt2037	81.64	92.44	+1.13	98.10	+1.2	GNAT family N-acetyltransferase
Smlt2038	330.79	355.63	+1.08	257.85	-1.28	carbon-nitrogen hydrolase
Smlt2039	144.18	90.49	-1.59	58.70	-2.46	agmatine deiminase family protein
Smlt2040	323.29	123.78	-2.61	70.06	-4.61	hypothetical protein
Smlt2041	591.20	415.31	-1.42	274.10	-2.16	type B 50S ribosomal protein L36
Smlt2042	1231.36	1537.35	+1.25	1243.02	+1.01	(d)CMP kinase

Smlt2043	4670.48	5829.73	+1.25	4473.48	-1.04	30S ribosomal protein S1
Smlt2044	607.11	655.21	+1.08	479.76	-1.27	integration host factor subunit beta
Smlt2045	200.00	237.90	+1.19	274.38	+1.37	lipopolysaccharide assembly protein LapA domain-containing protein
Smlt2046	249.08	244.64	-1.02	267.66	+1.07	lipopolysaccharide assembly protein LapB
Smlt2047	67.02	75.80	+1.13	61.02	-1.1	glycosyltransferase family 4 protein
Smlt2048	160.84	174.12	+1.08	187.14	+1.16	polysaccharide biosynthesis protein
Smlt2049	293.52	288.86	-1.02	322.99	+1.1	UTP--glucose-1-phosphate uridylyltransferase GalU
Smlt2051	173.12	520.83	+3.01	165.16	-1.05	acetyl-CoA C-acyltransferase
Smlt2052	126.42	313.77	+2.48	149.38	+1.18	3-hydroxyacyl-CoA dehydrogenase/enoyl-CoA hydratase family protein
Smlt2053	45.06	131.29	+2.91	71.35	+1.58	TetR family transcriptional regulator
Smlt2054	547.42	314.81	-1.74	344.62	-1.59	nucleoside-diphosphate kinase
Smlt2055	158.48	149.55	-1.06	99.80	-1.59	23S rRNA (adenine(2503)-C(2))-methyltransferase RlmN
Smlt2056	109.07	146.28	+1.34	85.33	-1.28	tetratricopeptide repeat protein
Smlt2057	248.64	391.95	+1.58	283.76	+1.14	DUF4115 domain-containing protein
Smlt2058	677.38	1034.77	+1.53	878.26	+1.3	tetratricopeptide repeat protein
Smlt2059	925.71	1431.28	+1.55	1153.65	+1.25	outer membrane protein assembly factor BamB
Smlt2060	291.98	370.98	+1.27	251.74	-1.16	ribosome biogenesis GTPase Der
Smlt2061	1.01	3.66	+3.64	2.05	+2.04	hypothetical protein
Smlt2062	0.59	1.31	+2.21	1.14	+1.92	LysE family transporter
Smlt2063	11.73	15.70	+1.34	23.43	+2.	helix-turn-helix domain-containing protein
Smlt2064	47.81	36.73	-1.3	36.31	-1.32	molybdopterin-synthase adenylyltransferase MoeB
Smlt2065	0.00	0.00	0	0.00	0	molybdopterin biosynthesis protein, pseudogene
Smlt2066	37.47	49.83	+1.33	44.15	+1.18	monovalent cation:proton antiporter-2 (CPA2) family protein
Smlt2068	0.00	0.00	0	0.00	0	repeat protein
Smlt2069	84.23	85.94	+1.02	126.24	+1.5	bifunctional methylenetetrahydrofolate dehydrogenase/methenyltetrahydrofolate cyclohydrolase FolD

Smlt2071	446.79	373.51	-1.2	360.08	-1.24	IMP dehydrogenase
Smlt2072	278.38	462.66	+1.66	428.23	+1.54	glutamine-hydrolyzing GMP synthase
Smlt2074	0.00	0.00	0	0.00	0	hypothetical protein
Smlt2075	0.00	0.00	0	0.00	0	hypothetical protein
Smlt2076	0.00	0.00	0	0.00	0	RES family NAD+ phosphorylase
Smlt2077	0.00	0.00	0	0.00	0	conserved hypothetical protein
Smlt2077A	0.00	0.00	0	0.00	0	hypothetical protein
Smlt2079	0.00	0.00	0	0.00	0	hypothetical protein
Smlt2080	19.79	26.46	+1.34	20.55	+1.04	LysR family transcriptional regulator
Smlt2081	0.00	0.00	0	0.00	0	type 1 glutamine amidotransferase domain-containing protein
Smlt2082	0.00	0.00	0	0.00	0	hypothetical protein
Smlt2083	91.73	51.82	-1.77	67.30	-1.36	hypothetical protein
Smlt2084	0.00	0.00	0	0.00	0	3-hydroxybutyrate dehydrogenase
Smlt2085	1.38	1.96	+1.42	1.17	-1.18	membrane protein
Smlt2086	12.17	13.65	+1.12	14.79	+1.22	DUF4180 domain-containing protein
Smlt2087	40.71	51.20	+1.26	71.93	+1.77	alpha/beta hydrolase
Smlt2088	0.00	0.00	0	0.00	0	hypothetical protein
Smlt2089	41.89	27.01	-1.55	13.63	-3.07	SRPBCC family protein
Smlt2090	6.70	5.02	-1.34	3.13	-2.14	helix-turn-helix domain-containing protein
Smlt2091	0.00	0.00	0	0.00	0	SRPBCC domain-containing protein
Smlt2092	54.57	86.20	+1.58	67.99	+1.25	GNAT family N-acetyltransferase
Smlt2093	275.30	279.52	+1.02	240.94	-1.14	oleate hydratase
Smlt2094	78.60	80.50	+1.02	78.25	-1.	TetR/AcrR family transcriptional regulator
Smlt2095	1.29	3.56	+2.76	1.65	+1.28	hypothetical protein
Smlt2096	0.00	0.00	0	0.00	0	chemotaxis protein CheW
Smlt2097	0.00	0.00	0	0.00	0	BLUF domain-containing protein
Smlt2098	2.86	3.32	+1.16	3.59	+1.25	transcriptional regulator FtrA
Smlt2099	1.64	2.10	+1.28	1.64	+1.	TonB-dependent outer membrane receptor
Smlt2100	1.43	2.29	+1.6	2.29	+1.61	FMN reductase

Smlt2101	0.21	1.38	+6.62	3.27	+15.76	DUF3088 family protein
Smlt2102	0.69	1.19	+1.72	1.16	+1.67	MsnO8 family LLM class oxidoreductase
Smlt2103	1.80	1.98	+1.1	2.26	+1.26	LLM class flavin-dependent oxidoreductase
Smlt2104	2.54	3.59	+1.41	3.10	+1.22	M20 family metallopeptidase
Smlt2105	0.00	0.00	0	0.00	0	GNAT family N-acetyltransferase
Smlt2106	1.73	2.51	+1.45	2.71	+1.56	amino acid ABC transporter permease/ATP-binding protein
Smlt2107	1.70	2.81	+1.66	3.35	+1.97	ABC transporter substrate-binding protein
Smlt2108	24.79	15.31	-1.62	11.29	-2.2	discoidin domain-containing protein
Smlt2109	0.00	0.00	0	0.00	0	conserved hypothetical protein
Smlt2110	0.00	0.00	0	0.00	0	DNA-binding protein
Smlt2111	7.77	12.75	+1.64	4.06	-1.91	MBL fold metallo-hydrolase
Smlt2112	118.64	477.82	+4.03	68.74	-1.73	TetR/AcrR family transcriptional regulator
Smlt2113	0.00	0.00	0	0.00	0	EAL domain-containing protein
Smlt2114	0.00	0.00	0	0.00	0	LysR family transcriptional regulator
Smlt2115	0.00	0.00	0	0.00	0	MFS transporter
Smlt2116	0.00	0.00	0	0.00	0	SDR family oxidoreductase
Smlt2117	0.00	0.00	0	0.00	0	GNAT family N-acetyltransferase
Smlt2118	0.00	0.00	0	0.00	0	metalloregulator ArsR/SmtB family transcription factor
Smlt2119	0.00	0.00	0	0.00	0	DUF2938 domain-containing protein
Smlt2120	0.00	0.00	0	0.00	0	aminoglycoside O-phosphotransferase APH(3')-IIc
Smlt2121	0.00	0.00	0	0.00	0	DoxX family protein
Smlt2123	7.91	8.16	+1.03	6.42	-1.23	TetR/AcrR family transcriptional regulator
Smlt2124	8.30	8.52	+1.03	5.54	-1.5	DUF418 domain-containing protein
Smlt2125	185.19	284.34	+1.54	266.74	+1.44	aminoglycoside phosphotransferase family protein
Smlt2126	41.18	33.82	-1.22	71.58	+1.74	DMT family transporter
Smlt2127	135.17	122.75	-1.1	143.17	+1.06	UDP-N-acetylmuramate dehydrogenase
Smlt2128	162.93	137.71	-1.18	157.49	-1.03	quinone-dependent dihydroorotate dehydrogenase
Smlt2129	57.30	61.18	+1.07	68.58	+1.2	class I SAM-dependent methyltransferase
Smlt2130	70.11	66.55	-1.05	63.44	-1.11	DUF4190 domain-containing protein

Smlt2131	55.11	89.33	+1.62	85.23	+1.55	SDR family oxidoreductase
Smlt2132	313.04	356.98	+1.14	446.13	+1.43	aldehyde dehydrogenase family protein
Smlt2133	12.92	20.80	+1.61	19.05	+1.47	hypothetical protein
Smlt2134	7.30	11.27	+1.54	9.35	+1.28	GNAT family N-acetyltransferase
Smlt2135	11.83	17.20	+1.45	11.47	-1.03	GNAT family N-acetyltransferase
Smlt2136	21.67	23.26	+1.07	21.71	+1.	helix-turn-helix domain-containing protein
Smlt2137	596.54	960.68	+1.61	754.24	+1.26	universal stress protein
Smlt2138	66.49	153.18	+2.3	116.97	+1.76	transcriptional regulator
Smlt2139	86.71	57.54	-1.51	75.49	-1.15	response regulator
Smlt2140	71.83	36.24	-1.98	63.60	-1.13	PAS domain-containing sensor histidine kinase
Smlt2141	69.61	53.48	-1.3	69.30	-1.	response regulator
Smlt2142	59.49	45.98	-1.29	85.48	+1.44	chemotaxis protein CheR
Smlt2143	41.23	24.99	-1.65	69.11	+1.68	chemotaxis protein CheB
Smlt2144	53.92	34.55	-1.56	92.13	+1.71	hybrid sensor histidine kinase/response regulator
Smlt2144A	0.00	0.00	0	0.00	0	hypothetical protein
Smlt2148	8.22	26.18	+3.18	6.41	-1.28	metalloregulator ArsR/SmtB family transcription factor
Smlt2149	8.26	33.43	+4.05	3.97	-2.08	hypothetical protein
Smlt2150	4.92	11.45	+2.33	1.33	-3.7	membrane protein
Smlt2151	48.55	96.31	+1.98	23.39	-2.08	linear amide C-N hydrolase
Smlt2152	29.00	46.18	+1.59	48.47	+1.67	DUF6491 family protein
Smlt2153	122.32	188.88	+1.54	156.18	+1.28	helix-turn-helix domain-containing protein
Smlt2154	48.68	46.98	-1.04	6.22	-7.83	DMT family transporter
Smlt2156	17.10	26.83	+1.57	16.29	-1.05	bifunctional aspartate kinase/homoserine dehydrogenase I
Smlt2157	9.15	18.91	+2.07	9.72	+1.06	homoserine kinase
Smlt2158	11.60	32.13	+2.77	9.86	-1.18	threonine synthase
Smlt2159	70.39	93.80	+1.33	72.38	+1.03	helix-turn-helix domain-containing protein
Smlt2160	273.15	235.98	-1.16	296.98	+1.09	histidine--tRNA ligase
Smlt2161	49.57	188.52	+3.8	9.75	-5.09	helix-turn-helix domain-containing protein
Smlt2162	63.90	256.33	+4.01	11.37	-5.62	ATP phosphoribosyltransferase

Smlt2163	34.32	110.35	+3.21	7.09	-4.84	histidinol dehydrogenase
Smlt2164	43.45	125.82	+2.9	14.13	-3.07	histidinol-phosphate transaminase
Smlt2165	46.05	214.26	+4.65	23.63	-1.95	bifunctional histidinol-phosphatase/imidazoleglycerol-phosphate dehydratase HisB
Smlt2166	24.63	145.83	+5.92	16.43	-1.5	imidazole glycerol phosphate synthase subunit HisH
Smlt2167	16.62	113.19	+6.81	10.13	-1.64	1-(5-phosphoribosyl)-5-[(5-phosphoribosylamino)methylideneamino]imidazole-4-carboxamide isomerase
Smlt2168	22.12	150.11	+6.79	16.44	-1.35	imidazole glycerol phosphate synthase subunit HisF
Smlt2169	92.15	401.10	+4.35	95.78	+1.04	bifunctional phosphoribosyl-AMP cyclohydrolase/phosphoribosyl-ATP diphosphatase HisIE
Smlt2170	178.63	93.34	-1.91	189.47	+1.06	calcineurin-like phosphoesterase family protein
Smlt2171	60.58	80.27	+1.33	106.84	+1.76	hypothetical protein
Smlt2172	81.59	60.50	-1.35	125.83	+1.54	glucokinase
Smlt2173	0.00	0.00	0	0.00	0	N(4)-(beta-N-acetylglucosaminyl)-L-asparaginase
Smlt2174	29.35	37.16	+1.27	48.17	+1.64	copper homeostasis protein CutC
Smlt2175	55.82	72.14	+1.29	125.63	+2.25	TonB-dependent outer membrane receptor
Smlt2176	26.10	34.45	+1.32	46.60	+1.79	heavy metal translocating P-type ATPase
Smlt2177	38.52	50.90	+1.32	49.56	+1.29	Cu(I)-responsive transcriptional regulator
Smlt2178	107.87	46.08	-2.34	169.68	+1.57	heavy-metal-associated domain-containing protein
Smlt2179	208.70	201.09	-1.04	278.98	+1.34	TonB-dependent outer membrane receptor
Smlt2180	198.49	461.37	+2.32	330.15	+1.66	GH92 family glycosyl hydrolase
Smlt2181	73.17	171.06	+2.34	194.29	+2.66	LacI family DNA-binding transcriptional regulator
Smlt2182	70.40	191.77	+2.72	183.03	+2.6	L-fucose:H+ symporter permease
Smlt2183	58.74	142.08	+2.42	162.70	+2.77	carbohydrate kinase
Smlt2184	78.16	189.64	+2.43	221.25	+2.83	AGE family epimerase/isomerase
Smlt2185	31.18	87.23	+2.8	102.62	+3.29	glycoside hydrolase family 2 protein
Smlt2187	190.55	129.93	-1.47	183.50	-1.04	acireductone synthase
Smlt2188	78.77	52.18	-1.51	56.49	-1.39	acireductone dioxygenase
Smlt2189	35.82	23.84	-1.5	34.25	-1.05	methylthioribulose 1-phosphate dehydratase

Smlt2190	60.92	200.99	+3.3	86.38	+1.42	hypothetical protein
Smlt2191	0.00	0.00	0	0.00	0	hypothetical protein
Smlt2192	49.16	92.51	+1.88	121.05	+2.46	amino acid permease
Smlt2193	134.85	222.02	+1.65	344.36	+2.55	amino acid permease
Smlt2194	119.65	109.71	-1.09	126.92	+1.06	NUDIX hydrolase
Smlt2195	73.94	72.68	-1.02	132.60	+1.79	phosphoglycerate dehydrogenase
Smlt2196	77.08	105.59	+1.37	146.64	+1.9	FAD-binding oxidoreductase
Smlt2197	49.26	100.39	+2.04	89.04	+1.81	DUF2388 domain-containing protein
Smlt2198	17.83	46.34	+2.6	30.18	+1.69	DUF4105 domain-containing protein
Smlt2199	81.56	113.01	+1.39	147.77	+1.81	HAMP domain-containing protein
Smlt2200	68.35	126.24	+1.85	101.18	+1.48	response regulator
Smlt2201	389.28	994.32	+2.55	402.06	+1.03	efflux RND transporter periplasmic adaptor subunit
Smlt2202	376.76	983.58	+2.61	317.02	-1.19	multidrug efflux RND transporter permease subunit
Smlt2203	193.77	408.77	+2.11	115.01	-1.68	nuclear transport factor 2 family protein
Smlt2204	879.07	1376.30	+1.57	487.88	-1.8	MliC family protein
Smlt2205	333.76	302.11	-1.1	677.57	+2.03	elongation factor P-like protein YeiP
Smlt2206	140.33	183.00	+1.3	229.87	+1.64	SDR family oxidoreductase
Smlt2207	51.86	109.35	+2.11	60.14	+1.16	hydroxymethylglutaryl-CoA lyase
Smlt2208	90.21	167.63	+1.86	122.60	+1.36	VOC family protein
Smlt2209	92.21	254.01	+2.75	148.57	+1.61	enoyl-CoA hydratase/isomerase family protein
Smlt2210	37.85	37.52	-1.01	100.89	+2.67	ferrous iron transport protein A
Smlt2211	67.11	103.17	+1.54	107.23	+1.6	ferrous iron transport protein B
Smlt2212	65.05	125.61	+1.93	81.69	+1.26	hypothetical protein
Smlt2213	1.96	1.62	-1.21	1.00	-1.95	DUF4304 domain-containing protein
Smlt2214	2.72	2.43	-1.12	3.68	+1.35	NAD(P)H-dependent oxidoreductase
Smlt2215	9.33	7.35	-1.27	11.93	+1.28	MerR family transcriptional regulator
Smlt2216	22.72	16.32	-1.39	34.02	+1.5	3-oxoacyl-ACP reductase
Smlt2217	157.93	166.27	+1.05	225.03	+1.42	4-hydroxy-tetrahydrodipicolinate reductase

Smlt2218	640.60	548.13	-1.17	433.98	-1.48	glutamine-hydrolyzing carbamoyl-phosphate synthase small subunit
Smlt2219	239.67	337.93	+1.41	257.77	+1.08	carbamoyl-phosphate synthase large subunit
Smlt2220	360.70	518.34	+1.44	338.51	-1.07	transcription elongation factor GreA
Smlt2221	94.20	143.10	+1.52	95.72	+1.02	hypothetical protein
Smlt2222	59.17	40.36	-1.47	106.51	+1.8	DUF1176 domain-containing protein
Smlt2223	108.93	142.72	+1.31	156.19	+1.43	single-stranded-DNA-specific exonuclease RecJ
Smlt2224	21.84	31.87	+1.46	35.48	+1.62	DUF2135 domain-containing protein
Smlt2225	0.00	0.00	0	0.00	0	acyltransferase family protein
Smlt2226	0.00	0.00	0	0.00	0	LytTR family transcriptional regulator
Smlt2227	13049.53	10258.26	-1.27	12847.08	-1.02	peptide chain release factor 2
Smlt2229	132.59	133.34	+1.01	162.59	+1.23	helix-turn-helix domain-containing protein
Smlt2230	22.90	38.18	+1.67	35.47	+1.55	NAD-dependent succinate-semialdehyde dehydrogenase
Smlt2231	53.07	68.59	+1.29	74.22	+1.4	acetolactate synthase large subunit
Smlt2232	518.80	436.21	-1.19	661.69	+1.28	lysine--tRNA ligase
Smlt2233	0.00	0.00	0	0.00	0	two-component system response regulator
Smlt2234	21.20	25.93	+1.22	19.89	-1.07	response regulator
Smlt2235	13.17	20.27	+1.54	19.58	+1.49	crotonase/enoyl-CoA hydratase family protein
Smlt2236	370.73	866.71	+2.34	395.26	+1.07	long-chain-fatty-acid--CoA ligase
Smlt2237	47.89	55.61	+1.16	86.44	+1.8	choline dehydrogenase
Smlt2238	70.99	50.77	-1.4	99.55	+1.4	betaine-aldehyde dehydrogenase
Smlt2239	115.07	43.64	-2.64	199.53	+1.73	transcriptional regulator BetI
Smlt2240	5.93	5.85	-1.01	37.45	+6.32	BCCT family transporter
Smlt2241	322.77	321.99	-1.	61.50	-5.25	aconitate hydratase AcnA
Smlt2242	24.73	23.03	-1.07	18.46	-1.34	outer membrane protein assembly factor BamE
Smlt2243	52.27	29.21	-1.79	38.24	-1.37	AbrB/MazE/SpoVT family DNA-binding domain-containing protein
Smlt2244	70.67	40.24	-1.76	69.06	-1.02	type II toxin-antitoxin system VapC family toxin

Smlt2245	327.88	192.61	-1.7	157.90	-2.08	bifunctional aconitate hydratase 2/2-methylisocitrate dehydratase
Smlt2246	36.22	4.84	-7.48	32.81	-1.1	hypothetical protein
Smlt2247	11.73	4.14	-2.83	12.29	+1.05	hypothetical protein
Smlt2248	17.34	3.49	-4.97	13.92	-1.25	chemotaxis response regulator protein-glutamate methylesterase
Smlt2249	14.78	1.39	-10.6	10.00	-1.48	chemoreceptor glutamine deamidase CheD
Smlt2250	12.39	3.06	-4.05	8.49	-1.46	chemotaxis protein CheR
Smlt2251	76.43	12.05	-6.34	47.82	-1.6	methyl-accepting chemotaxis protein
Smlt2253	0.00	0.00	0	0.00	0	IS110-like element ISStma7 family transposase
Smlt2254	105.80	25.88	-4.09	99.53	-1.06	methyl-accepting chemotaxis protein
Smlt2255	136.66	17.98	-7.6	80.73	-1.69	hypothetical protein
Smlt2256	102.21	9.47	-10.79	54.18	-1.89	chemotaxis protein CheW
Smlt2257	29.71	15.59	-1.91	22.05	-1.35	flagellar brake protein
Smlt2258	19.07	4.14	-4.6	25.78	+1.35	methyl-accepting chemotaxis protein
Smlt2260	158.10	35.42	-4.46	77.70	-2.03	chemotaxis protein CheA
Smlt2261	259.35	39.78	-6.52	131.80	-1.97	response regulator
Smlt2262	468.24	51.96	-9.01	267.04	-1.75	STAS domain-containing protein
Smlt2263	0.00	0.00	0	0.00	0	chemotaxis protein CheW
Smlt2264	42.27	26.46	-1.6	41.48	-1.02	ParA family protein
Smlt2265	27.65	16.12	-1.72	26.61	-1.04	flagellar motor protein MotD
Smlt2266	115.28	59.17	-1.95	85.67	-1.35	flagellar motor protein
Smlt2267	40.95	66.28	+1.62	63.06	+1.54	chemotaxis protein CheA
Smlt2268	104.01	136.46	+1.31	136.26	+1.31	protein phosphatase CheZ
Smlt2269	70.22	83.62	+1.19	74.46	+1.06	chemotaxis response regulator CheY
Smlt2270	41.45	72.01	+1.74	46.58	+1.12	RNA polymerase sigma factor FliA
Smlt2271	57.57	87.60	+1.52	58.86	+1.02	P-loop NTPase
Smlt2272	7.13	36.63	+5.13	15.71	+2.2	flagellar biosynthesis protein FlhF
Smlt2273	5.30	15.54	+2.93	17.22	+3.25	flagellar biosynthesis protein FlhA
Smlt2274	5.73	7.72	+1.35	8.76	+1.53	flagellar biosynthesis protein FlhB

Smlt2275	12.26	8.98	-1.37	10.98	-1.12	CocE/NonD family hydrolase
Smlt2276	12.67	16.05	+1.27	6.38	-1.99	bifunctional diguanylate cyclase/phosphodiesterase
Smlt2277	6.47	8.40	+1.3	9.18	+1.42	flagellar biosynthetic protein FliR
Smlt2278	7.26	25.57	+3.52	18.14	+2.5	flagellar biosynthetic protein FliQ
Smlt2279	9.38	22.61	+2.41	14.19	+1.51	flagellar type III secretion system pore protein FliP
Smlt2280	9.69	19.61	+2.02	11.80	+1.22	flagellar biosynthetic protein FliO
Smlt2281	12.34	45.06	+3.65	24.14	+1.96	flagellar motor switch protein FliN
Smlt2282	7.65	25.74	+3.36	22.14	+2.89	flagellar motor switch protein FliM
Smlt2283	8.91	22.09	+2.48	27.34	+3.07	flagellar basal body-associated FliL family protein
Smlt2284	11.39	47.13	+4.14	15.76	+1.38	flagellar hook-length control protein FliK
Smlt2285	4.35	18.84	+4.33	8.42	+1.94	flagellar export protein FliJ
Smlt2286	9.47	37.15	+3.93	18.10	+1.91	FliI/YscN family ATPase
Smlt2287	17.38	67.64	+3.89	37.59	+2.16	flagellar assembly protein FliH
Smlt2288	31.01	99.21	+3.2	72.95	+2.35	flagellar motor switch protein FliG
Smlt2289	26.92	76.73	+2.85	58.81	+2.18	flagellar M-ring protein FliF
Smlt2290	13.80	25.00	+1.81	27.79	+2.01	flagellar hook-basal body complex protein FliE
Smlt2291	0.00	0.00	0	0.00	0	ISPsy9 like transposase
Smlt2292	0.00	0.00	0	0.00	0	ISPsy9 like transposase for insertion sequence
Smlt2293	0.00	0.00	0	0.00	0	hypothetical protein
Smlt2294	0.00	0.00	0	0.00	0	hypothetical protein
Smlt2295	125.86	165.67	+1.32	468.65	+3.72	sigma-54 dependent transcriptional regulator
Smlt2296	129.25	173.65	+1.34	637.87	+4.94	response regulator transcription factor
Smlt2297	46.63	70.68	+1.52	247.50	+5.31	RNA polymerase factor sigma-54
Smlt2299	68.49	58.35	-1.17	183.31	+2.68	response regulator transcription factor
Smlt2300	113.68	54.74	-2.08	78.51	-1.45	PilZ domain-containing protein
Smlt2301	35.39	11.60	-3.05	27.59	-1.28	hypothetical protein
Smlt2302	212.09	85.39	-2.48	123.05	-1.72	flagellar export chaperone FliS
Smlt2303	232.51	150.85	-1.54	234.68	+1.01	flagellar filament capping protein FliD
Smlt2304	354.01	76.24	-4.64	474.30	+1.34	flagellin

Smlt2305	74.81	27.95	-2.68	167.50	+2.24	flagellin
Smlt2306	114.05	14.10	-8.09	79.30	-1.44	flagellin
Smlt2307	28.77	136.30	+4.74	42.80	+1.49	flagellar hook-associated protein FlgL
Smlt2308	43.49	137.86	+3.17	105.16	+2.42	flagellar hook-associated protein FlgK
Smlt2309	14.39	70.51	+4.9	26.97	+1.87	flagellar assembly peptidoglycan hydrolase FlgJ
Smlt2310	15.91	70.10	+4.4	31.85	+2.	flagellar basal body P-ring protein FlgI
Smlt2311	20.34	85.23	+4.19	49.46	+2.43	flagellar basal body L-ring protein FlgH
Smlt2312	31.08	109.44	+3.52	75.49	+2.43	flagellar basal-body rod protein FlgG
Smlt2313	17.99	65.45	+3.64	30.40	+1.69	flagellar basal body rod protein FlgF
Smlt2314	15.65	68.24	+4.36	30.87	+1.97	flagellar hook protein FlgE
Smlt2315	14.55	40.02	+2.75	23.64	+1.62	flagellar basal body rod modification protein FlgD
Smlt2316	9.05	26.30	+2.9	19.32	+2.13	flagellar basal body rod protein FlgC
Smlt2317	17.71	43.91	+2.48	50.63	+2.86	flagellar basal body rod protein FlgB
Smlt2318	94.54	10.56	-8.95	53.19	-1.78	chemotaxis protein
Smlt2319	63.23	65.72	+1.04	71.35	+1.13	flagellar basal body P-ring formation protein FlgA
Smlt2320	367.12	97.60	-3.76	192.68	-1.91	flagellar biosynthesis anti-sigma factor FlgM
Smlt2321	320.27	84.67	-3.78	142.90	-2.24	flagellar protein FlgN
Smlt2322	90.81	72.61	-1.25	135.45	+1.49	two-component sensor histidine kinase
Smlt2323	70.34	70.81	+1.01	104.36	+1.48	EAL domain-containing protein
Smlt2324	32.45	30.66	-1.06	66.41	+2.05	PAS domain S-box protein
Smlt2325	71.22	11.85	-6.01	63.69	-1.12	GGDEF domain-containing phosphodiesterase
Smlt2326	40.30	30.36	-1.33	35.72	-1.13	hypothetical protein
Smlt2328	0.00	0.00	0	0.00	0	IS110-like element ISStma6 family transposase
Smlt2329	282.73	197.58	-1.43	181.62	-1.56	hypothetical protein
Smlt2330	177.00	29.41	-6.02	125.96	-1.41	methyl-accepting chemotaxis protein
Smlt2331	210.99	163.41	-1.29	187.36	-1.13	high frequency lysogenization protein HflD
Smlt2332	109.83	87.56	-1.25	123.45	+1.12	tRNA 2-thiouridine(34) synthase MnmA
Smlt2333	109.18	75.45	-1.45	108.56	-1.01	NUDIX hydrolase
Smlt2334	235.15	119.23	-1.97	232.95	-1.01	hypothetical protein

Smlt2335	116.44	124.41	+1.07	107.56	-1.08	ATP-dependent Clp protease adapter ClpS
Smlt2336	32.64	60.37	+1.85	28.22	-1.16	APH(6) family putative aminoglycoside O-phosphotransferase
Smlt2337	263.94	611.83	+2.32	246.85	-1.07	ATP-dependent Clp protease ATP-binding subunit ClpA
Smlt2338	7.66	3.03	-2.52	1.66	-4.61	hypothetical protein
Smlt2339	1874.57	792.42	-2.37	686.49	-2.73	translation initiation factor IF-1
Smlt2340	40.60	82.86	+2.04	35.46	-1.14	leucyl/phenylalanyl-tRNA--protein transferase
Smlt2341	27.39	28.98	+1.06	42.83	+1.56	hypothetical protein
Smlt2342	36.86	43.11	+1.17	52.10	+1.41	GNAT family N-acetyltransferase
Smlt2343	132.18	228.52	+1.73	200.79	+1.52	thioredoxin-disulfide reductase
Smlt2344	225.96	189.40	-1.19	239.93	+1.06	DNA translocase FtsK
Smlt2345	164.02	55.59	-2.95	262.11	+1.6	DUF3857 domain-containing protein
Smlt2346	217.69	232.00	+1.07	210.11	-1.04	outer membrane lipoprotein chaperone LolA
Smlt2348	366.07	505.67	+1.38	313.54	-1.17	replication-associated recombination protein A
Smlt2349	11.79	7.47	-1.58	10.62	-1.11	DUF805 domain-containing protein
Smlt2350	0.00	0.00	0	0.00	0	hypothetical protein
Smlt2351	0.00	0.00	0	0.00	0	IS110-like element ISStma6 family transposase
Smlt2352	0.00	0.00	0	0.00	0	hypothetical protein
Smlt2353	15.09	45.28	+3.	1107.43	+73.37	alpha/beta hydrolase
Smlt2354	4.87	179.25	+36.78	2000.26	+410.47	ATP-binding protein
Smlt2355	4.16	201.80	+48.46	1813.75	+435.5	ABC transporter substrate-binding protein
Smlt2356	1.08	58.81	+54.29	330.69	+305.27	iron ABC transporter permease
Smlt2357	2.18	64.20	+29.49	610.07	+280.26	ABC transporter ATP-binding protein
Smlt2358	153.91	151.30	-1.02	739.09	+4.8	hypothetical protein
Smlt2360	0.00	0.00	0	0.00	0	IS481-like element ISStma1 family transposase
Smlt2361	23.76	37.04	+1.56	27.65	+1.16	elongation factor G
Smlt2362	105.22	45.41	-2.32	39.59	-2.66	hypothetical protein
Smlt2364	168.74	144.82	-1.17	287.39	+1.7	SapC family protein
Smlt2365	0.00	0.00	0	0.00	0	response regulator
Smlt2366	0.00	0.00	0	0.00	0	response regulator transcription factor

Smlt2367	5.51	15.42	+2.8	32.55	+5.91	lipocalin family protein
Smlt2368	0.00	0.00	0	0.00	0	cyclopropane-fatty-acyl-phospholipid synthase
Smlt2369	1.73	3.07	+1.78	9.22	+5.34	DUF1295 domain-containing protein
Smlt2370	1.24	2.89	+2.33	5.31	+4.27	DUF2878 domain-containing protein
Smlt2371	2.15	6.01	+2.79	17.11	+7.94	cyclopropane-fatty-acyl-phospholipid synthase family protein
Smlt2372	1.96	2.06	+1.05	11.73	+5.98	DUF1365 domain-containing protein
Smlt2373	3.48	8.20	+2.36	18.18	+5.22	FAD-dependent oxidoreductase
Smlt2374	2.62	7.52	+2.88	12.70	+4.86	acyl-CoA desaturase
Smlt2375	0.98	2.06	+2.1	3.66	+3.73	hypothetical protein
Smlt2377	49.66	39.86	-1.25	92.77	+1.87	sigma-70 family RNA polymerase sigma factor
Smlt2378	32.39	32.78	+1.01	74.94	+2.31	ChrR family anti-sigma-E factor
Smlt2379	79.31	88.84	+1.12	159.12	+2.01	hypothetical protein
Smlt2380	64.97	90.61	+1.39	120.72	+1.86	hybrid sensor histidine kinase/response regulator
Smlt2381	124.85	126.39	+1.01	185.54	+1.49	response regulator
Smlt2382	86.95	97.87	+1.13	146.01	+1.68	GAF domain-containing protein
Smlt2383	54.62	112.34	+2.06	88.50	+1.62	hypothetical protein
Smlt2384	10.55	30.30	+2.87	32.07	+3.04	tryptophan-rich sensory protein
Smlt2385	19.60	40.99	+2.09	28.64	+1.46	hypothetical protein
Smlt2386	0.00	0.00	0	0.00	0	GNAT family N-acetyltransferase
Smlt2387	0.00	0.00	0	0.00	0	hypothetical protein
Smlt2388	0.00	0.00	0	0.00	0	transmembrane protein
Smlt2389	0.00	0.00	0	0.00	0	hypothetical protein
Smlt2390	0.00	0.00	0	0.00	0	hypothetical protein
Smlt2391	0.00	0.00	0	0.00	0	transmembrane protein
Smlt2392	0.00	0.00	0	0.00	0	adhesin
Smlt2393	0.00	0.00	0	0.00	0	DNA-binding protein
Smlt2394	0.00	0.00	0	0.00	0	transmembrane protein
Smlt2395	0.00	0.00	0	0.00	0	hypothetical protein
Smlt2397	0.00	0.00	0	0.00	0	hypothetical protein

Smlt2398	0.00	0.00	0	0.00	0	DUF2523 domain-containing protein
Smlt2399	0.00	0.00	0	0.00	0	hypothetical protein
Smlt2400	0.00	0.00	0	0.00	0	DNA-binding protein
Smlt2401	0.00	0.00	0	0.00	0	hypothetical protein
Smlt2403	0.00	0.00	0	0.00	0	hypothetical protein
Smlt2404	0.00	0.00	0	0.00	0	hypothetical protein
Smlt2405	0.00	0.00	0	0.00	0	cysteine hydrolase
Smlt2406	0.00	0.00	0	0.00	0	hypothetical protein
Smlt2407	0.00	0.00	0	0.00	0	hypothetical protein
Smlt2407A	0.00	0.00	0	0.00	0	DNA-binding protein
Smlt2408	0.00	0.00	0	0.00	0	transposase
Smlt2409	0.00	0.00	0	0.00	0	Hg(II)-responsive transcriptional regulator
Smlt2410	0.00	0.00	0	0.00	0	mercuric ion transporter MerT
Smlt2411	0.00	0.00	0	0.00	0	mercury resistance system periplasmic binding protein MerP
Smlt2412	0.00	0.00	0	0.00	0	mercury(II) reductase
Smlt2413	0.00	0.00	0	0.00	0	ISL3-like element ISStma11 family transposase
Smlt2414	0.00	0.00	0	0.00	0	ISXac3 like transposase for insertion sequence element
Smlt2416	0.00	0.00	0	0.00	0	insertion sequence protein
Smlt2417	0.00	0.00	0	0.00	0	IS3-like element ISStma13 family transposase
Smlt2418	0.00	0.00	0	0.00	0	NAD(P)/FAD-dependent oxidoreductase
Smlt2419	0.00	0.00	0	0.00	0	metalloregulator ArsR/SmtB family transcription factor
Smlt2420	0.00	0.00	0	0.00	0	MFS transporter
Smlt2421	0.00	0.00	0	0.00	0	arsenate reductase (glutaredoxin)
Smlt2422	0.00	0.00	0	0.00	0	arsenical resistance protein ArsH
Smlt2423	0.00	0.00	0	0.00	0	metalloregulator ArsR/SmtB family transcription factor
Smlt2424	0.00	0.00	0	0.00	0	arsenate reductase ArsC
Smlt2425	0.00	0.00	0	0.00	0	ACR3 family arsenite efflux transporter
Smlt2426	0.00	0.00	0	0.00	0	thioredoxin-disulfide reductase

Smlt2427	0.00	0.00	0	0.00	0	ArsO family NAD(P)H-dependent flavin-containing monooxygenase
Smlt2428	0.00	0.00	0	0.00	0	helix-turn-helix domain-containing protein
Smlt2431	0.00	0.00	0	0.00	0	hypothetical protein
Smlt2432	0.00	0.00	0	0.00	0	TolC family protein
Smlt2433	0.00	0.00	0	0.00	0	efflux RND transporter periplasmic adaptor subunit
Smlt2434	0.00	0.00	0	0.00	0	CusA/CzcA family heavy metal efflux RND transporter
Smlt2435	0.00	0.00	0	0.00	0	DUF4198 domain-containing protein
Smlt2436	0.00	0.00	0	0.00	0	TonB-dependent outer membrane receptor
Smlt2437	0.00	0.00	0	0.00	0	arginase family protein
Smlt2438	0.00	0.00	0	0.00	0	DUF305 domain-containing protein
Smlt2439	0.00	0.00	0	0.00	0	hypothetical protein
Smlt2440	0.00	0.00	0	0.00	0	copper-translocating P-type ATPase
Smlt2441	0.00	0.00	0	0.00	0	copper homeostasis membrane protein CopD
Smlt2442	0.00	0.00	0	0.00	0	copper homeostasis periplasmic binding protein CopC
Smlt2443	0.00	0.00	0	0.00	0	four-helix bundle copper-binding protein
Smlt2444	0.00	0.00	0	0.00	0	DUF411 domain-containing protein
Smlt2445	0.00	0.00	0	0.00	0	nuclear transport factor 2 family protein
Smlt2446	0.00	0.00	0	0.00	0	cytochrome c
Smlt2447	0.00	0.00	0	0.00	0	copper-binding protein CopB
Smlt2448	0.00	0.00	0	0.00	0	copper resistance system multicopper oxidase
Smlt2449	0.00	0.00	0	0.00	0	transcriptional regulator CopL
Smlt2450	0.00	0.00	0	0.00	0	helix-turn-helix domain-containing protein
Smlt2451	0.00	0.00	0	0.00	0	conserved hypothetical protein
Smlt2452	0.00	0.00	0	0.00	0	hypothetical protein
Smlt2453	0.00	0.00	0	0.00	0	DNA-binding protein
Smlt2454	0.00	0.00	0	0.00	0	AAA family ATPase
Smlt2455	0.00	0.00	0	0.00	0	AAA family ATPase
Smlt2456	0.00	0.00	0	0.00	0	hypothetical protein

Smlt2457	0.00	0.00	0	0.00	0	CusA/CzcA family heavy metal efflux RND transporter
Smlt2458	0.00	0.00	0	0.00	0	efflux RND transporter periplasmic adaptor subunit
Smlt2459	0.00	0.00	0	0.00	0	TolC family protein
Smlt2460	0.00	0.00	0	0.00	0	hypothetical protein
Smlt2461	0.00	0.00	0	0.00	0	cation transporter
Smlt2462	0.00	0.00	0	0.00	0	FTR1 family protein
Smlt2463	0.00	0.00	0	0.00	0	helix-turn-helix domain-containing protein
Smlt2464	0.00	0.00	0	0.00	0	site-specific integrase
Smlt2465	0.00	0.00	0	0.00	0	Tn3 family transposase
Smlt2466	0.00	0.00	0	0.00	0	hypothetical protein
Smlt2467	0.00	0.00	0	0.00	0	hypothetical protein
Smlt2468	0.00	0.00	0	0.00	0	DUF4209 domain-containing protein
Smlt2469	0.00	0.00	0	0.00	0	hypothetical protein
Smlt2470	0.00	0.00	0	0.00	0	hypothetical protein
Smlt2471	0.00	0.00	0	0.00	0	DNA-binding protein
Smlt2472	0.00	0.00	0	0.00	0	site-specific integrase
Smlt2474	0.00	0.00	0	0.00	0	hypothetical protein
Smlt2475	0.00	0.00	0	0.00	0	WYL domain-containing protein
Smlt2476	0.00	0.00	0	0.00	0	DUF1508 domain-containing protein
Smlt2477	0.00	0.00	0	0.00	0	hypothetical protein
Smlt2478	0.00	0.00	0	0.00	0	IS3-like element ISStma5 family transposase
Smlt2479	0.00	0.00	0	0.00	0	ISXac3 like transposase
Smlt2479A	0.00	0.00	0	0.00	0	conserved hypothetical protein
Smlt2481	0.00	0.00	0	0.00	0	ATP-binding protein
Smlt2482	0.00	0.00	0	0.00	0	hypothetical protein
Smlt2482A	0.00	0.00	0	0.00	0	conserved hypothetical protein
Smlt2482B	0.00	0.00	0	0.00	0	phage-related protein
Smlt2486	0.00	0.00	0	0.00	0	phage-related protein, similar to zonula occudens toxin
Smlt2487	0.00	0.00	0	0.00	0	integrase

Smlt2488	0.00	0.00	0	0.00	0	TM0106 family RecB-like putative nuclease
Smlt2489	0.00	0.00	0	0.00	0	IS3-like element ISStma9 family transposase
Smlt2490	0.00	0.00	0	0.00	0	ISXac3 like transposase family protein
Smlt2491	0.00	0.00	0	0.00	0	ATP-binding protein
Smlt2492	0.00	0.00	0	0.00	0	conserved hypothetical protein
Smlt2493	0.00	0.00	0	0.00	0	hypothetical protein
Smlt2494	0.00	0.00	0	0.00	0	hypothetical protein
Smlt2495	0.00	0.00	0	0.00	0	hypothetical protein
Smlt2496	0.00	0.00	0	0.00	0	hypothetical protein
Smlt2497	22.04	4.07	-5.41	11.50	-1.92	tetratricopeptide repeat protein
Smlt2498	22.69	40.25	+1.77	27.98	+1.23	hypothetical protein
Smlt2499	0.00	0.00	0	0.00	0	conserved hypothetical protein
Smlt2500	0.00	0.00	0	0.00	0	hypothetical protein
Smlt2501	0.00	0.00	0	0.00	0	nucleotidyltransferase family protein
Smlt2502	0.00	0.00	0	0.00	0	XdhC family protein
Smlt2503	86.37	95.98	+1.11	8.99	-9.61	xanthine dehydrogenase family protein molybdopterin-binding subunit
Smlt2504	54.47	56.34	+1.03	4.87	-11.17	xanthine dehydrogenase family protein subunit M
Smlt2505	137.18	210.42	+1.53	42.52	-3.23	aldehyde dehydrogenase iron-sulfur subunit
Smlt2506	0.00	0.00	0	0.00	0	hypothetical protein
Smlt2507	0.00	0.00	0	0.00	0	Ohr family peroxiredoxin
Smlt2508	0.00	0.00	0	0.00	0	SDR family oxidoreductase
Smlt2509	0.00	0.00	0	0.00	0	LysR family transcriptional regulator
Smlt2510	0.00	0.00	0	0.00	0	transcriptional regulator
Smlt2511	1.88	0.87	-2.18	0.78	-2.4	carboxymuconolactone decarboxylase family protein
Smlt2512	0.00	0.00	0	0.00	0	cupin domain-containing protein
Smlt2513	0.00	0.00	0	0.00	0	RNA polymerase sigma-70 factor
Smlt2514	11.05	15.90	+1.44	8.34	-1.33	MBL fold metallo-hydrolase
Smlt2516	4.50	5.41	+1.2	8.96	+1.99	alpha/beta hydrolase

Smlt2518	0.00	0.00	0	0.00	0	hypothetical protein
Smlt2519	0.00	0.00	0	0.00	0	hypothetical protein
Smlt2521	0.00	0.00	0	0.00	0	hypothetical protein
Smlt2522	0.00	0.00	0	0.00	0	hypothetical protein
Smlt2523	0.00	0.00	0	0.00	0	excinuclease ABC subunit UvrA
Smlt2524	0.00	0.00	0	0.00	0	hypothetical protein
Smlt2525	26.91	23.82	-1.13	45.33	+1.68	hypothetical protein
Smlt2526	18.54	17.50	-1.06	20.34	+1.1	low affinity iron permease family protein
Smlt2527	6.69	7.76	+1.16	5.60	-1.19	FAD-dependent oxidoreductase
Smlt2528	67.52	87.45	+1.3	47.00	-1.44	BLUF domain-containing protein
Smlt2529	46.80	66.62	+1.42	45.62	-1.03	hypothetical protein
Smlt2530	2.72	5.64	+2.07	5.51	+2.03	DNA ligase D
Smlt2531	0.85	3.29	+3.86	7.53	+8.84	DUF3606 domain-containing protein
Smlt2532	14.69	13.36	-1.1	46.08	+3.14	DUF892 family protein
Smlt2533	1.44	3.40	+2.36	3.71	+2.58	endonuclease
Smlt2534	24.74	49.29	+1.99	20.56	-1.2	DUF72 domain-containing protein
Smlt2535	7.25	11.38	+1.57	14.61	+2.02	BLUF domain-containing protein
Smlt2536	21.18	13.87	-1.53	41.94	+1.98	hypothetical protein
Smlt2537	6.80	8.15	+1.2	8.70	+1.28	manganese catalase family protein
Smlt2538	8.52	10.04	+1.18	13.10	+1.54	ferritin-like domain-containing protein
Smlt2540	14.39	17.57	+1.22	21.31	+1.48	response regulator
Smlt2541	2.46	2.09	-1.18	1.60	-1.54	BLUF domain-containing protein
Smlt2543	2.96	0.85	-3.5	5.99	+2.02	glycosyltransferase
Smlt2544	3.33	5.23	+1.57	10.20	+3.06	methyltransferase domain-containing protein
Smlt2545	2.09	2.19	+1.05	4.73	+2.27	PIG-L family deacetylase
Smlt2546	2.96	5.12	+1.73	9.04	+3.05	hypothetical protein
Smlt2547	3.89	3.63	-1.07	3.45	-1.13	hypothetical protein
Smlt2548	18.98	20.23	+1.07	15.33	-1.24	glutathione-dependent formaldehyde dehydrogenase
Smlt2549	7.58	12.77	+1.68	17.94	+2.37	hypothetical protein

Smlt2550	0.00	0.00	0	0.00	0	transmembrane protein
Smlt2551	0.00	0.00	0	0.00	0	transmembrane protein
Smlt2552	1.65	5.18	+3.14	3.51	+2.13	cysteine hydrolase
Smlt2553	4.19	11.38	+2.72	5.57	+1.33	hypothetical protein
Smlt2554	10.79	20.29	+1.88	11.08	+1.03	hypothetical protein
Smlt2555	57.39	115.83	+2.02	53.77	-1.07	LacI family transcriptional regulator
Smlt2556	23.66	83.72	+3.54	15.20	-1.56	phosphoenolpyruvate--protein phosphotransferase
Smlt2557	18.83	74.50	+3.96	14.59	-1.29	1-phosphofructokinase family hexose kinase
Smlt2558	9.65	39.20	+4.06	4.85	-1.99	fructose-specific PTS transporter subunit EIIC
Smlt2559	7.58	35.36	+4.67	4.07	-1.86	carbohydrate porin
Smlt2560	17.19	15.92	-1.08	19.38	+1.13	aromatic alcohol reductase
Smlt2561	29.32	35.77	+1.22	42.62	+1.45	helix-turn-helix transcriptional regulator
Smlt2562	20.11	32.86	+1.63	28.83	+1.43	fasciclin domain-containing protein
Smlt2563	8.90	12.90	+1.45	15.57	+1.75	serine hydrolase
Smlt2566	72.40	67.38	-1.07	98.03	+1.35	TonB-dependent outer membrane receptor
Smlt2567	18.84	22.66	+1.2	17.72	-1.06	MFS transporter
Smlt2568	109.96	123.16	+1.12	191.77	+1.74	LacI family DNA-binding transcriptional regulator
Smlt2569	272.41	252.10	-1.08	301.35	+1.11	glycoside hydrolase family 3 protein
Smlt2570	36.49	31.53	-1.16	42.09	+1.15	arsenic transporter
Smlt2571	1.26	2.67	+2.11	0.99	-1.27	phosphate porin
Smlt2572	9.70	11.46	+1.18	8.76	-1.11	ABC transporter permease
Smlt2573	6.87	13.77	+2.	9.17	+1.33	ABC transporter ATP-binding protein
Smlt2574	12.32	17.52	+1.42	16.47	+1.34	ABC transporter substrate-binding protein
Smlt2575	4.97	6.15	+1.24	9.40	+1.89	4-oxalomesaconate tautomerase
Smlt2576	22.79	127.51	+5.59	30.19	+1.32	LysR family transcriptional regulator
Smlt2577	25.79	28.61	+1.11	27.63	+1.07	excinuclease ABC subunit UvrA
Smlt2578	0.00	0.00	0	0.00	0	hypothetical protein
Smlt2579	110.13	342.98	+3.11	124.41	+1.13	BLUF domain-containing protein
Smlt2580	9.50	11.84	+1.25	10.60	+1.12	hypothetical protein

Smlt2581	0.00	0.00	0	0.00	0	hypothetical protein
Smlt2582	0.00	0.00	0	0.00	0	methylenetetrahydrofolate reductase
Smlt2583	51.58	245.94	+4.77	28.40	-1.82	LysR family transcriptional regulator
Smlt2584	2.18	6.37	+2.92	1.53	-1.43	FMN reductase
Smlt2585	2.24	7.49	+3.35	3.44	+1.54	DUF1852 domain-containing protein
Smlt2586	10.56	28.94	+2.74	8.79	-1.2	methionine synthase
Smlt2587	31.61	47.00	+1.49	18.15	-1.74	hypothetical protein
Smlt2588	52.40	49.53	-1.06	58.20	+1.11	trypsin-like serine protease
Smlt2589	7.41	5.86	-1.26	10.57	+1.42	serine hydrolase
Smlt2590	4.65	8.86	+1.9	5.16	+1.11	PHB depolymerase family esterase
Smlt2591	5.07	7.91	+1.56	11.65	+2.3	TonB-dependentouter membrane receptor
Smlt2592	48.03	57.73	+1.2	64.18	+1.34	alpha/beta hydrolase
Smlt2593	19.44	19.88	+1.02	24.54	+1.26	MaoC family dehydratase
Smlt2594	9.42	8.87	-1.06	21.31	+2.26	PAS-domain containing protein
Smlt2595	75.62	82.21	+1.09	112.67	+1.49	response regulator transcription factor
Smlt2596	8.47	18.94	+2.24	14.99	+1.77	fatty acid--CoA ligase
Smlt2597	33.45	39.20	+1.17	25.20	-1.33	NAD(P)H-dependent oxidoreductase
Smlt2598	55.94	35.60	-1.57	40.81	-1.37	hypothetical protein
Smlt2599	212.81	292.52	+1.37	121.73	-1.75	FadR family transcriptional regulator
Smlt2600	6.05	9.06	+1.5	7.67	+1.27	TonB-dependent outer membrane receptor
Smlt2601	9.42	24.57	+2.61	14.51	+1.54	polysaccharide lyase 6 family protein
Smlt2602	11.57	33.85	+2.93	18.78	+1.62	alginate lyase family protein
Smlt2603	3.24	11.03	+3.41	5.97	+1.85	MFS transporter
Smlt2604	6.41	24.97	+3.9	31.09	+4.85	glucose 1-dehydrogenase
Smlt2605	7.36	22.48	+3.05	29.29	+3.98	GDSL-type esterase/lipase family protein
Smlt2606	7.61	20.06	+2.64	22.81	+3.	sugar kinase
Smlt2607	43.64	88.30	+2.02	76.11	+1.74	hypothetical protein
Smlt2608	8.31	23.61	+2.84	5.33	-1.56	hypothetical protein
Smlt2609	14.31	38.14	+2.66	16.89	+1.18	alpha/beta fold hydrolase

Smlt2610	0.00	0.00	0	0.00	0	conserved hypothetical protein
Smlt2611	0.51	0.34	-1.51	0.59	+1.15	DNA/RNA non-specific endonuclease
Smlt2612	101.07	66.10	-1.53	98.45	-1.03	HAD family phosphatase
Smlt2613	26.76	28.45	+1.06	32.61	+1.22	hypothetical protein
Smlt2614	27.61	27.34	-1.01	43.12	+1.56	VOC family protein
Smlt2615	61.69	39.44	-1.56	76.01	+1.23	AraC family transcriptional regulator
Smlt2616	73.20	67.85	-1.08	94.55	+1.29	LysR family transcriptional regulator
Smlt2617	2.10	2.48	+1.18	2.23	+1.06	twin-arginine translocation signal domain-containing protein
Smlt2618	0.00	0.00	0	0.00	0	PD40 domain-containing protein
Smlt2619	1.88	1.93	+1.02	2.32	+1.24	hypothetical protein
Smlt2620	2.68	2.96	+1.1	1.13	-2.39	hypothetical protein
Smlt2621	12.23	9.82	-1.25	6.47	-1.89	sigma-54 dependent transcriptional regulator
Smlt2622	0.57	1.57	+2.76	0.22	-2.6	hypothetical protein
Smlt2623	2.20	1.35	-1.63	1.08	-2.04	hypothetical protein
Smlt2624	0.00	0.00	0	0.00	0	hypothetical protein
Smlt2625	0.96	1.89	+1.96	1.44	+1.49	C39 family peptidase
Smlt2626	0.00	0.00	0	0.00	0	hypothetical protein
Smlt2627	0.93	0.84	-1.12	0.52	-1.81	transporter
Smlt2628	194.24	264.83	+1.36	186.42	-1.04	SPFH domain-containing protein
Smlt2629	76.45	55.71	-1.37	65.48	-1.17	membrane protein
Smlt2630	94.75	102.64	+1.08	101.24	+1.07	hypothetical protein
Smlt2631	0.00	0.00	0	0.00	0	AprI/Inh family metalloprotease inhibitor
Smlt2632	6.05	6.50	+1.07	4.41	-1.37	cation diffusion facilitator family transporter
Smlt2633	10.91	14.91	+1.37	13.03	+1.19	DNA-3-methyladenine glycosylase
Smlt2634	3.09	5.12	+1.66	5.50	+1.78	hypothetical protein
Smlt2635	8.49	8.08	-1.05	11.45	+1.35	PAS domain S-box protein
Smlt2636	7.52	9.03	+1.2	8.71	+1.16	exodeoxyribonuclease III
Smlt2637	3.09	3.88	+1.26	2.65	-1.17	trehalose synthase
Smlt2638	3.51	5.10	+1.45	6.46	+1.84	TIGR03885 family FMN-dependent LLM class oxidoreductase

Smlt2639	0.39	2.60	+6.62	1.28	+3.27	sensory rhodopsin transducer
Smlt2640	1.48	2.21	+1.49	5.38	+3.64	glucose 1-dehydrogenase
Smlt2641	6.60	7.98	+1.21	6.39	-1.03	mechanosensitive ion channel
Smlt2642	13.19	57.86	+4.39	12.49	-1.06	SbiA
Smlt2643	13.91	73.90	+5.31	13.24	-1.05	SbiB
Smlt2644	15.08	33.99	+2.25	20.37	+1.35	hypothetical protein
Smlt2645	35.12	78.44	+2.23	49.14	+1.4	response regulator
Smlt2646	65.88	152.64	+2.32	70.13	+1.06	HAMP domain-containing protein
Smlt2647	14.56	22.44	+1.54	9.10	-1.6	ABC transporter six-transmembrane domain-containing protein
Smlt2648	128.10	130.06	+1.02	125.86	-1.02	winged helix DNA-binding domain-containing protein
Smlt2649	78.36	174.35	+2.22	107.72	+1.37	TetR/AcrR family transcriptional regulator
Smlt2650	0.00	0.00	0	0.00	0	TonB-dependent outer membrane receptor
Smlt2652	31.58	13.49	-2.34	38.98	+1.23	conjugal transfer protein TrbP
Smlt2653	38.02	23.26	-1.63	45.84	+1.21	prolyl aminopeptidase
Smlt2654	2.76	3.61	+1.3	2.80	+1.01	hypothetical protein
Smlt2655	2.68	4.46	+1.67	2.37	-1.13	magnesium-translocating P-type ATPase
Smlt2656	12.46	15.11	+1.21	5.38	-2.32	MgtC/SapB family protein
Smlt2657	21.49	19.14	-1.12	43.68	+2.03	two-component sensor histidine kinase
Smlt2658	20.90	21.90	+1.05	36.46	+1.74	response regulator transcription factor
Smlt2659	0.00	0.00	0	0.00	0	DUF1697 domain-containing protein
Smlt2660	0.00	0.00	0	0.00	0	DUF3224 domain-containing protein
Smlt2661	0.00	0.00	0	0.00	0	EamA family transporter
Smlt2662	9.26	13.05	+1.41	16.25	+1.76	low temperature requirement protein A
Smlt2663	4.52	7.79	+1.72	35.05	+7.76	hypothetical protein
Smlt2664	4.74	16.96	+3.58	120.75	+25.48	PacI, FecI-like RNA polymerase sigma factor
Smlt2665	4.87	20.90	+4.29	204.34	+41.98	PacR, FecR-like protein
Smlt2666	2.51	22.94	+9.13	114.16	+45.43	PacA, FecA-like TonB-dependent outer membrane receptor
Smlt2667	10.80	24.59	+2.28	164.51	+15.24	L1 family subclass B3 metallo-beta-lactamase
Smlt2668	24.01	26.80	+1.12	13.70	-1.75	DUF411 domain-containing protein

Smlt2669	0.00	0.00	0	0.00	0	transmembrane anchor protein
Smlt2670	40.71	40.68	-1.	47.39	+1.16	DUF4034 domain-containing protein
Smlt2671	35.77	38.08	+1.06	48.66	+1.36	FdhF/YdeP family oxidoreductase
Smlt2672	33.84	62.20	+1.84	66.09	+1.95	formate dehydrogenase accessory sulfurtransferase FdhD
Smlt2675	5.05	4.03	-1.25	3.35	-1.51	carboxymuconolactone decarboxylase family protein
Smlt2676	0.00	0.00	0	0.00	0	AraC family transcriptional regulator
Smlt2677	214.97	529.87	+2.46	172.80	-1.24	TetR/AcrR family transcriptional regulator
Smlt2678	2.07	10.80	+5.23	1.51	-1.37	MFS transporter
Smlt2679	0.00	0.00	0	0.00	0	hypothetical protein
Smlt2680	1.34	1.74	+1.3	1.68	+1.26	DUF3299 domain-containing protein
Smlt2681	3.69	2.71	-1.36	3.67	-1.	DUF3299 domain-containing protein
Smlt2682	1.50	2.59	+1.72	1.27	-1.18	ABC transporter permease
Smlt2683	1.73	2.27	+1.31	1.50	-1.16	ATP-binding cassette domain-containing protein
Smlt2684	2.12	2.03	-1.05	1.79	-1.18	DUF2796 domain-containing protein
Smlt2685	0.00	0.00	0	0.00	0	MerC domain-containing protein
Smlt2686	14.86	29.39	+1.98	14.18	-1.05	LysR family transcriptional regulator
Smlt2687	1.31	2.07	+1.58	1.55	+1.18	2,5-didehydrogluconate reductase DkgB
Smlt2688	1.43	1.58	+1.1	1.19	-1.2	TIGR03571 family LLM class oxidoreductase
Smlt2689	1.61	0.80	-2.01	1.11	-1.45	cyclase family protein
Smlt2690	6.75	9.24	+1.37	4.56	-1.48	hypothetical protein
Smlt2691	0.00	0.00	0	0.00	0	copper homeostasis periplasmic binding protein CopC
Smlt2692	0.00	0.00	0	0.00	0	copper homeostasis membrane protein CopD
Smlt2693	0.00	0.00	0	0.00	0	heavy metal response regulator transcription factor
Smlt2694	0.00	0.00	0	0.00	0	heavy metal sensor histidine kinase
Smlt2695	53.77	82.09	+1.53	27.21	-1.98	LysR family transcriptional regulator
Smlt2696	3.34	4.36	+1.3	2.14	-1.56	SDR family oxidoreductase
Smlt2697	2.18	4.03	+1.85	2.66	+1.22	CusA/CzcA family heavy metal efflux RND transporter
Smlt2698	0.77	1.91	+2.48	1.88	+2.43	efflux RND transporter periplasmic adaptor subunit
Smlt2699	1.51	2.47	+1.63	2.53	+1.68	TolC family protein

Smlt2700	42.22	45.85	+1.09	55.89	+1.32	response regulator
Smlt2701	29.00	42.59	+1.47	31.59	+1.09	sensor histidine kinase
Smlt2702	250.61	284.42	+1.13	458.30	+1.83	GH92 family glycosyl hydrolase
Smlt2703	0.00	0.00	0	0.00	0	thioredoxin family protein
Smlt2704	79.92	165.59	+2.07	291.75	+3.65	host attachment protein
Smlt2706	74.33	87.50	+1.18	54.29	-1.37	glycine betaine/L-proline transporter ProP
Smlt2707	58.36	81.03	+1.39	82.87	+1.42	hypothetical protein
Smlt2708	42.09	27.73	-1.52	22.09	-1.91	hypothetical protein
Smlt2709	100.31	148.63	+1.48	113.52	+1.13	type II toxin-antitoxin system Phd/YefM family antitoxin
Smlt2710	82.86	115.93	+1.4	170.75	+2.06	PAS domain-containing protein
Smlt2711	128.45	261.29	+2.03	529.82	+4.12	response regulator
Smlt2712	2.59	62.09	+23.97	802.47	+309.72	outer membrane protein
Smlt2713	4.83	525.04	+108.72	9564.36	+1980.49	extracellular protein
Smlt2714	5.38	254.29	+47.23	3390.63	+629.8	FecA-like TonB-dependent outer membrqne receptor
Smlt2715	11.34	59.90	+5.28	310.74	+27.39	FecR-like protein
Smlt2716	17.05	25.09	+1.47	322.90	+18.93	FecI-like RNA polymerase sigma factor
Smlt2718	56.45	105.45	+1.87	61.57	+1.09	LysR family transcriptional regulator
Smlt2719	3.57	4.51	+1.26	4.11	+1.15	pirin family protein
Smlt2720	4.87	6.84	+1.4	13.40	+2.75	hydrolase
Smlt2721	18.32	16.33	-1.12	28.93	+1.58	hydrolase
Smlt2722	115.83	109.46	-1.06	119.16	+1.03	OsmC family protein
Smlt2723	0.69	0.76	+1.1	1.14	+1.67	hypothetical protein
Smlt2724	2.55	4.57	+1.8	3.65	+1.43	putative porin
Smlt2725	2.83	4.62	+1.63	2.61	-1.08	TonB family protein
Smlt2726	10.74	14.27	+1.33	8.33	-1.29	biopolymer transporter ExbD
Smlt2727	1.61	3.73	+2.32	2.24	+1.4	MotA/TolQ/ExbB proton channel family protein
Smlt2728	1.40	2.52	+1.8	1.31	-1.07	BamA/TamA family outer membrane protein
Smlt2729	2.02	4.70	+2.33	2.59	+1.28	hypothetical protein
Smlt2730	3.10	3.78	+1.22	2.38	-1.3	general secretion pathway protein

Smlt2731	0.00	0.00	0	0.00	0	type II secretion system minor pseudopilin GspH
Smlt2732	0.64	1.19	+1.84	1.07	+1.67	type II secretion system minor pseudopilin GspI
Smlt2733	6.62	7.44	+1.12	3.20	-2.07	prepilin-type N-terminal cleavage/methylation domain-containing protein
Smlt2735	0.00	0.00	0	0.00	0	IS110-like element ISStma6 family transposase
Smlt2737	0.00	0.00	0	0.00	0	filamentous haemagglutinin family protein
Smlt2738	0.00	0.00	0	0.00	0	substrate-binding domain-containing protein
Smlt2740	1.26	1.53	+1.21	0.80	-1.58	type II secretion system inner membrane protein GspF
Smlt2741	3.10	2.96	-1.05	3.03	-1.02	type II secretion system ATPase GspE
Smlt2742	1.90	2.79	+1.47	2.51	+1.32	type II secretion system secretin GspD
Smlt2743	0.86	0.79	-1.09	1.43	+1.67	type II secretion system protein M
Smlt2744	2.33	2.86	+1.23	2.61	+1.12	general secretion pathway protein GspL
Smlt2745	1.00	1.93	+1.93	0.93	-1.08	type II secretion system minor pseudopilin GspK
Smlt2746	3.79	8.74	+2.31	4.56	+1.21	type II secretion system major pseudopilin GspG
Smlt2747	0.64	1.28	+1.99	0.94	+1.46	hypothetical protein
Smlt2748	1.64	1.05	-1.55	1.00	-1.64	hypothetical protein
Smlt2749	5.23	7.38	+1.41	9.78	+1.87	DUF4880 domain-containing protein
Smlt2750	1.56	3.29	+2.11	1.15	-1.36	sigma-70 family RNA polymerase sigma factor
Smlt2751	3.35	1.58	-2.11	1.06	-3.17	TonB-dependent outer membrane receptor
Smlt2753	59.34	56.00	-1.06	53.21	-1.12	Lrp/AsnC family transcriptional regulator
Smlt2754	9.93	5.02	-1.98	7.55	-1.31	AzlC family ABC transporter permease
Smlt2755	11.49	9.51	-1.21	8.65	-1.33	AzlD family protein
Smlt2756	7.86	6.71	-1.17	34.07	+4.34	glycogen debranching protein GlgX
Smlt2757	4.77	5.69	+1.19	26.71	+5.59	malto-oligosyltrehalose synthase
Smlt2758	3.64	5.46	+1.5	18.75	+5.16	4-alpha-glucanotransferase
Smlt2759	7.32	8.47	+1.16	29.38	+4.01	malto-oligosyltrehalose trehalohydrolase
Smlt2760	10.09	12.66	+1.25	33.59	+3.33	1,4-alpha-glucan branching enzyme
Smlt2761	10.52	9.47	-1.11	16.15	+1.54	glycogen synthase GlgA
Smlt2762	8.61	1.16	-7.4	2.63	-3.27	PAS sensor domain-containing protein

Smlt2763	4.73	1.30	-3.63	1.86	-2.55	sulfite reductase flavoprotein subunit alpha
Smlt2764	18.71	23.25	+1.24	25.73	+1.38	ATP-binding cassette domain-containing protein
Smlt2765	40.78	63.25	+1.55	59.89	+1.47	molybdate ABC transporter permease subunit
Smlt2766	97.79	142.01	+1.45	165.35	+1.69	molybdate ABC transporter substrate-binding protein
Smlt2767	0.00	0.00	0	0.00	0	helix-turn-helix domain-containing protein
Smlt2768	0.00	0.00	0	0.00	0	coproporphyrinogen III oxidase
Smlt2769	0.00	0.00	0	0.00	0	NarK/NasA family nitrate transporter
Smlt2770	0.00	0.00	0	0.00	0	peptidylprolyl isomerase
Smlt2771	0.00	0.00	0	0.00	0	respiratory nitrate reductase subunit gamma
Smlt2772	0.00	0.00	0	0.00	0	nitrate reductase molybdenum cofactor assembly chaperone
Smlt2773	0.00	0.00	0	0.00	0	nitrate reductase subunit beta
Smlt2774	0.00	0.00	0	0.00	0	nitrate reductase subunit alpha
Smlt2775	0.00	0.00	0	0.00	0	NarK family nitrate/nitrite MFS transporter
Smlt2776	0.00	0.00	0	0.00	0	hypothetical protein
Smlt2777	0.00	0.00	0	0.00	0	molybdenum cofactor guanylyltransferase
Smlt2778	169.00	352.70	+2.09	251.91	+1.49	molybdenum cofactor biosynthesis protein MoaE
Smlt2779	46.51	83.51	+1.8	49.62	+1.07	MoaD/ThiS family protein
Smlt2780	0.00	0.00	0	0.00	0	molybdopterin molybdotransferase MoeA
Smlt2781	74.26	114.59	+1.54	104.35	+1.41	bifunctional molybdenum cofactor biosynthesis protein MoaC/MoaB
Smlt2782	103.45	166.05	+1.61	145.01	+1.4	GTP 3',8-cyclase MoaA
Smlt2783	0.00	0.00	0	0.00	0	hypothetical protein
Smlt2784	0.00	0.00	0	0.00	0	hypothetical protein
Smlt2785	3.22	7.10	+2.21	2.47	-1.3	hypothetical protein
Smlt2786	65.97	90.87	+1.38	30.35	-2.17	hypothetical protein
Smlt2787	0.00	0.00	0	0.00	0	conserved hypothetical protein
Smlt2788	0.00	0.00	0	0.00	0	transmembrane protein
Smlt2789	0.00	0.00	0	0.00	0	hypothetical protein
Smlt2790	0.00	0.00	0	0.00	0	transmembrane protein

Smlt2791	0.00	0.00	0	0.00	0	hypothetical protein
Smlt2792	0.00	0.00	0	0.00	0	Rhs-family transmembrane protein
Smlt2793	0.00	0.00	0	0.00	0	hypothetical protein
Smlt2794	28.25	45.04	+1.59	35.56	+1.26	alpha/beta hydrolase
Smlt2795	47.42	81.31	+1.71	32.51	-1.46	helix-turn-helix transcriptional regulator
Smlt2796	1.53	1.69	+1.1	3.01	+1.97	FUSC family protein
Smlt2797	5.03	8.65	+1.72	11.23	+2.23	biotin/lipoyl-binding protein
Smlt2798	0.00	0.00	0	0.00	0	efflux transporter outer membrane subunit
Smlt2799	0.00	0.00	0	0.00	0	RcnB family protein
Smlt2800	49.70	54.99	+1.11	72.51	+1.46	nuclear transport factor 2 family protein
Smlt2801	33.17	27.85	-1.19	47.22	+1.42	response regulator transcription factor
Smlt2802	0.00	0.00	0	0.00	0	HAMP domain-containing histidine kinase
Smlt2803	5.07	1.93	-2.62	3.37	-1.5	diacylglycerol kinase
Smlt2804	1.46	1.29	-1.13	1.60	+1.1	phosphoethanolamine transferase EptA
Smlt2805	183.13	103.43	-1.77	180.70	-1.01	M14 family metallocarboxypeptidase
Smlt2806	56.37	38.54	-1.46	39.56	-1.42	DUF3348 family protein
Smlt2807	64.70	108.14	+1.67	77.19	+1.19	DUF802 domain-containing protein
Smlt2808	59.82	145.82	+2.44	101.79	+1.7	OmpA family protein
Smlt2809	38.41	72.60	+1.89	42.79	+1.11	DUF2894 domain-containing protein
Smlt2816	6.24	11.35	+1.82	169.82	+27.21	transposase
Smlt2817	5.36	63.24	+11.79	575.72	+107.33	EntA, 2,3-dihydro-2,3-dihydroxybenzoate dehydrogenase
Smlt2818	4.19	91.64	+21.89	1177.56	+281.35	EntF, enterobactin synthase
Smlt2819	0.00	0.00	0	0.00	0	EntB'
Smlt2820	4.17	99.36	+23.83	1952.19	+468.28	EntB, isochorismatase family protein
Smlt2821	2.10	77.81	+37.08	1520.03	+724.47	EntE
Smlt2822	0.63	21.04	+33.32	409.80	+648.97	EntC, isochorismate synthase
Smlt2823	3.59	22.80	+6.35	607.56	+169.22	EntS, MFS transporter
Smlt2824	14.74	33.06	+2.24	99.18	+6.73	DUF3817 domain-containing protein
Smlt2825	85.38	140.52	+1.65	111.03	+1.3	TIGR03571 family LLM class oxidoreductase

Smlt2826	11.95	19.97	+1.67	46.06	+3.85	GNAT family N-acetyltransferase
Smlt2827	13.98	20.24	+1.45	31.98	+2.29	thioredoxin family protein
Smlt2828	4.07	12.41	+3.05	6.67	+1.64	superoxide dismutase
Smlt2829	3.53	6.21	+1.76	2.92	-1.21	flavin reductase family protein
Smlt2830	1.80	4.46	+2.48	2.33	+1.3	tetratricopeptide repeat protein
Smlt2831	541.29	783.20	+1.45	620.14	+1.15	acyl-CoA desaturase
Smlt2832	386.27	755.11	+1.95	451.88	+1.17	ferredoxin reductase
Smlt2833	70.46	172.08	+2.44	155.10	+2.2	HTH-type transcriptional repressor FabR
Smlt2834	0.00	0.00	0	0.00	0	autotransporter outer membrane beta-barrel domain-containing protein
Smlt2835	20.11	15.41	-1.31	62.01	+3.08	TonB-dependent outer membrane receptor
Smlt2836	0.00	0.00	0	0.00	0	redoxin domain-containing protein
Smlt2837	164.72	323.94	+1.97	197.28	+1.2	manganese-binding transcriptional regulator MntR
Smlt2838	64.13	37.66	-1.7	69.19	+1.08	Nramp family divalent metal transporter
Smlt2839	49.80	103.29	+2.07	408.46	+8.2	thioredoxin family protein
Smlt2840	152.68	237.79	+1.56	924.73	+6.06	flavodoxin
Smlt2841	147.17	205.55	+1.4	1383.78	+9.4	ribonucleotide-diphosphate reductase subunit beta
Smlt2842	205.01	248.91	+1.21	1418.56	+6.92	ribonucleoside-diphosphate reductase subunit alpha
Smlt2843	78.25	186.04	+2.38	65.32	-1.2	zinc-binding dehydrogenase
Smlt2844	0.00	0.00	0	0.00	0	hypothetical protein
Smlt2845	2.27	2.85	+1.26	2.44	+1.08	TonB-dependent outer membrane receptor
Smlt2846	155.51	205.42	+1.32	96.24	-1.62	choline BCCT transporter BetT
Smlt2847	0.00	0.00	0	0.00	0	VOC family protein
Smlt2848	0.00	0.00	0	0.00	0	FecI-like RNA polymerase sigma factor
Smlt2849	0.00	0.00	0	0.00	0	FecR-like protein
Smlt2850	0.00	0.00	0	0.00	0	FecA-like TonB-dependent outer membrane receptor
Smlt2851	411.42	985.48	+2.4	175.18	-2.35	multidrug efflux SMR transporter
Smlt2852	869.18	2332.70	+2.68	465.21	-1.87	hypothetical protein
Smlt2853	28.67	59.17	+2.06	30.78	+1.07	LysR family transcriptional regulator

Smlt2857	196.64	183.28	-1.07	153.08	-1.28	M14 family metallocarboxypeptidase
Smlt2858	3.37	18.78	+5.58	145.31	+43.15	TonB-dependent outer membrane receptor
Smlt2859	16.67	12.53	-1.33	22.13	+1.33	chloride channel protein
Smlt2860	19.35	25.06	+1.3	25.58	+1.32	MgtC/SapB family protein
Smlt2861	46.20	136.82	+2.96	29.90	-1.55	MerR family transcriptional regulator
Smlt2862	12.66	14.77	+1.17	14.94	+1.18	class I SAM-dependent methyltransferase
Smlt2863	0.52	2.57	+4.97	1.69	+3.27	hypothetical protein
Smlt2864	2.25	1.89	-1.19	1.45	-1.55	OmpA family protein
Smlt2865	4.13	3.39	-1.22	1.50	-2.75	YadA-like family protein
Smlt2866	7.30	15.35	+2.1	4.25	-1.72	helix-turn-helix domain-containing protein
Smlt2867	19.67	32.55	+1.66	3.22	-6.11	Flp family type IVb pilin
Smlt2868	7.18	10.82	+1.51	1.43	-5.01	prepilin peptidase
Smlt2869	2.85	4.01	+1.41	1.21	-2.34	pilus assembly protein
Smlt2870	2.98	6.17	+2.07	1.62	-1.84	Flp pilus assembly protein CpaB
Smlt2871	3.78	7.55	+2.	3.01	-1.25	type II and III secretion system protein family protein
Smlt2872	2.55	7.00	+2.75	2.01	-1.27	fimbrial protein
Smlt2873	3.18	6.90	+2.17	2.53	-1.26	CpaF family protein
Smlt2874	2.52	5.30	+2.1	2.45	-1.03	type II secretion system F family protein
Smlt2875	2.74	3.47	+1.27	1.83	-1.5	type II secretion system F family protein
Smlt2876	3.13	5.32	+1.7	2.86	-1.09	hypothetical protein
Smlt2877	3.94	7.43	+1.89	2.65	-1.49	DUF3613 domain-containing protein
Smlt2878	0.00	0.00	0	0.00	0	exported protein, pseudogene
Smlt2880	224.92	136.67	-1.65	118.87	-1.89	DUF2968 domain-containing protein
Smlt2882	3.02	2.42	-1.25	2.53	-1.19	alpha/beta hydrolase
Smlt2883	3.11	4.90	+1.57	3.22	+1.03	MFS transporter
Smlt2884	3.41	3.63	+1.07	3.16	-1.08	alginate export family protein
Smlt2885	2.20	2.43	+1.1	2.30	+1.05	amidohydrolase
Smlt2886	1.42	2.34	+1.66	0.95	-1.49	DoxX family membrane protein
Smlt2887	2.07	1.52	-1.36	1.54	-1.34	hydrolase

Smlt2888	0.36	0.20	-1.81	0.28	-1.3	AraC family transcriptional regulator
Smlt2889	0.68	0.25	-2.72	0.52	-1.3	hypothetical protein
Smlt2890	7.17	8.46	+1.18	12.63	+1.76	sensor histidine kinase
Smlt2891	8.53	12.47	+1.46	17.90	+2.1	response regulator transcription factor
Smlt2892	8.78	17.77	+2.02	6.99	-1.26	MFS transporter
Smlt2893	12.97	23.26	+1.79	10.90	-1.19	LysR family transcriptional regulator
Smlt2894	28.86	79.94	+2.77	74.34	+2.58	amidohydrolase family protein
Smlt2895	15.90	39.46	+2.48	41.40	+2.6	ankyrin repeat domain-containing protein
Smlt2896	39.41	39.14	-1.01	66.26	+1.68	LysR family transcriptional regulator
Smlt2897	26.34	33.62	+1.28	108.81	+4.13	DUF3806 domain-containing protein
Smlt2898	9.16	17.20	+1.88	33.56	+3.67	amidohydrolase family protein
Smlt2899	158.19	98.10	-1.61	151.68	-1.04	hypothetical protein
Smlt2900	0.00	0.00	0	0.00	0	hypothetical protein
Smlt2901	4.64	7.41	+1.6	4.18	-1.11	TonB-dependent outer membrane receptor
Smlt2902	0.63	1.11	+1.77	0.43	-1.45	GNAT family N-acetyltransferase
Smlt2903	31.44	28.16	-1.12	43.52	+1.38	CDP-diacylglycerol diphosphatase
Smlt2904	174.66	387.98	+2.22	166.25	-1.05	hypothetical protein
Smlt2905	145.97	65.27	-2.24	66.43	-2.2	hypothetical protein
Smlt2906	80.85	78.21	-1.03	101.52	+1.26	hypothetical protein
Smlt2907	70.48	48.52	-1.45	40.37	-1.75	D-lactate dehydrogenase
Smlt2908	99.40	83.12	-1.2	66.64	-1.49	FMN-dependent L-lactate dehydrogenase LldD
Smlt2909	30.06	24.14	-1.25	29.35	-1.02	transcriptional regulator LldR
Smlt2910	62.99	24.42	-2.58	6.25	-10.07	L-lactate permease
Smlt2911	35.95	42.79	+1.19	33.77	-1.06	MgtC/SapB family protein
Smlt2912	103.91	147.86	+1.42	121.47	+1.17	SDR family oxidoreductase
Smlt2913	77.29	72.64	-1.06	141.68	+1.83	AraC family transcriptional regulator
Smlt2915	37.44	38.09	+1.02	110.24	+2.94	CocE/NonD family hydrolase
Smlt2916	0.00	0.00	0	0.00	0	DUF4440 domain-containing protein
Smlt2917	5.05	5.69	+1.13	3.99	-1.27	MFS transporter

Smlt2918	13.45	12.89	-1.04	11.79	-1.14	LysR family transcriptional regulator
Smlt2919	157.99	116.55	-1.36	131.71	-1.2	glycoside hydrolase family 3 C-terminal domain-containing protein
Smlt2920	14.21	21.99	+1.55	17.12	+1.2	glyoxalase/bleomycin resistance/dioxygenase family protein
Smlt2921	55.07	33.04	-1.67	68.02	+1.24	AraC family transcriptional regulator
Smlt2922	0.60	0.38	-1.59	0.73	+1.21	sterol desaturase family protein
Smlt2923	21.89	10.10	-2.17	21.62	-1.01	LysR family transcriptional regulator
Smlt2924	0.58	0.77	+1.32	0.27	-2.17	DsbA family protein
Smlt2925	1.72	1.32	-1.3	1.03	-1.66	cyclase family protein
Smlt2926	5.15	2.03	-2.54	4.43	-1.16	translesion DNA synthesis-associated protein ImuA
Smlt2927	4.33	2.42	-1.79	5.73	+1.32	DNA polymerase Y family protein
Smlt2928	2.91	2.59	-1.12	3.97	+1.36	error-prone DNA polymerase
Smlt2929	18.89	10.17	-1.86	30.46	+1.61	hypothetical protein
Smlt2930	36.91	26.89	-1.37	18.64	-1.98	transcriptional regulator PtsJ
Smlt2931	168.91	66.43	-2.54	17.03	-9.92	YggS family pyridoxal phosphate-dependent enzyme
Smlt2932	92.80	41.83	-2.22	10.03	-9.25	glutamine amidotransferase
Smlt2933	110.58	58.42	-1.89	14.94	-7.4	hypothetical protein
Smlt2934	21.27	10.67	-1.99	7.43	-2.86	hypothetical protein
Smlt2935	0.89	3.91	+4.41	219.80	+247.8	FecI-like RNA polymerase sigma factor
Smlt2936	1.87	5.56	+2.97	384.21	+205.39	FecR-like protein
Smlt2937	0.91	7.47	+8.17	311.95	+340.93	FecA-like TonB-dependent outer membrane receptor
Smlt2938	1.32	12.77	+9.69	383.34	+290.9	iron regulated lipoprotein
Smlt2939	0.84	8.27	+9.79	387.97	+459.26	TonB, energy transducer
Smlt2940	575.09	186.28	-3.09	25.44	-22.6	RidA family protein
Smlt2941	1512.85	719.81	-2.1	98.31	-15.39	cation diffusion facilitator family transporter
Smlt2942	4629.32	1910.46	-2.42	308.97	-14.98	lysine decarboxylase
Smlt2943	510.33	254.81	-2.	88.55	-5.76	arginine/agmatine antiporter
Smlt2944	1663.97	866.51	-1.92	314.22	-5.3	porin
Smlt2946	220.37	281.85	+1.28	371.67	+1.69	thiol:disulfide interchange protein DsbG

Smlt2947	55.61	70.57	+1.27	61.91	+1.11	TlpA family protein disulfide reductase
Smlt2948	10.84	19.16	+1.77	6.22	-1.74	helix-turn-helix domain-containing protein
Smlt2949	60.35	71.45	+1.18	210.00	+3.48	heavy metal-responsive transcriptional regulator
Smlt2950	198.52	441.90	+2.23	90.46	-2.19	GNAT family N-acetyltransferase
Smlt2951	14.65	13.21	-1.11	20.07	+1.37	SOS response-associated peptidase family protein
Smlt2952	123.68	98.48	-1.26	100.08	-1.24	response regulator
Smlt2953	7.31	8.18	+1.12	6.08	-1.2	GGDEF domain-containing protein
Smlt2954	5.12	3.01	-1.7	2.78	-1.84	methyl-accepting chemotaxis protein
Smlt2955	0.00	0.00	0	0.00	0	acetyltransferase
Smlt2956	23.05	19.86	-1.16	20.99	-1.1	DUF5694 domain-containing protein
Smlt2957	0.00	0.00	0	0.00	0	antibiotic biosynthesis monooxygenase
Smlt2958	0.00	0.00	0	0.00	0	hypothetical protein
Smlt2959	96.64	100.67	+1.04	107.35	+1.11	PAS domain S-box protein
Smlt2960	178.77	213.21	+1.19	199.15	+1.11	RNA-binding transcriptional accessory protein
Smlt2961	1371.44	2141.45	+1.56	998.10	-1.37	hypothetical protein
Smlt2962	41.61	56.01	+1.35	41.83	+1.01	hypothetical protein
Smlt2963	80.10	88.60	+1.11	97.05	+1.21	YfeK family protein
Smlt2964	76.88	83.27	+1.08	131.84	+1.71	class III poly(R)-hydroxyalkanoic acid synthase subunit PhaC
Smlt2965	82.48	85.69	+1.04	155.56	+1.89	class III poly(R)-hydroxyalkanoic acid synthase subunit PhaE
Smlt2966	91.21	76.84	-1.19	201.21	+2.21	phosphatidylcholine/phosphatidylserine synthase
Smlt2967	69.26	62.24	-1.11	226.04	+3.26	class I SAM-dependent methyltransferase
Smlt2970	7.13	11.28	+1.58	2.10	-3.39	MATE family efflux transporter
Smlt2971	74.20	172.72	+2.33	42.72	-1.74	DUF2239 family protein
Smlt2972	182.86	89.32	-2.05	165.07	-1.11	8-oxo-dGTP diphosphatase
Smlt2973	75.69	96.90	+1.28	83.40	+1.1	DUF1249 domain-containing protein
Smlt2974	152.81	143.21	-1.07	156.99	+1.03	kinase/pyrophosphorylase
Smlt2975	791.99	975.69	+1.23	1054.03	+1.33	phosphoenolpyruvate synthase
Smlt2977	82.62	105.27	+1.27	80.52	-1.03	mechanosensitive ion channel

Smlt2978	14.80	32.13	+2.17	9.61	-1.54	alkene reductase
Smlt2979	91.06	158.79	+1.74	113.78	+1.25	EcsC family protein
Smlt2981	239.87	208.64	-1.15	321.79	+1.34	oligoribonuclease
Smlt2982	112.45	58.63	-1.92	101.16	-1.11	tRNA adenosine(34) deaminase TadA
Smlt2983	25.13	20.10	-1.25	23.58	-1.07	helix-turn-helix transcriptional regulator
Smlt2984	2.06	1.95	-1.06	3.13	+1.52	NAD(P)H-dependent oxidoreductase
Smlt2985	75.40	75.98	+1.01	96.97	+1.29	acetyltransferase
Smlt2986	104.53	75.16	-1.39	119.95	+1.15	hypothetical protein
Smlt2987	68.85	54.81	-1.26	58.57	-1.18	N-acetyltransferase
Smlt2988	54.40	51.92	-1.05	79.64	+1.46	hypothetical protein
Smlt2989	85.54	86.63	+1.01	195.55	+2.29	hypothetical protein
Smlt2990	139.96	157.50	+1.13	278.92	+1.99	lipase
Smlt2991	22.60	14.05	-1.61	13.03	-1.73	hypothetical protein
Smlt2992	74.42	85.92	+1.15	57.86	-1.29	hypothetical protein
Smlt2993	214.67	210.69	-1.02	150.32	-1.43	hypothetical protein
Smlt2994	137.82	112.13	-1.23	85.66	-1.61	DUF4189 domain-containing protein
Smlt2995	0.00	0.00	0	0.00	0	DUF4189 domain-containing protein
Smlt2996	0.00	0.00	0	0.00	0	DUF4189 domain-containing protein
Smlt2997	302.71	400.64	+1.32	211.63	-1.43	type IV secretion system protein
Smlt2998	388.27	366.33	-1.06	224.72	-1.73	hypothetical protein
Smlt2999	287.29	179.40	-1.6	155.32	-1.85	VirB4 family type IV secretion/conjugal transfer ATPase
Smlt3000	916.95	450.70	-2.03	581.99	-1.58	VirB3 family type IV secretion system protein
Smlt3001	1438.23	807.94	-1.78	820.70	-1.75	TrbC/VirB2 family protein
Smlt3002	190.99	120.48	-1.59	99.28	-1.92	transglycosylase SLT domain-containing protein
Smlt3003	196.58	120.81	-1.63	120.00	-1.64	P-type DNA transfer ATPase VirB11
Smlt3004	281.44	136.53	-2.06	98.47	-2.86	TrbI/VirB10 family protein
Smlt3005	231.41	117.54	-1.97	57.57	-4.02	TrbG/VirB9 family P-type conjugative transfer protein
Smlt3006	220.64	103.93	-2.12	52.80	-4.18	type IV secretion system protein
Smlt3007	405.32	270.24	-1.5	88.86	-4.56	hypothetical protein

Smlt3008	138.50	77.94	-1.78	76.11	-1.82	type IV secretory system conjugative DNA transfer family protein
Smlt3009	151.08	162.84	+1.08	93.99	-1.61	hypothetical protein
Smlt3010	0.00	0.00	0	0.00	0	HD domain-containing protein
Smlt3011	17.17	8.51	-2.02	12.62	-1.36	MFS transporter
Smlt3012	48.54	44.20	-1.1	22.33	-2.17	MerR family transcriptional regulator
Smlt3013	3.53	4.03	+1.14	2.34	-1.51	NADH:flavin oxidoreductase/NADH oxidase family protein
Smlt3014	47.73	34.01	-1.4	48.90	+1.02	membrane protein
Smlt3015	0.00	0.00	0	0.00	0	hypothetical protein
Smlt3016	0.00	0.00	0	0.00	0	hypothetical protein
Smlt3017	0.00	0.00	0	0.00	0	hypothetical protein
Smlt3018	11.81	24.39	+2.07	22.92	+1.94	hypothetical protein
Smlt3019	21.46	18.31	-1.17	25.81	+1.2	PAS domain-containing hybrid sensor histidine kinase/response regulator
Smlt3020	16.49	41.05	+2.49	11.39	-1.45	FAD-dependent monooxygenase
Smlt3021	46.52	144.46	+3.11	15.54	-2.99	TetR/AcrR family transcriptional regulator
Smlt3022	7.46	35.46	+4.75	629.93	+84.42	TonB-dependent outer membrane receptor
Smlt3023	190.39	242.02	+1.27	375.77	+1.97	hypothetical protein
Smlt3024	101.55	101.55	+1.	148.28	+1.46	hypothetical protein
Smlt3025	121.12	110.15	-1.1	199.32	+1.65	hypothetical protein
Smlt3026	52.00	77.93	+1.5	73.35	+1.41	helix-turn-helix transcriptional regulator
Smlt3027	50.80	33.78	-1.5	58.56	+1.15	oxidoreductase
Smlt3028	101.47	170.27	+1.68	122.48	+1.21	hypothetical protein
Smlt3029	0.00	0.00	0	0.00	0	ankyrin repeat domain-containing protein
Smlt3030	49.22	42.17	-1.17	61.92	+1.26	UvrD-helicase domain-containing protein
Smlt3031	0.00	0.00	0	0.00	0	phage-related protein
Smlt3032	0.00	0.00	0	0.00	0	conserved hypothetical protein
Smlt3033	0.00	0.00	0	0.00	0	ankyrin repeat domain-containing protein
Smlt3034	0.00	0.00	0	0.00	0	AAA family ATPase
Smlt3035	0.00	0.00	0	0.00	0	hypothetical protein

Smlt3036	0.00	0.00	0	0.00	0	hypothetical protein
Smlt3037	0.00	0.00	0	0.00	0	hypothetical protein
Smlt3038	0.00	0.00	0	0.00	0	hypothetical protein
Smlt3039	0.00	0.00	0	0.00	0	Y-family DNA polymerase
Smlt3040	0.00	0.00	0	0.00	0	hypothetical protein
Smlt3041	0.00	0.00	0	0.00	0	hypothetical protein
Smlt3042	0.00	0.00	0	0.00	0	ISXac3 like transposase
Smlt3043	5.76	10.69	+1.86	1.91	-3.01	IS3-like element ISStma14 family transposase
Smlt3044	0.00	0.00	0	0.00	0	integrase, pseudogene
Smlt3045	0.00	0.00	0	0.00	0	hypothetical protein
Smlt3046	0.00	0.00	0	0.00	0	M15 family metallopeptidase
Smlt3048	0.00	0.00	0	0.00	0	adhesin
Smlt3049	0.00	0.00	0	0.00	0	hypothetical protein
Smlt3050	0.00	0.00	0	0.00	0	GIY-YIG nuclease family protein
Smlt3051	0.00	0.00	0	0.00	0	relaxase domain-containing protein
Smlt3052	0.00	0.00	0	0.00	0	HIRAN domain-containing protein
Smlt3053	0.00	0.00	0	0.00	0	DUF853 family protein
Smlt3054	0.00	0.00	0	0.00	0	ankyrin repeat domain-containing protein
Smlt3055	0.00	0.00	0	0.00	0	hypothetical protein
Smlt3056	0.00	0.00	0	0.00	0	hypothetical protein
Smlt3057	0.00	0.00	0	0.00	0	hypothetical protein
Smlt3058	0.00	0.00	0	0.00	0	TraM recognition domain-containing protein
Smlt3059	0.00	0.00	0	0.00	0	hypothetical protein
Smlt3060	0.00	0.00	0	0.00	0	hypothetical protein
Smlt3061	0.00	0.00	0	0.00	0	hypothetical protein
Smlt3062	0.00	0.00	0	0.00	0	hypothetical protein
Smlt3062A	0.00	0.00	0	0.00	0	hypothetical protein
Smlt3062AA	0.00	0.00	0	0.00	0	hypothetical protein
Smlt3065	0.00	0.00	0	0.00	0	HNH endonuclease

Smlt3066	0.00	0.00	0	0.00	0	hypothetical protein
Smlt3067	0.00	0.00	0	0.00	0	hypothetical protein
Smlt3068	0.00	0.00	0	0.00	0	hypothetical protein
Smlt3069	0.00	0.00	0	0.00	0	ParA family protein
Smlt3070	0.00	0.00	0	0.00	0	hypothetical protein
Smlt3071	0.00	0.00	0	0.00	0	type IV pilus protein
Smlt3073	0.00	0.00	0	0.00	0	hypothetical protein
Smlt3074	0.00	0.00	0	0.00	0	hypothetical protein
Smlt3075	0.00	0.00	0	0.00	0	hypothetical protein
Smlt3076	0.00	0.00	0	0.00	0	conserved hypothetical protein
Smlt3077	0.00	0.00	0	0.00	0	hypothetical protein
Smlt3078	0.00	0.00	0	0.00	0	hypothetical protein
Smlt3079	0.00	0.00	0	0.00	0	hypothetical protein
Smlt3080	0.00	0.00	0	0.00	0	hypothetical protein
Smlt3081	0.00	0.00	0	0.00	0	hypothetical protein
Smlt3082	0.00	0.00	0	0.00	0	hypothetical protein
Smlt3083	0.00	0.00	0	0.00	0	transposase
Smlt3083A	0.00	0.00	0	0.00	0	transposase, pseudogene
Smlt3084	0.00	0.00	0	0.00	0	hypothetical protein
Smlt3085	0.00	0.00	0	0.00	0	hypothetical protein
Smlt3086	0.00	0.00	0	0.00	0	site-specific integrase
Smlt3087	0.00	0.00	0	0.00	0	conserved hypothetical protein, pseudogene
Smlt3089	222.24	333.53	+1.5	244.91	+1.1	LysR family transcriptional regulator
Smlt3090	5.64	8.35	+1.48	4.83	-1.17	NAD(P)H-dependent oxidoreductase
Smlt3091	61.00	217.82	+3.57	74.87	+1.23	hypothetical protein
Smlt3092	212.95	174.70	-1.22	201.93	-1.05	serine--tRNA ligase
Smlt3093	42.12	81.24	+1.93	54.32	+1.29	TonB, energy transducer
Smlt3094	78.42	504.68	+6.44	60.58	-1.29	TonB, energy transducer
Smlt3095	0.00	0.00	0	0.00	0	hypothetical protein

Smlt3096	74.88	105.84	+1.41	127.17	+1.7	3-phosphoshikimate 1-carboxyvinyltransferase
Smlt3097	172.74	177.29	+1.03	298.50	+1.73	prephenate dehydratase
Smlt3098	152.94	129.16	-1.18	203.67	+1.33	3-phosphoserine/phosphohydroxythreonine transaminase
Smlt3099	95.39	69.89	-1.36	105.27	+1.1	FHA domain-containing protein
Smlt3100	142.32	56.06	-2.54	31.60	-4.5	polyhydroxyalkanoic acid system family protein
Smlt3101	125.51	169.60	+1.35	68.13	-1.84	phasin family protein
Smlt3103	34.38	21.89	-1.57	48.23	+1.4	restriction endonuclease
Smlt3104	0.00	0.00	0	0.00	0	type II toxin-antitoxin system HicB family antitoxin
Smlt3105	46.23	75.20	+1.63	72.21	+1.56	histidine utilization repressor
Smlt3106	81.89	222.39	+2.72	188.34	+2.3	formimidoylglutamate deiminase
Smlt3107	56.18	103.79	+1.85	107.21	+1.91	imidazolonepropionase
Smlt3108	146.00	283.89	+1.94	323.78	+2.22	histidine ammonia-lyase
Smlt3109	73.63	177.19	+2.41	234.06	+3.18	N-formylglutamate deformylase
Smlt3110	188.44	554.87	+2.94	405.87	+2.15	urocanate hydratase
Smlt3111	0.00	0.00	0	0.00	0	hypothetical protein
Smlt3112	0.00	0.00	0	0.00	0	hypothetical protein
Smlt3114	0.00	0.00	0	0.00	0	serine hydrolase
Smlt3115	13.57	10.04	-1.35	9.95	-1.36	TonB-dependent outer membrane receptor
Smlt3116	235.90	457.99	+1.94	205.30	-1.15	hypothetical protein
Smlt3117	0.00	0.00	0	0.00	0	hypothetical protein
Smlt3118	0.00	0.00	0	0.00	0	hypothetical protein
Smlt3119	0.00	0.00	0	0.00	0	transmembrane protein
Smlt3120	0.00	0.00	0	0.00	0	nuclear transport factor 2 family protein
Smlt3121	27.88	26.14	-1.07	27.11	-1.03	TetR/AcrR family transcriptional regulator
Smlt3122	24.58	9.45	-2.6	22.50	-1.09	thioesterase family protein
Smlt3123	81.11	56.51	-1.44	99.94	+1.23	SGNH/GDSL hydrolase family protein
Smlt3124	26.77	8.44	-3.17	41.51	+1.55	DUF2268 domain-containing putative Zn-dependent protease
Smlt3125	33.48	39.48	+1.18	57.80	+1.73	hypothetical protein
Smlt3126	95.13	625.89	+6.58	478.28	+5.03	hypothetical protein

Smlt3128	21.43	22.56	+1.05	44.30	+2.07	prolyl oligopeptidase family serine peptidase
Smlt3129	77.45	216.30	+2.79	68.48	-1.13	hypothetical protein
Smlt3130	64.92	44.76	-1.45	94.79	+1.46	hypothetical protein
Smlt3131	65.08	56.50	-1.15	83.53	+1.28	DUF3037 domain-containing protein
Smlt3132	0.00	0.00	0	0.00	0	serine hydrolase
Smlt3133	0.00	0.00	0	0.00	0	conserved hypothetical protein
Smlt3134	0.00	0.00	0	0.00	0	LysR family transcriptional regulator
Smlt3135	0.00	0.00	0	0.00	0	zinc-dependent alcohol dehydrogenase family protein
Smlt3136	6.35	7.80	+1.23	5.45	-1.16	membrane-bound PQQ-dependent dehydrogenase, glucose/quinate/shikimate family
Smlt3137	650.32	688.86	+1.06	605.32	-1.07	DNA gyrase subunit A
Smlt3138	176.56	207.24	+1.17	187.60	+1.06	S-methyl-5-thioribose-1-phosphate isomerase
Smlt3139	176.03	262.13	+1.49	222.49	+1.26	DUF3011 domain-containing protein
Smlt3140	90.76	71.02	-1.28	125.01	+1.38	EF-P lysine aminoacylase GenX
Smlt3141	138.69	108.01	-1.28	175.69	+1.27	GNAT family N-acetyltransferase
Smlt3142	97.20	71.95	-1.35	119.01	+1.22	NAD-dependent DNA ligase LigA
Smlt3143	17.96	13.68	-1.31	29.44	+1.64	pyridoxal phosphate-dependent aminotransferase
Smlt3144	188.95	269.56	+1.43	207.90	+1.1	cell division protein ZipA
Smlt3145	174.27	247.37	+1.42	186.09	+1.07	chromosome segregation protein SMC
Smlt3146	0.00	0.00	0	0.00	0	hypothetical protein
Smlt3147	0.00	0.00	0	0.00	0	DUF1963 domain-containing protein
Smlt3148	969.76	843.21	-1.15	657.63	-1.47	50S ribosomal protein L9
Smlt3149	2319.98	1599.04	-1.45	1598.67	-1.45	30S ribosomal protein S18
Smlt3150	1641.33	850.54	-1.93	961.60	-1.71	30S ribosomal protein S6
Smlt3152	97.06	96.22	-1.01	62.59	-1.55	CbrC family protein
Smlt3153	102.53	145.04	+1.41	118.88	+1.16	iron-sulfur cluster assembly accessory protein
Smlt3154	298.77	149.69	-2.	366.62	+1.23	asparagine--tRNA ligase
Smlt3155	94.50	81.81	-1.16	147.76	+1.56	hypothetical protein
Smlt3156	125.66	114.09	-1.1	121.41	-1.03	hypothetical protein

Smlt3157	30.21	27.73	-1.09	42.62	+1.41	FMN-binding negative transcriptional regulator
Smlt3158	130.21	91.73	-1.42	131.80	+1.01	carbonate dehydratase
Smlt3159	117.86	116.82	-1.01	228.40	+1.94	3-hydroxyanthranilate 3,4-dioxygenase
Smlt3160	167.11	159.73	-1.05	258.49	+1.55	kynureninase
Smlt3161	81.68	50.17	-1.63	98.31	+1.2	FAD-dependent monooxygenase
Smlt3162	84.51	73.55	-1.15	133.38	+1.58	exodeoxyribonuclease I
Smlt3163	112.71	104.14	-1.08	170.68	+1.51	DUF2461 domain-containing protein
Smlt3164	79.90	229.99	+2.88	87.86	+1.1	DUF2939 domain-containing protein
Smlt3165	54.49	56.43	+1.04	83.61	+1.53	5'-nucleotidase
Smlt3166	39.06	58.36	+1.49	53.49	+1.37	NAD kinase
Smlt3167	769.74	1127.75	+1.47	886.24	+1.15	NAD-glutamate dehydrogenase
Smlt3169	31.56	42.04	+1.33	47.45	+1.5	TetR/AcrR family transcriptional regulator
Smlt3170	99.28	117.22	+1.18	141.33	+1.42	efflux RND transporter periplasmic adaptor subunit
Smlt3171	169.65	147.46	-1.15	241.84	+1.43	multidrug efflux RND transporter permease subunit
Smlt3172	44.37	14.43	-3.07	38.73	-1.15	hypothetical protein
Smlt3173	5.50	10.60	+1.93	7.35	+1.34	hypothetical protein
Smlt3174	652.64	552.38	-1.18	817.35	+1.25	acyl-CoA dehydrogenase family protein
Smlt3175	197.57	602.57	+3.05	120.44	-1.64	metalloregulator ArsR/SmtB family transcription factor
Smlt3176	92.31	238.23	+2.58	53.70	-1.72	homocysteine S-methyltransferase family protein
Smlt3177	67.67	212.65	+3.14	55.95	-1.21	methionine synthase
Smlt3178	8.38	10.15	+1.21	9.37	+1.12	MFS transporter
Smlt3179	237.70	248.72	+1.05	362.44	+1.52	DUF2058 family protein
Smlt3179A	107.04	135.57	+1.27	143.32	+1.34	SlyX family protein
Smlt3181	487.67	655.56	+1.34	598.80	+1.23	UDP-glucose/GDP-mannose dehydrogenase family protein
Smlt3182	979.45	1189.12	+1.21	1245.87	+1.27	FKBP-type peptidyl-prolyl cis-trans isomerase
Smlt3183	114.25	151.81	+1.33	130.63	+1.14	glutathione peroxidase
Smlt3184	16.22	37.91	+2.34	19.44	+1.2	hypothetical protein
Smlt3185	33.51	63.09	+1.88	24.71	-1.36	GntR family transcriptional regulator
Smlt3186	55.17	110.07	+2.	46.87	-1.18	ABC transporter ATP-binding protein

Smlt3187	72.25	151.83	+2.1	73.71	+1.02	membrane protein
Smlt3188	72.08	173.97	+2.41	74.46	+1.03	DUF4097 family beta strand repeat-containing protein
Smlt3189	47.55	120.17	+2.53	53.87	+1.13	hypothetical protein
Smlt3190	110.09	124.59	+1.13	126.98	+1.15	hypothetical protein
Smlt3191	223.07	246.03	+1.1	1371.70	+6.15	class II fumarate hydratase
Smlt3193	194.17	152.64	-1.27	261.16	+1.35	adenylosuccinate lyase
Smlt3195	177.04	252.99	+1.43	163.57	-1.08	cupin domain-containing protein
Smlt3196	86.66	115.93	+1.34	92.93	+1.07	GNAT family N-acetyltransferase
Smlt3197	738.73	870.44	+1.18	843.56	+1.14	2-oxoglutarate dehydrogenase E1 component
Smlt3198	503.96	554.97	+1.1	509.96	+1.01	dihydrolipoyllysine-residue succinyltransferase
Smlt3199	1138.81	1390.72	+1.22	1289.31	+1.13	dihydrolipoyl dehydrogenase
Smlt3200	0.00	0.00	0	0.00	0	hypothetical protein
Smlt3201	1.06	0.78	-1.36	1.77	+1.67	hypothetical protein
Smlt3202	2.75	3.03	+1.1	2.90	+1.05	alkaline phosphatase D family protein
Smlt3203	5.98	2.93	-2.04	5.51	-1.08	TonB-dependent outer membrane receptor
Smlt3204	285.83	213.88	-1.34	229.52	-1.25	replicative DNA helicase
Smlt3205	107.65	102.17	-1.05	195.17	+1.81	NAD(P)H-dependent oxidoreductase
Smlt3206	78.38	114.26	+1.46	103.00	+1.31	asparagine synthase (glutamine-hydrolyzing)
Smlt3207	74.60	143.18	+1.92	107.52	+1.44	MFS transporter
Smlt3208	151.82	303.27	+2.	229.01	+1.51	glutathione S-transferase family protein
Smlt3209	103.17	169.30	+1.64	53.25	-1.94	GNAT family N-acetyltransferase
Smlt3210	1162.26	3443.63	+2.96	646.16	-1.8	glycine zipper 2TM domain-containing protein
Smlt3211	42.35	87.75	+2.07	61.12	+1.44	hypothetical protein
Smlt3212	68.28	84.59	+1.24	58.66	-1.16	TatD family hydrolase
Smlt3213	255.87	256.21	+1.	178.18	-1.44	tRNA threonylcarbamoyladenosine dehydratase
Smlt3214	25.48	21.42	-1.19	21.98	-1.16	hypothetical protein
Smlt3215	273.86	331.09	+1.21	134.10	-2.04	glycine zipper 2TM domain-containing protein
Smlt3216	1361.08	1625.43	+1.19	2044.26	+1.5	cold-shock protein
Smlt3217	19.39	14.04	-1.38	7.51	-2.58	DUF456 domain-containing protein

Smlt3218	166.59	171.21	+1.03	184.93	+1.11	phospholipase A
Smlt3219	162.26	201.90	+1.24	183.67	+1.13	arsenate reductase (glutaredoxin)
Smlt3220	212.44	177.64	-1.2	272.47	+1.28	glutathione S-transferase family protein
Smlt3221	115.21	156.32	+1.36	349.13	+3.03	hypothetical protein
Smlt3222	561.15	451.26	-1.24	38.36	-14.63	fumarate hydratase
Smlt3223	4.20	4.08	-1.03	4.72	+1.12	RNA polymerase sigma-70 factor
Smlt3224	11.48	24.06	+2.09	12.15	+1.06	carboxymuconolactone decarboxylase family protein
Smlt3226	31.41	28.75	-1.09	40.23	+1.28	ABC transporter ATP-binding protein/permease
Smlt3227	192.26	171.19	-1.12	520.14	+2.71	ferredoxin--NADP reductase
Smlt3228	48.06	84.41	+1.76	71.83	+1.49	glutathione peroxidase
Smlt3229	43.94	94.05	+2.14	112.69	+2.56	M3 family metallopeptidase
Smlt3230	0.00	0.00	0	0.00	0	hypothetical protein
Smlt3231	99.72	95.12	-1.05	68.59	-1.45	cyclopropane fatty acyl phospholipid synthase
Smlt3232	314.24	277.97	-1.13	169.47	-1.85	DUF3298 and DUF4163 domain-containing protein
Smlt3233	456.36	309.16	-1.48	368.71	-1.24	N-acetyltransferase
Smlt3234	255.04	31.84	-8.01	88.35	-2.89	hypothetical protein
Smlt3235	113.27	200.35	+1.77	220.33	+1.95	rhomboid family intramembrane serine protease
Smlt3236	95.71	121.44	+1.27	232.78	+2.43	oligopeptide:H+ symporter
Smlt3237	110.03	271.53	+2.47	73.88	-1.49	endonuclease/exonuclease/phosphatase family protein
Smlt3238	4558.38	2100.76	-2.17	2487.00	-1.83	superoxide dismutase
Smlt3239	42.77	81.17	+1.9	58.71	+1.37	ribonuclease
Smlt3240	42.43	90.43	+2.13	36.46	-1.16	barstar family protein
Smlt3241	46.85	93.79	+2.	59.14	+1.26	hypothetical protein
Smlt3242	345.56	628.52	+1.82	323.73	-1.07	NfuA family Fe-S biogenesis protein
Smlt3243	188.60	343.68	+1.82	253.99	+1.35	4a-hydroxytetrahydrobiopterin dehydratase
Smlt3244	202.86	312.62	+1.54	211.50	+1.04	TonB, energy transducer
Smlt3245	17.59	30.91	+1.76	38.17	+2.17	zinc transporter ZupT
Smlt3246	75.78	59.73	-1.27	107.13	+1.41	RluA family pseudouridine synthase
Smlt3247	547.10	729.72	+1.33	429.27	-1.27	Rne/Rng family ribonuclease

Smlt3248	108.20	43.71	-2.48	54.93	-1.97	endonuclease/exonuclease/phosphatase family protein
Smlt3249	107.33	64.73	-1.66	84.71	-1.27	discoidin domain-containing protein
Smlt3250	124.85	65.20	-1.91	89.13	-1.4	carbohydrate ABC transporter permease
Smlt3251	82.06	41.22	-1.99	69.61	-1.18	sugar ABC transporter permease
Smlt3252	104.86	49.88	-2.1	66.57	-1.58	sugar ABC transporter substrate-binding protein
Smlt3253	120.35	48.93	-2.46	103.55	-1.16	hypothetical protein
Smlt3254	0.00	0.00	0	0.00	0	TonB-dependent outer membrane receptor
Smlt3255	269.26	165.10	-1.63	271.25	+1.01	LacI family transcriptional regulator
Smlt3256	170.54	106.30	-1.6	195.22	+1.14	BolA family transcriptional regulator
Smlt3257	118.62	93.73	-1.27	120.14	+1.01	YciI family protein
Smlt3259	238.75	307.15	+1.29	121.07	-1.97	segregation/condensation protein A
Smlt3260	116.79	154.70	+1.32	84.08	-1.39	SMC-Scp complex subunit ScpB
Smlt3261	103.68	211.69	+2.04	108.65	+1.05	rRNA pseudouridine synthase
Smlt3262	13.86	21.30	+1.54	13.48	-1.03	helix-turn-helix transcriptional regulator
Smlt3263	1.63	2.30	+1.41	0.83	-1.97	MFS transporter
Smlt3264	4.10	3.68	-1.12	3.15	-1.3	hypothetical protein
Smlt3265	209.38	211.71	+1.01	212.47	+1.01	hypothetical protein
Smlt3266	92.21	87.11	-1.06	146.34	+1.59	amidohydrolase
Smlt3267	75.99	76.17	+1.	116.78	+1.54	pyridoxal phosphate-dependent aminotransferase
Smlt3268	44.76	27.33	-1.64	48.43	+1.08	heme ABC exporter ATP-binding protein CcmA
Smlt3269	46.47	58.81	+1.27	61.69	+1.33	heme exporter protein CcmB
Smlt3270	82.53	75.42	-1.09	52.61	-1.57	heme ABC transporter permease CcmC
Smlt3271	0.00	0.00	0	0.00	0	heme exporter protein CcmD
Smlt3272	59.89	60.86	+1.02	44.02	-1.36	cytochrome c maturation protein CcmE
Smlt3273	37.50	38.87	+1.04	32.79	-1.14	heme lyase CcmF/NrfE family subunit
Smlt3274	168.48	175.27	+1.04	166.01	-1.01	DsbE family thiol:disulfide interchange protein
Smlt3275	133.03	156.59	+1.18	179.18	+1.35	cytochrome c-type biogenesis protein CcmH
Smlt3276	61.46	90.84	+1.48	82.27	+1.34	cytochrome c biogenesis protein
Smlt3277	123.76	121.46	-1.02	174.67	+1.41	homoserine O-acetyltransferase

Smlt3278	14.42	14.71	+1.02	13.10	-1.1	DUF1294 domain-containing protein
Smlt3279	209.03	166.39	-1.26	73.28	-2.85	thiol reductant ABC exporter subunit CydC
Smlt3280	193.64	111.37	-1.74	49.71	-3.89	thiol reductant ABC exporter subunit CydD
Smlt3282	3245.63	1437.83	-2.26	393.87	-8.24	cytochrome ubiquinol oxidase subunit I
Smlt3283	2142.76	960.19	-2.23	215.04	-9.96	cytochrome d ubiquinol oxidase subunit II
Smlt3284	1393.21	754.43	-1.85	89.64	-15.54	cytochrome bd-I oxidase subunit CydX
Smlt3287	31.95	19.87	-1.61	16.55	-1.93	MFS transporter
Smlt3289	14.09	22.97	+1.63	43.00	+3.05	poly-beta-1,6 N-acetyl-D-glucosamine export porin PgaA
Smlt3290	3.04	8.82	+2.9	9.37	+3.08	poly-beta-1,6-N-acetyl-D-glucosamine N-deacetylase PgaB
Smlt3291	2.46	9.00	+3.66	10.00	+4.07	poly-beta-1,6 N-acetyl-D-glucosamine synthase
Smlt3292	5.12	19.78	+3.86	13.72	+2.68	poly-beta-1,6-N-acetyl-D-glucosamine biosynthesis protein PgaD
Smlt3293	52.69	79.14	+1.5	74.53	+1.41	glutamate-5-semialdehyde dehydrogenase
Smlt3294	15.63	25.50	+1.63	13.89	-1.13	glutamate 5-kinase
Smlt3295	25.42	48.53	+1.91	38.08	+1.5	YciI family protein
Smlt3296	37.87	64.71	+1.71	43.15	+1.14	argininosuccinate lyase
Smlt3297	37.62	45.78	+1.22	26.97	-1.39	N-acetyl-gamma-glutamyl-phosphate reductase
Smlt3298	0.00	0.00	0	0.00	0	GNAT family N-acetyltransferase
Smlt3299	36.19	62.16	+1.72	22.21	-1.63	acetylglutamate kinase
Smlt3300	35.24	73.03	+2.07	23.97	-1.47	acetylornithine deacetylase
Smlt3301	48.94	101.68	+2.08	33.35	-1.47	argininosuccinate synthase
Smlt3302	34.16	68.87	+2.02	20.18	-1.69	N-acetylornithine carbamoyltransferase
Smlt3303	0.00	0.00	0	0.00	0	transmembrane protein
Smlt3304	339.01	601.82	+1.78	245.86	-1.38	hypothetical protein
Smlt3305	178.77	108.39	-1.65	156.92	-1.14	cysteine--tRNA ligase
Smlt3306	50.76	35.08	-1.45	57.82	+1.14	SufE family protein
Smlt3307	42.17	56.00	+1.33	49.66	+1.18	MFS transporter
Smlt3308	373.55	342.65	-1.09	346.62	-1.08	RNA polymerase-binding protein DksA
Smlt3309	27.50	37.84	+1.38	42.83	+1.56	membrane protein insertion efficiency factor YidD
Smlt3310	243.15	374.28	+1.54	346.92	+1.43	dihydroorotase

Smlt3311	170.42	280.84	+1.65	219.88	+1.29	M23 family metallopeptidase
Smlt3312	105.34	161.19	+1.53	151.65	+1.44	phytoene synthase
Smlt3313	92.95	100.88	+1.09	93.37	+1.	phosphoglycolate phosphatase
Smlt3314	115.35	141.56	+1.23	123.04	+1.07	bifunctional 2-polyprenyl-6-hydroxyphenol methylase/3-demethylubiquinol 3-O-methyltransferase UbiG
Smlt3315	113.65	92.73	-1.23	106.02	-1.07	TRZ/ATZ family hydrolase
Smlt3316	1596.48	1325.51	-1.2	1299.70	-1.23	elongation factor P
Smlt3317	72.80	73.62	+1.01	75.55	+1.04	EF-P beta-lysylation protein EpmB
Smlt3318	61.32	46.62	-1.32	50.24	-1.22	GGDEF domain-containing protein
Smlt3319	183.44	169.95	-1.08	122.63	-1.5	phosphate/phosphite/phosphonate ABC transporter substrate-binding protein
Smlt3320	87.03	92.90	+1.07	92.93	+1.07	RNA methyltransferase
Smlt3321	315.52	346.74	+1.1	156.87	-2.01	inositol monophosphatase
Smlt3323	92.18	377.30	+4.09	64.03	-1.44	protease HtpX
Smlt3324	36.50	28.00	-1.3	52.10	+1.43	tRNA glutamyl-Q(34) synthetase GluQRS
Smlt3325	103.89	110.59	+1.06	168.83	+1.63	acetoacetyl-CoA reductase
Smlt3326	213.48	253.68	+1.19	292.53	+1.37	polyhydroxyalkanoate synthesis repressor PhaR
Smlt3327	136.61	190.38	+1.39	155.22	+1.14	TraB/GumN family protein
Smlt3328	97.45	84.45	-1.15	109.13	+1.12	DUF1684 domain-containing protein
Smlt3329	252.11	339.89	+1.35	337.70	+1.34	DNA mismatch repair endonuclease MutL
Smlt3330	262.22	363.11	+1.38	257.13	-1.02	N-acetylmuramoyl-L-alanine amidase
Smlt3331	382.29	260.09	-1.47	466.81	+1.22	tRNA (adenosine(37)-N6)-threonylcarbamoyltransferase complex ATPase subunit type 1 TsaE
Smlt3332	106.11	101.12	-1.05	116.19	+1.09	bifunctional ADP-dependent NAD(P)H-hydrate dehydratase/NAD(P)H-hydrate epimerase
Smlt3333	34.14	53.52	+1.57	36.68	+1.07	tRNA epoxyqueuosine(34) reductase QueG
Smlt3334	49.02	63.77	+1.3	54.90	+1.12	exodeoxyribonuclease VII large subunit
Smlt3335	0.00	0.00	0	0.00	0	penicillin binding protein
Smlt3336	99.94	90.80	-1.1	120.25	+1.2	M48 family metallopeptidase
Smlt3337	93.88	111.41	+1.19	129.39	+1.38	formylglycine-generating enzyme family protein

Smlt3338	60.11	44.60	-1.35	78.98	+1.31	ribonuclease D
Smlt3339	0.00	0.00	0	0.00	0	DUF4034 domain-containing protein
Smlt3340	112.56	115.17	+1.02	89.26	-1.26	TonB-dependent outer membrane receptor
Smlt3342	10.14	14.60	+1.44	26.94	+2.66	hypothetical protein
Smlt3344	80.83	75.49	-1.07	76.13	-1.06	hypothetical protein
Smlt3345	6.33	6.77	+1.07	8.14	+1.28	SDR family oxidoreductase
Smlt3346	16.99	23.98	+1.41	25.64	+1.51	methylated-DNA--[protein]-cysteine S-methyltransferase
Smlt3347	16.39	12.57	-1.3	28.49	+1.74	NADP-dependent oxidoreductase
Smlt3348	10.52	8.59	-1.22	21.15	+2.01	lipocalin family protein
Smlt3351	18.65	41.51	+2.23	24.90	+1.34	LEA type 2 family protein
Smlt3352	93.58	158.95	+1.7	100.77	+1.08	acyl-CoA dehydrogenase C-terminal domain-containing protein
Smlt3354	83.21	134.53	+1.62	125.99	+1.51	HNH endonuclease
Smlt3355	276.33	313.67	+1.14	310.91	+1.13	1-deoxy-D-xylulose-5-phosphate synthase
Smlt3357	806.81	436.76	-1.85	269.42	-2.99	hypothetical protein
Smlt3358	181.43	196.64	+1.08	102.36	-1.77	DUF3011 domain-containing protein
Smlt3359	8.01	16.08	+2.01	8.82	+1.1	hypothetical protein
Smlt3360	68.52	86.99	+1.27	102.68	+1.5	hypothetical protein
Smlt3361	37.38	35.91	-1.04	26.74	-1.4	DUF1653 domain-containing protein
Smlt3362	110.40	116.30	+1.05	73.32	-1.51	TetR/AcrR family transcriptional regulator
Smlt3363	86.94	70.95	-1.23	70.65	-1.23	multidrug transporter
Smlt3364	21.67	32.03	+1.48	36.47	+1.68	DUF1328 domain-containing protein
Smlt3365	35.00	51.32	+1.47	21.21	-1.65	LysE family translocator
Smlt3366	131.32	159.90	+1.22	187.55	+1.43	aspartate/glutamate racemase family protein
Smlt3367	115.90	68.07	-1.7	81.49	-1.42	hypothetical protein
Smlt3368	2.96	4.40	+1.49	5.97	+2.02	DUF3011 domain-containing protein
Smlt3369	14.26	23.61	+1.66	16.71	+1.17	hypothetical protein
Smlt3370	16.41	29.62	+1.8	31.21	+1.9	GNAT family N-acetyltransferase
Smlt3371	557.13	603.56	+1.08	507.23	-1.1	MerR family transcriptional regulator
Smlt3372	763.51	978.07	+1.28	956.79	+1.25	integration host factor subunit alpha

Smlt3373	136.99	173.65	+1.27	170.79	+1.25	phenylalanine--tRNA ligase subunit beta
Smlt3374	208.70	170.14	-1.23	224.80	+1.08	phenylalanine--tRNA ligase subunit alpha
Smlt3375	3716.81	2092.31	-1.78	1873.76	-1.98	50S ribosomal protein L20
Smlt3376	0.00	0.00	0	0.00	0	50S ribosomal protein L35
Smlt3377	2582.21	2106.18	-1.23	2054.48	-1.26	translation initiation factor IF-3
Smlt3378	222.54	268.04	+1.2	220.68	-1.01	threonine--tRNA ligase
Smlt3379	10.66	12.32	+1.16	8.29	-1.29	hypothetical protein
Smlt3380	0.00	0.00	0	0.00	0	hypothetical protein
Smlt3381	27.94	30.40	+1.09	31.22	+1.12	PepSY domain-containing protein
Smlt3382	32.42	38.95	+1.2	71.95	+2.22	VOC family protein
Smlt3383	31.02	35.97	+1.16	52.84	+1.7	glycoside hydrolase family 18 protein
Smlt3384	0.00	0.00	0	0.00	0	hypothetical protein
Smlt3385	1063.52	1036.82	-1.03	980.62	-1.08	polyribonucleotide nucleotidyltransferase
Smlt3386	3601.10	2534.68	-1.42	4009.84	+1.11	30S ribosomal protein S15
Smlt3387	63.78	34.23	-1.86	32.22	-1.98	tRNA pseudouridine(55) synthase TruB
Smlt3388	352.44	365.05	+1.04	339.83	-1.04	30S ribosome-binding factor RbfA
Smlt3389	684.63	941.31	+1.37	613.08	-1.12	translation initiation factor IF-2
Smlt3390	853.69	999.31	+1.17	710.65	-1.2	transcription termination factor NusA
Smlt3391	1175.59	820.11	-1.43	799.83	-1.47	ribosome maturation factor RimP
Smlt3392	270.78	242.01	-1.12	73.10	-3.7	NADH-quinone oxidoreductase subunit NuoN
Smlt3393	180.12	132.37	-1.36	52.63	-3.42	NADH-quinone oxidoreductase subunit M
Smlt3394	379.64	329.91	-1.15	130.14	-2.92	NADH-quinone oxidoreductase subunit L
Smlt3395	275.47	211.52	-1.3	90.23	-3.05	NADH-quinone oxidoreductase subunit NuoK
Smlt3396	153.66	115.86	-1.33	43.75	-3.51	NADH-quinone oxidoreductase subunit J
Smlt3397	154.34	123.17	-1.25	54.63	-2.83	NADH-quinone oxidoreductase subunit NuoI
Smlt3398	290.70	240.01	-1.21	77.21	-3.77	NADH-quinone oxidoreductase subunit NuoH
Smlt3399	470.56	392.85	-1.2	159.32	-2.95	NADH-quinone oxidoreductase subunit NuoG
Smlt3400	881.87	754.76	-1.17	308.79	-2.86	NADH-quinone oxidoreductase subunit NuoF
Smlt3401	1007.56	840.44	-1.2	299.28	-3.37	NADH-quinone oxidoreductase subunit NuoE

Smlt3402	604.61	549.34	-1.1	178.93	-3.38	NADH-quinone oxidoreductase subunit D
Smlt3403	568.99	462.00	-1.23	190.53	-2.99	NADH-quinone oxidoreductase subunit C
Smlt3404	725.57	438.15	-1.66	263.21	-2.76	NADH-quinone oxidoreductase subunit B
Smlt3405	778.67	409.21	-1.9	404.89	-1.92	NADH-quinone oxidoreductase subunit A
Smlt3406	659.46	587.67	-1.12	293.83	-2.24	preprotein translocase subunit SecG
Smlt3407	531.86	620.87	+1.17	552.10	+1.04	triose-phosphate isomerase
Smlt3409	240.76	301.81	+1.25	219.55	-1.1	isopenicillin N synthase family oxygenase
Smlt3410	38.32	43.79	+1.14	20.92	-1.83	DUF3413 domain-containing protein
Smlt3411	93.42	129.07	+1.38	64.82	-1.44	glycosyltransferase family 4 protein
Smlt3412	258.70	326.08	+1.26	240.80	-1.07	polysaccharide deacetylase family protein
Smlt3413	99.44	111.80	+1.12	77.01	-1.29	UDP-glucose 4-epimerase GalE
Smlt3414	210.63	205.05	-1.03	335.77	+1.59	phosphoglucosamine mutase
Smlt3415	402.46	294.78	-1.37	578.14	+1.44	acetyl-CoA carboxylase, carboxyltransferase subunit beta
Smlt3417	40.30	38.93	-1.04	42.03	+1.04	tryptophan synthase subunit alpha
Smlt3418	0.00	0.00	0	0.00	0	hypothetical protein
Smlt3419	37.82	44.67	+1.18	55.64	+1.47	tryptophan synthase subunit beta
Smlt3420	17.75	22.30	+1.26	15.27	-1.16	LysR family transcriptional regulator
Smlt3421	0.00	0.00	0	0.00	0	conserved hypothetical protein, pseudogene
Smlt3422	0.00	0.00	0	0.00	0	hypothetical protein
Smlt3423	179.25	212.86	+1.19	224.91	+1.25	phosphoribosylanthranilate isomerase
Smlt3424	126.34	116.21	-1.09	176.66	+1.4	tRNA pseudouridine(38-40) synthase TruA
Smlt3425	172.81	147.13	-1.17	187.81	+1.09	VOC family protein
Smlt3426	111.06	99.82	-1.11	150.52	+1.36	fimbrial protein FimV
Smlt3427	169.33	198.00	+1.17	124.99	-1.35	aspartate-semialdehyde dehydrogenase
Smlt3428	112.59	149.56	+1.33	145.65	+1.29	D-glycerate dehydrogenase
Smlt3429	235.41	253.05	+1.07	298.92	+1.27	chorismate synthase
Smlt3430	255.37	198.14	-1.29	272.19	+1.07	50S ribosomal protein L3 N(5)-glutamine methyltransferase
Smlt3431	22.93	36.55	+1.59	32.76	+1.43	SCO family protein
Smlt3432	76.47	106.38	+1.39	96.23	+1.26	archaetidylserine decarboxylase

Smlt3433	180.91	159.93	-1.13	232.12	+1.28	ribonuclease E inhibitor RraB
Smlt3434	180.81	146.17	-1.24	282.28	+1.56	transglycosylase SLT domain-containing protein
Smlt3435	202.61	215.94	+1.07	219.47	+1.08	DUF853 family protein
Smlt3436	120.75	106.66	-1.13	133.72	+1.11	transcription elongation factor GreB
Smlt3437	0.00	0.00	0	0.00	0	DUF3025 domain-containing protein
Smlt3438	84.81	59.25	-1.43	22.66	-3.74	30S ribosomal protein S12 methylthiotransferase RimO
Smlt3439	44.21	52.61	+1.19	104.25	+2.36	dienelactone hydrolase family protein
Smlt3440	134.57	221.26	+1.64	159.46	+1.18	HIT family protein
Smlt3441	30.97	49.52	+1.6	31.56	+1.02	DUF5668 domain-containing protein
Smlt3442	262.37	229.11	-1.15	283.92	+1.08	dCTP deaminase
Smlt3443	481.48	317.69	-1.52	249.78	-1.93	iron-sulfur cluster carrier protein ApbC
Smlt3444	0.00	0.00	0	0.00	0	TonB-dependent outer membrane receptor
Smlt3446	143.34	220.40	+1.54	92.24	-1.55	TonB-dependent outer membrane receptor
Smlt3447	240.24	296.81	+1.24	505.56	+2.1	peptidase
Smlt3449	0.00	0.00	0	0.00	0	TonB-dependent outer membrane receptor
Smlt3450	184.25	170.28	-1.08	220.71	+1.2	M13 family metallopeptidase
Smlt3451	28.48	19.50	-1.46	42.96	+1.51	VOC family protein
Smlt3452	45.74	72.79	+1.59	24.91	-1.84	NAD(P)/FAD-dependent oxidoreductase
Smlt3453	85.78	105.38	+1.23	98.29	+1.15	rhomboid family intramembrane serine protease
Smlt3454	87.41	95.95	+1.1	119.23	+1.36	MGMT family protein
Smlt3455	37.58	28.45	-1.32	34.04	-1.1	DMT family transporter
Smlt3456	55.70	59.26	+1.06	89.09	+1.6	glutathione-disulfide reductase
Smlt3457	15.19	15.03	-1.01	36.18	+2.38	FAD-dependent oxidoreductase
Smlt3458	38.27	22.72	-1.68	43.42	+1.13	DUF418 domain-containing protein
Smlt3459	377.14	187.92	-2.01	514.20	+1.36	peptidylprolyl isomerase
Smlt3460	35.55	40.52	+1.14	72.31	+2.03	C40 family peptidase
Smlt3461	217.35	107.40	-2.02	564.73	+2.6	C40 family peptidase
Smlt3462	64.78	52.35	-1.24	47.18	-1.37	dicarboxylate/amino acid:cation symporter
Smlt3463	31.01	29.49	-1.05	51.36	+1.66	alkaline phosphatase

Smlt3464	52.47	39.01	-1.35	71.16	+1.36	WG repeat-containing protein
Smlt3465	22.91	18.29	-1.25	27.44	+1.2	tRNA lysidine(34) synthetase TilS
Smlt3466	57.77	46.22	-1.25	60.39	+1.05	exodeoxyribonuclease VII small subunit
Smlt3467	196.51	138.84	-1.42	269.76	+1.37	polyprenyl synthetase family protein
Smlt3468	292.34	231.39	-1.26	205.13	-1.43	DUF4870 domain-containing protein
Smlt3469	85.37	197.10	+2.31	139.63	+1.64	metalloprotease PmbA
Smlt3470	328.13	341.97	+1.04	287.12	-1.14	ribosome-associated protein
Smlt3471	156.37	400.60	+2.56	178.77	+1.14	metalloprotease TldD
Smlt3472	50.05	84.90	+1.7	84.32	+1.68	TIGR02099 family protein
Smlt3473	193.25	206.14	+1.07	212.22	+1.1	ribonuclease G
Smlt3474	43.95	62.57	+1.42	34.06	-1.29	Maf family nucleotide pyrophosphatase
Smlt3475	44.24	95.03	+2.15	32.51	-1.36	SIMPL domain-containing protein
Smlt3477	111.30	153.35	+1.38	47.18	-2.36	TonB, energy transducer
Smlt3478	0.00	0.00	0	0.00	0	TonB-dependent outer membrane receptor
Smlt3479	19.35	25.27	+1.31	7.76	-2.49	basic amino acid/polyamine antiporter
Smlt3480	139.91	86.90	-1.61	80.58	-1.74	23S rRNA (pseudouridine(1915)-N(3))-methyltransferase RlmH
Smlt3481	202.34	109.91	-1.84	208.80	+1.03	ribosome silencing factor
Smlt3482	53.53	59.44	+1.11	83.63	+1.56	nicotinate-nucleotide adenylyltransferase
Smlt3483	153.48	210.71	+1.37	210.60	+1.37	DNA polymerase III subunit delta
Smlt3484	270.27	454.50	+1.68	385.57	+1.43	LPS assembly lipoprotein LptE
Smlt3485	436.60	328.11	-1.33	395.14	-1.1	leucine--tRNA ligase
Smlt3486	37.39	28.56	-1.31	48.29	+1.29	DUF998 domain-containing protein
Smlt3487	79.17	161.44	+2.04	52.51	-1.51	thioredoxin
Smlt3488	66.22	55.61	-1.19	37.88	-1.75	FecR domain-containing protein
Smlt3489	76.97	68.56	-1.12	41.39	-1.86	CHASE2 domain-containing protein
Smlt3490	17.31	14.51	-1.19	16.82	-1.03	DUF4442 domain-containing protein
Smlt3491	65.15	43.33	-1.5	53.02	-1.23	hypothetical protein
Smlt3492	55.36	46.02	-1.2	66.81	+1.21	DUF502 domain-containing protein
Smlt3493	27.05	24.45	-1.11	34.21	+1.26	queuosine precursor transporter

Smlt3494	50.34	88.81	+1.76	88.83	+1.76	DUF885 family protein
Smlt3495	17.46	25.79	+1.48	29.08	+1.67	serine hydrolase
Smlt3496	11.73	26.29	+2.24	18.60	+1.59	VOC family protein
Smlt3497	143.49	427.58	+2.98	378.74	+2.64	DUF2147 domain-containing protein
Smlt3498	121.80	103.02	-1.18	127.24	+1.04	methionine--tRNA ligase
Smlt3499	45.80	43.78	-1.05	54.37	+1.19	HAD-IB family hydrolase
Smlt3500	17.30	13.66	-1.27	6.37	-2.72	Rnf electron transport complex subunit RnfB
Smlt3501	699.58	449.43	-1.56	686.11	-1.02	RidA family protein
Smlt3502	18.69	13.56	-1.38	23.33	+1.25	bifunctional lysylphosphatidylglycerol flippase/synthetase MprF
Smlt3503	56.20	59.91	+1.07	65.09	+1.16	virulence factor family protein
Smlt3504	41.85	46.58	+1.11	60.85	+1.45	DUF998 domain-containing protein
Smlt3505	9.53	8.61	-1.11	7.83	-1.22	oxidoreductase-like protein
Smlt3506	128.79	232.47	+1.81	157.17	+1.22	hypothetical protein
Smlt3507	18.13	28.09	+1.55	23.39	+1.29	tetratricopeptide repeat protein
Smlt3508	595.31	369.66	-1.61	358.11	-1.66	alkylphosphonate utilization protein
Smlt3509	10.52	2.93	-3.59	5.19	-2.03	Hsp70 family protein
Smlt3510	170.54	299.10	+1.75	72.83	-2.34	YkgJ family cysteine cluster protein
Smlt3511	226.19	537.80	+2.38	59.69	-3.79	hypothetical protein
Smlt3512	31.71	109.23	+3.44	35.15	+1.11	DUF4097 family beta strand repeat-containing protein
Smlt3513	25.56	112.64	+4.41	38.52	+1.51	hypothetical protein
Smlt3514	32.03	158.05	+4.93	47.24	+1.47	sigma-70 family RNA polymerase sigma factor
Smlt3515	85.93	103.92	+1.21	156.82	+1.82	mechanosensitive ion channel family protein
Smlt3517	205.57	477.97	+2.33	189.25	-1.09	DksA/TraR family C4-type zinc finger protein
Smlt3518	208.49	199.34	-1.05	267.20	+1.28	DUF2058 domain-containing protein
Smlt3519	14.60	12.95	-1.13	33.08	+2.27	M48 family metallopeptidase
Smlt3520	72.27	40.94	-1.77	86.63	+1.2	RNA-binding protein
Smlt3521	31.85	32.54	+1.02	40.58	+1.27	phospholipase D family protein
Smlt3522	15.54	4.25	-3.66	11.59	-1.34	HDOD domain-containing protein
Smlt3523	247.89	202.07	-1.23	185.00	-1.34	Glu/Leu/Phe/Val dehydrogenase

Smlt3524	64.14	79.39	+1.24	64.29	+1.	autotransporter serine protease
Smlt3525	341.40	421.98	+1.24	470.86	+1.38	thiolase family protein
Smlt3526	9371.36	5328.49	-1.76	12908.38	+1.38	hypothetical protein
Smlt3527	66.96	81.78	+1.22	79.09	+1.18	hypothetical protein
Smlt3528	38.53	70.70	+1.84	58.67	+1.52	acyl-CoA dehydrogenase family protein
Smlt3529	11.02	23.48	+2.13	20.79	+1.89	ligase-associated DNA damage response exonuclease
Smlt3530	12.66	29.18	+2.3	38.87	+3.07	ATP-dependent DNA ligase
Smlt3531	7.23	11.90	+1.65	25.45	+3.52	ligase-associated DNA damage response DEXH box helicase
Smlt3532	2.23	8.94	+4.01	11.55	+5.18	ligase-associated DNA damage response endonuclease PdeM
Smlt3533	788.03	1086.75	+1.38	1318.17	+1.67	hypothetical protein
Smlt3534	54.84	64.38	+1.17	73.11	+1.33	FAD-dependent monooxygenase
Smlt3535	654.52	702.12	+1.07	778.08	+1.19	cold-shock protein
Smlt3536	162.30	129.63	-1.25	180.98	+1.12	S-methyl-5'-thioinosine phosphorylase
Smlt3537	130.01	105.08	-1.24	151.70	+1.17	hypoxanthine-guanine phosphoribosyltransferase
Smlt3538	127.23	119.09	-1.07	131.39	+1.03	beta-N-acetylhexosaminidase
Smlt3539	90.78	253.17	+2.79	57.24	-1.59	DsbA family oxidoreductase
Smlt3540	177.63	148.24	-1.2	177.00	-1.	CYTH domain-containing protein
Smlt3541	126.79	64.93	-1.95	25.78	-4.92	23S rRNA (uracil(1939)-C(5))-methyltransferase RlmD
Smlt3542	300.86	269.32	-1.12	275.82	-1.09	hybrid sensor histidine kinase/response regulator
Smlt3544	0.00	0.00	0	0.00	0	ISPsy9, transposase orfa
Smlt3545	0.00	0.00	0	0.00	0	ISPys9 like transposase
Smlt3546	0.00	0.00	0	0.00	0	conserved hypothetical protein
Smlt3547	66.50	66.09	-1.01	79.47	+1.2	DNA repair protein RecO
Smlt3548	380.09	444.07	+1.17	542.25	+1.43	GTPase Era
Smlt3549	251.76	287.69	+1.14	271.91	+1.08	ribonuclease III
Smlt3550	468.28	408.38	-1.15	365.03	-1.28	DUF4845 domain-containing protein
Smlt3551	339.23	454.06	+1.34	337.73	-1.	signal peptidase I
Smlt3552	469.31	439.66	-1.07	386.57	-1.21	translation elongation factor 4
Smlt3553	311.50	635.16	+2.04	307.16	-1.01	DegQ family serine endoprotease

Smlt3554	142.33	322.68	+2.27	127.32	-1.12	sigma-E factor negative regulatory protein
Smlt3555	322.10	736.15	+2.29	344.83	+1.07	RNA polymerase sigma factor RpoE
Smlt3556	114.45	116.85	+1.02	132.60	+1.16	enoyl-CoA hydratase/isomerase family protein
Smlt3558	30.19	38.32	+1.27	11.77	-2.57	pirin family protein
Smlt3559	2081.31	2109.66	+1.01	1084.26	-1.92	aquaporin Z
Smlt3560	549.13	792.78	+1.44	274.39	-2.	hypothetical protein
Smlt3561	273.00	347.13	+1.27	235.59	-1.16	pirin family protein
Smlt3562	71.27	167.70	+2.35	243.45	+3.42	carbon starvation protein A
Smlt3563	63.33	159.36	+2.52	166.63	+2.63	putative selenoprotein
Smlt3565	59.18	137.99	+2.33	35.97	-1.65	DUF819 family protein
Smlt3566	21.65	26.66	+1.23	23.70	+1.09	VOC family protein
Smlt3567	73.87	72.33	-1.02	68.66	-1.08	HAMP domain-containing histidine kinase
Smlt3568	100.32	142.73	+1.42	87.61	-1.15	response regulator transcription factor
Smlt3569	34.25	27.54	-1.24	21.78	-1.57	glycosyltransferase family 39 protein
Smlt3570	4.21	6.19	+1.47	2.92	-1.44	phosphatase PAP2 family protein
Smlt3571	10.42	8.02	-1.3	7.90	-1.32	phosphoethanolamine transferase
Smlt3573	0.00	0.00	0	0.00	0	IS110-like element ISStma7 family transposase
Smlt3574	95.62	78.20	-1.22	117.23	+1.23	M2 family metallopeptidase
Smlt3576	103.02	31.96	-3.22	161.16	+1.56	hypothetical protein
Smlt3577	10.84	31.63	+2.92	362.92	+33.47	siderophore-interacting protein
Smlt3578	28.57	21.97	-1.3	10.04	-2.85	multidrug effflux MFS transporter
Smlt3579	422.98	368.71	-1.15	417.59	-1.01	aminomethyl-transferring glycine dehydrogenase
Smlt3580	52.28	101.01	+1.93	33.06	-1.58	VOC family protein
Smlt3581	89.78	93.17	+1.04	73.22	-1.23	amino acid permease
Smlt3582	89.89	122.09	+1.36	120.34	+1.34	AraC family transcriptional regulator
Smlt3583	4.00	4.38	+1.09	15.89	+3.97	catalase
Smlt3584	18.70	31.96	+1.71	46.99	+2.51	hypothetical protein
Smlt3585	10.28	12.32	+1.2	14.21	+1.38	alpha/beta hydrolase
Smlt3586	42.17	37.28	-1.13	60.68	+1.44	hypothetical protein

Smlt3587	20.21	6.86	-2.95	12.55	-1.61	hypothetical protein
Smlt3588	67.81	5.28	-12.84	20.72	-3.27	methyl-accepting chemotaxis protein
Smlt3589	5.01	6.65	+1.33	10.86	+2.17	L,D-transpeptidase family protein
Smlt3590	4.34	4.54	+1.05	9.36	+2.16	M23 family metallopeptidase
Smlt3591	22.40	25.10	+1.12	28.19	+1.26	hypothetical protein
Smlt3592	348.00	284.26	-1.22	376.52	+1.08	adenylosuccinate synthase
Smlt3593	0.00	0.00	0	0.00	0	hypothetical protein
Smlt3594	9.32	7.60	-1.23	3.89	-2.39	DUF2065 family protein
Smlt3595	367.73	677.10	+1.84	423.06	+1.15	protease modulator HflC
Smlt3596	615.20	935.74	+1.52	618.35	+1.01	FtsH protease activity modulator HflK
Smlt3597	30.66	38.76	+1.26	40.93	+1.34	YhdH/YhfP family quinone oxidoreductase
Smlt3598	258.82	113.83	-2.27	122.59	-2.11	twitching motility response regulator PilH
Smlt3599	266.42	298.53	+1.12	192.05	-1.39	DnaJ domain-containing protein
Smlt3600	279.73	171.04	-1.64	27.65	-10.12	peroxiredoxin
Smlt3601	487.98	273.12	-1.79	13.78	-35.41	ferritin-like domain-containing protein
Smlt3602	19.43	9.64	-2.02	21.60	+1.11	penicillin-binding protein 1C
Smlt3603	127.15	78.19	-1.63	128.07	+1.01	alpha-2-macroglobulin family protein
Smlt3604	138.77	176.64	+1.27	127.97	-1.08	bifunctional 2-methylcitrate dehydratase/aconitate hydratase
Smlt3605	263.79	291.21	+1.1	233.73	-1.13	2-methylaconitate cis-trans isomerase PrpF
Smlt3606	0.00	0.00	0	0.00	0	type II toxin-antitoxin system RelE/ParE family toxin
Smlt3607	0.00	0.00	0	0.00	0	type II toxin-antitoxin system ParD family antitoxin
Smlt3608	326.54	625.46	+1.92	185.06	-1.76	Fe/S-dependent 2-methylisocitrate dehydratase AcnD
Smlt3609	698.62	1370.17	+1.96	626.55	-1.12	2-methylcitrate synthase
Smlt3610	406.50	1074.90	+2.64	369.23	-1.1	methylisocitrate lyase
Smlt3611	29.61	67.91	+2.29	19.08	-1.55	propionate catabolism operon regulatory protein PrpR
Smlt3612	0.00	0.00	0	0.00	0	hypothetical protein
Smlt3613	61.20	31.87	-1.92	18.50	-3.31	DoxX family protein
Smlt3614	40.60	39.82	-1.02	35.74	-1.14	hypothetical protein
Smlt3615	0.00	0.00	0	0.00	0	aminoglycoside N-acetyltransferase AAC(6')-Iz

Smlt3616	230.96	186.63	-1.24	249.02	+1.08	pyridoxamine 5'-phosphate oxidase
Smlt3617	146.88	195.02	+1.33	202.71	+1.38	hypothetical protein
Smlt3618	67.27	68.92	+1.02	158.78	+2.36	dodecin family protein
Smlt3619	112.33	133.91	+1.19	161.30	+1.44	shikimate kinase
Smlt3620	127.01	127.22	+1.	129.32	+1.02	3-dehydroquinate synthase
Smlt3621	21.36	28.77	+1.35	24.14	+1.13	WGR domain-containing protein
Smlt3622	86.95	102.32	+1.18	93.31	+1.07	uroporphyrinogen decarboxylase
Smlt3623	36.31	38.53	+1.06	45.23	+1.25	multidrug transporter subunit MdtD
Smlt3625	42.83	43.79	+1.02	53.38	+1.25	hybrid sensor histidine kinase/response regulator
Smlt3626	0.00	0.00	0	0.00	0	sensor histidine kinase/response regulator fusion protein, pseudogene
Smlt3627	7.74	8.02	+1.04	6.41	-1.21	YceI family protein
Smlt3628	6.84	10.74	+1.57	8.85	+1.29	cytochrome b
Smlt3629	99.48	125.30	+1.26	157.56	+1.58	YceI family protein
Smlt3630	0.00	0.00	0	0.00	0	glutaredoxin family protein
Smlt3631	287.68	275.69	-1.04	176.60	-1.63	L-serine ammonia-lyase
Smlt3632	63.36	81.69	+1.29	48.52	-1.31	alpha/beta hydrolase
Smlt3633	8.61	10.44	+1.21	1.71	-5.04	homoserine dehydrogenase
Smlt3634	10.98	12.99	+1.18	2.84	-3.87	O-succinylhomoserine (thiol)-lyase
Smlt3635	30.47	21.69	-1.4	8.96	-3.4	homoserine O-succinyltransferase
Smlt3636	14.10	15.75	+1.12	20.76	+1.47	M23 family metallopeptidase
Smlt3637	160.56	99.68	-1.61	117.84	-1.36	peptide chain release factor 3
Smlt3638	78.82	170.29	+2.16	65.88	-1.2	hemolysin III family protein
Smlt3639	28.88	43.96	+1.52	32.07	+1.11	AsmA family protein
Smlt3640	92.50	162.53	+1.76	62.51	-1.48	CBS domain-containing protein
Smlt3641	151.83	135.56	-1.12	143.13	-1.06	glycosyltransferase family 2 protein
Smlt3642	84.82	86.32	+1.02	116.44	+1.37	monofunctional biosynthetic peptidoglycan transglycosylase
Smlt3643	88.97	167.41	+1.88	46.67	-1.91	Hsp33 family molecular chaperone HslO
Smlt3644	337.29	537.12	+1.59	246.43	-1.37	hypothetical protein

Smlt3645	248.72	1716.43	+6.9	104.32	-2.38	TonB-dependent outer membrane receptor
Smlt3646	257.23	494.58	+1.92	360.76	+1.4	TetR/AcrR family transcriptional regulator
Smlt3647	58.15	174.27	+3.	30.58	-1.9	alpha/beta hydrolase
Smlt3648	109.84	310.14	+2.82	113.40	+1.03	acyl-CoA dehydrogenase
Smlt3649	19.98	63.11	+3.16	14.55	-1.37	DUF4442 domain-containing protein
Smlt3650	73.03	91.96	+1.26	118.45	+1.62	DUF1304 domain-containing protein
Smlt3651	18.41	13.80	-1.33	29.53	+1.6	MFS transporter
Smlt3652	167.15	147.92	-1.13	299.53	+1.79	serine hydrolase
Smlt3653	13.71	17.87	+1.3	12.98	-1.06	SDR family oxidoreductase
Smlt3654	15.77	23.45	+1.49	14.94	-1.06	LysR family transcriptional regulator
Smlt3655	0.00	0.00	0	0.00	0	hypothetical protein
Smlt3656	0.00	0.00	0	0.00	0	ABC transporter protein
Smlt3657	601.07	650.42	+1.08	682.92	+1.14	glycine cleavage system protein GcvH
Smlt3658	319.47	544.53	+1.7	383.18	+1.2	glycine cleavage system aminomethyltransferase GcvT
Smlt3659	0.00	0.00	0	0.00	0	NfeD family protein
Smlt3660	259.57	559.82	+2.16	259.37	-1.	SPFH/Band 7/PHB domain protein
Smlt3661	24.88	28.55	+1.15	31.94	+1.28	DUF962 domain-containing protein
Smlt3662	50.26	54.45	+1.08	73.75	+1.47	YnfA family protein
Smlt3663	122.25	131.23	+1.07	192.81	+1.58	nucleoside triphosphate pyrophosphohydrolase
Smlt3664	111.59	106.27	-1.05	148.47	+1.33	3'(2'),5'-bisphosphate nucleotidase CysQ
Smlt3665	259.88	223.62	-1.16	422.99	+1.63	ADP compounds hydrolase NudE
Smlt3666	51.94	54.21	+1.04	66.31	+1.28	adenosylmethionine--8-amino-7-oxononanoate transaminase
Smlt3667	94.70	102.47	+1.08	118.68	+1.25	16S rRNA (uracil(1498)-N(3))-methyltransferase
Smlt3668	155.98	81.58	-1.91	29.10	-5.36	chemotaxis protein CheW
Smlt3669	245.12	109.73	-2.23	54.43	-4.5	hypothetical protein
Smlt3670	313.13	228.78	-1.37	71.96	-4.35	Hpt domain-containing protein
Smlt3671	462.30	158.79	-2.91	84.62	-5.46	methyl-accepting chemotaxis protein
Smlt3672	447.83	89.93	-4.98	58.27	-7.68	chemotaxis protein CheW
Smlt3673	430.15	103.36	-4.16	67.92	-6.33	response regulator

Smlt3674	1372.32	505.78	-2.71	461.41	-2.97	twitching motility response regulator PilG
Smlt3675	90.59	71.70	-1.26	125.64	+1.39	glutathione synthase
Smlt3676	83.49	67.87	-1.23	114.19	+1.37	TonB, energy transducer
Smlt3677	133.90	96.24	-1.39	105.10	-1.27	tRNA (adenosine(37)-N6)-threonylcarbamoyltransferase complex dimerization subunit type 1 TsaB
Smlt3678	95.12	62.53	-1.52	86.85	-1.1	ATP-dependent DNA helicase
Smlt3680	149.11	255.93	+1.72	204.16	+1.37	hypothetical protein
Smlt3681	141.94	193.06	+1.36	186.64	+1.31	penicillin-binding protein 1B
Smlt3682	88.43	47.17	-1.87	101.56	+1.15	glycosyltransferase family 2 protein
Smlt3683	44.51	23.72	-1.88	45.13	+1.01	hypothetical protein
Smlt3684	34.72	39.37	+1.13	40.09	+1.15	hypothetical protein
Smlt3685	220.56	163.42	-1.35	325.43	+1.48	bifunctional (p)ppGpp synthetase/guanosine-3',5'-bis(diphosphate) 3'-pyrophosphohydrolase
Smlt3686	143.21	148.42	+1.04	237.99	+1.66	hypothetical protein
Smlt3687	78.72	89.46	+1.14	56.12	-1.4	ATP-dependent RNA helicase HrpA
Smlt3688	3700.03	1999.82	-1.85	4195.25	+1.13	DNA starvation/stationary phase protection protein
Smlt3689	144.40	113.69	-1.27	115.35	-1.25	DNA helicase RecQ
Smlt3690	29.29	61.36	+2.09	38.63	+1.32	CopL family metal-binding regulatory protein
Smlt3691	5.21	7.77	+1.49	9.26	+1.78	copper resistance protein/multicopper oxidase
Smlt3692	12.08	17.86	+1.48	17.68	+1.46	copper resistance protein B
Smlt3693	24.84	15.57	-1.6	41.45	+1.67	GNAT family N-acetyltransferase
Smlt3694	1.77	2.71	+1.53	2.90	+1.64	CPBP family intramembrane metalloprotease
Smlt3695	39.93	11.80	-3.38	38.56	-1.04	response regulator
Smlt3696	42.64	59.18	+1.39	87.69	+2.06	prolyl oligopeptidase family serine peptidase
Smlt3697	22.09	22.99	+1.04	48.49	+2.2	alpha/beta hydrolase
Smlt3698	231.97	241.73	+1.04	424.85	+1.83	hypothetical protein
Smlt3699	6.93	16.78	+2.42	7.66	+1.1	DUF2268 domain-containing putative Zn-dependent protease
Smlt3700	110.60	79.45	-1.39	83.79	-1.32	7-cyano-7-deazaguanine synthase QueC
Smlt3701	95.80	34.49	-2.78	38.22	-2.51	7-carboxy-7-deazaguanine synthase QueE

Smlt3702	377.81	486.46	+1.29	282.76	-1.34	tol-pal system protein YbgF
Smlt3703	1181.35	1880.58	+1.59	1361.29	+1.15	peptidoglycan-associated lipoprotein Pal
Smlt3704	203.51	190.84	-1.07	200.44	-1.02	Tol-Pal system beta propeller repeat protein TolB
Smlt3705	214.34	230.75	+1.08	243.88	+1.14	cell envelope integrity protein TolA
Smlt3706	104.22	71.75	-1.45	74.43	-1.4	protein TolR
Smlt3707	374.47	341.62	-1.1	283.71	-1.32	protein TolQ
Smlt3708	196.52	266.33	+1.36	212.94	+1.08	tol-pal system-associated acyl-CoA thioesterase
Smlt3709	167.38	144.73	-1.16	162.48	-1.03	Holliday junction branch migration DNA helicase RuvB
Smlt3710	111.25	91.64	-1.21	139.46	+1.25	potassium transporter Kup
Smlt3711	134.29	90.20	-1.49	171.99	+1.28	Holliday junction branch migration protein RuvA
Smlt3712	300.72	156.51	-1.92	483.00	+1.61	crossover junction endodeoxyribonuclease RuvC
Smlt3713	980.66	472.56	-2.08	1469.99	+1.5	YebC/PmpR family DNA-binding transcriptional regulator
Smlt3715	86.35	67.16	-1.29	71.77	-1.2	alpha/beta fold hydrolase
Smlt3716	167.55	175.47	+1.05	208.42	+1.24	GNAT family N-acetyltransferase
Smlt3718	239.18	144.50	-1.66	322.16	+1.35	aspartate--tRNA ligase
Smlt3719	22.27	35.06	+1.57	31.88	+1.43	DUF3011 domain-containing protein
Smlt3720	103.93	242.19	+2.33	134.22	+1.29	hypothetical protein
Smlt3721	29.04	17.19	-1.69	41.80	+1.44	cation:proton antiporter
Smlt3722	17.94	60.66	+3.38	105.31	+5.87	L2 family class A beta-lactamase
Smlt3723	6.78	8.83	+1.3	14.00	+2.06	LysR family transcriptional regulator AmpR
Smlt3724	183.44	208.25	+1.14	141.53	-1.3	ABC-F family ATP-binding cassette domain-containing protein
Smlt3725	14.62	26.13	+1.79	17.02	+1.16	TonB-dependent outer membrane receptor
Smlt3726	194.75	333.36	+1.71	121.52	-1.6	Kef family K(+) transporter
Smlt3728	7.09	9.11	+1.29	6.89	-1.03	aminotransferase class V-fold PLP-dependent enzyme
Smlt3729	0.00	0.00	0	0.00	0	conserved hypothetical protein
Smlt3730	10.70	8.25	-1.3	6.15	-1.74	two-component sensor histidine kinase
Smlt3731	4.05	2.70	-1.5	2.31	-1.75	MipA/OmpV family protein
Smlt3732	703.11	820.99	+1.17	143.63	-4.9	ATP-dependent chaperone ClpB
Smlt3733	88.51	139.06	+1.57	55.90	-1.58	DUF3014 domain-containing protein

Smlt3734	17.07	28.55	+1.67	18.58	+1.09	glutathione S-transferase
Smlt3735	11.51	11.56	+1.	18.29	+1.59	membrane protein
Smlt3737	92.50	84.40	-1.1	110.20	+1.19	pyruvate oxidase
Smlt3738	0.00	0.00	0	0.00	0	SH3 domain-containing protein
Smlt3739	57.13	70.96	+1.24	33.41	-1.71	DUF3574 domain-containing protein
Smlt3740	39.37	103.75	+2.64	8.92	-4.41	TonB-dependent outer membrane receptor
Smlt3741	185.06	146.44	-1.26	105.70	-1.75	trehalose-phosphatase
Smlt3742	160.02	195.47	+1.22	200.13	+1.25	glycoside hydrolase family 15 protein
Smlt3743	137.70	166.93	+1.21	286.80	+2.08	alpha,alpha-trehalose-phosphate synthase (UDP-forming)
Smlt3744	29.95	29.06	-1.03	35.92	+1.2	thiol-disulfide oxidoreductase DCC family protein
Smlt3745	13.79	22.50	+1.63	23.95	+1.74	DUF4166 domain-containing protein
Smlt3746	27.19	49.53	+1.82	58.17	+2.14	peptidoglycan editing factor PgeF
Smlt3747	70.90	96.30	+1.36	86.01	+1.21	23S rRNA pseudouridine(1911/1915/1917) synthase RluD
Smlt3748	134.57	132.94	-1.01	183.79	+1.37	outer membrane protein assembly factor BamD
Smlt3749	0.88	0.90	+1.02	0.87	-1.01	lipase
Smlt3751	245.65	284.13	+1.16	244.07	-1.01	NAD+ synthase
Smlt3752	1392.42	1503.65	+1.08	2971.30	+2.13	succinate--CoA ligase subunit alpha
Smlt3753	2234.75	2401.29	+1.07	4750.23	+2.13	ADP-forming succinate--CoA ligase subunit beta
Smlt3754	86.00	40.51	-2.12	20.70	-4.16	PAS domain-containing protein
Smlt3755	132.03	77.14	-1.71	103.96	-1.27	sigma-54 dependent transcriptional regulator
Smlt3756	333.59	206.78	-1.61	132.57	-2.52	type IV-A pilus assembly ATPase PilB
Smlt3757	0.00	0.00	0	0.00	0	pilin
Smlt3758	0.00	0.00	0	0.00	0	pilin
Smlt3759	492.23	143.34	-3.43	87.17	-5.65	type II secretion system F family protein
Smlt3760	496.53	203.55	-2.44	193.69	-2.56	A24 family peptidase
Smlt3761	193.79	94.20	-2.06	111.58	-1.74	dephospho-CoA kinase
Smlt3763	0.00	0.00	0	0.00	0	conserved hypothetical protein
Smlt3764	0.00	0.00	0	0.00	0	hypothetical protein
Smlt3765	0.00	0.00	0	0.00	0	HAMP domain-containing histidine kinase

Smlt3766	484.40	581.49	+1.2	492.63	+1.02	response regulator transcription factor
Smlt3767	174.16	232.80	+1.34	415.40	+2.39	hypothetical protein
Smlt3768	87.25	100.46	+1.15	198.68	+2.28	hypothetical protein
Smlt3769	54.55	41.83	-1.3	69.48	+1.27	30S ribosomal protein S6--L-glutamate ligase
Smlt3770	56.98	90.41	+1.59	84.74	+1.49	acyl-CoA thioesterase
Smlt3771	0.00	0.00	0	0.00	0	hypothetical protein
Smlt3772	0.00	0.00	0	0.00	0	hypothetical protein
Smlt3773	403.34	525.67	+1.3	551.91	+1.37	autotransporter domain-containing esterase
Smlt3774	55.14	93.13	+1.69	33.44	-1.65	sulfur carrier protein ThiS
Smlt3775	140.55	131.02	-1.07	146.51	+1.04	thiazole synthase
Smlt3776	272.35	242.90	-1.12	293.59	+1.08	tRNA (guanosine(46)-N7)-methyltransferase TrmB
Smlt3777	83.43	92.20	+1.11	103.43	+1.24	SLC13 family permease
Smlt3778	0.00	0.00	0	0.00	0	hypothetical protein
Smlt3779	112.87	120.58	+1.07	45.87	-2.46	Rieske (2Fe-2S) protein
Smlt3780	80.74	121.84	+1.51	70.58	-1.14	hypothetical protein
Smlt3781	70.24	104.08	+1.48	138.16	+1.97	fumarylacetoacetate hydrolase family protein
Smlt3782	132.55	174.19	+1.31	137.28	+1.04	large-conductance mechanosensitive channel protein MscL
Smlt3783	60.28	98.29	+1.63	122.58	+2.03	M28 family peptidase
Smlt3784	81.27	144.14	+1.77	106.55	+1.31	rRNA methyltransferase
Smlt3785	37.47	58.42	+1.56	34.08	-1.1	alanine:cation symporter family protein
Smlt3786	0.00	0.00	0	0.00	0	GNAT family N-acetyltransferase
Smlt3787	8.49	14.08	+1.66	37.58	+4.43	efflux RND transporter permease subunit
Smlt3788	20.43	26.45	+1.29	67.71	+3.31	efflux RND transporter periplasmic adaptor subunit
Smlt3789	31.01	146.11	+4.71	89.63	+2.89	TonB-dependent copper outer membrane receptor
Smlt3790	0.00	0.00	0	0.00	0	DUF2946 domain-containing protein
Smlt3793	31.41	60.87	+1.94	44.43	+1.41	LysR family transcriptional regulator
Smlt3794	52.07	68.20	+1.31	62.29	+1.2	cysteine synthase A
Smlt3795	214.50	403.07	+1.88	95.42	-2.25	O-acetyl-ADP-ribose deacetylase
Smlt3796	294.54	150.34	-1.96	195.78	-1.5	hypothetical protein

Smlt3797	1069.20	1369.91	+1.28	1440.59	+1.35	fructose-bisphosphate aldolase class I
Smlt3798	254.76	134.57	-1.89	261.05	+1.02	pyruvate kinase
Smlt3799	299.62	297.41	-1.01	457.94	+1.53	HAD hydrolase-like protein
Smlt3800	241.96	225.88	-1.07	280.99	+1.16	phosphoglycerate kinase
Smlt3802	126.75	134.97	+1.06	129.93	+1.03	DUF3999 domain-containing protein
Smlt3803	43.20	35.30	-1.22	46.51	+1.08	DUF2339 domain-containing protein
Smlt3804	2139.74	1950.28	-1.1	2510.05	+1.17	type I glyceraldehyde-3-phosphate dehydrogenase
Smlt3805	23046.99	9695.03	-2.38	3755.01	-6.14	outer membrane beta-barrel protein
Smlt3806	133.85	105.64	-1.27	123.53	-1.08	S1/P1 nuclease
Smlt3807	368.98	311.69	-1.18	235.33	-1.57	MBL fold metallo-hydrolase
Smlt3808	71.65	83.19	+1.16	31.08	-2.31	hypothetical protein
Smlt3809	85.45	100.73	+1.18	43.87	-1.95	TonB family protein
Smlt3810	48.41	67.96	+1.4	37.84	-1.28	BlaI/MecI/CopY family transcriptional regulator
Smlt3811	15.00	33.99	+2.27	11.79	-1.27	flavin reductase family protein
Smlt3812	81.26	138.40	+1.7	82.32	+1.01	acetyl-CoA hydrolase
Smlt3813	223.58	260.41	+1.16	267.52	+1.2	transketolase
Smlt3814	164.67	155.48	-1.06	168.57	+1.02	dicarboxylate/amino acid:cation symporter
Smlt3815	41.39	46.40	+1.12	46.31	+1.12	BatD family protein
Smlt3816	0.00	0.00	0	0.00	0	VWA domain-containing protein
Smlt3817	53.26	64.90	+1.22	60.74	+1.14	VWA domain-containing protein
Smlt3818	56.13	65.63	+1.17	56.01	-1.	DUF4381 family protein
Smlt3819	47.91	41.51	-1.15	44.36	-1.08	DUF58 domain-containing protein
Smlt3820	106.92	74.35	-1.44	68.62	-1.56	MoxR family ATPase
Smlt3821	478.31	162.67	-2.94	53.66	-8.91	type IV pilus secretin PilQ family protein
Smlt3822	828.01	316.93	-2.61	108.19	-7.65	pilus assembly protein PilP
Smlt3823	241.11	86.74	-2.78	26.28	-9.17	type 4a pilus biogenesis protein PilO
Smlt3824	0.00	0.00	0	0.00	0	PilN domain-containing protein
Smlt3825	879.76	196.07	-4.49	85.30	-10.31	pilus assembly protein PilM
Smlt3826	92.04	92.46	+1.	100.15	+1.09	penicillin-binding protein 1A

Smlt3827	93.28	134.27	+1.44	101.16	+1.08	hypothetical protein
Smlt3828	0.00	0.00	0	0.00	0	hypothetical protein
Smlt3829	0.00	0.00	0	0.00	0	YadA-like family protein
Smlt3830	0.00	0.00	0	0.00	0	giant cable pilus chaperone protein
Smlt3831	0.00	0.00	0	0.00	0	minor pilin and initiator protein
Smlt3832	0.00	0.00	0	0.00	0	outer membrane usher (colonisation factor antigen I subunit c)
Smlt3833	0.00	0.00	0	0.00	0	CS1 type fimbrial major subunit
Smlt3834	0.00	0.00	0	0.00	0	membrane protein
Smlt3835	701.81	643.35	-1.09	1095.09	+1.56	citrate synthase
Smlt3836	1592.62	917.86	-1.74	579.34	-2.75	type B 50S ribosomal protein L31
Smlt3837	131.76	89.93	-1.47	144.25	+1.09	nucleoside hydrolase
Smlt3838	100.46	78.15	-1.29	99.49	-1.01	ATP-dependent DNA helicase RecG
Smlt3839	184.89	127.05	-1.46	194.58	+1.05	RidA family protein
Smlt3840	118.58	106.62	-1.11	136.60	+1.15	bifunctional (p)ppGpp synthetase/guanosine-3',5'-bis(diphosphate) 3'-pyrophosphohydrolase
Smlt3841	2706.71	2155.81	-1.26	2013.83	-1.34	DNA-directed RNA polymerase subunit omega
Smlt3842	162.23	109.18	-1.49	164.09	+1.01	guanylate kinase
Smlt3843	0.00	0.00	0	0.00	0	formate dehydrogenase-N subunit alpha
Smlt3845	89.36	128.41	+1.44	8.33	-10.72	formate dehydrogenase subunit beta
Smlt3846	105.18	139.04	+1.32	8.63	-12.19	formate dehydrogenase subunit gamma
Smlt3848	125.35	184.82	+1.47	170.86	+1.36	formate dehydrogenase accessory protein FdhE
Smlt3849	75.08	106.55	+1.42	101.29	+1.35	L-seryl-tRNA(Sec) selenium transferase
Smlt3850	92.14	139.09	+1.51	121.88	+1.32	selenocysteine-specific translation elongation factor
Smlt3851	229.33	195.73	-1.17	197.47	-1.16	selenide, water dikinase SelD
Smlt3852	0.00	0.00	0	0.00	0	helix-turn-helix domain-containing protein
Smlt3853	0.00	0.00	0	0.00	0	nucleotidyl transferase AbiEii/AbiGii toxin family protein
Smlt3854	160.69	113.15	-1.42	155.10	-1.04	YicC family protein
Smlt3855	120.26	69.44	-1.73	72.75	-1.65	ribonuclease PH
Smlt3856	85.84	63.57	-1.35	67.75	-1.27	VOC family protein

Smlt3857	79.31	52.40	-1.51	60.42	-1.31	RdgB/HAM1 family non-canonical purine NTP pyrophosphatase
Smlt3858	50.69	33.49	-1.51	29.85	-1.7	radical SAM family heme chaperone HemW
Smlt3859	140.57	134.13	-1.05	173.65	+1.24	DUF1631 family protein
Smlt3860	111.18	104.93	-1.06	115.97	+1.04	PilZ domain-containing protein
Smlt3861	279.17	145.03	-1.92	448.41	+1.61	Xaa-Pro dipeptidase
Smlt3862	0.00	0.00	0	0.00	0	transposon-related protein
Smlt3863	243.17	220.84	-1.1	249.90	+1.03	aminopeptidase P N-terminal domain-containing protein
Smlt3864	89.13	73.19	-1.22	83.54	-1.07	YecA family protein
Smlt3865	168.31	244.54	+1.45	286.80	+1.7	cell division protein ZapA
Smlt3866	38.97	48.88	+1.25	41.46	+1.06	5-formyltetrahydrofolate cyclo-ligase
Smlt3867	66.91	40.56	-1.65	72.13	+1.08	EVE domain-containing protein
Smlt3868	165.45	145.72	-1.14	182.24	+1.1	ribose-5-phosphate isomerase RpiA
Smlt3869	247.41	250.89	+1.01	351.20	+1.42	DUF192 domain-containing protein
Smlt3870	139.65	222.73	+1.59	182.91	+1.31	SirB1 family protein
Smlt3871	150.01	109.19	-1.37	154.67	+1.03	rubredoxin
Smlt3872	34.51	43.26	+1.25	47.23	+1.37	thiamine phosphate synthase
Smlt3873	274.41	202.31	-1.36	276.01	+1.01	glutamate-1-semialdehyde 2,1-aminomutase
Smlt3873A	876.77	367.90	-2.38	1257.11	+1.43	azurin
Smlt3875	18.38	22.35	+1.22	32.96	+1.79	HAD-IA family hydrolase
Smlt3876	350.86	221.78	-1.58	456.70	+1.3	acetylornithine transaminase
Smlt3877	22.97	41.83	+1.82	38.10	+1.66	ion transporter
Smlt3878	269.62	36.36	-7.42	119.71	-2.25	YiiG family protein
Smlt3879	110.75	150.24	+1.36	138.07	+1.25	NAD(P)-dependent oxidoreductase
Smlt3880	206.13	348.34	+1.69	234.79	+1.14	hypothetical protein
Smlt3881	30.78	40.69	+1.32	17.03	-1.81	DUF2867 domain-containing protein
Smlt3882	90.18	171.41	+1.9	76.43	-1.18	serine/threonine-protein kinase
Smlt3883	150.94	287.72	+1.91	147.46	-1.02	bifunctional DedA family/phosphatase PAP2 family protein
Smlt3884	104.17	127.02	+1.22	135.31	+1.3	LON peptidase substrate-binding domain-containing protein

Smlt3885	142.95	146.27	+1.02	183.90	+1.29	UDP-N-acetylmuramate:L-alanyl-gamma-D-glutamyl-meso-diaminopimelate ligase
Smlt3886	1978.74	1345.10	-1.47	1699.91	-1.16	adenylate kinase
Smlt3887	64.30	118.20	+1.84	157.87	+2.46	6-phosphofructokinase
Smlt3888	33.83	58.66	+1.73	77.86	+2.3	DUF488 family protein
Smlt3889	153.93	357.04	+2.32	96.29	-1.6	hypothetical protein
Smlt3891	53.09	77.78	+1.47	35.82	-1.48	sodium-translocating pyrophosphatase
Smlt3892	1.99	11.90	+5.98	34.57	+17.37	TonB, energy transducer
Smlt3893	7.43	20.31	+2.73	77.33	+10.4	ExbD, bipolymerase transportor
Smlt3894	6.52	24.08	+3.7	95.07	+14.59	ExbB, TonB-system energizer
Smlt3895	3.38	15.11	+4.47	96.14	+28.42	YbaN family protein
Smlt3896	5.51	19.38	+3.52	67.21	+12.19	heme oxygenase
Smlt3897	480.14	488.20	+1.02	559.36	+1.17	DUF2789 domain-containing protein
Smlt3898	2.22	14.91	+6.71	92.66	+41.71	FecA-like TonB-dependent outer membrane receptor
Smlt3899	9.67	13.69	+1.42	56.21	+5.82	FecR-like protein
Smlt3900	9.03	19.59	+2.17	630.59	+69.84	FecI-like RNA polymerase sigma factor
Smlt3900A	0.95	0.70	-1.36	1.04	+1.09	hypothetical protein
Smlt3902	915.84	631.46	-1.45	662.44	-1.38	inorganic diphosphatase
Smlt3903	124.11	55.31	-2.24	53.17	-2.33	HDOD domain-containing protein
Smlt3904	12.02	5.22	-2.3	8.68	-1.39	hypothetical protein
Smlt3905	0.00	0.00	0	0.00	0	TonB-dependent outer membrane receptor
Smlt3906	184.97	37.44	-4.94	225.80	+1.22	helix-turn-helix transcriptional regulator
Smlt3907	240.94	260.79	+1.08	151.73	-1.59	winged helix-turn-helix domain-containing protein
Smlt3908	23.34	24.88	+1.07	18.24	-1.28	ion channel
Smlt3909	40.70	36.30	-1.12	25.82	-1.58	phosphomethylpyrimidine synthase ThiC
Smlt3910	0.00	0.00	0	0.00	0	BCCT family transporter
Smlt3911	0.00	0.00	0	0.00	0	hypothetical protein
Smlt3912	14.21	29.33	+2.06	26.48	+1.86	gamma-glutamyltransferase
Smlt3913	7.04	30.97	+4.4	6.54	-1.08	ketol-acid reductoisomerase

Smlt3914	2.52	9.72	+3.86	2.79	+1.11	acetolactate synthase 2 catalytic subunit
Smlt3915	4.49	9.57	+2.13	1.95	-2.3	acetolactate synthase
Smlt3916	2.97	8.15	+2.75	1.80	-1.65	threonine dehydratase
Smlt3917	6.51	22.70	+3.49	6.70	+1.03	2-isopropylmalate synthase
Smlt3918	2.22	5.76	+2.59	2.45	+1.1	class I SAM-dependent methyltransferase
Smlt3919	16.31	26.79	+1.64	11.64	-1.4	3-isopropylmalate dehydratase large subunit
Smlt3920	11.59	20.55	+1.77	9.26	-1.25	3-isopropylmalate dehydratase small subunit
Smlt3921	30.60	40.19	+1.31	47.55	+1.55	3-isopropylmalate dehydrogenase
Smlt3922	7.21	10.21	+1.42	6.96	-1.04	SDR family oxidoreductase
Smlt3923	62.08	67.58	+1.09	51.17	-1.21	LysR family transcriptional regulator
Smlt3924	73.27	102.50	+1.4	59.82	-1.22	efflux RND transporter permease subunit
Smlt3925	65.86	181.39	+2.75	63.71	-1.03	efflux RND transporter periplasmic adaptor subunit
Smlt3926	216.00	427.33	+1.98	174.43	-1.24	TetR/AcrR family transcriptional regulator
Smlt3927	492.05	574.65	+1.17	474.39	-1.04	protein-L-isoaspartate O-methyltransferase
Smlt3928	510.41	738.51	+1.45	760.42	+1.49	TolC family outer membrane protein
Smlt3929	9.66	10.04	+1.04	7.45	-1.3	hypothetical protein
Smlt3930	92.75	84.60	-1.1	121.85	+1.31	lipid IV(A) 3-deoxy-D-manno-octulosonic acid transferase
Smlt3931	176.50	265.08	+1.5	199.59	+1.13	LpxL/LpxP family Kdo(2)-lipid IV(A) lauroyl/palmitoleoyl acyltransferase
Smlt3932	27.40	34.23	+1.25	22.27	-1.23	O-antigen ligase family protein
Smlt3933	112.08	64.85	-1.73	67.68	-1.66	glycosyltransferase
Smlt3934	425.72	303.78	-1.4	346.65	-1.23	zinc-finger domain-containing protein
Smlt3935	40.71	52.95	+1.3	95.29	+2.34	GH92 family glycosyl hydrolase
Smlt3936	0.00	0.00	0	0.00	0	hypothetical protein
Smlt3938	0.00	0.00	0	0.00	0	IS481-like element ISStma1 family transposase
Smlt3940	699.45	929.17	+1.33	807.43	+1.15	NADP-dependent malic enzyme
Smlt3942	196.01	262.33	+1.34	98.04	-2.	dicarboxylate/amino acid:cation symporter
Smlt3943	273.62	304.33	+1.11	146.65	-1.87	porin
Smlt3944	35.80	32.39	-1.11	24.02	-1.49	PAS-domain containing protein

Smlt3946	97.29	104.02	+1.07	127.60	+1.31	response regulator transcription factor
Smlt3947	14.64	16.54	+1.13	32.59	+2.23	ABC transporter substrate-binding protein
Smlt3948	11.65	9.14	-1.27	23.06	+1.98	sensor histidine kinase
Smlt3949	38.57	28.21	-1.37	61.47	+1.59	response regulator transcription factor
Smlt3950	3.51	4.96	+1.42	666.64	+190.18	porin
Smlt3951	2.89	6.64	+2.29	182.97	+63.24	CitMHS family transporter
Smlt3952	0.00	0.00	0	0.00	0	acetoacetyl-CoA reductase
Smlt3953	10.69	11.49	+1.07	19.37	+1.81	hypothetical protein
Smlt3954	1.97	1.59	-1.24	3.87	+1.97	TonB-dependent outer membrane receptor
Smlt3955	58.86	53.60	-1.1	78.90	+1.34	flavohemoglobin expression-modulating QEGLA motif protein
Smlt3956	192.90	207.76	+1.08	271.51	+1.41	ribosome small subunit-dependent GTPase A
Smlt3957	127.67	192.09	+1.5	220.65	+1.73	pyridoxal phosphate-dependent aminotransferase
Smlt3958	145.91	192.37	+1.32	292.12	+2.	SGNH/GDSL hydrolase family protein
Smlt3959	18.74	17.91	-1.05	40.56	+2.16	class I SAM-dependent methyltransferase
Smlt3960	123.15	231.55	+1.88	120.24	-1.02	polysaccharide deacetylase family protein
Smlt3961	938.67	925.62	-1.01	980.37	+1.04	Grx4 family monothiol glutaredoxin
Smlt3962	126.64	79.96	-1.58	173.68	+1.37	DUF924 family protein
Smlt3963	184.66	70.13	-2.63	169.10	-1.09	AMP nucleosidase
Smlt3964	41.17	44.72	+1.09	67.25	+1.63	amidohydrolase
Smlt3965	36.00	60.00	+1.67	58.17	+1.62	amidohydrolase family protein
Smlt3966	18.22	32.99	+1.81	26.82	+1.47	2-hydroxychromene-2-carboxylate isomerase
Smlt3967	129.82	132.12	+1.02	205.86	+1.59	DegV family protein
Smlt3968	583.37	794.87	+1.36	942.01	+1.61	hypothetical protein
Smlt3969	2.83	3.00	+1.06	2.67	-1.06	efflux transporter outer membrane subunit
Smlt3970	1.18	2.15	+1.82	1.87	+1.58	HlyD family secretion protein
Smlt3971	4.39	3.59	-1.22	4.09	-1.07	MFS transporter
Smlt3972	41.23	36.52	-1.13	49.71	+1.21	LysR family transcriptional regulator
Smlt3973	1.69	1.12	-1.51	1.43	-1.18	universal stress protein
Smlt3974	1.12	1.59	+1.42	0.25	-4.55	hypothetical protein

Smlt3975	10.17	12.20	+1.2	10.14	-1.	GGDEF domain-containing protein
Smlt3976	66.96	92.50	+1.38	60.04	-1.12	S-formylglutathione hydrolase
Smlt3977	50.89	83.89	+1.65	72.03	+1.42	YqcC family protein
Smlt3978	120.84	158.89	+1.31	116.78	-1.03	S-(hydroxymethyl)glutathione dehydrogenase/class III alcohol dehydrogenase
Smlt3979	24.31	35.57	+1.46	16.27	-1.49	formaldehyde-responsive transcriptional repressor FrmR
Smlt3980	45.94	51.24	+1.12	46.59	+1.01	LysR family transcriptional regulator
Smlt3981	9.90	4.37	-2.27	3.07	-3.23	alkene reductase
Smlt3982	53.27	17.30	-3.08	69.83	+1.31	SMI1/KNR4 family protein
Smlt3983	21.52	23.54	+1.09	29.81	+1.39	VOC family protein
Smlt3984	1.19	2.03	+1.71	2.00	+1.68	hypothetical protein
Smlt3985	63.68	181.32	+2.85	56.55	-1.13	TetR/AcrR family transcriptional regulator
Smlt3986	32.88	49.73	+1.51	25.11	-1.31	LysR family transcriptional regulator
Smlt3987	43.93	62.77	+1.43	45.25	+1.03	NAD(P)-dependent alcohol dehydrogenase
Smlt3988	67.22	58.81	-1.14	97.49	+1.45	glutamate--cysteine ligase
Smlt3989	90.58	145.74	+1.61	125.02	+1.38	VIT family protein
Smlt3990	49.72	50.22	+1.01	57.81	+1.16	helix-turn-helix transcriptional regulator
Smlt3991	6.44	4.45	-1.45	6.35	-1.01	MBL fold metallo-hydrolase
Smlt3992	217.69	441.90	+2.03	265.23	+1.22	ribosome biogenesis GTP-binding protein YihA/YsxC
Smlt3992A	137.99	201.70	+1.46	151.34	+1.1	cytochrome c4
Smlt3993	52.46	56.87	+1.08	41.88	-1.25	thiol:disulfide interchange protein DsbA/DsbL
Smlt3994	144.67	224.63	+1.55	164.35	+1.14	thiol:disulfide interchange protein DsbA/DsbL
Smlt3995	106.96	144.07	+1.35	127.91	+1.2	endonuclease/exonuclease/phosphatase family protein
Smlt3996	7.28	10.42	+1.43	5.53	-1.32	EamA family transporter
Smlt3997	16.70	12.23	-1.37	43.49	+2.6	Lrp/AsnC family transcriptional regulator
Smlt3998	11.06	7.99	-1.39	15.43	+1.39	M23 family metallopeptidase
Smlt3999	3.07	47.07	+15.32	1314.56	+427.96	TonB-dependent outer membrane receptor
Smlt3999A	0.00	0.00	0	0.00	0	hypothetical protein
Smlt4003	6.32	30.26	+4.79	5.64	-1.12	TonB-dependent outer membrane receptor

Smlt4005	0.00	0.00	0	0.00	0	hypothetical protein
Smlt4006	0.00	0.00	0	0.00	0	hypothetical protein
Smlt4007	106.91	218.04	+2.04	126.80	+1.19	transglycosylase SLT domain-containing protein
Smlt4008	86.20	110.86	+1.29	96.13	+1.12	multifunctional CCA addition/repair protein
Smlt4009	131.73	141.72	+1.08	162.58	+1.23	SseB family protein
Smlt4010	0.00	0.00	0	0.00	0	hypothetical protein
Smlt4011	0.00	0.00	0	0.00	0	hypothetical protein
Smlt4012	0.00	0.00	0	0.00	0	hemolysin
Smlt4013	0.00	0.00	0	0.00	0	hypothetical protein
Smlt4014	49.17	16.03	-3.07	54.43	+1.11	peptidase
Smlt4016	0.00	0.00	0	0.00	0	hypothetical protein
Smlt4017	0.00	0.00	0	0.00	0	hypothetical protein
Smlt4018	3.44	3.89	+1.13	2.91	-1.18	autotransporter outer membrane beta-barrel domain-containing protein
Smlt4019	18.78	111.08	+5.92	52.03	+2.77	heparan-alpha-glucosaminide N-acetyltransferase domain-containing protein
Smlt4020	24.15	137.50	+5.69	70.20	+2.91	N-acetylglucosamine-6-phosphate deacetylase
Smlt4021	26.67	128.95	+4.84	80.50	+3.02	SIS domain-containing protein
Smlt4022	24.14	70.50	+2.92	87.22	+3.61	LacI family DNA-binding transcriptional regulator
Smlt4023	50.50	127.82	+2.53	72.03	+1.43	sugar MFS transporter
Smlt4025	60.32	45.51	-1.33	47.32	-1.27	glucokinase
Smlt4026	21.96	129.57	+5.9	21.91	-1.	TonB-dependent outer membrane receptor
Smlt4027	27.69	59.73	+2.16	44.52	+1.61	family 20 glycosylhydrolase
Smlt4028	31.08	33.02	+1.06	36.49	+1.17	membrane protein
Smlt4029	44.66	55.67	+1.25	60.29	+1.35	SAM-dependent methyltransferase
Smlt4030	47.30	80.04	+1.69	71.77	+1.52	pteridine reductase
Smlt4031	55.85	20.03	-2.79	62.36	+1.12	2-amino-4-hydroxy-6-hydroxymethyldihydropteridine diphosphokinase
Smlt4032	32.43	66.53	+2.05	31.58	-1.03	hypothetical protein

Smlt4033	0.00	0.00	0	0.00	0	conserved hypothetical protein, pseudogene
Smlt4037	178.97	257.72	+1.44	269.63	+1.51	response regulator
Smlt4038	237.64	345.60	+1.45	374.94	+1.58	response regulator
Smlt4039	138.47	183.36	+1.32	228.12	+1.65	PAS domain-containing protein
Smlt4040	112.90	151.00	+1.34	294.79	+2.61	BON domain-containing protein
Smlt4041	50.05	63.11	+1.26	80.34	+1.61	oxidoreductase
Smlt4042	251.82	195.68	-1.29	913.01	+3.63	decarboxylating 6-phosphogluconate dehydrogenase
Smlt4043	47.40	43.89	-1.08	59.35	+1.25	hypothetical protein
Smlt4044	80.63	75.73	-1.06	58.93	-1.37	DUF2242 domain-containing protein
Smlt4045	291.16	474.98	+1.63	530.67	+1.82	carboxy terminal-processing peptidase
Smlt4046	530.16	503.27	-1.05	509.80	-1.04	lipoyl synthase
Smlt4047	203.49	248.76	+1.22	144.42	-1.41	lipoyl(octanoyl) transferase LipB
Smlt4048	198.62	215.59	+1.09	161.07	-1.23	YbeD family protein
Smlt4049	79.53	95.84	+1.21	370.03	+4.65	lipid A deacylase LpxR family protein
Smlt4050	335.86	427.09	+1.27	468.38	+1.39	D-alanyl-D-alanine carboxypeptidase
Smlt4051	174.74	219.09	+1.25	181.74	+1.04	septal ring lytic transglycosylase RlpA family protein
Smlt4052	368.43	354.91	-1.04	336.93	-1.09	lytic murein transglycosylase B
Smlt4054	7.16	8.21	+1.15	6.32	-1.13	rod shape-determining protein RodA
Smlt4055	30.48	34.53	+1.13	72.26	+2.37	rod shape-determining protein RodA
Smlt4056	30.54	38.87	+1.27	66.03	+2.16	penicillin-binding protein 2
Smlt4057	14.74	17.63	+1.2	25.75	+1.75	rod shape-determining protein MreD
Smlt4058	31.15	31.42	+1.01	54.80	+1.76	rod shape-determining protein MreC
Smlt4059	229.43	207.07	-1.11	356.87	+1.56	rod shape-determining protein
Smlt4060	173.35	118.28	-1.47	236.29	+1.36	carbohydrate kinase family protein
Smlt4061	0.00	0.00	0	0.00	0	transposase, pseudogene
Smlt4062	76.11	97.45	+1.28	116.58	+1.53	HAD family hydrolase
Smlt4063	155.63	183.21	+1.18	254.94	+1.64	hypothetical protein
Smlt4064	383.04	766.12	+2.	503.75	+1.32	M1 family metallopeptidase

Smlt4065	599.01	546.65	-1.1	608.63	+1.02	bifunctional demethylmenaquinone methyltransferase/2-methoxy-6-polyprenyl-1,4-benzoquinol methylase UbiE
Smlt4066	146.13	111.84	-1.31	294.59	+2.02	nucleoside deaminase
Smlt4067	23.40	30.28	+1.29	30.64	+1.31	hypothetical protein
Smlt4068	19.52	14.36	-1.36	37.85	+1.94	hypothetical protein
Smlt4069	52.40	52.32	-1.	55.00	+1.05	low affinity iron permease family protein
Smlt4070	27.18	129.33	+4.76	34.97	+1.29	SmeF
Smlt4071	36.31	226.19	+6.23	41.95	+1.16	SmeE
Smlt4072	36.14	228.24	+6.32	48.73	+1.35	SmeD
Smlt4073	112.07	169.16	+1.51	121.16	+1.08	SmeT
Smlt4074	58.92	104.14	+1.77	66.96	+1.14	Gfo/Idh/MocA family oxidoreductase
Smlt4075	241.66	504.73	+2.09	105.12	-2.3	ATP-dependent protease ATPase subunit HslU
Smlt4076	66.65	176.48	+2.65	33.96	-1.96	ATP-dependent protease subunit HslV
Smlt4077	101.28	120.20	+1.19	119.32	+1.18	tyrosine recombinase XerC
Smlt4078	389.82	553.39	+1.42	426.40	+1.09	DUF484 family protein
Smlt4079	325.57	454.73	+1.4	332.00	+1.02	diaminopimelate epimerase
Smlt4080	210.72	345.88	+1.64	317.22	+1.51	lipoprotein
Smlt4081	53.80	70.69	+1.31	67.13	+1.25	YbaN family protein
Smlt4082	159.04	254.03	+1.6	135.52	-1.17	oligopeptidase B
Smlt4083	46.05	63.23	+1.37	57.90	+1.26	pyridoxal phosphate-dependent aminotransferase
Smlt4084	166.32	249.98	+1.5	374.97	+2.25	prolyl oligopeptidase family serine peptidase
Smlt4085	52.36	88.84	+1.7	34.52	-1.52	hypothetical protein
Smlt4086	112.03	146.93	+1.31	184.22	+1.64	lipocalin family protein
Smlt4087	39.12	49.92	+1.28	23.93	-1.63	ABC transporter permease
Smlt4088	52.36	28.14	-1.86	36.10	-1.45	methionine ABC transporter ATP-binding protein
Smlt4089	99.77	137.45	+1.38	71.93	-1.39	DMT family transporter
Smlt4090	385.57	277.31	-1.39	492.48	+1.28	YajQ family cyclic di-GMP-binding protein
Smlt4091	193.21	104.03	-1.86	219.11	+1.13	DUF1415 domain-containing protein
Smlt4092	62.55	37.87	-1.65	53.48	-1.17	SDR family oxidoreductase

Smlt4093	87.31	222.38	+2.55	74.87	-1.17	M3 family metallopeptidase
Smlt4094	18.99	33.89	+1.78	18.89	-1.01	hypothetical protein
Smlt4095	167.46	114.29	-1.47	134.17	-1.25	PLP-dependent cysteine synthase family protein
Smlt4096	145.13	81.43	-1.78	140.85	-1.03	lactoylglutathione lyase
Smlt4097	93.32	163.87	+1.76	68.82	-1.36	VOC family protein
Smlt4098	542.26	732.56	+1.35	713.73	+1.32	CBS domain-containing protein
Smlt4099	331.84	346.84	+1.05	371.80	+1.12	glutamine--fructose-6-phosphate transaminase (isomerizing)
Smlt4100	1.89	3.45	+1.83	2.43	+1.28	efflux RND transporter periplasmic adaptor subunit
Smlt4101	2.70	2.98	+1.1	2.08	-1.3	ABC transporter ATP-binding protein
Smlt4102	0.00	0.00	0	0.00	0	ABC transporter permease
Smlt4103	0.00	0.00	0	0.00	0	FtsX-like permease family protein
Smlt4104	9.18	16.07	+1.75	9.12	-1.01	PDZ domain-containing protein
Smlt4105	121.01	152.06	+1.26	137.60	+1.14	sigma-54 dependent transcriptional regulator
Smlt4106	81.64	86.88	+1.06	106.68	+1.31	GHKL domain-containing protein
Smlt4107	83.59	79.14	-1.06	104.61	+1.25	GNAT family N-acetyltransferase
Smlt4108	141.81	123.33	-1.15	236.00	+1.66	bifunctional UDP-N-acetylglucosamine diphosphorylase/glucosamine-1-phosphate N-acetyltransferase GlmU
Smlt4109	31.81	22.96	-1.39	40.06	+1.26	GtrA family protein
Smlt4110	547.91	516.47	-1.06	571.38	+1.04	F0F1 ATP synthase subunit epsilon
Smlt4111	1156.45	1138.81	-1.02	1328.89	+1.15	F0F1 ATP synthase subunit beta
Smlt4112	1010.08	1190.89	+1.18	1042.46	+1.03	F0F1 ATP synthase subunit gamma
Smlt4113	1006.59	1208.45	+1.2	926.02	-1.09	F0F1 ATP synthase subunit alpha
Smlt4114	267.88	333.09	+1.24	151.83	-1.76	F0F1 ATP synthase subunit delta
Smlt4115	436.85	414.20	-1.05	281.12	-1.55	F0F1 ATP synthase subunit B
Smlt4116	818.51	673.70	-1.21	468.87	-1.75	ATP synthase c chain
Smlt4117	799.71	583.91	-1.37	386.06	-2.07	F0F1 ATP synthase subunit A
Smlt4118	793.92	716.46	-1.11	376.89	-2.11	hypothetical protein
Smlt4119	125.02	243.89	+1.95	429.57	+3.44	hypothetical protein

Smlt4120	330.05	457.40	+1.39	405.82	+1.23	dihydrolipoyl dehydrogenase
Smlt4121	441.81	651.51	+1.47	545.42	+1.23	dihydrolipoyllysine-residue acetyltransferase
Smlt4122	28.49	21.44	-1.33	19.93	-1.43	DNA-deoxyinosine glycosylase
Smlt4123	1021.99	2924.45	+2.86	359.70	-2.84	outer membrane beta-barrel protein
Smlt4124	28.29	42.09	+1.49	25.72	-1.1	glycerol-3-phosphate dehydrogenase
Smlt4125	21.41	25.51	+1.19	31.00	+1.45	glycerol kinase GlpK
Smlt4126	298.93	469.96	+1.57	385.07	+1.29	MetQ/NlpA family ABC transporter substrate-binding protein
Smlt4127	0.00	0.00	0	0.00	0	DeoR family regulatory protein
Smlt4128	84.42	103.75	+1.23	164.63	+1.95	DUF481 domain-containing protein
Smlt4129	169.14	226.04	+1.34	135.08	-1.25	hydroxymethylbilane synthase
Smlt4130	144.61	145.02	+1.	195.92	+1.35	LytTR family DNA-binding domain-containing protein
Smlt4131	41.81	32.59	-1.28	65.41	+1.56	sensor histidine kinase
Smlt4132	110.35	90.13	-1.22	188.06	+1.7	alpha/beta hydrolase
Smlt4133	119.04	125.26	+1.05	162.05	+1.36	glucans biosynthesis glucosyltransferase MdoH
Smlt4134	0.00	0.00	0	0.00	0	glucosyltransferase, pseudogene
Smlt4135	16.18	58.43	+3.61	1780.96	+110.09	TonB-dependent outer membrane receptor
Smlt4136	0.00	0.00	0	0.00	0	hypothetical protein
Smlt4137	110.27	16.55	-6.66	187.09	+1.7	DUF2628 domain-containing protein
Smlt4138	28.65	12.55	-2.28	33.39	+1.17	methyl-accepting chemotaxis protein
Smlt4139	116.64	104.22	-1.12	198.47	+1.7	NAD(P)-dependent oxidoreductase
Smlt4140	17.74	21.75	+1.23	50.37	+2.84	general stress protein
Smlt4141	3.17	2.49	-1.28	15.87	+5.	MFS transporter
Smlt4142	37.46	130.73	+3.49	54.04	+1.44	TetR/AcrR family transcriptional regulator
Smlt4143	41.03	81.06	+1.98	80.65	+1.97	VOC family protein
Smlt4144	47.64	88.99	+1.87	77.12	+1.62	hypothetical protein
Smlt4145	22.71	6.62	-3.43	155.00	+6.83	autotransporter domain-containing protein
Smlt4146	161.59	124.68	-1.3	88.44	-1.83	hypothetical protein
Smlt4147	231.73	131.89	-1.76	187.76	-1.23	hypothetical protein
Smlt4148	176.88	132.96	-1.33	235.83	+1.33	RHS repeat protein

Smlt4149	101.36	117.72	+1.16	439.01	+4.33	PA2169 family four-helix-bundle protein
Smlt4150	0.00	0.00	0	0.00	0	hypothetical protein
Smlt4151	7.61	8.54	+1.12	7.76	+1.02	TonB-dependent outer membrane receptor
Smlt4152	36.86	43.17	+1.17	25.68	-1.44	hypothetical protein
Smlt4153	8.64	9.27	+1.07	5.97	-1.45	DMT family transporter
Smlt4154	10.50	7.55	-1.39	11.71	+1.12	helix-turn-helix transcriptional regulator
Smlt4155	5.40	6.22	+1.15	4.92	-1.1	glutathione S-transferase family protein
Smlt4156	197.91	133.97	-1.48	103.04	-1.92	hypothetical protein
Smlt4157	334.26	286.62	-1.17	253.52	-1.32	H-NS histone family protein
Smlt4158	17.46	31.12	+1.78	18.14	+1.04	DoxX family protein
Smlt4159	27.25	26.23	-1.04	27.64	+1.01	serine hydrolase
Smlt4160	67.76	64.27	-1.05	45.58	-1.49	hypothetical protein
Smlt4161	0.00	0.00	0	0.00	0	FG-GAP-like repeat-containing protein
Smlt4162	0.00	0.00	0	0.00	0	phage regulatory protein
Smlt4163	0.00	0.00	0	0.00	0	hypothetical protein
Smlt4164	0.00	0.00	0	0.00	0	hypothetical protein
Smlt4165	779.38	953.94	+1.22	621.11	-1.25	RNA polymerase sigma factor RpoD
Smlt4166	19.66	23.20	+1.18	19.51	-1.01	D-tyrosyl-tRNA(Tyr) deacylase
Smlt4167	98.65	106.86	+1.08	137.22	+1.39	lauroyl acyltransferase
Smlt4168	181.16	191.78	+1.06	124.58	-1.45	GTP cyclohydrolase II RibA
Smlt4169	24.23	43.67	+1.8	40.83	+1.69	PH domain-containing protein
Smlt4170	16.90	21.68	+1.28	21.61	+1.28	PH domain-containing protein
Smlt4171	84.08	86.10	+1.02	102.30	+1.22	CDP-glycerol glycerophosphotransferase family protein
Smlt4172	87.18	61.46	-1.42	86.11	-1.01	glycosyltransferase family 2 protein
Smlt4173	86.15	81.34	-1.06	108.95	+1.26	glycosyltransferase family 4 protein
Smlt4174	0.00	0.00	0	0.00	0	O-antigen ligase family protein
Smlt4175	17.78	19.66	+1.11	16.81	-1.06	glycosyltransferase family 39 protein
Smlt4176	0.00	0.00	0	0.00	0	16S rRNA (cytosine(967)-C(5))-methyltransferase RsmB
Smlt4177	103.06	96.52	-1.07	92.50	-1.11	methionyl-tRNA formyltransferase

Smlt4178	491.18	577.49	+1.18	491.75	+1.	peptide deformylase
Smlt4179	332.50	94.56	-3.52	75.27	-4.42	LysM peptidoglycan-binding domain-containing protein
Smlt4180	11.76	16.67	+1.42	4.75	-2.48	DNA-processing protein DprA
Smlt4181	563.40	1147.68	+2.04	1096.82	+1.95	DUF494 family protein
Smlt4182	234.80	347.83	+1.48	321.26	+1.37	GYF domain-containing protein
Smlt4183	185.46	226.66	+1.22	229.30	+1.24	RDD family protein
Smlt4184	137.21	175.22	+1.28	232.89	+1.7	DNA topoisomerase I
Smlt4185	121.45	114.16	-1.06	131.09	+1.08	Sua5/YciO/YrdC/YwlC family protein
Smlt4186	52.67	48.83	-1.08	84.65	+1.61	DUF4124 domain-containing protein
Smlt4187	32.10	47.67	+1.49	47.90	+1.49	hypothetical protein
Smlt4188	75.69	67.67	-1.12	106.72	+1.41	SDR family oxidoreductase
Smlt4189	91.32	219.90	+2.41	89.91	-1.02	hypothetical protein
Smlt4190	155.77	264.89	+1.7	243.45	+1.56	signal peptide peptidase SppA
Smlt4191	56.89	49.12	-1.16	74.33	+1.31	MATE family efflux transporter
Smlt4192	102.33	92.44	-1.11	146.90	+1.44	DUF3106 domain-containing protein
Smlt4193	71.64	82.20	+1.15	104.56	+1.46	membrane protein
Smlt4194	39.36	50.80	+1.29	51.04	+1.3	primosomal protein N'
Smlt4195	212.83	189.70	-1.12	481.02	+2.26	glutathione S-transferase
Smlt4196	131.75	137.38	+1.04	163.74	+1.24	NYN domain-containing protein
Smlt4197	29.72	51.37	+1.73	60.16	+2.02	NAD(P)/FAD-dependent oxidoreductase
Smlt4198	24.64	21.89	-1.13	26.05	+1.06	hypothetical protein
Smlt4199	18.33	16.37	-1.12	27.77	+1.52	nuclear transport factor 2 family protein
Smlt4200	0.00	0.00	0	0.00	0	hypothetical protein
Smlt4201	0.00	0.00	0	0.00	0	GIY-YIG nuclease family protein
Smlt4202	16.10	4.58	-3.52	2.95	-5.45	trimeric intracellular cation channel family protein
Smlt4203	56.37	83.66	+1.48	109.18	+1.94	3-deoxy-7-phosphoheptulonate synthase
Smlt4204	46.46	81.58	+1.76	76.03	+1.64	hypothetical protein
Smlt4205	19.35	9.57	-2.02	16.41	-1.18	hypothetical protein
Smlt4206	36.43	32.56	-1.12	31.94	-1.14	DNA alkylation repair protein

Smlt4207	17.01	9.30	-1.83	14.17	-1.2	MarR family transcriptional regulator
Smlt4208	38.95	48.67	+1.25	53.89	+1.38	HAMP domain-containing histidine kinase
Smlt4209	31.41	38.19	+1.22	29.18	-1.08	response regulator transcription factor
Smlt4210	3.00	3.58	+1.19	1.71	-1.75	aspartyl protease family protein
Smlt4211	19.30	29.80	+1.54	17.50	-1.1	serine hydrolase
Smlt4213	104.84	97.06	-1.08	177.44	+1.69	DUF6445 family protein
Smlt4214	5717.02	3805.00	-1.5	3617.52	-1.58	chaperonin GroEL
Smlt4215	4522.97	2315.83	-1.95	3055.96	-1.48	co-chaperone GroES
Smlt4216	19.07	25.13	+1.32	29.46	+1.54	endonuclease
Smlt4217	31.14	28.47	-1.09	42.56	+1.37	divalent-cation tolerance protein CutA
Smlt4218	99.89	108.96	+1.09	132.31	+1.32	thioredoxin family protein
Smlt4219	50.26	82.31	+1.64	58.05	+1.15	TlpA family protein disulfide reductase
Smlt4220	0.00	0.00	0	0.00	0	PEPSY domain transmembrane protein
Smlt4221	0.00	0.00	0	0.00	0	flavodoxin NAD-binding oxidoreductase
Smlt4222	4.43	4.10	-1.08	3.08	-1.44	methyl-accepting chemotaxis protein
Smlt4223	36.08	31.09	-1.16	42.99	+1.19	VOC family protein
Smlt4224	15.69	12.89	-1.22	23.67	+1.51	response regulator transcription factor
Smlt4225	8.63	6.95	-1.24	13.79	+1.6	two-component sensor histidine kinase
Smlt4226	0.89	0.59	-1.51	0.48	-1.86	DUF2141 domain-containing protein
Smlt4227	1.88	2.18	+1.16	1.90	+1.01	carotenoid oxygenase family protein
Smlt4228	37.54	40.38	+1.08	32.77	-1.15	ABC transporter substrate-binding protein
Smlt4229	18.01	17.38	-1.04	16.93	-1.06	LysR family transcriptional regulator
Smlt4231	11.17	23.62	+2.11	15.13	+1.35	GIY-YIG nuclease family protein
Smlt4232	5.63	6.16	+1.09	4.08	-1.38	alkaline phosphatase
Smlt4233	37.75	29.09	-1.3	23.07	-1.64	D-alanyl-D-alanine carboxypeptidase
Smlt4234	305.77	206.44	-1.48	319.78	+1.05	TetR/AcrR family transcriptional regulator
Smlt4235	15.03	8.22	-1.83	13.69	-1.1	NAD(P)H-dependent oxidoreductase
Smlt4236	0.00	0.00	0	0.00	0	hypothetical protein
Smlt4237	28.68	38.58	+1.35	48.46	+1.69	isocitrate lyase/phosphoenolpyruvate mutase family protein

Smlt4238	1.65	1.71	+1.03	1.87	+1.13	DOPA 4,5-dioxygenase family protein
Smlt4239	73.02	31.85	-2.29	83.59	+1.14	type II 3-dehydroquinate dehydratase
Smlt4240	465.88	294.99	-1.58	586.46	+1.26	acetyl-CoA carboxylase biotin carboxyl carrier protein
Smlt4241	492.99	419.43	-1.18	731.47	+1.48	acetyl-CoA carboxylase biotin carboxylase subunit
Smlt4242	177.13	220.07	+1.24	162.64	-1.09	hypothetical protein
Smlt4243	193.49	235.71	+1.22	203.53	+1.05	hypothetical protein
Smlt4244	50.76	44.44	-1.14	59.33	+1.17	50S ribosomal protein L11 methyltransferase
Smlt4245	60.87	76.59	+1.26	104.24	+1.71	DUF3426 domain-containing protein
Smlt4246	44.25	38.28	-1.16	57.42	+1.3	DNA-binding transcriptional regulator Fis
Smlt4247	15.29	9.02	-1.7	17.40	+1.14	phosphatidate cytidylyltransferase
Smlt4248	26.33	13.73	-1.92	23.04	-1.14	1-acyl-sn-glycerol-3-phosphate acyltransferase
Smlt4249	24.79	12.54	-1.98	38.30	+1.55	hypothetical protein
Smlt4250	13.29	5.35	-2.48	13.99	+1.05	phosphatase PAP2/dual specificity phosphatase family protein
Smlt4251	37.53	16.71	-2.25	41.77	+1.11	bifunctional alpha/beta hydrolase/class I SAM-dependent methyltransferase
Smlt4252	110.17	56.27	-1.96	114.53	+1.04	CDP-alcohol phosphatidyltransferase family protein
Smlt4253	95.86	76.73	-1.25	140.24	+1.46	bifunctional phosphoribosylaminoimidazolecarboxamide formyltransferase/IMP cyclohydrolase
Smlt4254	85.79	87.79	+1.02	146.90	+1.71	phosphoribosylamine--glycine ligase
Smlt4255	160.75	262.82	+1.63	56.73	-2.83	DNA-binding transcriptional regulator
Smlt4256	174.94	394.30	+2.25	120.95	-1.45	type II toxin-antitoxin system RelE/ParE family toxin
Smlt4258	824.28	1420.74	+1.72	595.77	-1.38	RNA polymerase sigma factor RpoH
Smlt4259	269.69	242.47	-1.11	382.86	+1.42	uracil-DNA glycosylase
Smlt4260	159.65	165.70	+1.04	252.20	+1.58	response regulator
Smlt4261	120.81	130.23	+1.08	148.14	+1.23	permease-like cell division protein FtsX
Smlt4262	181.74	330.96	+1.82	208.25	+1.15	cell division ATP-binding protein FtsE
Smlt4263	312.14	333.01	+1.07	256.13	-1.22	ATP-dependent RNA helicase RhlB
Smlt4264	1079.30	1667.64	+1.55	2395.99	+2.22	thioredoxin
Smlt4265	263.41	348.16	+1.32	408.51	+1.55	transcription termination factor Rho

Smlt4266	24.56	18.96	-1.3	38.59	+1.57	sensor domain-containing diguanylate cyclase
Smlt4267	222.88	190.28	-1.17	389.64	+1.75	TonB, energy transducer
Smlt4268	27.61	42.44	+1.54	38.55	+1.4	bifunctional isocitrate dehydrogenase kinase/phosphatase
Smlt4269	48.80	90.13	+1.85	81.69	+1.67	hypothetical protein
Smlt4270	6.51	4.59	-1.42	7.78	+1.2	roadblock/LC7 domain-containing protein
Smlt4271	1.51	2.12	+1.4	3.80	+2.52	ATP/GTP-binding protein
Smlt4272	5.40	4.32	-1.25	6.08	+1.12	hypothetical protein
Smlt4273	508.30	546.39	+1.07	657.88	+1.29	NADP-dependent isocitrate dehydrogenase
Smlt4275	243.53	192.01	-1.27	497.68	+2.04	hypothetical protein
Smlt4276	234.14	226.66	-1.03	701.07	+2.99	LysM peptidoglycan-binding domain-containing protein
Smlt4277	118.94	126.67	+1.06	97.78	-1.22	NADPH-dependent 7-cyano-7-deazaguanine reductase QueF
Smlt4278	109.55	133.11	+1.22	187.05	+1.71	M20 family metallopeptidase
Smlt4279	59.47	71.77	+1.21	71.02	+1.19	efflux RND transporter periplasmic adaptor subunit
Smlt4280	29.33	32.59	+1.11	32.30	+1.1	efflux RND transporter permease subunit
Smlt4281	15.91	20.14	+1.27	22.78	+1.43	efflux RND transporter permease subunit
Smlt4282	42.65	63.06	+1.48	41.98	-1.02	TIGR01666 family membrane protein
Smlt4283	46.19	19.25	-2.4	31.31	-1.48	SDR family oxidoreductase
Smlt4284	94.07	99.61	+1.06	136.59	+1.45	MFS transporter
Smlt4285	484.58	178.81	-2.71	751.49	+1.55	CD225/dispanin family protein
Smlt4286	301.04	110.92	-2.71	217.25	-1.39	CD225/dispanin family protein
Smlt4287	76.81	32.03	-2.4	54.75	-1.4	DUF2752 domain-containing protein
Smlt4289	144.93	195.90	+1.35	139.45	-1.04	regulatory signaling modulator protein AmpE
Smlt4290	315.48	425.61	+1.35	284.45	-1.11	NAD(+) diphosphatase
Smlt4291	323.65	775.83	+2.4	205.10	-1.58	iron-sulfur cluster insertion protein ErpA
Smlt4292	208.03	182.67	-1.14	118.76	-1.75	polymer-forming cytoskeletal protein
Smlt4293	108.84	137.97	+1.27	137.24	+1.26	membrane protein
Smlt4294	54.17	87.64	+1.62	76.75	+1.42	DUF4126 domain-containing protein
Smlt4295	150.63	195.57	+1.3	189.12	+1.26	EAL domain-containing protein
Smlt4296	42.30	63.09	+1.49	66.28	+1.57	response regulator

Smlt4297	1156.71	958.93	-1.21	402.24	-2.88	bacterioferritin
Smlt4298	143.61	225.81	+1.57	1054.62	+7.34	bacterioferritin-associated ferredoxin
Smlt4299	268.92	402.84	+1.5	399.19	+1.48	RNA pyrophosphohydrolase
Smlt4301	719.43	579.39	-1.24	531.40	-1.35	30S ribosomal protein S9
Smlt4302	2243.95	1534.13	-1.46	1747.91	-1.28	50S ribosomal protein L13
Smlt4303	164.82	259.90	+1.58	358.01	+2.17	2-polyprenyl-3-methyl-6-methoxy-1,4-benzoquinone monooxygenase
Smlt4304	41.81	20.73	-2.02	37.55	-1.11	quaternary ammonium compound efflux SMR transporter SugE
Smlt4305	364.84	188.05	-1.94	290.21	-1.26	adenosylmethionine decarboxylase
Smlt4306	58.68	81.63	+1.39	59.21	+1.01	cyclic AMP receptor protein,catabolite gene activator
Smlt4307	32.95	47.06	+1.43	40.23	+1.22	sulfite exporter TauE/SafE family protein
Smlt4308	54.68	71.59	+1.31	92.62	+1.69	haloacid dehalogenase-like hydrolase
Smlt4309	68.76	106.22	+1.54	110.83	+1.61	indole-3-glycerol phosphate synthase TrpC
Smlt4310	41.94	59.09	+1.41	114.29	+2.73	anthranilate phosphoribosyltransferase
Smlt4311	38.34	53.06	+1.38	113.68	+2.96	aminodeoxychorismate/anthranilate synthase component II
Smlt4312	55.48	100.68	+1.81	160.50	+2.89	SIMPL domain-containing protein
Smlt4313	131.59	188.03	+1.43	282.65	+2.15	anthranilate synthase component I
Smlt4314	89.21	102.60	+1.15	150.60	+1.69	lipid kinase YegS
Smlt4315	155.16	87.67	-1.77	167.30	+1.08	GNAT family N-acetyltransferase
Smlt4316	222.73	176.99	-1.26	261.79	+1.18	ribulose-phosphate 3-epimerase
Smlt4317	70.37	96.05	+1.36	161.20	+2.29	DnaJ domain-containing protein
Smlt4318	110.61	91.27	-1.21	127.61	+1.15	phosphoribosylaminoimidazolesuccinocarboxamide synthase
Smlt4319	60.97	109.38	+1.79	58.90	-1.04	DUF962 domain-containing protein
Smlt4320	15.88	38.11	+2.4	14.91	-1.07	monovalent cation/H+ antiporter subunit A
Smlt4321	48.73	122.14	+2.51	64.51	+1.32	Na+/H+ antiporter subunit C
Smlt4322	14.09	38.02	+2.7	17.24	+1.22	monovalent cation/H+ antiporter subunit D
Smlt4323	12.34	20.51	+1.66	15.49	+1.25	Na+/H+ antiporter subunit E
Smlt4324	7.14	19.27	+2.7	11.49	+1.61	K+/H+ antiporter subunit F
Smlt4325	36.15	64.63	+1.79	65.97	+1.82	Na+/H+ antiporter subunit G

Smlt4326	39.27	41.25	+1.05	31.36	-1.25	sodium:calcium antiporter
Smlt4327	75.63	114.22	+1.51	76.86	+1.02	hypothetical protein
Smlt4328	1.83	2.01	+1.1	1.13	-1.61	hypothetical protein
Smlt4329	429.22	335.85	-1.28	358.90	-1.2	homogentisate 1,2-dioxygenase
Smlt4330	877.05	629.97	-1.39	709.44	-1.24	4-hydroxyphenylpyruvate dioxygenase
Smlt4331	80.88	86.55	+1.07	83.88	+1.04	MarR family winged helix-turn-helix transcriptional regulator
Smlt4332	8.64	6.46	-1.34	7.71	-1.12	PDDEXK nuclease domain-containing protein
Smlt4333	29.23	31.67	+1.08	29.91	+1.02	hypothetical protein
Smlt4334	184.88	367.64	+1.99	208.41	+1.13	thioredoxin family protein
Smlt4335	262.13	358.23	+1.37	253.30	-1.03	oligopeptide:H+ symporter
Smlt4336	73.59	44.64	-1.65	73.92	+1.	tryptophan 2,3-dioxygenase
Smlt4338	1686.49	3118.70	+1.85	3393.52	+2.01	pyruvate dehydrogenase (acetyl-transferring) E1 component subunit alpha
Smlt4339	822.30	1498.18	+1.82	1324.79	+1.61	alpha-ketoacid dehydrogenase subunit beta
Smlt4340	272.83	519.84	+1.91	453.83	+1.66	hypothetical protein
Smlt4341	725.52	1204.39	+1.66	1450.11	+2.	2-oxo acid dehydrogenase subunit E2
Smlt4342	4.87	3.87	-1.26	6.06	+1.25	VOC family protein
Smlt4343	12.89	19.98	+1.55	26.68	+2.07	YafY family transcriptional regulator
Smlt4344	48.25	79.40	+1.65	101.09	+2.1	NAD-dependent dehydratase
Smlt4345	26.55	23.30	-1.14	34.41	+1.3	GGDEF domain-containing protein
Smlt4346	70.92	49.45	-1.43	109.17	+1.54	DUF808 domain-containing protein
Smlt4347	52.14	62.14	+1.19	64.87	+1.24	AarF/ABC1/UbiB kinase family protein
Smlt4348	34.23	77.43	+2.26	122.50	+3.58	phosphoglycerol transferase I
Smlt4350	75.99	77.30	+1.02	106.01	+1.4	DUF3772 domain-containing protein
Smlt4352	305.52	224.86	-1.36	368.27	+1.21	hypothetical protein
Smlt4353	5.64	14.79	+2.62	16.51	+2.93	lipocalin family protein
Smlt4354	7.30	14.04	+1.92	18.69	+2.56	FAD-binding oxidoreductase
Smlt4356	99.44	249.41	+2.51	111.57	+1.12	hypothetical protein
Smlt4358	14.91	11.67	-1.28	13.92	-1.07	PepSY domain-containing protein

Smlt4359	3.89	5.15	+1.32	2.79	-1.39	PepSY domain-containing protein
Smlt4360	14.05	20.42	+1.45	24.19	+1.72	response regulator transcription factor
Smlt4361	11.20	15.19	+1.36	11.72	+1.05	HAMP domain-containing histidine kinase
Smlt4362	0.00	0.00	0	0.00	0	hypothetical protein
Smlt4363	50.97	15.29	-3.33	38.45	-1.33	DUF6122 family protein
Smlt4364	154.94	63.83	-2.43	145.28	-1.07	arginine decarboxylase
Smlt4365	225.83	73.82	-3.06	208.39	-1.08	polyamine aminopropyltransferase
Smlt4366	209.63	176.67	-1.19	250.78	+1.2	aspartyl/asparaginyl beta-hydroxylase domain-containing protein
Smlt4368	65.34	78.03	+1.19	34.77	-1.88	kinase
Smlt4369	287.06	237.61	-1.21	357.61	+1.25	IS481-like element ISStma12 family transposase
Smlt4370	521.32	463.08	-1.13	503.65	-1.04	P-II family nitrogen regulator
Smlt4371	119.08	156.15	+1.31	177.75	+1.49	accessory factor UbiK family protein
Smlt4372	54.94	55.65	+1.01	63.94	+1.16	NAD(+)--rifampin ADP-ribosyltransferase
Smlt4373	0.00	0.00	0	0.00	0	DUF4279 domain-containing protein
Smlt4374	0.00	0.00	0	0.00	0	hypothetical protein
Smlt4375	0.00	0.00	0	0.00	0	hypothetical protein
Smlt4376	0.00	0.00	0	0.00	0	hypothetical protein
Smlt4378	0.00	0.00	0	0.00	0	DUF998 domain-containing protein
Smlt4380	0.00	0.00	0	0.00	0	conserved hypothetical exported protein
Smlt4381	0.00	0.00	0	0.00	0	hypothetical protein
Smlt4382	58.11	81.31	+1.4	133.55	+2.3	DUF1629 domain-containing protein
Smlt4383	20.38	22.55	+1.11	42.59	+2.09	AHH domain-containing protein
Smlt4384	0.00	0.00	0	0.00	0	carbon-nitrogen hydrolase family protein
Smlt4385	4.32	5.82	+1.35	3.94	-1.1	YifB family Mg chelatase-like AAA ATPase
Smlt4386	0.00	0.00	0	0.00	0	hypothetical protein
Smlt4387	2.83	1.90	-1.49	2.80	-1.01	TonB-dependent outer membrane receptor
Smlt4389	0.00	0.00	0	0.00	0	hypothetical protein
Smlt4390	29.29	16.36	-1.79	34.15	+1.17	sugar-phosphatase
Smlt4391	31.39	95.97	+3.06	18.20	-1.72	exopolysaccharide biosynthesis protein

Smlt4392	49.46	83.64	+1.69	78.85	+1.59	sorbosone dehydrogenase family protein
Smlt4392A	4.96	12.07	+2.44	5.78	+1.17	hypothetical protein
Smlt4394	0.00	0.00	0	0.00	0	hypothetical protein
Smlt4395	20.18	21.47	+1.06	35.25	+1.75	S8 family serine peptidase
Smlt4396	18.72	22.68	+1.21	30.68	+1.64	hypothetical protein
Smlt4397	6.09	6.84	+1.12	6.38	+1.05	SCO family protein
Smlt4398	0.91	1.50	+1.66	0.87	-1.04	cytochrome o ubiquinol oxidase subunit IV
Smlt4399	2.13	0.92	-2.33	1.18	-1.8	cytochrome o ubiquinol oxidase subunit III
Smlt4400	4.08	6.48	+1.59	3.96	-1.03	cytochrome o ubiquinol oxidase subunit I
Smlt4401	9.49	4.77	-1.99	5.76	-1.65	ubiquinol oxidase subunit II
Smlt4402	72.15	132.96	+1.84	101.02	+1.4	PLP-dependent aminotransferase family protein
Smlt4403	14.32	22.66	+1.58	16.82	+1.17	hypothetical protein
Smlt4404	4.78	2.77	-1.72	4.83	+1.01	DUF6436 domain-containing protein
Smlt4405	3.73	6.81	+1.82	7.06	+1.89	PLP-dependent aminotransferase family protein
Smlt4409	45.92	35.65	-1.29	37.61	-1.22	aminotransferase class V-fold PLP-dependent enzyme
Smlt4410	4.24	1.94	-2.18	6.70	+1.58	TonB-dependent outer membrane receptor
Smlt4411	0.00	0.00	0	0.00	0	hypothetical protein
Smlt4412	11.02	9.55	-1.15	24.88	+2.26	AraC family transcriptional regulator
Smlt4413	4.61	6.01	+1.3	7.81	+1.69	alpha/beta hydrolase
Smlt4414	24.12	29.84	+1.24	47.00	+1.95	VOC family protein
Smlt4415	21.31	23.07	+1.08	28.62	+1.34	TPM domain-containing protein
Smlt4416	31.12	29.68	-1.05	55.72	+1.79	M3 family metallopeptidase
Smlt4417	55.55	73.95	+1.33	121.26	+2.18	hypothetical protein
Smlt4418	33.67	24.55	-1.37	39.14	+1.16	hypothetical protein
Smlt4420	38.84	27.56	-1.41	139.25	+3.59	TIGR00366 family protein
Smlt4422	59.79	42.81	-1.4	58.34	-1.03	4-hydroxybenzoate octaprenyltransferase
Smlt4423	38.70	25.51	-1.52	21.57	-1.79	hypothetical protein
Smlt4424	0.00	0.00	0	0.00	0	hypothetical protein
Smlt4426	0.00	0.00	0	0.00	0	hypothetical protein

Smlt4428	0.00	0.00	0	0.00	0	conserved hypothetical protein
Smlt4429	6.10	10.77	+1.77	8.25	+1.35	hypothetical protein
Smlt4430	0.00	0.00	0	0.00	0	Rhs-family exported protein
Smlt4431	42.98	60.25	+1.4	47.93	+1.12	Gfo/Idh/MocA family oxidoreductase
Smlt4432	4.33	5.14	+1.19	4.62	+1.07	TonB-dependent outer membrane receptor
Smlt4433	4.78	4.93	+1.03	3.40	-1.41	NPCBM/NEW2 domain-containing protein
Smlt4434	8.62	10.01	+1.16	8.68	+1.01	SIS domain-containing protein
Smlt4435	4.58	5.94	+1.3	4.26	-1.07	ROK family protein
Smlt4436	7.40	6.92	-1.07	7.21	-1.03	D-tagatose-bisphosphate aldolase, class II, non-catalytic subunit
Smlt4437	28.63	25.91	-1.1	45.72	+1.6	DeoR family transcriptional regulator
Smlt4438	36.43	32.23	-1.13	50.55	+1.39	N-acetylglucosamine-6-phosphate deacetylase
Smlt4439	0.00	0.00	0	0.00	0	MFS transporter
Smlt4440	0.00	0.00	0	0.00	0	hypothetical protein
Smlt4441	84.82	93.74	+1.11	67.03	-1.27	HdeD family acid-resistance protein
Smlt4443	83.77	55.46	-1.51	17.83	-4.7	hemagglutinin repeat-containing protein
Smlt4444A	167.38	93.31	-1.79	56.21	-2.98	hypothetical protein
Smlt4447A	148.51	106.13	-1.4	105.27	-1.41	hypothetical protein
Smlt4447B	0.00	0.00	0	0.00	0	conserved hypothetical protein
Smlt4447C	74.73	40.43	-1.85	41.38	-1.81	hypothetical protein
Smlt4450	0.00	0.00	0	0.00	0	haemagglutinin, pseudogene
Smlt4451	0.00	0.00	0	0.00	0	cell surface haemagluttinin protein, pseudogene
Smlt4452	21.15	6.70	-3.16	33.99	+1.61	hemagglutinin repeat-containing protein
Smlt4453	145.51	14.14	-10.29	86.64	-1.68	ShlB/FhaC/HecB family hemolysin secretion/activation protein
Smlt4454	4.37	3.50	-1.25	2.77	-1.58	ComF family protein
Smlt4455	10.05	10.38	+1.03	8.99	-1.12	SMP-30/gluconolactonase/LRE family protein
Smlt4458	4.34	4.37	+1.01	13.95	+3.21	chloride channel protein
Smlt4459	11.70	17.69	+1.51	11.77	+1.01	biotin synthase BioB
Smlt4460	49.40	39.49	-1.25	62.47	+1.26	8-amino-7-oxononanoate synthase
Smlt4461	30.84	23.46	-1.31	58.69	+1.9	pimeloyl-ACP methyl ester esterase BioH

Smlt4462	27.07	23.04	-1.17	48.47	+1.79	SDR family oxidoreductase
Smlt4463	44.47	29.80	-1.49	67.08	+1.51	malonyl-ACP O-methyltransferase BioC
Smlt4469	49.45	79.97	+1.62	57.23	+1.16	YdcF family protein
Smlt4470	67.64	113.95	+1.68	84.99	+1.26	pyridoxal-phosphate dependent enzyme
Smlt4471	0.00	0.00	0	0.00	0	hypothetical protein
Smlt4472	0.00	0.00	0	0.00	0	conserved hypothetical protein
Smlt4473	37.50	25.79	-1.45	50.66	+1.35	tRNA uridine-5-carboxymethylaminomethyl(34) synthesis enzyme MnmG
Smlt4474	4.58	4.52	-1.01	4.28	-1.07	multidrug efflux transporter outer membrane subunit SmeC
Smlt4475	2.87	2.03	-1.41	2.18	-1.32	multidrug efflux RND transporter permease subunit SmeB
Smlt4476	1.26	0.70	-1.81	1.19	-1.06	multidrug efflux RND transporter periplasmic adaptor subunit SmeA
Smlt4477	21.70	18.67	-1.16	37.64	+1.73	sensor histidine kinase efflux regulator BaeS
Smlt4478	8.52	8.20	-1.04	19.65	+2.31	response regulator
Smlt4479	61.91	46.25	-1.34	82.30	+1.33	nucleotidyltransferase domain-containing protein
Smlt4480	139.29	120.32	-1.16	243.63	+1.75	gamma carbonic anhydrase family protein
Smlt4481	73.32	45.94	-1.6	86.12	+1.17	RDD family protein
Smlt4482	26.73	14.41	-1.85	26.76	+1.	stage II sporulation protein M
Smlt4483	25.13	21.07	-1.19	35.42	+1.41	DUF4129 domain-containing protein
Smlt4484	19.60	17.20	-1.14	25.35	+1.29	DUF4350 domain-containing protein
Smlt4485	19.86	20.31	+1.02	37.08	+1.87	MoxR family ATPase
Smlt4486	14.80	11.98	-1.24	24.51	+1.66	DUF58 domain-containing protein
Smlt4487	0.00	0.00	0	0.00	0	conserved hypothetical protein
Smlt4489	59.11	84.48	+1.43	56.56	-1.05	hypothetical protein
Smlt4490	316.36	555.54	+1.76	369.99	+1.17	glutathionylspermidine synthase family protein
Smlt4491	382.51	998.75	+2.61	362.19	-1.06	DUF1190 domain-containing protein
Smlt4492	26.73	109.39	+4.09	46.49	+1.74	dihydroxy-acid dehydratase
Smlt4493	105.33	108.27	+1.03	139.41	+1.32	ATP-dependent DNA helicase DinG
Smlt4494	58.14	63.69	+1.1	62.20	+1.07	hypothetical protein

Smlt4495	255.06	262.46	+1.03	399.63	+1.57	shikimate dehydrogenase
Smlt4495A	31.41	25.81	-1.22	50.80	+1.62	PD40 domain-containing protein
Smlt4496	247.13	316.44	+1.28	367.64	+1.49	hypothetical protein
Smlt4497	168.61	301.01	+1.79	341.39	+2.02	porphobilinogen synthase
Smlt4498	53.96	73.77	+1.37	72.31	+1.34	bestrophin family protein
Smlt4499	435.12	897.08	+2.06	493.94	+1.14	hypothetical protein
Smlt4500	154.03	290.55	+1.89	110.66	-1.39	hypothetical protein
Smlt4501	56.71	79.68	+1.4	117.12	+2.07	DUF885 domain-containing protein
Smlt4502	153.86	209.60	+1.36	197.31	+1.28	membrane protein
Smlt4503	211.01	244.72	+1.16	295.60	+1.4	S9 family peptidase
Smlt4504	24.15	18.93	-1.28	21.12	-1.14	glutathione S-transferase N-terminal domain-containing protein
Smlt4505	23.55	14.60	-1.61	32.52	+1.38	TIGR00645 family protein
Smlt4506	74.57	54.55	-1.37	50.45	-1.48	TonB, energy transducer
Smlt4507	0.00	0.00	0	0.00	0	hypothetical protein
Smlt4508	4.44	4.07	-1.09	8.93	+2.01	helix-turn-helix transcriptional regulator
Smlt4509	2.91	3.33	+1.14	6.55	+2.25	transposase
Smlt4510	40.08	44.79	+1.12	51.66	+1.29	ABC transporter permease
Smlt4511	34.82	41.87	+1.2	46.89	+1.35	ATP-binding cassette domain-containing protein
Smlt4512	40.39	46.09	+1.14	71.66	+1.77	alpha/beta hydrolase
Smlt4513	0.00	0.00	0	0.00	0	GGDEF domain signalling protein
Smlt4514	2.57	2.21	-1.17	1.59	-1.62	DUF1203 domain-containing protein
Smlt4515	8.68	9.08	+1.05	7.31	-1.19	lytic polysaccharide monooxygenase
Smlt4516	4.45	4.62	+1.04	4.16	-1.07	lytic polysaccharide monooxygenase
Smlt4517	149.08	181.03	+1.21	108.24	-1.38	ferredoxin--NADP reductase
Smlt4518	131.73	120.71	-1.09	147.32	+1.12	ABC transporter ATP-binding protein
Smlt4519	137.46	147.27	+1.07	143.80	+1.05	ABC transporter permease
Smlt4520	0.00	0.00	0	0.00	0	hypothetical protein
Smlt4521	45.69	35.44	-1.29	80.41	+1.76	2-dehydropantoate 2-reductase
Smlt4522	0.00	0.00	0	0.00	0	epimerase

Smlt4523	46.11	31.07	-1.48	47.31	+1.03	TatD family hydrolase
Smlt4524	51.59	46.86	-1.1	74.50	+1.44	ATP-dependent helicase HrpB
Smlt4525	0.00	0.00	0	0.00	0	hypothetical protein
Smlt4526	85.87	66.55	-1.29	81.39	-1.05	pseudouridine synthase
Smlt4527	0.00	0.00	0	0.00	0	hypothetical protein
Smlt4528	78.82	107.63	+1.37	179.95	+2.28	class I SAM-dependent methyltransferase
Smlt4529	184.98	87.37	-2.12	240.56	+1.3	aldo/keto reductase
Smlt4532	57.90	21.22	-2.73	27.40	-2.11	slipin family protein
Smlt4533	36.56	23.30	-1.57	18.48	-1.98	RtcB family protein
Smlt4534	29.71	87.60	+2.95	25.42	-1.17	NAD(P)/FAD-dependent oxidoreductase
Smlt4535	22.32	55.28	+2.48	17.35	-1.29	Rrf2 family transcriptional regulator
Smlt4536	143.54	64.68	-2.22	148.88	+1.04	methyl-accepting chemotaxis protein
Smlt4537	15.11	35.38	+2.34	11.74	-1.29	LysR family transcriptional regulator
Smlt4538	1.24	0.96	-1.29	0.45	-2.74	MFS transporter
Smlt4539	92.52	57.31	-1.61	77.08	-1.2	2,5-didehydrogluconate reductase DkgB
Smlt4540	0.00	0.00	0	0.00	0	response regulator
Smlt4541	0.00	0.00	0	0.00	0	hypothetical protein
Smlt4543	0.00	0.00	0	0.00	0	YdcF family protein
Smlt4544	103.43	73.95	-1.4	126.29	+1.22	alpha/beta hydrolase
Smlt4545	0.00	0.00	0	0.00	0	colicin secretion/processing ATP-binding protein, pseudogene
Smlt4546	0.00	0.00	0	0.00	0	hypothetical protein
Smlt4547	0.00	0.00	0	0.00	0	transposase, pseudogene
Smlt4548	0.00	0.00	0	0.00	0	phage-related protein
Smlt4549	0.00	0.00	0	0.00	0	conserved hypothetical protein, pseudogene
Smlt4550	63.37	83.99	+1.33	110.31	+1.74	bifunctional acetate--CoA ligase family protein/GNAT family N-acetyltransferase
Smlt4551	31.07	15.41	-2.02	20.46	-1.52	magnesium transporter
Smlt4552	41.53	24.69	-1.68	53.89	+1.3	monovalent cation:proton antiporter-2 (CPA2) family protein
Smlt4553	122.48	23.64	-5.18	185.36	+1.51	ankyrin repeat domain-containing protein

Smlt4554	25.71	9.88	-2.6	27.20	+1.06	YcgL domain-containing protein
Smlt4555	92.66	168.46	+1.82	104.59	+1.13	hypothetical protein
Smlt4556	59.33	113.81	+1.92	79.09	+1.33	beta-ketoacyl-ACP synthase
Smlt4557	89.71	150.82	+1.68	92.90	+1.04	3-ketoacyl-ACP reductase FabG2
Smlt4558	50.59	64.58	+1.28	81.20	+1.6	hotdog family protein
Smlt4559	37.35	56.02	+1.5	60.59	+1.62	beta-ketoacyl-[acyl-carrier-protein] synthase family protein
Smlt4560	16.21	18.19	+1.12	20.93	+1.29	DUF3261 domain-containing protein
Smlt4561	35.20	55.53	+1.58	45.53	+1.29	tryptophan 7-halogenase
Smlt4562	36.84	47.51	+1.29	42.29	+1.15	hypothetical protein
Smlt4563	80.11	104.79	+1.31	128.06	+1.6	outer membrane lipoprotein carrier protein LolA
Smlt4564	41.89	63.17	+1.51	57.68	+1.38	acyl-CoA thioesterase
Smlt4565	46.67	70.37	+1.51	62.28	+1.33	aromatic amino acid ammonia-lyase
Smlt4566	28.25	26.94	-1.05	22.48	-1.26	acyltransferase
Smlt4567	37.94	41.00	+1.08	34.65	-1.1	glycosyltransferase family 2 protein
Smlt4568	70.57	79.79	+1.13	71.93	+1.02	AMP-binding protein
Smlt4569	105.99	108.06	+1.02	56.79	-1.87	hypothetical protein
Smlt4570	141.73	171.55	+1.21	76.92	-1.84	acyl carrier protein
Smlt4571	189.07	212.17	+1.12	134.09	-1.41	phosphopantetheine-binding protein
Smlt4572	68.50	69.54	+1.02	51.59	-1.33	1-acyl-sn-glycerol-3-phosphate acyltransferase
Smlt4573	71.14	62.17	-1.14	53.70	-1.32	beta-ketoacyl synthase chain length factor
Smlt4574	18.81	36.59	+1.95	45.66	+2.43	cytochrome d ubiquinol oxidase subunit II
Smlt4575	35.65	46.77	+1.31	69.41	+1.95	cytochrome ubiquinol oxidase subunit I
Smlt4576	0.00	0.00	0	0.00	0	ABC transporter permease
Smlt4577	0.00	0.00	0	0.00	0	ABC transporter permease
Smlt4578	0.00	0.00	0	0.00	0	hypothetical protein
Smlt4579	0.00	0.00	0	0.00	0	sigma-70 family RNA polymerase sigma factor
Smlt4580	15.31	13.36	-1.15	19.84	+1.3	hypothetical protein
Smlt4581	58.52	74.11	+1.27	165.06	+2.82	S9 family peptidase
Smlt4582	25.67	23.18	-1.11	55.95	+2.18	NUDIX domain-containing protein

Smlt4583	10.35	5.49	-1.89	13.53	+1.31	hypothetical protein
Smlt4584	10.82	4.67	-2.32	12.68	+1.17	hypothetical protein
Smlt4585	29.43	15.36	-1.92	30.01	+1.02	hypothetical protein
Smlt4586	38.75	33.48	-1.16	56.40	+1.46	oxygen-dependent coproporphyrinogen oxidase
Smlt4587	26.50	15.23	-1.74	31.00	+1.17	hypothetical protein
Smlt4588	120.48	266.74	+2.21	189.59	+1.57	DUF421 domain-containing protein
Smlt4589	135.31	170.61	+1.26	192.81	+1.42	DNA polymerase I
Smlt4590	406.87	506.16	+1.24	477.29	+1.17	DUF2782 domain-containing protein
Smlt4591	2158.15	4047.61	+1.88	797.83	-2.71	universal stress protein
Smlt4592	175.79	236.99	+1.35	237.89	+1.35	hypothetical protein
Smlt4593	0.00	0.00	0	0.00	0	hypothetical protein
Smlt4594	22.37	29.26	+1.31	31.19	+1.39	low molecular weight protein tyrosine phosphatase family protein
Smlt4595	262.49	386.49	+1.47	220.25	-1.19	DNA helicase II
Smlt4596	126.46	158.31	+1.25	149.12	+1.18	FAD/NAD(P)-binding protein
Smlt4597	23.06	26.97	+1.17	37.30	+1.62	cardiolipin synthase
Smlt4598	897.57	668.52	-1.34	638.79	-1.41	50S ribosomal protein L33
Smlt4599	5851.93	4567.20	-1.28	3868.83	-1.51	50S ribosomal protein L28
Smlt4601	31.09	35.67	+1.15	41.52	+1.34	glycosyltransferase
Smlt4602	97.57	69.98	-1.39	74.25	-1.31	DNA polymerase III subunit epsilon
Smlt4603	0.00	0.00	0	0.00	0	LysR family transcriptional regulator
Smlt4604	2.32	3.84	+1.66	3.42	+1.47	RidA family protein
Smlt4605	144.00	1106.74	+7.69	163.83	+1.14	hypothetical protein
Smlt4606	11.72	101.50	+8.66	5.01	-2.34	TolC family protein
Smlt4607	12.63	130.79	+10.36	5.21	-2.42	efflux RND transporter periplasmic adaptor subunit
Smlt4608	22.41	160.22	+7.15	6.43	-3.49	CusA/CzcA family heavy metal efflux RND transporter
Smlt4609	31.85	34.86	+1.09	75.09	+2.36	DUF885 family protein
Smlt4610	103.99	82.71	-1.26	98.58	-1.05	lipid-A-disaccharide synthase N-terminal domain-containing protein
Smlt4611	152.25	103.91	-1.47	164.06	+1.08	glycosyltransferase family 2 protein

Smlt4612	97.25	63.39	-1.53	107.70	+1.11	NAD-dependent epimerase/dehydratase family protein
Smlt4613	148.43	136.96	-1.08	158.74	+1.07	suppressor of fused domain protein
Smlt4614	211.83	238.59	+1.13	301.30	+1.42	ParB/RepB/Spo0J family partition protein
Smlt4615	400.79	331.63	-1.21	430.10	+1.07	ParA family protein
Smlt4616	109.97	137.48	+1.25	65.88	-1.67	16S rRNA (guanine(527)-N(7))-methyltransferase RsmG
Smlt4617	54.66	51.58	-1.06	79.97	+1.46	4'-phosphopantetheinyl transferase superfamily protein
Smlt4618	0.00	0.00	0	0.00	0	hypothetical protein
Smlt4619	201.84	174.08	-1.16	242.39	+1.2	exodeoxyribonuclease III
Smlt4620	13.83	22.94	+1.66	27.45	+1.99	coniferyl aldehyde dehydrogenase
Smlt4621	15.89	52.41	+3.3	47.54	+2.99	MHS family MFS transporter
Smlt4622	67.53	119.49	+1.77	307.34	+4.55	hypothetical protein
Smlt4623	348.86	503.78	+1.44	1088.43	+3.12	acetate--CoA ligase
Smlt4624	127.13	170.51	+1.34	355.81	+2.8	response regulator transcription factor
Smlt4626	35.47	26.86	-1.32	38.98	+1.1	manganese efflux pump MntP family protein
Smlt4627	24.06	31.98	+1.33	50.46	+2.1	hybrid sensor histidine kinase/response regulator
Smlt4628	84.07	89.58	+1.07	166.03	+1.97	S46 family peptidase
Smlt4629	0.00	0.00	0	0.00	0	molecular chaperone HscC
Smlt4630	0.00	0.00	0	0.00	0	hypothetical protein
Smlt4631	0.00	0.00	0	0.00	0	heat-shock protein
Smlt4632	132.81	162.81	+1.23	206.53	+1.56	translocation/assembly module TamB
Smlt4633	202.52	202.50	-1.	328.06	+1.62	autotransporter assembly complex protein TamA
Smlt4634	166.05	178.21	+1.07	242.29	+1.46	glycine--tRNA ligase subunit beta
Smlt4635	206.51	131.18	-1.57	220.89	+1.07	glycine--tRNA ligase subunit alpha
Smlt4636	48.17	47.12	-1.02	72.50	+1.51	GspE/PulE family protein
Smlt4637	64.19	92.33	+1.44	112.40	+1.75	glutamine amidotransferase
Smlt4638	78.12	77.45	-1.01	101.15	+1.29	hypothetical protein
Smlt4639	154.01	169.60	+1.1	229.70	+1.49	twin-arginine translocase subunit TatC
Smlt4640	264.05	289.74	+1.1	534.12	+2.02	Sec-independent protein translocase protein TatB
Smlt4641	371.36	637.08	+1.72	701.84	+1.89	Sec-independent protein translocase subunit TatA

Smlt4642	195.74	385.81	+1.97	201.00	+1.03	hypothetical protein
Smlt4643	117.05	108.94	-1.07	112.58	-1.04	ferrochelatase
Smlt4644	68.69	65.97	-1.04	65.37	-1.05	alpha/beta hydrolase
Smlt4645	58.03	9.60	-6.05	33.60	-1.73	methyl-accepting chemotaxis protein
Smlt4647	31.86	35.08	+1.1	51.68	+1.62	benzoate/H(+) symporter BenE family transporter
Smlt4648	0.00	0.00	0	0.00	0	transmembrane protein
Smlt4649	108.27	95.19	-1.14	134.67	+1.24	D-hexose-6-phosphate mutarotase
Smlt4650	41.47	58.48	+1.41	50.55	+1.22	lytic murein transglycosylase
Smlt4651	9.82	21.67	+2.21	30.70	+3.13	L-histidine N(alpha)-methyltransferase
Smlt4652	15.94	25.38	+1.59	27.52	+1.73	ergothioneine biosynthesis protein EgtB
Smlt4653	28.90	6.75	-4.28	25.15	-1.15	GGDEF domain-containing protein
Smlt4654	18.42	36.83	+2.	19.39	+1.05	YchJ family protein
Smlt4655	11.21	4.91	-2.29	8.25	-1.36	TSUP family transporter
Smlt4656	109.83	99.80	-1.1	204.12	+1.86	ATP-binding protein
Smlt4657	27.39	27.49	+1.	50.93	+1.86	DUF4194 domain-containing protein
Smlt4658	76.47	46.80	-1.63	88.85	+1.16	DUF3375 domain-containing protein
Smlt4659	223.48	136.07	-1.64	302.51	+1.35	GTP cyclohydrolase I FolE
Smlt4660	174.77	397.36	+2.27	72.24	-2.42	MarR family transcriptional regulator
Smlt4661	6.46	8.72	+1.35	2.21	-2.93	DUF1656 domain-containing protein
Smlt4662	30.55	55.82	+1.83	26.13	-1.17	HlyD family secretion protein
Smlt4663	12.63	25.16	+1.99	13.47	+1.07	efflux transporter outer membrane subunit
Smlt4664	9.17	28.75	+3.14	18.80	+2.05	FUSC family protein
Smlt4665	97.32	126.56	+1.3	124.82	+1.28	hypothetical protein
Smlt4666	0.00	0.00	0	0.00	0	conserved hypothetical protein
Smlt4667	32.15	47.72	+1.48	52.17	+1.62	exodeoxyribonuclease V subunit alpha
Smlt4668	52.91	73.00	+1.38	75.44	+1.43	exodeoxyribonuclease V subunit beta
Smlt4669	53.93	73.55	+1.36	82.32	+1.53	exodeoxyribonuclease V subunit gamma
Smlt4670	278.94	502.64	+1.8	245.37	-1.14	ATP-binding cassette domain-containing protein
Smlt4671	123.55	221.40	+1.79	116.39	-1.06	MlaE family lipid ABC transporter permease subunit

Smlt4672	106.42	191.90	+1.8	99.72	-1.07	outer membrane lipid asymmetry maintenance protein MlaD
Smlt4673	374.48	569.05	+1.52	366.86	-1.02	ABC transporter substrate-binding protein
Smlt4674	19.09	45.06	+2.36	28.42	+1.49	STAS domain-containing protein
Smlt4675	186.01	300.79	+1.62	237.37	+1.28	VacJ family lipoprotein
Smlt4676	69.18	70.70	+1.02	111.17	+1.61	glutathione peroxidase
Smlt4677	38.17	43.58	+1.14	47.49	+1.24	hypothetical protein
Smlt4678	159.93	246.15	+1.54	122.36	-1.31	DNA recombination protein RmuC
Smlt4679	26.00	26.63	+1.02	76.45	+2.94	glutathione S-transferase N-terminal domain-containing protein
Smlt4680	6.73	4.62	-1.46	6.78	+1.01	zinc-binding alcohol dehydrogenase family protein
Smlt4681	5.27	6.50	+1.23	7.33	+1.39	LysR family transcriptional regulator
Smlt4682	13.04	14.79	+1.13	13.89	+1.06	leucine efflux protein LeuE
Smlt4683	0.00	0.00	0	0.00	0	hypothetical protein
Smlt4684	0.00	0.00	0	0.00	0	suppressor of fused domain protein
Smlt4685	128.93	156.60	+1.21	161.87	+1.26	amino acid permease
Smlt4686	112.00	133.56	+1.19	200.97	+1.79	dipeptidase
Smlt4687	609.61	649.55	+1.07	501.09	-1.22	class I SAM-dependent rRNA methyltransferase
Smlt4688	45.33	114.81	+2.53	24.01	-1.89	TerC family protein
Smlt4689	29.14	111.03	+3.81	25.97	-1.12	rhomboid family intramembrane serine protease
Smlt4690	48.36	41.11	-1.18	72.58	+1.5	glycerophosphodiester phosphodiesterase
Smlt4691	47.64	31.18	-1.53	46.10	-1.03	tRNA uridine-5-carboxymethylaminomethyl(34) synthesis GTPase MnmE
Smlt4692	36.44	24.87	-1.47	44.57	+1.22	polysaccharide deacetylase family protein
Smlt4693	243.69	292.07	+1.2	344.51	+1.41	membrane protein insertase YidC
Smlt4694	186.59	152.27	-1.23	145.80	-1.28	ribonuclease P protein component
Smlt4695	418.69	231.29	-1.81	187.81	-2.23	50S ribosomal protein L34

[a]TPM, Transcripts Per Kilobase Million

[b]Negative fold changes represent genes that were significantly downregulated in response to *smeYZ* inactivation or DIP treatment, whereas positive fold changes represent upregulation in response to *smeYZ* deletion or DIP treatment.

Table S2 Bacterial strains and plasmids used in this study

Primer	Genotype or properties	Reference
S. maltophilia		
KJ	A clinical *S. maltophilia* isolate	1
KJΔYZ	*S. maltophilia* KJ mutant of *smeYZ*, *ΔsmeYZ*	2
KJΔEnt	*S. maltophilia* KJ mutant of *entF* and *entA* genes; *ΔentF, ΔentA*	3
KJΔYZΔEnt	*S. maltophilia* KJ mutant of *entF, entA,* and *smeYZ*; *ΔentF, ΔentA, ΔsmeYZ*	This study
KJΔSbiAB	*S. maltophilia* KJ mutant of *sbiAB* operon; *ΔsbiAB*	This study
KJΔDEF	*S. maltophilia* KJ mutant of *smeDEF* operon; *ΔsmeDEF*	4
KJΔYZΔSbiAB	*S. maltophilia* KJ mutant of *smeYZ* and *sbiAB* operons; *ΔsmeYZ, ΔsbiAB*	This study
KJΔYZΔDEF	*S. maltophilia* KJ mutant of *smeYZ* and *smeDEF* operons; *ΔsmeYZ, ΔsmeDEF*	This study
KJΔYZΔSbiABΔDEF	*S. maltophilia* KJ mutant of *smeYZ, sbiAB* and *smeDEF* operon;, *ΔsmeYZ, ΔsbiAB, ΔsmeDEF*	This study
KJΔFur	*S. maltophilia* KJ mutant of *fur, Δfur*	3
KJΔFurΔEnt	*S. maltophilia* KJ mutant of *fur, entF,* and *entA; Δfur, ΔentF, ΔentA*	This study
KJΔFurΔYZ	*S. maltophilia* KJ mutant of *fur* and *smeYZ; Δfur, ΔsmeYZ*	This study
KJΔFurΔSbiAB	*S. maltophilia* KJ mutant of *fur* and *sbiAB; Δfur, ΔsbiAB*	This study
KJΔFurΔDEF	*S. maltophilia* KJ mutant of *fur* and *smeDEF; Δfur, ΔsmeDEF*	This study
KJΔFurΔYZΔSbiAB	*S. maltophilia* KJ mutant of *fur, smeYZ,* and *sbiAB; Δfur, ΔsbiAB*	This study
KJΔFurΔYZΔDEF	*S. maltophilia* KJ mutant of *fur, smeYZ,* and *smeDEF; Δfur, ΔsmeYZ, ΔsmeDEF*	This study
KJΔFurΔYZΔSbiABΔDEF	*S. maltophilia* KJ mutant of *fur, smeYZ, sbiAB,* and *smeDEF; Δfur, ΔsmeYZ, ΔsbiAB, ΔsmeDEF*	This study
E. coli		
DH5a	F- φ80d/*acZΔM15* Δ(*lacZYA-argF*)*U169 deoR recA1 endA1 hsdR17* (r_k^- m_k^+) *phoA supE44λ thi-1 gyrA96 relA1*	Invitrogen
S17-1	λ*pir*$^+$ mating strain	5
**Plasmids**		

pEX18Tc	*sacB oriT*, Tc^r	6
pRK415	Mobilizable broad-host-range plasmid cloning vector, RK2 origin; Tc	7
pRKXylE	A pRK415-derived vector for the construction of promoter-*xylE* transcription fusion, the orientation of *xylE* gene in this plasmid is opposite to that of P_{lacZ} of pRK415; Tc^r	8
pSmeYZ	pRK415 with an intact *smeYZ* genes; Tc^r	This study
$pEntS_{xylE}$	pRK415 with a 446-bp DNA fragment upstream from the *entS* start codon and a P_{entS}::*xylE* transcriptional fusion	This study
$pSmeU1_{xylE}$	pRK415 with a 570-bp DNA fragment upstream from the *smeU1* start codon and a P_{smeU1}::*xylE* transcriptional fusion	9
pΔYZ	pEX18Tc with an internal-deletion *smeYZ* genes; Tc^r	2
pΔDEF	pEX18Tc with an internal-deletion *smeDEF* genes; Tc^r	4
pΔSbiAB	pEX18Tc with an internal-deletion *sbiAB* genes; Tc^r	This study
pΔEnt	pEX18Tc with an internal-deletion *entFA* genes; Tc^r	3
pΔFur	pEX18Tc with an internal-deletion *fur* gene; Tc^r	3

1. Hu RM, Huang KJ, Wu LT, Hsiao YJ, Yang TC. 2008. Induction of L1 and L2 β-lactamases of *Stenotrophomonas maltophilia*. Antimicrob Agents Chemother 52:1198-1200.
2. Lin YT, Huang YW, Chen SJ, Chang CW, Yang TC. 2015. The SmeYZ efflux pump of *Stenotrophomonas maltophilia* contributes to drug resistance, virulence-related characteristics, and virulence in mice. Antimicrob Agents Chemother 59:4067-4073.
3. Liao CH, Chen WC, Li LH, Lin YT, Pan SY, Yang TC. 2020. AmpR of *Stenotrophomonas maltophilia* is involved in stenobactin synthesis and enhanced β-lactam resistance in an iron-depleted condition. J Antimicrob Chemother 75:3544-3551.
4. Huang YW, Lin CW, Ning HC, Lin YT, Chang YC, Yang TC. 2017. Overexpression of SmeDEF efflux pump decreases aminoglycoside resistance in *Stenotrophomonas maltophilia*. Antimicrob Agents Chemother 61:e02685-16.
5. Simon R, O'Connell M, Labes M, Puhler A. 1986. A. Plasmid vector for the genetic analysis and manipulation of rhizobia and other Gram-negative bacteria. Methods Enzymol 118:640-659.
6. Hoang TT, Karkhoff-Schweizer RR, Kutchma AJ, Schweizer HP. 1998. A broad-host-range Flp-FRT recombination system for site-specific excision of chromosomally-located DNA sequences: application for isolation of unmarked *Pseudomonas aeruginosa* mutants. Gene 212:77-86.

7. Keen NT, Tamaki S, Kobaysahi D, Trollinger D. 1998. Improved broad host-range plasmids for DNA cloning in gram-negative bacteria. Gene 70:191-197.
8. Huang YW, Liou RS, Lin YT, Huang HH, Yang TC. 2014. A linkage between SmeIJK efflux pump, cell envelope integrity, and σE-mediated envelope stress response in *Stenotrophomonas maltophilia*. PLoS One 9:e111784.
9. Chen CH, Huang CC, Chung TC, Hu RM, Huang YW, Yang TC. Contribution of resistance-nodulation division efflux pump operon *smeU1-V-W-U2-X* to multidrug resistance of *Stenotrophomonas maltophilia*. Antimicrob Agents Chemother 55:5826–5833.

Table S3 PCR primers used in this study

Primer	Sequence (5’→3’)	Purpose	Reference
SbiAN-F	GATCTAGACCAGTGACGCCA	pΔSbiAB construction	This study
SbiAN-R	CAAAGCTTGTGACCGCAGAA		
SbiBC-F	CTGGGGGTACCGTTCCTGTTC	pΔSbiAB construction	This study
SbiBC-R	GTGAATTCAGGTGGTGGACA		
EntSN-F	TGGAGCTCGTACGGCCCGAT	pEntS$_{xylE}$ construction	This study
EntSN-R	CGTCTAGAGCAGCCAACACCA		
SmeYZ-F	CATTCTAGACGATCAGGACG	pSmeYZ construction	This study
SmeYZ-R	CTGGAGCTCATGCAGGTCTT		
EntCQ71-F	GACTATGCGTCAGCGGTT	qRT-PCR	This study
EntCQ71-R	ACCACCTTCTGGAGGTTCAGT		
FepAQ97-F	AACCGCATGTACCGCAAC	qRT-PCR	This study
FepAQ97-R	AGTTGTTGACCGCCTCCA		
SpaAQ93-F	GCTTGGCCTATGAGAAGCAG	qRT-PCR	This study
SpaAQ93-R	ATGAGGTCGAGTCCATCAGC		
0795Q93-F	CCTGCTCAGCAAACTGGTCT	qRT-PCR	This study
0795Q93-R	AGCACATTGGTATCGGTGGT		
2858Q96-F	AGCCTGTCCAACATCGAATC	qRT-PCR	This study
2858Q96-R	GGTGCTGAAGTTGATGATGC		
SmeBQ198-F	CGCCATCTCGCTGCTGTTC	qRT-PCR	1
SmeBQ198-R	ATGCCGTTCTTCGCTGCC		
SmeEQ108-F	TCAAGCCGCTGAAGAAGG	qRT-PCR	This study
SmeEQ108-R	GCTGGTAGCTTTCGCTGGT		
SmeHQ207-F	GGCTACTCGGCGATCAAC	qRT-PCR	1

SmeHQ207-R	CAGGCACAGGAACACCAC		
SmeJQ96-F	CGATGATGTGCGCCTACCT	qRT-PCR	This study
SmeJQ96-R	CGTCCACAGGTTCTGTTTGC		
SmeNQ198-F	CAAGACCTCCACTGCCAAC	qRT-PCR	1
SmeNQ198-R	AACAGCCAGATCACCGCC		
SmePQ114-F	CTTCGACTACGCTGTGATGC	qRT-PCR	This study
SmePQ114-R	ATCCACACCTTCTCGTCCTG		
SmeWQ103-F	CCGTGTATGACACCACCATC	qRT-PCR	This study
SmeWQ103-R	AGGAACAGGATCACCACCAG		
EmrBQ107-F	GCATATCTCGGCCTATCAGC	qRT-PCR	This study
EmrBQ107-R	CCTGGTGGTTGATCATGTTG		
1444Q116-F	GGACCACATGATCACCTTCC	qRT-PCR	This study
1444Q116-R	CGCTGGAATACAGCTGCTC		
MacBQ107-F	CAGTTCTTCCGGGTCAAGG	qRT-PCR	This study
MacBQ107-R	GTCTGGGTGTTCTCGTCGAT		
SbiBQ105-F	GCAGGTCGAGTCACAGATCA	qRT-PCR	This study
SbiBQ105-R	ACGACCAGCGTTTCAATCTC		
rDNA-F	GACCTTGCGCGATTGAATG	qRT-PCR	1
rDNA-R	CGGATCGTCGCCTTGGT		

1. Chen CH, Huang CC, Chung TC, Hu RM, Huang YW, Yang TC. Contribution of resistance-nodulation division efflux pump operon *smeU1-V-W-U2-X* to multidrug resistance of *Stenotrophomonas maltophilia*. Antimicrob Agents Chemother 55:5826–5833.
